# A General Catalyst Controlled Route to Prostaglandin F2_α_

**DOI:** 10.1021/acs.orglett.2c03718

**Published:** 2022-11-29

**Authors:** Laura Cunningham, Sourabh Mishra, Leon Matthews, Stephen P. Fletcher

**Affiliations:** Department of Chemistry, Chemistry Research Laboratory, University of Oxford, Oxford OX1 3TA, U.K.

## Abstract

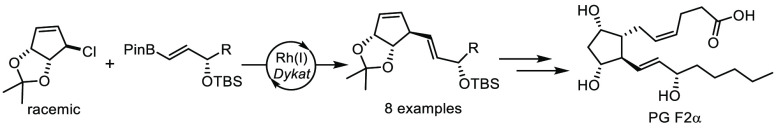

We report a general, catalyst-controlled route to prostaglandin F2_α_ and its analogues. The approach uses a Rh-catalyzed dynamic kinetic asymmetric Suzuki–Miyaura coupling reaction between a racemic bicyclic allyl chloride and alkenyl boronic esters bearing chiral alcohols to give cyclopentyl intermediates bearing 3 contiguous stereocenters. The route provides advanced intermediates in 99% ee as a single diastereoisomer in all cases examined, with the absolute stereochemistry of the cyclopentane core controlled by the ligand. Intermediates that could be used to produce prostaglandin analogues such as bimatoprost, latanoprost, fluprostenol, and cloprostenol were synthesized. The final two stereocenters were installed via Pd-catalyzed Tsuji–Trost alkylation and iodolactonization. The synthesis of PG F2_α_ was achieved in 19% yield in 16 longest linear steps.

Prostaglandins (PGs) play a key role in the regulation of pain and inflammatory responses in the body, and multiple PG analogues feature in the WHO’s list of essential medicines. Their synthesis is an academic challenge which has been investigated for decades, and there is significant industrial and medical relevance in developing new approaches for their synthesis.

The PG F2_α_ family features a cyclopentyl core with 4 contiguous stereocenters and 2 aliphatic side chains where the bottom chain bears a stereogenic allyl alcohol. Corey’s landmark synthesis of PG F2_α_ and PG E_2_ was an exemplary approach to prostaglandins,^1^ with many modern routes to prostaglandins converging on the key Corey lactone intermediate of the route described over 50 years ago (Figure 1a).^2−5^ Although many stereospecific and diastereoselective approaches to prostaglandins have been described, often employing enantiopure starting materials or the use of stoichiometric chiral reagents,^6−8^ few catalytic asymmetric approaches exist where a catalyst exclusively controls the configuration of the product.

**Figure 1:**
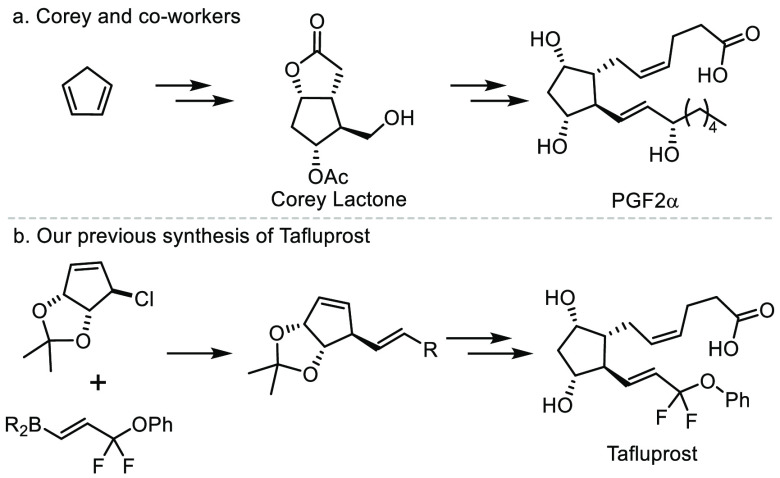
(a) Corey’s original synthesis of PGF2_α_. (b). Our SMC approach to tafluprost.

While the earliest catalytic asymmetric syntheses were pioneered by Corey,^9^ modern methods have enabled a broader array of approaches to the asymmetric synthesis of prostaglandins. Asymmetric allylic alkylation,^10,11^ cyclizations,^12,13^ 1,4-additions/aldol cascades,^14,15^ as well as aldol and Michael reactions have all found success in the synthesis of PG derivatives.^16,17^ Previously, we reported an asymmetric Suzuki–Miyaura coupling (SMC) approach to the PG analogue tafluprost, coupling two complex parts, a racemic bicyclic allyl chloride and an alkenyl boronic acid (Figure 1b).^18^ However, that work did not examine the applicability of *sp*^3^–*sp*^2^ SMC to diastereoselective reactions in which an enantiopure boronic ester is employed, which would be necessary to access PG F2_α_ and the vast majority of marketed PG analogues which possess a chiral allylic alcohol on the bottom chain.^19^ We have recently shown that the allyl chloride in this work can be applied in a variety of asymmetric SMCs,^20^ including on a 100 g scale.^21^

Here, the enantioselective synthesis of alkenyl boronic esters possessing protected allylic alcohols, followed by their diastereoselective *sp*^3^–*sp*^2^ SMC, affords densely functionalized cyclopentenes which could serve as intermediates for a broad range of PGs and analogues (Figure 2).

**Figure 2:**
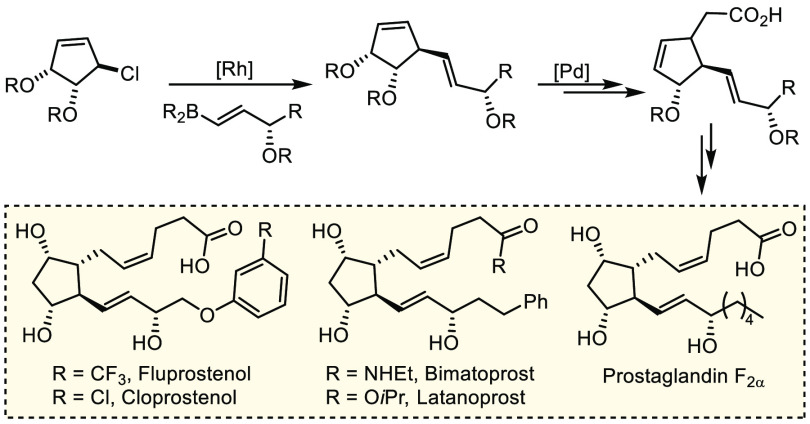
Proposed synthetic approach to PG F2_α_ and commercially available prostaglandin analogues. A selection of targets potentially accessible via this route is shown.

Starting from commercially available carboxylic acids **1**, the stereocontrolled synthesis of alkenyl boronic esters **4a**–**4h** bearing protected allylic alcohols was carried out in 6 steps (Scheme 1). Weinreb amide formation followed by the addition of TIPS acetylene provided a small series of ketones **2**. Asymmetric reduction using Noyori’s catalyst gave the desired enantiomer of alcohols **3**, and subsequent alkyne deprotection, alcohol protection, and borylation were used to access boronic esters **4a**–**4h** in all cases with 99% ee.

**Scheme 1:**
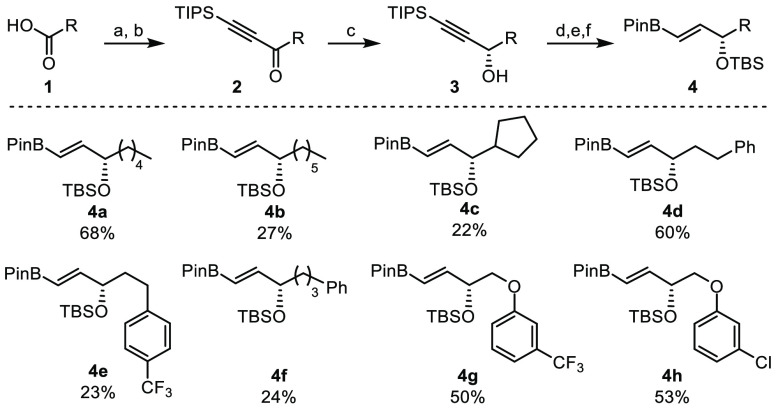
Synthesis of Alkenyl Boronic Esters Bearing Enantiomerically Enriched Allyl Halides Reagents and conditions: (a) *N*,*O*-dimethylhydroxylamine.HCl, EDC, DMAP, rt, 1 h; (b) TIPS acetylene, *n*-BuLi, THF, 0 °C; (c) RuCl[(*S*,*S*)-TsDPen](mesitylene), IPA, rt, 10 min; (d) TBAF, THF, 1 h; (e) TBSCl, imidazole, DMAP, DCM, 0 °C, 1 h; (f) 4-methylaminobenzoic acid, HBPin, heptane, 110 °C, 16 h. Yields shown are overall yields over 6 steps from carboxylic acids **1**.

The key consideration of this work is that the alkenyl boronic ester possesses an adjacent stereogenic alcohol. Unlike our previous synthesis of tafluprost, which employs an achiral boronic ester, significant issues could arise in this system due to competitive substrate control when using chiral nucleophiles. The addition of **4** to allyl chloride (*rac*)**-5** using (*rac*)-BINAP provided **6** with 88% conversion as a 1:1 mixture of 2 diastereomers (of 4 possible, see SI Figure S1), with both observed isomers possessing *cis*,*trans* relative stereochemistry in the cyclopentene core. Addition using (*S*)-BINAP gave **6** in ∼20:1 dr, and (*R*)-BINAP also gave an ∼1:20 mixture of isomers in favor of the other *cis*,*trans*-diasteroisomer (SI Figure S1). We found that (*S*)-DM Segphos is capable of providing desired **6** in 90% isolated yield as a single diastereoisomer as measured by ^1^H NMR spectroscopy on the crude reaction mixture.

In our previous work, 90% ee was achieved with 7:1 dr, where the minor diastereoisomer was the *cis,cis*-cyclopentene product. Here, it appears that the proximity of the sterically demanding TBS group improves the dr with respect to the relative stereochemistry of the cyclopentene core during C–C bond formation. A *cis*,*trans*-conformation is necessarily adopted, likely due to repulsion between the bulky nucleophile and the acetonide. While the relative stereochemistry about the cyclopentene core is substrate controlled, the absolute configuration about the core is determined by which enantiomer of ligand is used (SI Figure S2). As two different catalyst-controlled stereochemistry determining steps are used in this sequence the final product is expected to have an enantiomeric excess beyond the limits of standard detection methods. The use of these powerful catalyst-controlled steps to set the configuration of the side chain and core also offers the opportunity to access unnatural prostaglandin stereoisomers if desired.

In terms of generality of the route, a variety of boronic esters can be used to give products **6**–**13**. All of the substrates proceeded with >90% conversion and led to only a single detectable stereoisomer (Scheme 2). Products **6** (90% yield) and **8** (91% yield) are potentially precursors of PG F2_α_ and a cyclic analogue, respectively. Bimatoprost and latanoprost can theoretically be accessed from **9** which was isolated in 93% yield, and related analogues **10** and **11** were isolated in 79% and 82% yield, respectively. Boronic esters **4g** and **4h** gave **12** and **13** (85% and 94%) which are potentially intermediates toward travoprost, fluprostenol, and cloroprostenol.

**Scheme 2:**
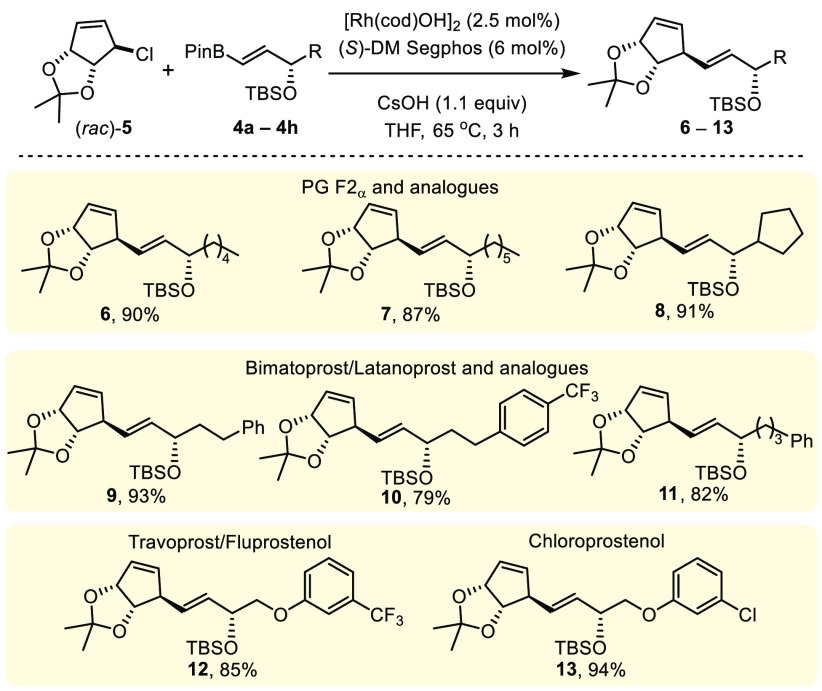
A Range of Alkenylboronic Esters **4** Can Yield Potential Precursors to Multiple PG Analogues Reactions carried out using 0.5 mmol (*rac*)-**5** and 0.6 mmol **4**. Compound **6** prepared using 5 mmol of *(rac)-***5** and 6 mmol of **4**. In each case only a single diastereoisomer was observed in the crude reaction mixture by ^1^H NMR spectroscopic analysis.

To establish the scalability and robustness of this method, the synthesis of **6** was conducted on a 5 mmol scale, which proceeded to give a single isomer of product in 90% yield. Overall, this approach sets three contiguous stereocenters in the cyclopentyl core in a single step with the required configuration to prepare PG F2_α_.

Compound **6** was used to make prostaglandin F2_α_ (Scheme 3). Acetic acid mediated global deprotection of **6** led to the corresponding triol in 79% yield. Formation of carbonate **14** was not as straightforward as in the synthesis of tafluprost—likely a consequence of the unprotected allylic alcohol. Reaction with triphosgene gives a complex mixture of products. CDI was found to be a suitable carbonyl synthon, affording **14** in 94% yield after purification.

**Scheme 3:**
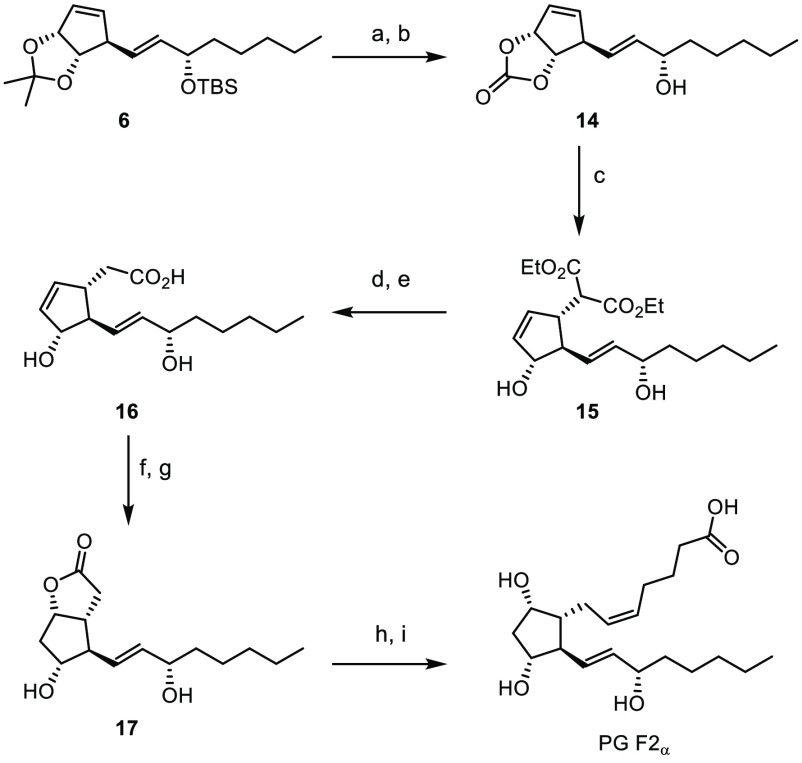
Synthesis of PG F2_α_ Reagents and conditions: (a) AcOH/H_2_O, 16 h, 79%; (b) CDI, Et_3_N, DCM/MeOH, 50 °C, 4 h, 94%; (c) diethyl malonate, [Pd(dppf)Cl_2_]_2_ (3 mol %), THF, rt, 1 h, 96%; (d)d] NaOH, THF/H_2_O, rt, 1 h; (e) CDI, THF, rt, 2 h, then 1 M NaOH, rt, 16 h; (f)] KI, I_2_, NaHCO_3_, THF/H_2_O, 0 °C to rt, 48 h, 69% over 3 steps; (g)] Bu_3_SnH, AIBN, C_6_H_6_, 80 °C, 1 h, 78%; (h) DIBAL-H, DCM, −78 °C to rt, 1 h; (i)] (4-carboxybutyl)triphenylphosphonium bromide, KHMDS, THF/PhMe, 0 °C, 1 h, 82% over two steps. Abbreviations: dppf, 1,1′- bis(diphenylphosphino)ferrocene; rt, room temperature; CDI, 1,1′-carbonyldiimidazole; AIBN, azobis(isobutyronitrile); DIBAL-H, diisobutylaluminum hydride; KHMDS, potassium bis(trimethylsilyl) amide.

It is noteworthy that the presence of the unprotected allylic alcohol did not interfere with the Tsuji–Trost alkylation and addition of dimethyl malonate gave **15** in 96% yield as a single diastereoisomer. Ester hydrolysis and decarboxylation provided **16**, and subsequent iodolactonization led to the desired product in 69% yield over 3 steps. Tributyl tin hydride mediated dehalogenation enabled access to lactone **17**. NMR spectroscopic analysis and optical rotation of **17** confirmed the absolute and relative stereochemistry.^22−24^ The reduction of **17** gives rise to a hemiacetal which was used without purification in a *Z-*selective Wittig olefination to yield prostaglandin F2_α_ in 82% over two steps (Scheme 3).

In summary, an asymmetric total synthesis of prostaglandin F2_α_ in 19% overall yield was achieved in 16 steps in the longest linear sequence. Our approach uses a catalyst-controlled diastereoselective Rh-catalyzed Suzuki–Miyaura reaction between two complex coupling partners. This method sets 3 contiguous stereocenters in a single step to provide the cyclopentyl core with complete ligand control over absolute stereochemistry. The use of alkenyl boronic esters with protected allylic alcohols allows access to coupling products which may serve as precursors for multiple naturally occurring prostaglandins and synthetic analogues including bimatoprost, latanoprost, travoprost, fluprostenol, and cloprostenol.

## Notes

The data underlying this study are available in the published article and its online Supporting Information.

## Supporting Information Available

Detailed descriptions of synthetic procedures, characterization data, and NMR spectra (PDF)
