# Supplementary material for: A General Catalyst Controlled Route to Prostaglandin F2α
Source: Org Lett. 2022 Nov 29;24(48):8886–9. doi: 10.1021/acs.orglett.2c03718 (PMC9745796; doi:10.1021/acs.orglett.2c03718)
Supplement: Supplementary file 1 — ol2c03718_si_001.pdf [file ol2c03718_si_001.pdf]

A General Catalyst Controlled Route to Prostaglandin F2$_{\alpha}$

*Laura Cunningham, Sourabh Mishra, Leon Matthews, Stephen P. Fletcher**

stephen.fletcher@chem.ox.ac.uk

Department of Chemistry, Chemistry Research Laboratory, University of Oxford, Oxford, OX1 3TA, UK

Table of Contents

1. **Supplementary Figures**……………………………………………………………S3
2. **Experimental procedures**…………………………………………………………S4
3. **NMR spectra**………………………………………………………………………S36
4. **SFC Traces**………………………………………………………………………S90
5. **References**………………………………………………………………………..S98

Supplementary Figures

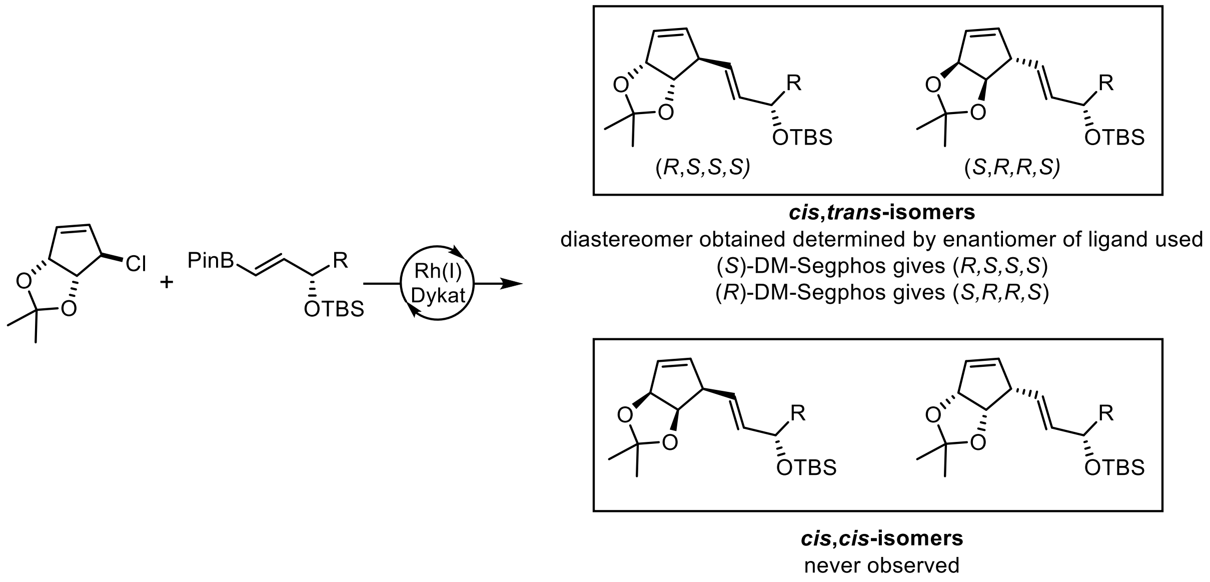

Figure S1. Structure of four possible diastereomers obtained from the reaction between (*rac*)-**5** and enantiopure Alkenyl boronic esters **4**.

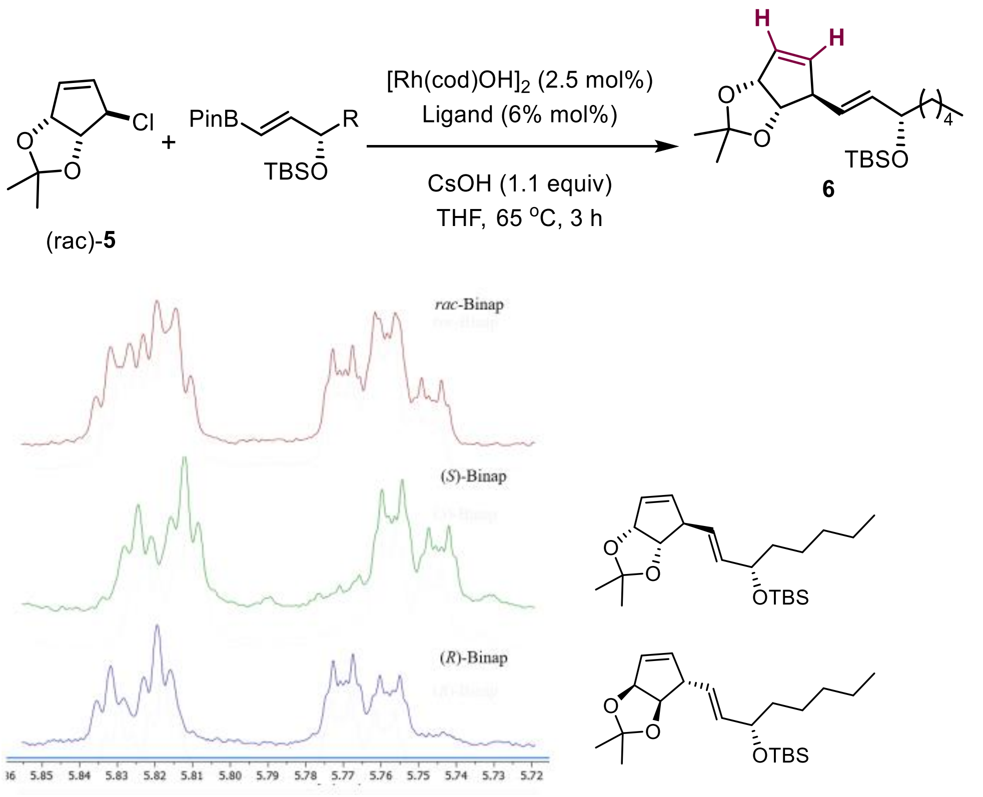

Figure S2. Comparison of ^{1}H NMR spectra of crude reaction mixture when employing *rac*-, (*S*)- or (*R*)-BINAP, signals shown correspond to the C-H signals of the alkene of the cyclopentene ring.

Experimental procedures

X.X General Methods

Procedures using oxygen and/or moisture-sensitive materials were performed with anhydrous solvents (vide infra) under an atmosphere of anhydrous argon in flame-dried flasks, using standard Schlenk techniques. Analytical thin-layer chromatography was performed on precoated glass-backed plates (Silica Gel 60 F254; Merck) and visualised using a combination of UV light (254 nm) and aqueous basic potassium permanganate stain. Flash column chromatography was carried out using Merck 60 Å silica gel.

Nuclear magnetic resonance (NMR) spectroscopy measurements were carried out at room temperature. ^{1}H NMR, ^{13}C NMR, COSY, and HSQC were carried out using Bruker AVB-400 (400/100 MHz), Bruker AVG-400 (400/100 MHz) and AVH-400 (400/100 MHz) spectrometers. Chemical shifts (δ) are reported in ppm relative to the residual solvent peak with corresponding coupling constants (*J*) in Hertz (Hz) and multiplicities (s: singlet, d: doublet, t: triplet, q: quartet, br s: broad singlet, qt: quartet of triplet, qd: quartet of doublet, dtt: doublet of triplet of triplet)

Infrared (IR, neat or thin film) spectroscopy was carried out on a Bruker Tensor 27 FT–IR spectrometer within internal calibration range of 4000 – 600 cm^{-1}. The samples are reported as absorption maxima in cm^{-1}.

Chiral SFC (supercritical fluid chromatography) separations were conducted on a Waters Acquity UPC2 system using Waters Empower software. Chiralpak® columns (150×3 mm, particle size 3 µm) were used as specified in the text. Solvents used were of HPLC grade (Fisher Scientific, Sigma Aldrich or Rathburn).

High Resolution Mass spectra were carried out by internal service at the University of Oxford. Electron spray ionisation (ESI+) were recorded on a Thermo Exactive with an orbitrap ion analyser.

Commercially available reagents and ligands were purchased from Sigma Aldrich, Alfa Aesar, Acros Organics, Fluorochem and Strem Chemicals and unless otherwise stated were used without further purification. Dry and deuterated solvents were purchased from Sigma Aldrich.

General procedure A: Synthesis of Weinreb Amides

Carboxylic acid (1 equiv) was dissolved in DCM (0.3M) at 0 ˚C followed by the addition of N,O-dimethylhydroxylamine·HCl (1.2 equiv), EDC(1.2 equiv), DMAP(0.1 equiv) and triethyl amine (1.2 equiv). The reaction was warmed to room temperature and stirred for one hour. The mixture was washed with 0.5M HCl and saturated aq. NaHCO3. The aqueous phase was extracted with DCM before drying with NaSO4 and concentrating under vacuum. The crude Weinreb amide was obtained in sufficient purity for subsequent reaction. In some cases, contamination with dimethylhydroxylamine is observed in the ^{1}H NMR of the crude product – this is accounted for in the calculated yields, and has no observed negative affect in subsequent reactions.

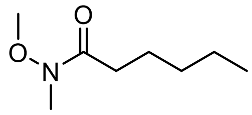

Weinreb amide **S1a** was prepared according to general procedure A from hexanoic acid (12.5 mL, 1 equiv, 100 mmol) in a 93% yield (14.8 g) as colourless oil and used without further purification.

^{1}H NMR (400 MHz, $CDCl_3$) δ 3.68 (s, 3H), 3.18 (s, 3H), 2.41 (t, *J* = 7.7 Hz, 2H), 1.69 – 1.55 (m, 4H), 1.33 (q, *J* = 3.6 Hz, 4H), 0.90 (td, *J* = 6.5, 2.5 Hz, 3H).

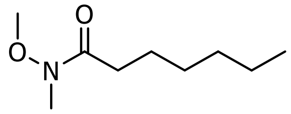

Prepared according to general procedure A from the corresponding carboxylic acid (7 mL, 1 equiv, 50 mmol), Weinreb amide **S1b** was isolated in 85% yield (7.36 g) as colourless oil.

^{1}H NMR (400 MHz, $CDCl_3$) δ 3.64 (s, 3H), 3.14 (s, 3H), 2.43 – 2.33 (m, 2H), 1.63 – 1.55 (m, 2H), 1.37 – 1.19 (m, 6H), 0.90 – 0.81 (m, 3H).

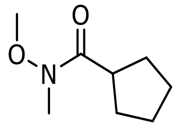

Prepared according to general procedure A from the corresponding carboxylic acid (5.4 mL, 1 equiv, 50 mmol), Weinreb amide **S1c** was isolated in 61% yield (4.79 g) as colourless oil.

^{1}H NMR (400 MHz, $CDCl_3$) δ 3.65 (s, 3H), 3.14 (s, 3H), 3.10 – 2.98 (m, 1H), 1.88 – 1.61 (m, 6H), 1.57 – 1.48 (m, 2H).

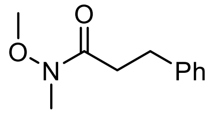

Weinreb amide **S1d** was prepared according to general procedure A from the corresponding carboxylic acid (5.3 g, 1 equiv, 35 mmol) in a 81% yield (5.4 g) as colourless oil.

^{1}H NMR (400 MHz, $CDCl_3$) δ 7.25 (qt, *J* = 15.8, 7.2 Hz, 5H), 3.61 (s, 3H), 3.18 (s, 3H), 2.96 (dd, *J* = 9.0, 6.9 Hz, 2H), 2.84 – 2.70 (m, 2H).

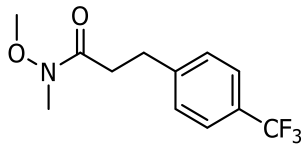

Prepared according to general procedure A from the corresponding carboxylic acid (5.45 g, 1 equiv, 25 mmol), Weinreb amide **S1e** was isolated in 88% yield (5.45 g) as colourless oil.

^{1}H NMR (400 MHz, $CDCl_3$) δ 7.52 (d, *J* = 8.0 Hz, 2H), 7.33 (d, *J* = 8.0 Hz, 2H), 3.61 (s, 3H), 3.16 (s, 3H), 3.01 (t, *J* = 7.7 Hz, 2H), 2.75 (t, *J* = 7.7 Hz, 2H).

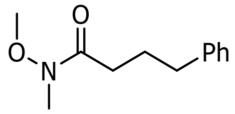

Prepared according to general procedure A from the corresponding carboxylic acid (4.1 g, 1 equiv, 25 mmol), Weinreb amide **S1f** was isolated in 73% yield (3.8 g) as colourless oil.

^{1}H NMR (400 MHz, $CDCl_3$) δ 7.35 – 7.26 (m, 2H), 7.26 – 7.16 (m, 3H), 3.64 (s, 3H), 3.19 (s, 3H), 2.71 (t, *J* = 7.6 Hz, 2H), 2.47 (t, *J* = 7.3 Hz, 2H), 2.06 – 1.93 (m, 2H).

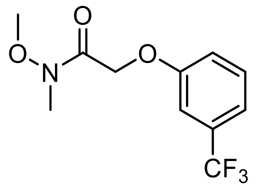

Weinreb amide **S1g** was prepared according to general procedure A from the corresponding carboxylic acid (5.5 g, 1 equiv, 25 mmol) in a 93% yield (6.2 g) as colourless oil.

^{1}H NMR (400 MHz, $CDCl_3$) δ 7.48 – 7.34 (m, 1H), 7.26 (s, 1H), 7.20 – 7.15 (m, 1H), 7.15 – 7.08 (m, 1H), 4.86 (s, 2H), 3.78 (s, 3H), 3.25 (s, 3H).

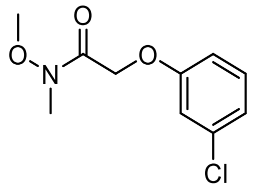

Weinreb amide **S1h** was prepared according to general procedure A from the corresponding carboxylic acid (6.1 g, 1 equiv, 33 mmol) in a 98% yield (7.4 g) as colourless oil.

^{1}H NMR (400 MHz, $CDCl_3$) δ 7.21 (dd, *J* = 8.6, 1.7 Hz, 1H), 7.03 – 6.92 (m, 2H), 6.91 – 6.82 (m, 1H), 4.82 (s, 2H), 3.93 – 3.54 (s, 3H), 3.26 (s, 3H).

General procedure B: Addition of TIPS acetylene to Weinreb amides

TIPS acetylene (1.2 equiv) was dissolved in anhydrous THF and cooled to 0 ˚C before the addition of *n*-BuLi (1.2 equiv, 2.5 M in hexanes) under inert atmosphere. This was stirred for 15 min, followed by the addition of neat Weinreb amide (1 equiv). The reaction was quenched after 30 min by the addition of sat. aq. NH_4Cl. The aqueous layer was extracted with Et_2O, and the combined organic layers were dried over Na_2SO_4, filtered and concentrated under vacuum. The resulting oil was purified via column chromatography to yield the product ketone.

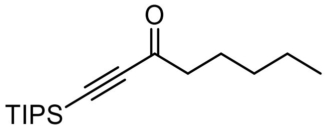

Ketone **2a** was prepared according to general procedure B from crude Weinreb amide **S1** (8 g, 50 mmol) in 86% yield (11.9 g) as colourless oil. Column conditions: pure hexane.

^{1}H NMR (400 MHz, $CDCl_3$) δ 2.55 (t, *J* = 7.4 Hz, 2H), 1.70 (tt, *J* = 7.4, 6.1 Hz, 2H), 1.33 – 1.31 (m, 4H), 1.20 – 1.02 (m, 21H), 0.94 – 0.84 (m, 3H).

^{13}C NMR (101 MHz, $CDCl_3$) δ 188.1, 104.2, 95.4, 45.6, 31.1, 24.0, 22.4, 18.5, 13.9, 11.0.

IR (neat) 2954, 2867, 2360, 1680, 1464, 882 cm^{-1}.

HRMS (ESI) m/z: $[M + Na]^+$ Calcd for $C_{17}H_{32}OSiNa$ 302.2115; Found 302.2112.

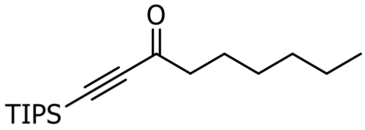

Ketone **2b** was prepared according to general procedure B from crude Weinreb amide **S1b** (42.6 mmol) in 78% yield (9.74 g) as colourless oil. Column conditions: pure hexane.

^{1}H NMR (400 MHz, $CDCl_3$) δ 2.54 (t, *J* = 7.4 Hz, 2H), 1.74 – 1.62 (m, 2H), 1.40 – 1.21 (m, 6H), 1.09 (s, 21H), 0.90 – 0.82 (m, 3H).

^{13}C NMR (101 MHz, $CDCl_3$) δ 188.0, 104.3, 95.2, 45.6, 31.5, 28.6, 24.3, 22.4, 18.4, 14.0, 11.0.

IR (neat) 2946, 2868, 1680, 1464, 1213, 1134, 1086, 997, 920, 883 cm^{-1}.

HRMS (ESI) m/z: $[M + H]^+$ Calcd for $C_{18}H_{35}OSi$ 295.2452; Found 295.2453.

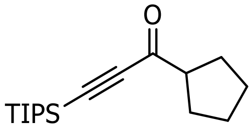

Ketone **2c** was prepared according to general procedure B from crude Weinreb amide **S1** (30.2 mmol) in 96% yield (8.07 g) as colourless oil. Column conditions: 0-2% diethylether in hexane.

^{1}H NMR (400 MHz, $CDCl_3$) δ 2.91 (tt, *J* = 8.7, 7.2 Hz, 1H), 2.02 – 1.82 (m, 4H), 1.74 – 1.53 (m, 4H), 1.10 (s, 21H).

^{13}C NMR (101 MHz, $CDCl_3$) δ 190.7, 103.6, 95.8, 53.8, 29.1, 26.1, 18.5, 11.0.

IR (neat) 2946, 2868, 1675, 1464, 1219, 1118, 1061, 997, 921, 883 cm^{-1}.

HRMS (ESI) m/z: $[M + H]^+$ Calcd for $C_{17}H_{31}OSi$ 279.2139; Found 279.2140.

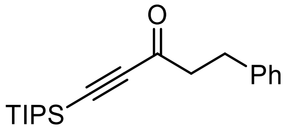

Ketone **2d** was prepared according to general procedure B from crude Weinreb amide **S1** (5 g, 24 mmol) in 100% yield (7.8 g) as colourless oil. Column conditions: pure hexane.

^{1}H NMR (400 MHz, $CDCl_3$) δ 7.35 – 7.25 (m, 2H), 7.25 – 7.17 (m, 3H), 3.06 – 2.97 (m, 2H), 2.91 (ddd, *J* = 8.3, 6.8, 1.1 Hz, 2H), 1.21 – 1.03 (m, 21H).

^{13}C NMR (101 MHz, $CDCl_3$) δ 186.6, 140.2, 128.6, 128.4, 126.3, 104.1, 96.1, 47.3, 30.1, 18.5, 11.0.

IR (neat) 2980, 2889, 2360, 1678, 1462, 679 cm^{-1}.

HRMS (ESI) m/z: $[M + H]^+$ Calcd for $C_{20}H_{31}OSi$ 315.2130; Found 315.2136.

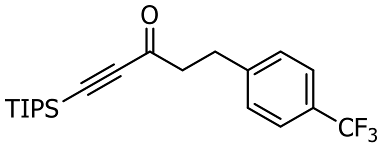

Ketone **2e** was prepared according to general procedure B from crude Weinreb amide **S1e** (21.9 mmol) in 68% yield (5.7 g) as colourless oil. Column conditions: pure hexane.

^{1}H NMR (400 MHz, $CDCl_3$) δ 7.47 (d, *J* = 8.1 Hz, 2H), 7.32 (d, *J* = 8.1 Hz, 2H), 2.99 (t, *J* = 7.5 Hz, 1H), 2.95 – 2.91 (m, 2H), 1.10 (s, 21H).

^{13}C NMR (101 MHz, $CDCl_3$) δ 185.8, 144.4, 128.7, 125.6, 125.5 (q, *J* = 3.7 Hz), 122.9, 104.0, 96.6, 46.6, 29.8, 18.5, 11.0.

^{19}F NMR (376 MHz, $CDCl_3$) δ -62.4.

IR (neat) 2947, 2869, 2360, 1679, 1464, 1326, 1165, 1125, 1068, 1019, 998, 920, 883, 833 cm^{-1}.

HRMS (ESI) m/z: $[M + H]^+$ Calcd for $C_{21}H_{30}F_3OSi$ 383.2013; Found 383.2019.

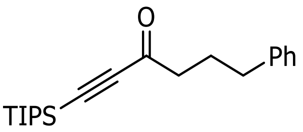

Ketone **2f** was prepared according to general procedure B from crude Weinreb amide **S1f** (18.4 mmol) in 80% yield (4.82 g) as colourless oil. Column conditions: pure hexane.

^{1}H NMR (400 MHz, $CDCl_3$) δ 7.32 – 7.27 (m, 2H), 7.24 – 7.15 (m, 3H), 2.76 – 2.50 (m, 4H), 2.16 – 1.93 (m, 2H), 1.12 (s, 21H).

^{13}C NMR (101 MHz, $CDCl_3$) δ 187.5, 141.3, 128.5, 128.4, 126.1, 104.2, 95.6, 44.9, 34.9, 25.8, 18.5, 11.0.

IR (neat) 2946, 2867, 1677, 1462, 1215, 1108, 1074, 1018, 997, 920, 883, 833 cm^{-1}.

HRMS (ESI) m/z: $[M + H]^+$ Calcd for $C_{21}H_{33}OSi$ 329.2295; Found 329.2301.

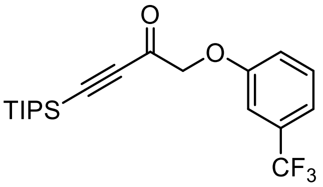

Ketone **2g** was prepared according to general procedure B from crude Weinreb amide **S1g** (7 g, 26 mmol) in 87% yield (8.1 g) as colourless oil. Column conditions: pure hexane.

^{1}H NMR (400 MHz, $CDCl_3$) δ 7.41 (t, J = 8.0 Hz, 1H), 7.26 (t, J = 2.8 Hz, 1H), 7.14 (q, J = 2.8 Hz, 1H), 7.03 (dd, J = 8.0, 2.8 Hz, 1H), 4.73 (s, 2H), 1.19 – 1.02 (m, 21H).

^{13}C NMR (101 MHz, $CDCl_3$) δ 182.6, 157.8, 132.1 (q, J = 32.6 Hz), 130.2, 118.5, 118.5, 117.7, 112.1, 102.0, 101.4, 73.2, 18.4, 10.9.

^{19}F NMR (376 MHz, $CDCl_3$) δ -62.82.

IR (neat) 2948, 2869, 1680, 1326, 1129 cm^{-1}.

HRMS (ESI) m/z: $[M + Na]^+$ Calcd for $C_{20}H_{27}F_3O_2SiNa$ 407.1625; Found 407.1621.

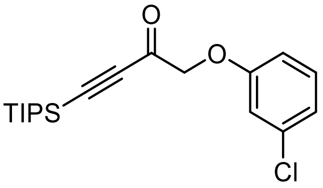

Ketone **2h** was prepared according to general procedure B from crude Weinreb amide **S1h** (3.3 g 14 mmol) in 86% yield (2.8 g) as colourless oil. Column conditions: pure hexane.

^{1}H NMR (400 MHz, $CDCl_3$) δ 7.21 (t, *J* = 8.1 Hz, 1H), 7.01 – 6.94 (m, 1H), 6.92 – 6.85 (m, 1H), 6.76 (ddd, *J* = 8.4, 2.5, 0.9 Hz, 1H), 4.68 (s, 2H), 1.08 (s, 21H).

^{13}C NMR (101 MHz, $CDCl_3$) δ 182.7, 158.4, 135.1, 130.4, 122.0, 115.5, 112.9, 101.8, 101.45, 73.3, 18.5, 10.9.

IR (neat) 2980, 2887, 2360, 1681, 1594, 679 cm^{-1}.

HRMS (ESI) m/z: $[M + H]^+$ Calcd for $C_{19}H_{28}ClO_2Si$ 351.1542; Found 351.1539.

General procedure C: Asymmetric reduction of ketone with Noyori's catalyst

Activation of Noyori's catalyst was carried out immediately prior to use following a procedure from Schiessl et al.[ref]

To a round bottom flask containing the activated RuCl[(S,S)-TsDPEN](mesitylene) (0.25 mol%), under an inert atmosphere, was added neat ketone (1 equiv) followed by the addition of IPA (0.2 M). The resulting orange solution was stirred for 10 min at RT before being concentrated under vacuum and purified by column chromatography to yield the product alcohol.

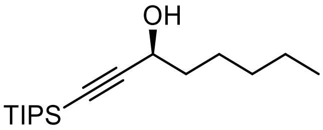

Alcohol **3a** was prepared according to general procedure C from ketone **2a** (17.0 g, 60 mmol) in 99% yield (17.0 g) and 99% ee as colourless oil. Column conditions: 10% diethylether in hexane.

^{1}H NMR (400 MHz, $CDCl_3$) δ 4.39 (t, *J* = 6.3 Hz, 1H), 1.80 – 1.61 (m, 2H), 1.55 – 1.42 (m, 2H), 1.40 – 1.20 (m, 4H), 1.15 – 1.00 (m, 21H), 0.96 – 0.84 (m, 3H).

^{13}C NMR (101 MHz, $CDCl_3$) δ ^{13}C NMR (101 MHz, $CDCl_3$) δ 108.9, 85.4, 63.1, 37.9, 31.4, 24.8, 22.6, 18.6, 13.9, 11.1.

IR (neat) 2943, 2866, 2361, 1464, 1126, 677 cm^{-1}.

HRMS (ESI) m/z: $[M + Na]^+$ Calcd for $C_{17}H_{34}OSiNa$ 305.2271; Found 305.2272.

SFC Conditions: The enantioselectivity was determined using Chiralpak IA; 1500 PSI, 30 °C; flow: 1.5 mL/min; from 1% to 10% MeOH in 10 min; 99.65:0.35 er (major enantiomer t_R = 2.55 min; minor enantiomer t_R = 2.68 min), **99% ee**.

$[\alpha]^{25}_D$ = −8.1 (c=0.36, $CHCl_3$)

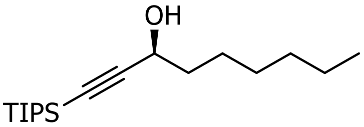

Alcohol **3b** was prepared according to general procedure C from crude ketone **2b** (10 mmol) in 96% yield (2.85 g) and 99% ee as colourless oil. Column conditions: 0-5% diethylether in hexane.

^{1}H NMR (400 MHz, $CDCl_3$) δ 4.37 (q, *J* = 6.3 Hz, 1H), 1.97 (d, *J* = 4.4 Hz, 1H), 1.77 – 1.60 (m, 2H), 1.46 (dt, *J* = 15.6, 7.3 Hz, 2H), 1.38 – 1.19 (m, 6H), 1.06 (s, 21 H), 0.93 – 0.81 (m, 1H).

^{13}C NMR (101 MHz, $CDCl_3$) δ 109.1, 85.4, 63.1, 38.1, 31.9, 29.0, 25.2, 22.7, 18.7, 14.2, 11.3.

IR (neat) 3337, 2943, 2866, 2170, 1465, 1383, 1040, 1016, 997, 920, 884 cm^{-1}.

HRMS (ESI) m/z: $[M + Na]^+$ Calcd for $C_{18}H_{36}OSiNa$ 319.2428; Found 319.2433.

SFC Conditions: The enantioselectivity was determined after Benzoyl protection and TIPS deprotection using Chiralpak IC; 1500 PSI, 30 °C; flow: 1.5 mL/min; from 1% to 30% MeOH in 5 min; 99.5:0.5 er (major enantiomer t_R = 1.36 min; minor enantiomer t_R = 1.45 min), **99% ee**.

[α]^{25}$_D$ = +2.9 (c=1.0, CH_3OH)

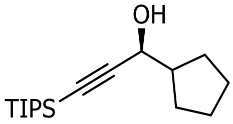

Alcohol **3c** was prepared according to general procedure C from crude ketone **2c** (10 mmol) in 95% yield (2.66 g) and 99% ee as colourless oil. Column conditions: 0-10% diethylether in hexane.

^{1}H NMR (500 MHz, $CDCl_3$) δ 4.44 – 4.15 (m, 1H), 2.26 – 2.08 (m, 1H), 1.86 – 1.70 (m, 2H), 1.68 – 1.36 (m, 6H), 1.06 (s, 21H).

^{13}C NMR (126 MHz, $CDCl_3$) δ 108.4, 85.6, 67.0, 46.4, 29.0, 28.4, 25.99, 25.96, 18.7, 11.3.

IR (neat) 3353, 2945, 2867, 2170, 1464, 1384, 1320, 1038, 997, 921, 884 cm^{-1}.

HRMS (ESI) m/z: $[M + Na]^+$ Calcd for $C_{17}H_{32}OSiNa$ 303.2115; Found 303.2128.

SFC Conditions: The enantioselectivity was determined after Benzoyl protection and TIPS deprotection using Chiralpak IC; 1500 PSI, 30 °C; flow: 1.5 mL/min; from 1% to 30% MeOH in 5 min; 99.75:0.25 er (major enantiomer t_R = 1.55 min; minor enantiomer t_R = 1.69 min), **99% ee**.

[α]^{25}$_D$ = +8.9 (c=1.0, $CHCl_3$)

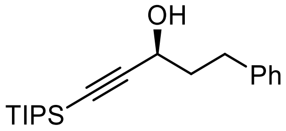

Alcohol **3d** was prepared according to general procedure C from ketone **2d** (4.5 g 14.3 mmol) in 96% yield (4.3 g) and 99% ee as colourless oil. Column conditions: 10% diethylether in hexane.

^{1}H NMR (400 MHz, $CDCl_3$) δ 7.25 – 7.15 (m, 2H), 7.12 (dt, *J* = 8.0, 2.0 Hz, 3H), 4.30 (q, *J* = 6.3 Hz, 1H), 2.73 (t, *J* = 7.9 Hz, 2H), 2.18 – 1.85 (m, 2H), 1.00 (m, 21H).

^{13}C NMR (101 MHz, $CDCl_3$) δ 141.4, 128.5, 128.5, 126.0, 108.5, 86.1, 62.4, 39.6, 31.5, 18.6, 11.2.

IR (neat) 2980, 2866, 2360, 1461, 1383, 741, 678 cm^{-1}.

HRMS (ESI) m/z: $[M + Na]^+$ Calcd for $C_{20}H_{32}OSiNa$ 339.2115; Found 339.2112.

SFC Conditions: The enantioselectivity was determined using Chiralpak IB; 1500 PSI, 30 °C; flow: 1.5 mL/min; from 1% to 10% MeOH in 10 min; 99.84:0.16 er (major enantiomer t_R = 2.46 min; minor enantiomer t_R = 2.78 min), **99% ee**.

[α]^{25}$_D$ = −22.3 (c=0.70, $CHCl_3$)

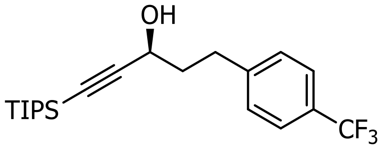

Alcohol **3e** was prepared according to general procedure C from crude ketone **2e** (10 mmol) in 84% yield (3.23 g) and 99% ee as colourless oil. Column conditions: 0-10% diethylether in hexane.

^{1}H NMR (500 MHz, $CDCl_3$) δ 7.54 (d, *J* = 8.0 Hz, 2H), 7.32 (d, *J* = 8.0 Hz, 2H), 4.40 (q, *J* = 6.1 Hz, 1H), 2.89 (t, *J* = 7.9 Hz, 2H), 2.10 – 1.96 (m, 2H), 1.09 (s, 21H).

^{13}C NMR (126 MHz, $CDCl_3$) δ 145.7, 128.9, 128.5 (q, *J* = 32.6 Hz), 125.5 (q, *J* = 3.8 Hz), 123.4, 108.3, 86.5, 62.2, 39.3, 31.4, 18.7, 11.3.

^{19}F NMR (376 MHz, $CDCl_3$) δ -62.4.

IR (neat) 3354, 2945, 2867, 1327, 1166, 1128, 1069, 1019, 884, 844 cm^{-1}.

HRMS (ESI) m/z: $[M + Na]^+$ Calcd for $C_{21}H_{31}F_3OSiNa$ 407.1989; Found 407.1972.

SFC Conditions: Chiralpak IG; 1500 PSI, 30 °C; flow: 1.5 mL/min; hold 1% MeOH for 3 min, then from 1% to 10% MeOH in 5 min; 99.65:0.35 er (major enantiomer t_R = 6.55 min; minor enantiomer t_R = 6.13 min), **99% ee**.

$[\alpha]^{25}_D$ = +27.6 (c=1.0, $CHCl_3$)

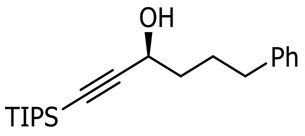

Alcohol **3f** was prepared according to general procedure C from crude ketone **2f** (10 mmol) in 88% yield (2.91 g) and 99% ee as colourless oil. Column conditions: 0-10% diethylether in hexane.

^{1}H NMR (500 MHz, $CDCl_3$) δ 7.31 (q, *J* = 7.9 Hz, 2H), 7.25 – 7.19 (m, 3H), 4.45 (q, *J* = 5.9 Hz, 1H), 2.71 (t, *J* = 7.5 Hz, 2H), 2.08 – 1.98 (m, 1H), 1.92 – 1.69 (m, 4H), 1.12 (s, 21H).

^{13}C NMR (126 MHz, $CDCl_3$) δ 142.2, 128.5, 128.4, 125.9, 108.8, 85.8, 63.0, 37.6, 35.6, 27.0, 18.7, 11.3.

IR (neat) 3345, 2944, 2866, 1463, 1073, 998, 919, 883 cm^{-1}.

HRMS (ESI) m/z: $[M + Na]^+$ Calcd for $C_{21}H_{34}OSiNa$ 353.2271; Found 353.2275.

SFC Conditions: Chiralpak IB; 1500 PSI, 30 °C; flow: 1.5 mL/min; from 1% to 30% MeOH in 5 min; 99.5:0.5 er (major enantiomer t_R = 2.60 min; minor enantiomer t_R = 2.53 min), **99% ee**.

$[\alpha]^{25}_D$ = +20.8 (c=1.0, $CHCl_3$)

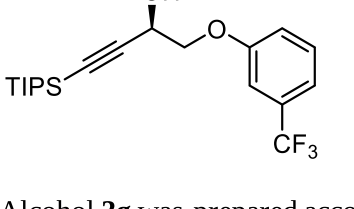

Alcohol **3g** was prepared according to general procedure C from crude ketone **2g** (3.3 g, 8.5 mmol) in 85% yield (2.8 g) and 99% ee as colourless oil. Column conditions: 10% diethylether in hexane.

^{1}H NMR (400 MHz, $CDCl_3$) δ 7.34 (t, *J* = 8.0 Hz, 1H), 7.17 (d, *J* = 7.2 Hz, 1H), 7.10 (t, *J* = 2.1 Hz, 1H), 7.04 (dd, *J* = 8.3, 2.6 Hz, 1H), 4.77 – 4.69 (m, 1H), 4.11 (dd, *J* = 9.5, 3.7 Hz, 1H), 4.04 (dd, *J* = 9.5, 6.8 Hz, 1H), 1.01 (s, 21H).

^{13}C NMR (101 MHz, $CDCl_3$) δ 158.5, 130.1, 118.2, 118.1, 111.8, 111.7, 104.1, 88.0, 71.9, 61.9, 18.5, 11.1.

^{19}F NMR (376 MHz, $CDCl_3$) δ -62.74.

IR (neat) 2980, 2888, 2360, 1461, 1131, 882 cm^{-1}.

HRMS (ESI) m/z: $[M + Na]^+$ Calcd for $C_{20}H_{29}F_3O_2SiNa$ 409.1781; Found 409.1780.

SFC Conditions: The enantioselectivity was determined using Chiralpak IB; 1500 PSI, 30 °C; flow: 1.5 mL/min; from 1% to 10% MeOH in 10 min; 99.47:0.53 er (major enantiomer t_R = 2.49 min; minor enantiomer t_R = 2.65 min), **99% ee**.

$[\alpha]^{25}_D$ = −8.1 (c=0.31, $CHCl_3$)

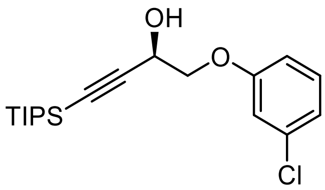

Alcohol **3h** was prepared according to general procedure C from crude ketone **2h** (2.6 g, 7.5 mmol) in 93% yield (2.5 g) and 99% ee as colourless oil. Column conditions: 10% diethylether in hexane.

^{1}H NMR (400 MHz, $CDCl_3$) δ 7.14 (t, *J* = 8.1 Hz, 1H), 6.90 (ddd, *J* = 8.1, 2.2, 0.9 Hz, 1H), 6.87 (t, *J* = 2.2 Hz, 1H), 6.76 (ddd, *J* = 8.1, 2.2, 0.9 Hz, 1H), 4.70 (dd, *J* = 7.0, 3.6 Hz, 1H), 4.05 (dd, *J* = 9.6, 3.6 Hz, 1H), 3.99 (dd, *J* = 9.5, 7.0 Hz, 1H), 1.00 (d, *J* = 3.8 Hz, 21H).

^{13}C NMR (101 MHz, $CDCl_3$) δ 159.0, 134.9, 130.3, 121.6, 115.3, 113.3, 104.1, 87.9, 71.9, 61.9, 18.6, 11.1.

IR (neat) 2980, 2866, 2360, 2341, 1595, 678 cm^{-1}.

HRMS (ESI) m/z: $[M + Na]^+$ Calcd for $C_{19}H_{29}ClO_2SiNa$ 375.1518; Found 375.1516.

SFC Conditions: The enantioselectivity was determined using Chiralpak IB; 1500 PSI, 30 °C; flow: 1.5 mL/min; from 1% to 10% MeOH in 10 min; 99.71:0.29 er (major enantiomer t_R = 1.83 min; minor enantiomer t_R = 2.00 min), **99% ee**.

$[\alpha]^{25}_D$ = −20.0 (c=0.11, $CHCl_3$)

General procedure D: Desilylation of alkyne

To a solution of alcohol (1 equiv) in THF (0.1 M) was added TBAF (1.2 equiv, 1.0 M in THF) at RT. After stirring for 1 h, the reaction was quenched with sat. aq. NH_4Cl and the aqueous layer was extracted with Et_2O. The combined organic layers were dried over Na_2SO_4, filtered and concentrated under vacuum. The resulting alcohol was used without further purification.

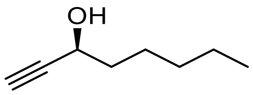

Alkyne **S2a** was prepared according to general procedure D from TIPS protected **3a** (56 mmol) and was used without further purification.

^{1}H NMR (400 MHz, $CDCl_3$) δ 4.37 (td, *J* = 6.6, 2.1 Hz, 1H), 2.44 (d, *J* = 2.0 Hz, 1H), 1.77 – 1.60 (m, 2H), 1.56-1.42 (m, 2H), 1.35 – 1.28 (m, 4H), 0.93 – 0.85 (m, 3H).

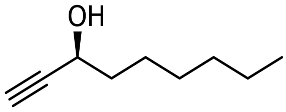

Alkyne **S2b** was prepared according to general procedure D from TIPS protected **3b** (12 mmol) and used without further purification.

^{1}H NMR (400 MHz, $CDCl_3$) δ 4.37 (td, *J* = 6.6, 2.1 Hz, 1H), 2.46 (d, *J* = 2.1 Hz, 1H), 1.76 – 1.64 (m, 2H), 1.52 – 1.38 (m, 2H), 1.36 – 1.25 (m, 6H), 0.91 – 0.85 (m, 3H).

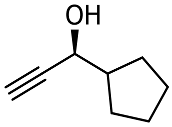

Alkyne **S2c** was prepared according to general procedure D from TIPS protected **3c** (7 mmol) and used without further purification.

^{1}H NMR (400 MHz, $CDCl_3$) δ 4.23 (dd, *J* = 7.1, 2.1 Hz, 1H), 2.42 (d, *J* = 2.1 Hz, 1H), 2.19 (h, *J* = 8.0 Hz, 1H), 1.79 (dddd, *J* = 16.1, 8.0, 6.2, 4.7 Hz, 2H), 1.72 – 1.46 (m, 4H), 1.51 – 1.35 (m, 2H).

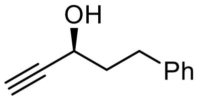

Alkyne **S2d** was prepared according to general procedure D from TIPS protected **3d** (10.6 mmol) and was used without further purification.

^{1}H NMR (400 MHz, $CDCl_3$) δ 7.30-7.26 (m, 2H), 7.25 – 7.17 (m, 3H), 4.37 (td, *J* = 6.6, 2.1 Hz, 1H), 2.81 (t, *J* = 7.8 Hz, 2H), 2.50 (s, 1H), 2.04 (ddt, *J* = 13.5, 10.8, 6.3 Hz, 2H).

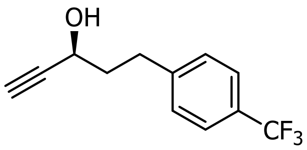

Alkyne **S2e** was prepared according to general procedure D from TIPS protected **3e** (6 mmol) and used without further purification.

^{1}H NMR (400 MHz, $CDCl_3$) δ 7.54 (d, *J* = 8.0 Hz, 2H), 7.33 (d, *J* = 8.0 Hz, 2H), 4.37 (t, *J* = 5.7 Hz, 1H), 2.87 (t, *J* = 7.9 Hz, 2H), 2.53 (d, *J* = 2.1 Hz, 1H), 2.12 – 1.92 (m, 2H).

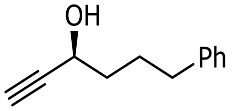

Alkyne **S2f** was prepared according to general procedure D from TIPS protected **3f** (9 mmol) and used without further purification.

^{1}H NMR (400 MHz, $CDCl_3$) δ 7.33 – 7.24 (m, 2H), 7.23 – 7.14 (m, 3H), 4.39 (td, *J* = 6.1, 1.9 Hz, 1H), 2.67 (t, *J* = 7.2 Hz, 2H), 2.46 (d, *J* = 2.1 Hz, 1H), 1.92 – 1.69 (m, 4H).

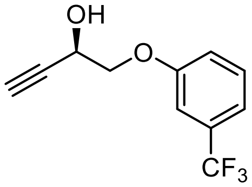

Alkyne **S2g** was prepared according to general procedure D from TIPS protected **3g** (10 mmol) and was used without further purification.

^{1}H NMR (400 MHz, $CDCl_3$) δ 7.33 (t, *J* = 8.0 Hz, 1H), 7.18 – 7.14 (m, 1H), 7.11 (t, *J* = 2.3z Hz, 1H), 7.06 (dd, *J* = 8.0, 2.3 Hz, 1H), 4.75 (ddd, *J* = 6.0, 4.8, 2.1 Hz, 1H), 4.26 – 3.97 (m, 2H), 2.44 (d, *J* = 2.1 Hz, 1H).

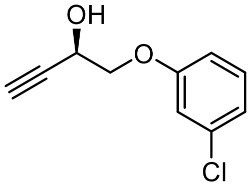

Alkyne **S2h** was prepared according to general procedure D from TIPS protected **3h** (7.6 mmol) and was used without further purification.

^{1}H NMR (400 MHz, $CDCl_3$) δ 7.21 (t, *J* = 8.1 Hz, 1H), 6.97 (ddd, *J* = 8.1, 2.2, 0.9 Hz, 1H), 6.94 (t, *J* = 2.2 Hz, 1H), 6.83 (ddd, *J* = 8.1, 2.2, 0.9 Hz, 1H), 4.88 – 4.71 (m, 1H), 4.13 (dd, *J* = 9.6, 3.8 Hz, 1H), 4.07 (dd, *J* = 9.6, 6.8 Hz, 1H), 2.53 (d, *J* = 2.2 Hz, 1H).

General procedure E: TBS protection of alcohol

Alcohol (1 equiv) was dissolved in DCM (1 M) along with DMAP (5 mol%) and imidazole (1.5 equiv) at 0 ˚C. TBS chloride was added and the resulting suspension was warmed to RT and stirred for one hour. The mixture was diluted with DCM and washed with sat. aq. NH_4Cl. The organic layer was separated and the aqueous layer was extracted with DCM. The combined organic layers were dried over Na_2SO_4 and concentrated under vacuum. Purification by column chromatography yields the desired product.

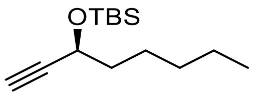

TBS protected alcohol **S3a** was prepared according to general procedure E from free alcohol **S2a** (56 mmol) in 97% yield (13.1 g) over two steps as colourless oil. Column conditions: 1% Et_3N in hexane.

^{1}H NMR (400 MHz, $CDCl_3$) δ 4.33 (td, *J* = 6.5, 2.1 Hz, 1H), 2.37 (d, *J* = 2.1 Hz, 1H), 1.72 – 1.61 (m, 2H), 1.53 – 1.36 (m, 2H), 1.30 (dt, *J* = 6.7, 3.3 Hz, 4H), 0.90 (s, 9H), 0.86 (m, 3H), 0.13 (s, 3H), 0.11 (s, 3H).

^{13}C NMR (101 MHz, $CDCl_3$) δ 85.8, 71.8, 62.8, 38.6, 31.4, 25.9, 24.8, 22.6, 18.2, 17.7, 17.2, 14.0, 12.3, −4.5, −5.1.

IR (neat) 2957, 2862, 2360, 1472, 1091, 837, 777 cm^{-1}.

HRMS (ESI) m/z: $[M + H]^+$ Calcd for $C_{14}H_{29}OSi$ 241.1982; Found 241.1983.

$[\alpha]^{25}_D$ = −9.2 (c=0.81, $CHCl_3$)

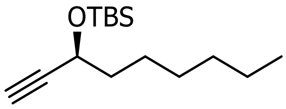

TBS protected alcohol **S3b** was prepared according to general procedure E from free alcohol **S2b** (12 mmol) in 98% yield over 2 steps (3.0 g) as colourless oil. Column conditions: Pure hexane.

^{1}H NMR (500 MHz, $CDCl_3$) δ 4.36 (td, *J* = 6.5, 1.7 Hz, 1H), 2.38 (d, *J* = 1.6 Hz, 1H), 1.75 – 1.62 (m, 2H), 1.52 – 1.38 (m, 2H), 1.30 (d, *J* = 12.1 Hz, 6H), 1.12 (t, *J* = 7.3 Hz, 3H), 0.93 (s, 9H), 0.15 (d, *J* = 12.4 Hz, 6H).

^{13}C NMR (126 MHz, $CDCl_3$) δ 85.9, 72.0, 62.9, 38.8, 32.0, 29.1, 25.9, 25.3, 22.8, 18.3, 14.2, −4.4, −4.9.

IR (neat) 3314, 2931, 2860, 1465, 1256, 1095, 1050, 1007, 884, 838, 778 cm^{-1}.

HRMS (ESI) m/z: $[M + H]^+$ Calcd for $C_{15}H_{31}OSi$ 255.2139; Found 255.2155.

$[\alpha]^{25}_D$ = −34.9 (c=1.0, $CHCl_3$)

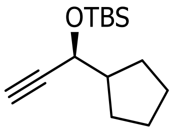

TBS protected alcohol **S3c** was prepared according to general procedure E from free alcohol **S2c** (7 mmol) in 69% yield over 2 steps (1.15 g) as colourless oil. Column conditions: Pure hexane.

^{1}H NMR (500 MHz, $CDCl_3$) δ 4.18 (dd, *J* = 6.8, 2.0 Hz, 1H), 2.33 (d, *J* = 2.0 Hz, 1H), 2.16 (h, *J* = 8.0 Hz, 1H), 1.82 – 1.67 (m, 2H), 1.67 – 1.48 (m, 4H), 1.42 (dp, *J* = 11.9, 4.0 Hz, 2H), 0.90 (s, 9H), 0.27 – -0.01 (d, *J* = 18.1 Hz, 6H).

^{13}C NMR (126 MHz, $CDCl_3$) δ 85.6, 72.0, 66.5, 47.1, 28.9, 28.6, 25.9, 25.9, 25.8, 18.4, −4.3, −5.0.

IR (neat) 3313, 2959, 2861, 1473, 1257, 1097, 840, 779 cm^{-1}.

HRMS (ESI) m/z: $[M + H]^+$ Calcd for $C_{14}H_{27}OSi$ 239.1826; Found 239.1830.

$[\alpha]^{25}_{D}$ = −45.4 (c=1.0, $CHCl_3$)

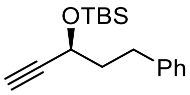

TBS protected alcohol **S3d** was prepared according to general procedure E from free alcohol **S2d** (10.6 mmol) in 95% yield (2.8 g) over two steps as colourless oil. Column conditions: 1% Et_3N in hexane.

^{1}H NMR (400 MHz, $CDCl_3$) δ 7.38 – 7.21 (m, 2H), 7.20-7.16 (m, 3H), 4.35 (td, *J* = 6.3, 2.1 Hz, 1H), 2.85 – 2.67 (m, 2H), 2.41 (dd, *J* = 6.8, 2.1 Hz, 1H), 2.06 – 1.92 (m, 2H), 0.90 (s, 9H), 0.12 (s, 3H), 0.09 (s, 3H).

^{13}C NMR (101 MHz, $CDCl_3$) δ 141.6, 128.5, 128.4, 125.9, 85.3, 72.4, 62.1, 40.2, 31.3, 25.8, 18.4, −4.6, −5.0.

IR (neat) 2980, 2888, 2360, 1472, 1384, 1091, 836 cm^{-1}.

HRMS (ESI) m/z: $[M + H]^+$ Calcd for $C_{17}H_{26}OSi$ 275.1826; Found 275.1825.

$[\alpha]^{25}_{D}$ = −11.4 (c=0.64, $CHCl_3$)

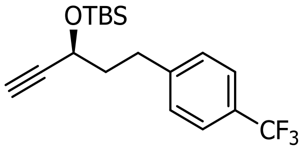

TBS protected alcohol **S3e** was prepared according to general procedure E from free alcohol **S2e** (6 mmol) in 86% yield over 2 steps (1.77 g) as colourless oil. Column conditions: Pure hexane.

^{1}H NMR (500 MHz, $CDCl_3$) δ 7.54 (d, *J* = 8.0 Hz, 2H), 7.31 (d, *J* = 8.0 Hz, 2H), 4.39 (td, *J* = 6.2, 1.9 Hz, 1H), 2.84 (q, *J* = 7.2 Hz, 2H), 2.44 (d, *J* = 2.0 Hz, 1H), 2.06 – 1.95 (m, 2H), 0.92 (s, 9H), 0.13 (d, *J* = 16.7 Hz, 6H).

^{13}C NMR (126 MHz, $CDCl_3$) δ 146.0, 128.9, 128.5 (q, *J* = 32.3 Hz), 125.4 (q, *J* = 3.8 Hz), 123.4, 85.1, 72.8, 62.1, 40.0, 31.3, 25.9, 18.3, −4.4, −4.9.

^{19}F NMR (376 MHz, $CDCl_3$) δ −62.3.

IR (neat) 3312, 2956, 2932, 2860, 1620, 1327, 1256, 1166, 1128, 1098, 1068, 839, 779 cm^{-1}.

HRMS (ESI) m/z: $[M + H]^+$ Calcd for $C_{18}H_{26}F_3OSi$ 343.1700; Found 343.1709.

$[\alpha]^{25}_D$ = −14.5 (c=1.0, $CHCl_3$)

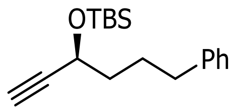

TBS protected alcohol **S3f** was prepared according to general procedure E from free alcohol **S2f** (9 mmol) in 89% yield over 2 steps (2.3 g) as colourless oil. Column conditions: Pure hexane.

^{1}H NMR (500 MHz, $CDCl_3$) δ 7.34 (t, *J* = 7.5 Hz, 1H), 7.24 (d, *J* = 7.3 Hz, 2H), 4.43 (td, *J* = 6.1, 1.9 Hz, 1H), 2.71 (t, *J* = 7.3 Hz, 1H), 2.42 (d, *J* = 2.0 Hz, 1H), 1.93 – 1.72 (m, 4H), 0.97 (s, 9H), 0.18 (d, *J* = 13.0 Hz, 6H).

^{13}C NMR (126 MHz, $CDCl_3$) δ 142.4, 128.5, 128.4, 125.9, 85.7, 72.2, 62.7, 38.2, 35.6, 26.9, 25.9, 18.3, −4.4, −4.9.

IR (neat) 3310, 3028, 2951, 2930, 2858, 1497, 1463, 1254, 1098, 1006, 839, 779, 747, 699 cm^{-1}.

HRMS (ESI) m/z: $[M + Na]^+$ Calcd for $C_{18}H_{28}OSiNa$ 311.1802; Found 311.1812.

$[\alpha]^{25}_D$ = −29.7 (c=1.0, $CHCl_3$)

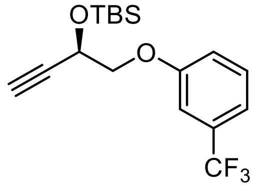

TBS protected alcohol **S3g** was prepared according to general procedure E from free alcohol **S2g** (7.2 mmol) in 83% yield (2.0 g) over two steps as colourless oil. Column conditions: 1% Et_3N in hexane.

^{1}H NMR (400 MHz, $CDCl_3$) δ 7.31 (t, *J* = 8.0 Hz, 1H), 7.14 (dq, *J* = 7.7, 0.9 Hz, 1H), 7.07 (d, *J* = 2.2 Hz, 1H), 7.01 (dd, *J* = 8.3, 2.6 Hz, 1H), 4.71 – 4.63 (m, 1H), 4.06-4.00 (m, 1H), 2.40 (dd, *J* = 2.1, 0.6 Hz, 1H), 0.84 (d, *J* = 0.6 Hz, 9H), 0.10 (s, 3H), 0.06 (s, 3H).

^{13}C NMR (101 MHz, $CDCl_3$) δ 159.0, 132.2 (q, *J* = 32.3 Hz),130.3, 118.5, 118.0 (q, *J* = 4.0 Hz), 111.8 (q, *J* = 3.9 Hz), 82.3, 74.0, 72.5, 62.3, 25.9, 18.3, −4.5, −4.7.

^{19}F NMR (376 MHz, $CDCl_3$) δ −62.71.

IR (neat) 2980, 2360, 1593, 1329, 1128, 837, 781 cm^{-1}.

HRMS (ESI) m/z: $[M + Na]^+$ Calcd for $C_{17}H_{23}F_3O_2SiNa$ 367.1312; Found 367.1310.

$[\alpha]^{25}_D$ = −33.1 (c=0.8, $CHCl_3$)

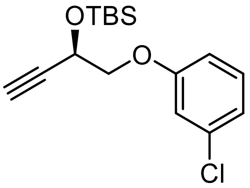

TBS protected alcohol **S3h** was prepared according to general procedure E from free alcohol **S2h** (5.6 mmol) in 86% yield (1.2 g) over two steps as colourless oil. Column conditions: 1% Et_3N in hexane.

^{1}H NMR (400 MHz, $CDCl_3$) δ 7.19 (dd, *J* = 8.4, 7.8 Hz, 1H), 6.98 – 6.89 (m, 2H), 6.80 (ddd, *J* = 8.4, 2.4, 0.9 Hz, 1H), 4.72 (ddd, *J* = 6.6, 5.2, 2.1 Hz, 1H), 4.11 – 3.99 (m, 2H), 2.47 (d, *J* = 2.2 Hz, 1H), 0.91 (s, 9H), 0.17 (s, 3H), 0.13 (s, 3H).

^{13}C NMR (101 MHz, $CDCl_3$) δ 159.3, 134.9, 130.2, 121.3, 115.2, 113.2, 82.1, 73.6, 72.2, 62.1, 25.7, 18.3, −4.8, −5.0.

IR (neat) 2980, 2887, 2360, 2341, 1596, 1122, 837 cm^{-1}.

HRMS (ESI) m/z: $[M + H]^+$ Calcd for $C_{16}H_{23}ClO_2Si$ 311.1229; Found 311.1230.

$[\alpha]^{25}_D$ = −4.3 (c=0.37, $CHCl_3$)

General procedure F: Borylation of alkyne

To a flame tried high pressure flask was added 4-methylaminobenzoic acid (5 mol%) and pinacolborane (3 equiv) under an inert atmosphere. A solution of alkyne (1 equiv) in dry heptane (1 M) was added, and the flask was sealed then heated to 110 °C using an oil bath for 16h. The flask was cooled, and the mixture was diluted with EtOAc and washed with NH_4Cl. The aqueous layer was separated and extracted with EtOAc, and the combined organic layers were dried over Na_2SO_4, filtered and concentrated under vacuum. The resulting oil was purified by column chromatography to yield the boronic ester.

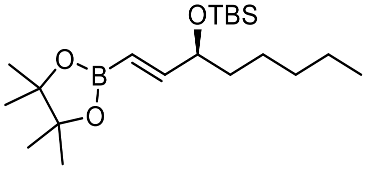

Alkenyl boronic ester **4a** was prepared according to general procedure E from alkyne **S3a** (15.8 mmol) in 88% yield (5.2 g) as colourless oil. Column conditions: 1% Et_3N in Hexane.

^{1}H NMR (400 MHz, $CDCl_3$) δ 6.55 (dd, *J* = 18.0, 4.7 Hz, 1H), 5.56 (dd, *J* = 18.0, 1.6 Hz, 1H), 4.13 (tdd, *J* = 6.1, 4.7, 1.6 Hz, 1H), 1.53 – 1.39 (m, 4H), 1.25 (s, 18H), 0.88 (s, 9H), 0.01 (d, *J* = 6.5 Hz, 6H).

^{13}C NMR (101 MHz, $CDCl_3$) δ 156.3, 83.1, 74.2, 37.5, 31.9, 25.9, 24.8, 24.8, 18.3, 14.1, −4.39, −4.86. (The C–B signal was not observed due to quadrupolar relaxation.)

IR (neat) 2958, 2931, 2360, 1643, 1356, 1146, 836 cm^{-1}

HRMS (ESI) m/z: $[M + Na]^+$ Calcd for $C_{20}H_{41}BO_3SiNa$ 391.2810; Found 391.2805.

$[\alpha]^{25}_D$ = −5.0 (c=0.26 $CHCl_3$)

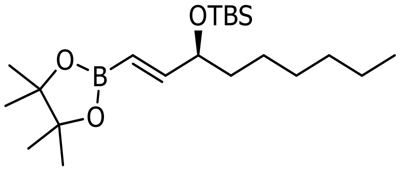

Alkenyl boronic ester **4b** was prepared according to general procedure E from alkyne **S3b** (5 mmol) in 43% yield (830 mg) as colourless oil. Column conditions: Pure hexane.

^{1}H NMR (400 MHz, $CDCl_3$) δ 6.55 (dd, *J* = 18.0, 4.9 Hz, 1H), 5.56 (dd, *J* = 18.0, 1.5 Hz, 1H), 4.14 (qd, *J* = 6.1, 1.4 Hz, 1H), 1.52 – 1.42 (m, 2H), 1.33 – 1.17 (m, 20H(12H+8H)), 0.87 (d, *J* = 8.7 Hz, 12H(9H+3H)), 0.01 (d, *J* = 6.6 Hz, 6H).

^{13}C NMR (101 MHz, $CDCl_3$) δ 156.4, 83.2, 74.3, 37.7, 32.0, 29.5, 26.1, 25.1, 24.9, 24.9, 22.8, 18.4, 18.3, 14.2, 12.6, -4.2, -4.7.

IR (neat) 2980, 2959, 2934, 2860, 2360, 2342, 1370, 1343, 1148, 1091, 1002, 972, 838 cm^{-1}

HRMS (ESI) m/z: $[M + Na]^+$ Calcd for $C_{21}H_{43}BO_3SiNa$ 405.2967; Found 405.2977.

$[\alpha]^{25}_D$ = −1.9 (c=1.0, $CHCl_3$)

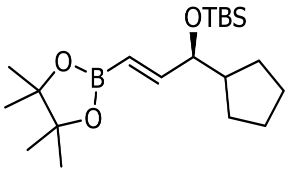

Alkenyl boronic ester **4c** was prepared according to general procedure E from alkyne **S3c** (5 mmol) in 57% yield (1.04 g) as colourless oil. Column conditions: Pure hexane.

^{1}H NMR (400 MHz, $CDCl_3$) δ 6.62 – 6.50 (m, 1H), 5.58 – 5.48 (m, 1H), 4.00 – 3.92 (m, 1H), 1.90 (dt, *J* = 15.8, 7.9 Hz, 1H), 1.66 – 1.32 (m, 8H), 1.25 (d, *J* = 1.7 Hz, 12H), 0.88 (s, 9H), -0.00 (d, *J* = 14.3 Hz, 6H).

^{13}C NMR (101 MHz, $CDCl_3$) δ 155.9, 83.2, 78.1, 46.3, 28.8, 28.5, 26.1, 25.8, 25.6, 25.0, 24.8, 18.4, -3.9, -4.7.

IR (neat) 2956, 2860, 1642, 1362, 1330, 1256, 1216, 1147, 1106, 1004, 972, 837, 757 cm^{-1}

HRMS (ESI) m/z: $[M + Na]^+$ Calcd for $C_{20}H_{39}BO_3SiNa$ 389.2654; Found 389.2689.

$[\alpha]^{25}_{D}$ = −17.8 (c=1.0, $CHCl_3$)

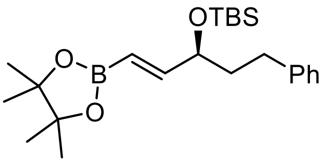

Alkenyl boronic ester **4d** was prepared according to general procedure E from alkyne **S3d** (3.5 mmol) in 81% yield (1.13 g) as colourless oil. Column conditions: 1% Et_3N in Hexane.

^{1}H NMR (400 MHz, $CDCl_3$) δ 7.32 – 7.24 (m, 2H), 7.17 (d, *J* = 7.4 Hz, 3H), 6.62 (dt, *J* = 18.0, 4.7 Hz, 1H), 5.64 (dd, *J* = 18.0, 1.6 Hz, 1H), 4.25 (qd, *J* = 5.8, 1.6 Hz, 1H), 2.71 – 2.56 (m, 2H), 1.94 – 1.76 (m, 2H), 1.28 (s, 12H), 0.92 (s, 9H), 0.05 (s, 3H), 0.03 (s, 3H).

^{13}C NMR (101 MHz, $CDCl_3$) δ 155.6, 142.5, 128.4, 128.3, 125.7, 83.2, 73.6, 39.1, 31.1, 26.0, 24.81, 24.76, 18.2, −4.4, −4.9.

IR (neat) 2980, 2360, 1645, 1328, 1130 cm^{-1}.

HRMS (ESI) m/z: $[M + Na]^+$ Calcd for $C_{23}H_{39}BO_3SiNa$ 425.2654; Found 425.2652.

$[\alpha]^{25}_{D}$ = +14.3 (c=0.6, $CHCl_3$)

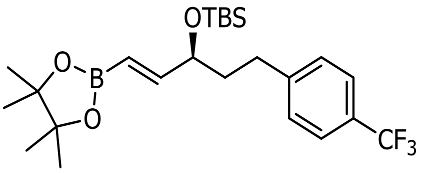

Alkenyl boronic ester **4e** was prepared according to general procedure E from alkyne **S3e** (5 mmol) in 54% yield (1.28 g) as colourless oil. Column conditions: Pure hexane.

^{1}H NMR (400 MHz, $CDCl_3$) δ 7.51 (d, *J* = 8.1 Hz, 2H), 7.27 (d, *J* = 8.1 Hz, 2H), 6.59 (dd, *J* = 18.0, 4.9 Hz, 1H), 5.65 (dd, *J* = 18.0, 1.4 Hz, 1H), 4.26 (qd, *J* = 5.6, 1.4 Hz, 1H), 2.77 – 2.64 (m, 2H), 1.90 – 1.77 (m, 2H), 1.28 (s, 12H), 0.92 (s, 9H), 0.04 (d, *J* = 5.6 Hz, 6H).

^{13}C NMR (101 MHz, $CDCl_3$) δ 155.3, 146.8, 128.8, 128.23 (ad, *J* = 32.3 Hz), 125.34 (q, *J* = 3.6 Hz), 83.3, 73.6, 38.9, 31.0, 26.1, 24.92, 24.87, 18.4, -4.2, -4.8.

^{19}F NMR (376 MHz, $CDCl_3$) δ -62.3.

IR (neat) 2980, 2957, 2932, 2859, 2360, 2343, 1642, 1370, 1327, 1255, 1217, 1165, 1146, 1127, 1069, 1020, 1001, 971 cm^{-1}

HRMS (ESI) m/z: $[M + Na]^+$ Calcd for $C_{24}H_{38}BF_3O_3SiNa$ 493.2528; Found 493.2528.

$[\alpha]^{25}_D$ = +9.5 (c=1.0, $CHCl_3$)

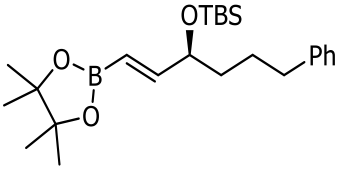

Alkenyl boronic ester **4f** was prepared according to general procedure E from alkyne **S3f** (5 mmol) in 53% yield (1.1 g) as colourless oil. Column conditions: Pure hexane.

^{1}H NMR (400 MHz, $CDCl_3$) δ 7.31 – 7.22 (m, 2H), 7.21 – 7.13 (m, 3H), 6.55 (dd, *J* = 18.0, 4.9 Hz, 1H), 5.58 (dd, *J* = 18.0, 1.5 Hz, 1H), 4.18 (qd, *J* = 6.0, 1.5 Hz, 1H), 2.60 (t, *J* = 7.6 Hz, 2H), 1.76 – 1.59 (m, 2H), 1.58 – 1.48 (m, 2H), 1.27 (s, 12H), 0.89 (s, 9H), 0.02 (d, *J* = 4.9 Hz, 6H).

^{13}C NMR (101 MHz, $CDCl_3$) δ 156.1, 142.6, 128.6, 128.4, 125.8, 83.2, 74.1, 37.1, 36.0, 26.7, 26.1, 24.94, 24.89, 18.4, -4.2, -4.7.

IR (neat) 2979, 2932, 2858, 2360, 1642, 1463, 1370, 1341, 1256, 1217, 1147, 1105, 1003, 971 cm^{-1}

HRMS (ESI) m/z: $[M + Na]^+$ Calcd for $C_{24}H_{41}BO_3SiNa$ 439.2810; Found 439.2833.

$[\alpha]^{25}_D$ = +3.7 (c=1.0, $CHCl_3$)

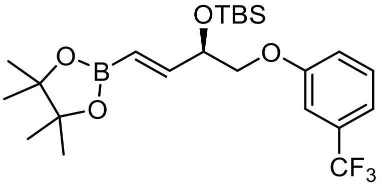

Alkenyl boronic ester **4g** was prepared according to general procedure E from alkyne **S3g** (3.5 mmol) in 88% yield (1.45g) as colourless oil. Column conditions: 1% Et_3N in Hexane.

^{1}H NMR (400 MHz, $CDCl_3$) δ 7.37 (t, *J* = 7.7 Hz, 1H), 7.19 (dd, *J* = 7.7, 2.3, 1H), 7.09 (t, *J* = 2.3 Hz, 1H), 7.04 (dd, *J* = 8.3, 2.3 Hz, 1H), 6.66 (dd, *J* = 18.0, 4.1 Hz, 1H), 5.86 (dd, *J* = 18.0, 1.8 Hz, 1H), 4.60 (dtd, *J* = 7.5, 4.1, 1.8 Hz, 1H), 3.95 (dd, *J* = 9.4, 4.1 Hz, 1H), 3.85 (dd, *J* = 9.4, 7.5 Hz, 1H), 1.28 (s, 12H), 0.91 (s, 9H), 0.09 (s, 3H), 0.08 (s, 3H).

^{13}C NMR (101 MHz, $CDCl_3$) δ 158.9, 151.0, 131.8 (app d, *J* = 32.3 Hz), 129.9, 118.1, 117.4 (app d, *J* = 3.9 Hz), 111.2, 111.1, 83.4, 72.5, 72.1, 25.9, 24.8, 24.8, 18.3, −4.6, −4.7.

^{19}F NMR (376 MHz, $CDCl_3$) δ −62.71.

IR (neat) 2980, 2360, 1645, 1448, 1328, 1130 cm^{-1}

HRMS (ESI) m/z: $[M + H]^+$ Calcd for $C_{23}H_{37}BF_3O_4Si$ 473.2501; Found 473.2498.

$[\alpha]^{25}_{D}$ = −2.2 (c=0.25, $CHCl_3$)

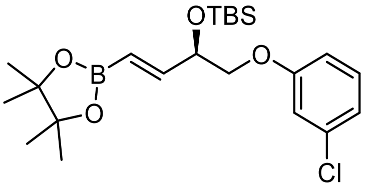

Alkenyl boronic ester **4h** was prepared according to general procedure E from alkyne **S3h** (2.9 mmol) in 78% yield (0.96 g) as colourless oil. Column conditions: 1% Et_3N in Hexane.

^{1}H NMR (400 MHz, $CDCl_3$) δ 7.17 (t, *J* = 8.0 Hz, 1H), 6.91 (ddd, *J* = 8.0, 2.2, 0.9 Hz, 1H), 6.87 (d, *J* = 2.2 Hz, 1H), 6.76 (ddd, *J* = 8.0, 2.2, 0.9 Hz, 1H), 6.65 (dd, *J* = 18.0, 4.0 Hz, 1H), 5.85 (dd, *J* = 18.0, 1.8 Hz, 1H), 4.58 (dtd, *J* = 7.6, 4.0, 1.8 Hz, 1H), 3.85 (s, 1H), 3.80 (dd, *J* = 9.5, 7.5 Hz, 1H), 1.28 (s, 12H), 0.91 (s, 9H), 0.09 (s, 3H), 0.08 (s, 3H).

^{13}C NMR (101 MHz, $CDCl_3$) δ 159.5, 151.1, 134.8, 130.2, 120.9, 114.9, 113.0, 83.4, 72.5, 72.1, 25.9, 24.83, 24.76, 18.3, −4.66, −4.71.

IR (neat) 2985, 2931, 2360, 1643, 1365, 1146, 836 cm^{-1}

HRMS (ESI) m/z: $[M + Na]^+$ Calcd for $C_{22}H_{36}BClO_4SiNa$ 461.2057; Found 461.2053.

$[\alpha]^{25}_{D}$ = −4.3 (c=0.37, $CHCl_3$)

General procedure G: Coupling of allyl chloride and Alkenyl boronic ester.

Flask A. $[Rh(cod)OH]_2$ (2.5 mol%) and (*S*)-DM-Segphos (6 mol%) were weighed and added to a 10 mL flame dried flask equipped with a magnetic stir bar. The flask was put under reduced pressure for 5 minutes, then back-filled with argon. This was repeated once more, before THF (1 mL) and CsOH (50 wt%, 1 equiv) was added and the flask was stirred at 65 °C using an oil bath for 30 min.

Flask B. Meanwhile, allyl chloride (*rac*)-**5** (1 equiv.) and Alkenyl boronic ester (1.2 equiv) were added to a flame dried 5 mL RBF and dissolved in dry THF (0.7 mL).

After flask A stirred for 30 min, the contents of flask B were added via syringe, and flask B was washed with 0.3 mL THF which was also added to flask A. Reaction progress was monitored by ^1H NMR spectroscopy. Upon completion (1-3 h), silica gel was added directly to the reaction mixture which was concentrated and dry loaded for purification by silica gel chromatography.

Note: For compound 6, no change was made to the experimental procedure when increasing scale to 5 mmol of allyl chloride other than the size of the flasks used – all other factors (time, temperature etc.) were consistent with the procedure used for reactions carried out on a 0.5 mmol scale. Flask A size used is 25 mL, and flask B size is 50 mL..

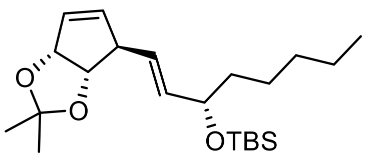

Coupled product **6** was prepared according to general procedure G from allyl chloride (*rac*)-**5** (5 mmol) in 90% yield (1.54 g) in 99% de as colourless oil. Column conditions: 0-10% EtOAc in hexane.

Note: when carried out on a 0.5 mmol scale, the isolated yield of this reaction was 88% (151 mg), and when carried out on 3.2 mmol scale, the isolated yield was 90% (1.10 g). In all cases, the de observed was 99%.

^{1}H NMR (400 MHz, $CDCl_3$) δ 5.82 (dt, *J* = 5.8, 1.8 Hz, 1H), 5.79 – 5.74 (m, 1H), 5.55 – 5.39 (m, 2H), 5.19 – 5.12 (m, 1H), 4.40 (d, *J* = 5.7 Hz, 1H), 4.02 (dt, *J* = 7.0, 5.4 Hz, 1H), 3.45 – 3.38 (m, 1H), 1.42 (s, 3H), 1.34 (s, 3H), 1.31 (s, 8H), 0.90 – 0.85 (m, 12H), 0.03 (s, 3H), 0.00 (s, 3H).

^{13}C NMR (101 MHz, $CDCl_3$) δ 135.5, 135.1, 131.2, 129.0, 110.1, 85.1, 83.8, 73.1, 53.9, 38.3, 31.8, 27.5, 25.9, 25.0, 22.6, 18.3, 14.0, -4.2, -4.8.

IR 2957, 2931, 2360, 2341, 1472, 1377, 1251, 1212, 1066, 810, 774 cm^{-1}.

$[\alpha]^{25}_D$ −141.1 (c=0.2, CHCl3)

HRMS (ESI) m/z: $[M + H]^+$ Calcd for $C_{22}H_{41}O_3Si$ 381.2820; Found 381.2812.

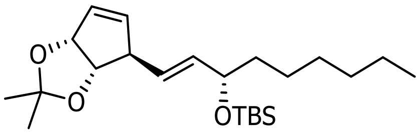

Coupled product **7** was prepared according to general procedure G from allyl chloride (*rac*)-**5** (0.5 mmol) in 87% yield (172 mg) in 99% de as colourless oil. Column conditions: 2-10% diethylether in hexane

^{1}H NMR (500 MHz, $CDCl_3$) δ 5.82 (dt, *J* = 5.7, 1.6 Hz, 1H), 5.75 (dd, *J* = 5.7, 2.3 Hz, 1H), 5.54 – 5.40 (m, 2H), 5.16 (d, *J* = 5.3 Hz, 1H), 4.41 (d, *J* = 5.7 Hz, 1H), 4.02 (q, *J* = 5.7 Hz, 1H), 3.42 (d, *J* = 6.1 Hz, 1H), 1.42 (s, 3H), 1.34 (s, 3H), 1.32 – 1.18 (m, 10H), 0.90 – 0.86 (m, 12H (9H +3H)), 0.02 (d, *J* = 11.6 Hz, 6H).

^{13}C NMR (126 MHz, $CDCl_3$) δ 135.6, 135.3, 131.3, 129.1, 110.3, 85.2, 83.9, 73.3, 54.0, 38.5, 32.0, 29.4, 27.6, 26.1, 26.0, 25.4, 22.8, 18.4, 14.2, -4.1, -4.6.

IR (neat) 2957, 2931, 2858, 1463, 1369, 1253, 1216, 1160, 1069, 972, 868, 836, 775 cm^{-1}.

HRMS (ESI) m/z: $[M + Na]^+$ Calcd for $C_{23}H_{42}O_3SiNa$ 417.2795; Found 417.2797.

$[\alpha]^{25}_D$ = −120.3 (c=1.0, $CHCl_3$)

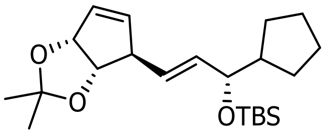

Coupled product **8** was prepared according to general procedure G from allyl chloride (*rac*)-**5** (0.5 mmol) in 91% yield (173 mg) in 99% de as colourless oil. Column conditions: 2-10% diethylether in hexane.

^{1}H NMR (500 MHz, $CDCl_3$) δ 5.82 (dt, *J* = 5.6, 1.7 Hz, 1H), 5.74 (dd, *J* = 5.6, 2.5 Hz, 1H), 5.52 – 5.40 (m, 2H), 5.18 – 5.13 (m, 1H), 4.40 (d, *J* = 5.7 Hz, 1H), 3.87 – 3.80 (m, 1H), 3.45 – 3.39 (m, 1H), 1.86 (h, *J* = 8.0 Hz, 1H), 1.73 – 1.63 (m, 1H), 1.58 – 1.44 (m, 6H), 1.42 (s, 3H), 1.34 (s, 3H), 1.21 – 1.11 (m, 1H), 0.87 (s, 9H), 0.05 – -0.03 (m, 6H).

^{13}C NMR (126 MHz, $CDCl_3$) δ 135.6, 134.8, 131.3, 129.6, 110.3, 85. 2, 83.9, 77.2, 54.0, 47.0, 29.0, 28.9, 27.6, 26.05, 26.00, 25.7, 25.6, 18.4, -3.7, -4.6.

IR (neat) 2956, 2859, 2360, 1463, 1369, 1253, 1215, 1159, 1054, 973, 868, 837, 775 cm^{-1}.

HRMS (ESI) m/z: $[M + Na]^+$ Calcd for $C_{22}H_{38}O_3SiNa$ 401.2482; Found 401.2517.

$[\alpha]^{25}_D$ = −126.9 (c=1.0, $CHCl_3$)

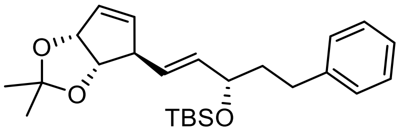

Coupled product **9** was prepared according to general procedure G from allyl chloride (*rac*)-**5** (0.5 mmol) in 93% yield (192 mg) in 99% de as colourless oil. Column conditions: 0-10% EtOAc in hexane.

^{1}H NMR (400 MHz, $CDCl_3$) δ 7.30-7.24 (m, 2H), 7.21-7.14 (m, 3H), 5.84 (dt, *J* = 5.7, 1.8 Hz, 1H), 5.79 – 5.74 (m, 1H), 5.58 – 5.43 (m, 2H), 5.16 (dq, *J* = 5.7, 1.3 Hz, 1H), 4.41 (d, *J* = 5.7 Hz, 1H), 4.11 (dt, *J* = 6.5, 5.3 Hz, 1H), 3.46 – 3.38 (m, 1H), 2.74 – 2.52 (m, 2H), 1.91 – 1.68 (m, 2H), 1.43 (s, j3H), 1.35 (s, 3H), 0.90 (s, 9H), 0.05 (s, 3H), 0.02 (s, 3H).

^{13}C NMR (101 MHz, $CDCl_3$) δ 142.4, 135.4, 134.7, 131.3, 129.6, 128.4, 128.3, 125.7, 110.2, 85.0, 83.8, 72.6, 53.9, 40.0, 31.6, 27.5, 26.0, 18.3, -4.1, -4.7.

IR (neat) 2980, 2888, 2360, 2341, 1461, 1381, 1156, 1071 cm^{-1}

HRMS (ESI) m/z: $[M + Na]^+$ Calcd for $C_{25}H_{38}O_3SiNa$ 437.2482; Found 437.2482.

$[\alpha]^{25}_{D}$ = -104.2 (c=0.2, $CHCl_3$)

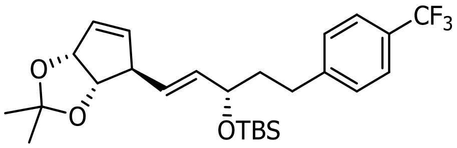

Coupled product **10** was prepared according to general procedure G from allyl chloride (*rac*)-**5** (0.5 mmol) in 79% yield (190 mg) in 99% de as colourless oil. Column conditions: 2-15% diethylether in hexane.

^{1}H NMR (400 MHz, $CDCl_3$) δ 7.52 (d, *J* = 8.0 Hz, 2H), 7.27 (d, *J* = 8.0 Hz, 2H), 5.84 (dt, *J* = 5.7, 1.7 Hz, 1H), 5.79 – 5.70 (m, 1H), 5.61 – 5.43 (m, 2H), 5.20 – 5.13 (m, 1H), 4.41 (d, *J* = 5.7 Hz, 1H), 4.12 (q, *J* = 5.7 Hz, 1H), 3.45 (dd, *J* = 4.8, 2.2 Hz, 1H), 2.80 – 2.58 (m, 2H), 1.88 – 1.68 (m, 2H), 1.42 (s, 3H), 1.35 (s, 3H), 0.90 (s, 9H), 0.05 (s, 6H).

^{13}C NMR (101 MHz, $CDCl_3$) δ 146.7, 135.4, 134.5, 131.5, 130.1, 128.8, 128.2 (ad, *J* = 32.3 Hz), 125.4 (q, *J* = 3.7 Hz), 124.50 (ad, *J* = 271.7 Hz), 110.4, 85.2, 83.9, 72.5, 54.0, 39.8, 31.5, 27.6, 26.0, 25.9, 18.4, -4.0, -4.6.

^{19}F NMR (376 MHz, $CDCl_3$) δ -62.3.

IR (neat) 2981, 2957, 2933, 2859, 2360, 2341, 1327, 1254, 1216, 1164, 1127, 1069, 1020, 972, 837, 776 cm^{-1}.

HRMS (ESI) m/z: $[M + Na]^+$ Calcd for $C_{26}H_{37}F_3O_3SiNa$ 505.2356; Found 505.2377.

$[\alpha]^{25}_{D}$ = −97.7 (c=0.25, $CHCl_3$)

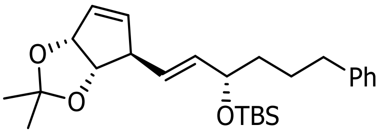

Coupled product **11** was prepared according to general procedure G from allyl chloride (*rac*)-**5** (0.5 mmol) in 82% yield (175 mg) in 99% de as colourless oil. Column conditions: 2-15% diethylether in hexane.

^{1}H NMR (400 MHz, $CDCl_3$) δ 7.31 – 7.20 (m, 2H), 7.20 – 7.10 (m, 3H), 5.81 (dt, *J* = 5.7, 1.7 Hz, 1H), 5.77 – 5.69 (m, 1H), 5.53 – 5.37 (m, 2H), 5.18 – 5.09 (m, 1H), 4.38 (d, *J* = 5.7 Hz, 1H), 4.05 (q, *J* = 5.7 Hz, 1H), 3.44 – 3.33 (m, 1H), 2.58 (t, *J* = 7.5 Hz, 2H), 1.69 – 1.43 (m, 4H), 1.40 (s, 3H), 1.33 (s, 3H), 0.86 (s, 9H), -0.00 (d, *J* = 9.9 Hz, 6H).

^{13}C NMR (101 MHz, $CDCl_3$) δ 142.6, 135.5, 135.0, 131.4, 129.4, 128.5, 128.4, 125.8, 110.3, 85.2, 83. 9, 73.0, 54.0, 37.9, 36.0, 27.6, 27.1, 26.1, 26.05, 25.95, 18.4, -4.0, -4.6.

IR (neat) 2932, 2858, 2360, 1497, 1462, 1370, 1253, 1214, 1159, 1053, 1005, 971 cm^{-1}.

HRMS (ESI) m/z: $[M + Na]^+$ Calcd for $C_{26}H_{40}O_3SiNa$ 451.2639; Found 451.2654.

$[\alpha]^{25}_{D}$ = −96.8 (c=1.0, $CHCl_3$)

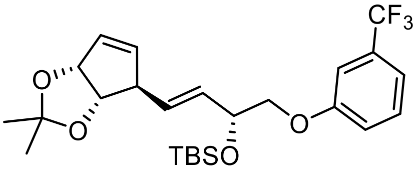

Coupled product **12** was prepared according to general procedure G from allyl chloride (*rac*)-**5** (0.5 mmol) in 85% yield (200 mg) in 99% de as colourless oil. Column conditions: 0-10% EtOAc in hexane.

^{1}H NMR (400 MHz, $CDCl_3$) δ 7.37 (td, *J* = 8.0, 1.0 Hz, 1H), 7.20 (ddt, *J* = 8.0, 2.4, 1.0 Hz, 1H), 7.08 (t, *J* = 2.4 Hz, 1H), 7.04 (dd, *J* = 8.0, 2.4 Hz, 1H), 5.91 – 5.81 (m, 1H), 5.81 – 5.71 (m, 2H), 5.56 (ddd, *J* = 15.5, 5.4, 1.2 Hz, 1H), 4.53 – 4.45 (m, 1H), 4.45 – 4.39 (m, 1H), 3.92 – 3.81 (m, 2H), 3.50 – 3.42 (m, 1H), 1.45 – 1.40 (m, 3H), 1.35 (d, *J* = 0.7 Hz, 3H), 0.95 – 0.86 (m, 9H), 0.14 – 0.05 (m, 6H).

^{13}C NMR (101 MHz, $CDCl_3$) δ 158.9, 135.1, 132.0, 131.6, 130.5, 123.0, 118.2, 117.4, 111.2, 110.3, 85.0, 83.7, 72.5, 71.4, 54.0, 27.5, 25.9, 18.3, -4.6, -4.7.

^{19}F NMR (376 MHz, $CDCl_3$) δ -62.69.

IR (neat) 2980, 2888, 2360, 2341, 1458, 1381, 1130, 955 cm^{-1}.

HRMS (ESI) m/z: $[M + Na]^+$ Calcd for $C_{25}H_{35}F_3O_4SiNa$ 507.2149; Found 507.2148.

$[\alpha]^{25}_{D}$ = −109.1 (c=0.23, $CHCl_3$)

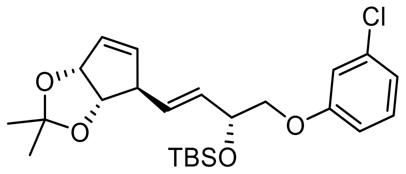

Coupled product **13** was prepared according to general procedure G from allyl chloride (*rac*)-**5** (0.5 mmol) in 94% yield (206 mg) in 99% de as colourless oil. Column conditions: 0-10% EtOAc in hexane.

^{1}H NMR (400 MHz, $CDCl_3$) δ 7.17 (t, *J* = 8.1 Hz, 1H), 6.91 (ddd, *J* = 8.1, 2.0, 0.9 Hz, 1H), 6.86 (t, *J* = 2.5 Hz, 1H), 6.75 (ddd, *J* = 8.1, 2.5, 0.9 Hz, 1H), 5.86 (dt, *J* = 5.7, 1.8 Hz, 1H), 5.81 – 5.69 (m, 2H), 5.59 – 5.49 (m, 1H), 5.16 (dq, *J* = 5.9, 1.4 Hz, 1H), 4.46 (q, *J* = 5.8 Hz, 1H), 4.41 (d, *J* = 5.7 Hz, 1H), 3.81 (d, *J* = 5.9 Hz, 2H), 3.51 – 3.43 (m, 1H), 1.42 (s, 3H), 1.35 (s, 3H), 0.89 (s, 9H), 0.07 (s, 6H).

^{13}C NMR (101 MHz, $CDCl_3$) δ 159.6, 135.1, 134.8, 131.8, 131.6, 130.5, 130.2, 120.9, 114.9, 113.0, 110.3, 85.0, 83.7, 72.5, 71.4, 54.0, 27.5, 25.8, -4.6, -4.7.

IR (neat) 2980, 2888, 2360, 1596, 1381, 1251, 955 cm^{-1}.

HRMS (ESI) m/z: $[M + Na]^+$ Calcd for $C_{24}H_{35}ClO_4SiNa$ 473.1885; Found 473.1884.

$[\alpha]^{25}_{D}$ = −110.1 (c=0.25, $CHCl_3$)

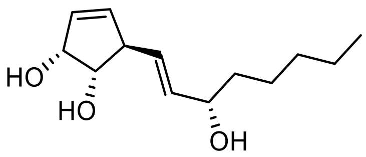

To cross-coupling product **6** (1.1 g, 2.9 mmol) was added AcOH (7 mL) and water (3 mL) and the resulting mixture was stirred at 40 ˚C using an oil bath for 16 h. The solution was concentrated under vacuum before directly loading onto a silica column. Elution with DCM/MeOH 5% yielded triol **S4** in 85% yield (560 mg) as an off-white amorphous solid.

^{1}H NMR (400 MHz, $CDCl_3$) δ 5.91 – 5.80 (m, 2H), 5.56 (dd, *J* = 4.5, 2.1 Hz, 2H), 4.55 (ddd, *J* = 5.8, 2.1, 1.3 Hz, 1H), 4.04 (dtt, *J* = 8.5, 6.3, 3.9 Hz, 1H), 3.86 (t, *J* = 5.4 Hz, 1H), 3.26 (s, 1H), 3.07 (s, 3H), 1.59 – 1.40 (m, 2H), 1.40 – 1.20 (m, 6H), 0.87 (t, *J* = 6.6 Hz, 3H).

^{13}C NMR (101 MHz, $CDCl_3$) δ 137.4, 134.7, 131.5, 130.9, 74.8, 72.9, 53.7, 37.1, 31.7, 25.2, 24.8, 22.6, 14.1.

IR 3340, 2968, 2886, 1465, 1369, 1331, 1307, 1160, 951, 628 cm^{-1}

HRMS (ESI) m/z: $[M + Na]^+$ Calcd for $C_{13}H_{22}O_3Na$ 249.1461; Found 249.1460.

$[\alpha]^{25}_D$ = −165.8 (c= 0.5, $CHCl_3$)

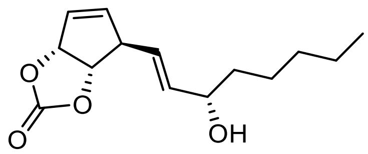

Triol **S4** (450 mg, 2.0 mmol) was dissolved in DCM, followed by the addition of Et_3N (3 mL, 16 mmol, 8 equiv) and CDI (1.80 g, 8 mmol, 4 equiv). The reaction was stirred at 50 ˚C using an oil bath for 2 hours and treated with sat. aq. NH_4Cl (20 mL). The aqueous layer was extracted with DCM (3 x 30 mL) and the combined organic layers were dried over Na_2SO_4, filtered and concentrated under vacuum. The crude product was purified by column chromatography (MeOH/DCM 2.5%), furnishing carbonate **14** as a colourless oil in 94% yield (469 mg).

^{1}H NMR (400 MHz, $CDCl_3$) δ 6.10 (dd, *J* = 5.8, 2.4 Hz, 1H), 5.95 (dt, *J* = 5.8, 1.9 Hz, 1H), 5.69 – 5.49 (m, 3H), 4.86 (d, *J* = 6.5 Hz, 1H), 4.15 – 4.05 (m, 1H), 3.74 – 3.67 (m, 1H), 1.50 (td, *J* = 7.2, 2.9 Hz, 2H), 1.33 – 1.23 (m, 6H), 0.93 – 0.85 (m, 3H).

^{13}C NMR (101 MHz, $CDCl_3$) δ 154.4, 139.2, 136.5, 128.0, 127.5, 84.7, 82.9, 72.1, 53.5, 37.3, 31.7, 25.0, 22.6, 14.0.

IR (neat) 2980, 2360, 1793, 1461, 1380, 1156, 769 cm^{-1}.

HRMS (ESI) m/z: $[M + Na]^+$ Calcd for $C_{14}H_{20}O_4Na$ 275.1254; Found 275.1250.

$[\alpha]^{25}_D$ = −239.2 (c=0.11, $CHCl_3$)

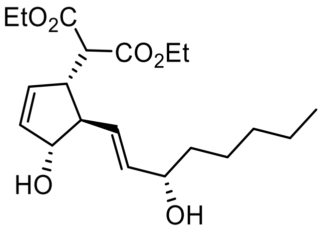

Dimethyl malonate (0.72 mL, 4.7 mmol, 3.5 equiv) was added via syringe to a suspension of NaH (60% in mineral oil, washed with pentane, 219 mg, 4.0 mmol, 3 equiv) in THF (7 mL) under argon atmosphere at 0 ˚C. After stirring for 5 minutes, this solution was transferred to a flask containing carbonate **14** (160 mg, 1.35 mmol, 1 equiv) and Pd(dppf)Cl_2 (19 mg, 0.27 mmol, 2 mol %) in THF (22 mol). After stirring for 1 hour at room temperature, the reaction solution was poured into aq. Sat. NH_4Cl (15 mL) and extracted with EtOAc (3 x 20 mL). The combined organic layers were dried over Na_2SO_4, filtered, and concentrated under vacuum. Silica gel chromatography (40% EtOAc in Hexane) yielded the desired product **15** in 96% yield (477 mg) as colourless oil.

^{1}H NMR (400 MHz, $CDCl_3$) δ 5.86 (s, 2H), 5.71 – 5.46 (m, 2H), 4.50 (t, *J* = 5.7 Hz, 1H), 4.26 – 4.12 (m, 4H), 4.11 – 3.98 (m, 1H), 3.43 (dd, *J* = 6.4, 1.1 Hz, 1H), 3.17 – 2.99 (m, 1H), 2.57 – 2.37 (m, 1H), 1.61 – 1.36 (m, 3H), 1.33 – 1.21 (m, 11 H), 0.95 – 0.82 (m, 3H).

^{13}C NMR (101 MHz, $CDCl_3$) δ 168.5, 168.2, 135.0, 134.4, 133.4, 131.5, 82.6, 72.6, 61.5, 61.5, 55.1, 54.7, 49.6, 37.3, 31.8, 25.2, 22.6, 14.2, 14.0.

IR (neat) 2980, 2888, 2360, 2341, 1732, 1461, 1381, 1252, 966 cm^{-1}.

HRMS (ESI) m/z: $[M + Na]^+$ Calcd for $C_{20}H_{32}O_6Na$ 391.2091; Found 391.2082.

$[\alpha]^{25}_D$ = +34 (c=0.4, $CHCl_3$)

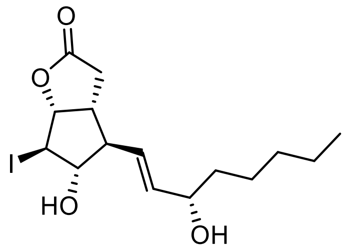

An aq. Solution of NaOH (1M, 18 mL, 18 mmol, 18 equiv) was added to a solution of diester **15** (360 mg, 1 mmol, 1 equiv) in THF (10 mL) at RT and stirred for 24 h. The solution was acidified with an aq. Solution of HCL (1 M, 60 mL) and extracted with EtOAc (3 x 50 mL). The combined organic layers were dried over Na_2SO_4 and concentrated under vacuum. The resulting pale-yellow oil was used in the next step without further purification.

The crude diacid was dissolved in THF (22 mL) and CDI (450 mg, 2.5 mmol, 2.5 equiv) was added at RT. After 2 hours, an aq. Solution of NaOH (22 mL, 22 mmol, 25 equiv) was added and the reaction mixture was stirred for 14 hours. The solution was acidified with an aq. Solution of HCL (1 M, 60 mL) and extracted with EtOAc (3 x 50 mL). The combined organic layers were dried over Na_2SO_4 and concentrated under vacuum. The resulting carboxylic acid **16** was used in the next step without further purification.

The crude carboxylic acid **16** was dissolved in THF (5 mL) and a solution of $NaHCO_3$ (640 mg, 17.9 mmol, 2 equiv) in H_2O (6 mL) was added. A solution of KI (989 mg, 6.1 mmol, 6 equiv) and I_2 (510 mg, 2.0 mmol, 2 equiv) was also added and the mixture was stirred for 24 h in the dark. The reaction mixture was poured onto a sat. aq. solution of $Na_2S_2O_3$ (40 mL) and was extracted with EtOAc (3 x 50 mL). The combined organic layers were dried over Na_2SO_4 and concentrated under vacuum. Purificaiton by silica gel chromatography (15% EtOAc in Hexane) to yield the iodolactonized **S5** product as a pale yellow oil in a 69% yield (274 mg) over 3 steps.

^{1}H NMR (400 MHz, Acetone) δ 5.91 – 5.61 (m, 2H), 5.18 (dd, *J* = 8.0, 4.8 Hz, 1H), 4.89 (d, *J* = 6.2 Hz, 1H), 4.14 (dd, *J* = 5.3, 4.2 Hz, 2H), 4.08 (td, *J* = 9.5, 6.1 Hz, 1H), 3.75 (d, *J* = 4.4 Hz, 1H), 3.05 – 2.85 (m, 8H), 2.46 (dd, *J* = 17.7, 2.3 Hz, 1H), 2.34 (td, *J* = 9.9, 6.9 Hz, 1H), 1.69 – 1.47 (m, 1H), 1.46 – 1.34 (m, 6H), 1.10 – 0.92 (m, 4H).

^{13}C NMR (101 MHz, Acetone) δ 175.6, 137.9, 128.4, 89.9, 84.0, 72.1, 54.1, 41.4, 38.1, 33.9, 33.3, 32.3, 25.7, 23.0, 14.0.

IR (neat) 2980, 2360, 2341, 1773, 1381, 1252, 967, 668 cm^{-1}.

HRMS (ESI) m/z: $[M + Na]^+$ Calcd for $C_{15}H_{23}IO_4Na$ 417.0533; Found 417.0526.

$[\alpha]^{25}_D$ = −14.8 (c=0.16, $CHCl_3$)

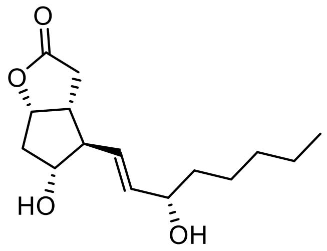

Tributyltinhydride (0.3 mL, 1.17 mmol, 5.0 equiv.) and azobisisobutyronitrile (AIBN) (6 mg, 39 µmol, 0.15 equiv.) were added to solution of iodolactone **S5** (90 mg, 0.23 mmol, 1.00 equiv.) in benzene (2.6 mL). The resulting mixture was stirred at 80 ˚C using an oil bath. After 1 h, the reaction mixture was cooled down to room temperature, solid KF (200 mg) was added and the resulting mixture was stirred for additional 1 h. The reaction mixture was then loaded directly on silica and the crude product purified by silica gel column chromatography (ethyl acetate/hexane = 30% to 70%, stationary phase K_2CO_3/silica = 1:9). The product **17** was obtained as a colorless oil (48 mg, 78%).

^{1}H NMR (400 MHz, $CDCl_3$) δ 5.55 (dd, *J* = 15.3, 7.4 Hz, 1H), 5.39 (dd, *J* = 15.2, 8.5 Hz, 1H), 4.87 (td, *J* = 7.0, 3.1 Hz, 1H), 4.00 (q, *J* = 6.8 Hz, 1H), 3.88 (q, *J* = 7.8 Hz, 1H), 2.72 (dd, *J* = 18.2, 9.5 Hz 1H), 2.51 (dq, *J* = 14.1, 7.7 Hz, 2H), 2.37 (dd, *J* = 17.9 Hz, 1H), 2.20 (q, *J* = 8.5 Hz, 1H), 1.88 (ddd, *J* = 14.7, 8.2, 3.2 Hz, 1H), 1.56-1.48 (m, 2H), 1.46-1.20 (m, 7H), 0.93 – 0.82 (m, 3H).

^{13}C NMR (101 MHz, $CDCl_3$) δ 177.2, 137.0, 130.5, 82.6, 76.3, 73.0, 56.2, 42.4, 39.7, 37.1, 34.1, 31.7, 25.2, 22.7, 14.1.

HRMS (ESI) m/z: $[M + H]^+$ Calcd for $C_{15}H_{24}O_4$ 269.1747; Found 269.1746.

$[\alpha]^{25}_D$ = −4.8 (c=0.15, $CHCl_3$) [−4.7, c = 0.72 $CHCl_3$],[1] [−7.2, c = 0.3, $CHCl_3$],[2] [−6.7, c = 1.0 $CHCl_3$].[3]

All physical data is in accordance with literature.[1-3]

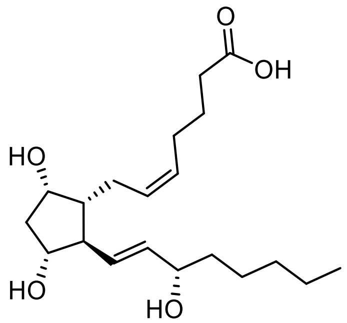

Lactone **17** (25 mg, 93 µmol, 1 equiv) was dissolved in DCM (2.5 mL) and DIBAL-H was added (0.38 mL, 380 µmol, 4 equiv) at -78 ˚C. After 1 h, the reaction was quenched with a sat. aq. solution of Rochelle's salt (20 mL) and stirred for one hour at RT. The reaction mixture was extracted with DCM (5 x 10 mL) and the combined organic layers were dried over Na_2SO_4, filtered, and concentrated under vacuum. The crude hemiacetal was used without further purification in the next step.

To a flame dried round bottom flask was added (4-Carboxybutyl)triphenylphosphonium bromide (240 mg, 0.54 mmol, 6 equiv) and dry THF (1 mL). To this was added a solution of KHMDS (0.5 M in toluene, 2.16 mmol, 1.08 mmol, 12 equiv) at 0 ˚C. After 15 minutes, a solution of hemiacetal in THF (1 mL) was added to the bright orange ylide solution. The reaction was stirred for 2 h at RT and was quenched with 3 mL of H_2O. The reaction mixture was extracted with Et_2O (2 x 10 mL). The aqueous layer was acidified with HCL (1M, 2 mL) and was extracted with DCM (10 x 5 mL). The organic phases were dired over Na_2SO_4, filtered and concentrated under vacuum. The resulting crude oil was purified by silica gel chromatography (65/30/5 EtOAc/Hex/AcOH) to yield the PG F2$_{\alpha}$ as a clear oil (27 mg, 82% over two steps).

^{1}H NMR (400 MHz, MeOD) δ 5.52 (t, *J* = 7.4 Hz, 3H), 5.44 – 5.32 (m, 1H), 4.13 (td, *J* = 5.5, 2.2 Hz, 1H), 4.05 (q, *J* = 6.4 Hz, 1H), 3.87 (q, *J* = 7.8 Hz, 1H), 2.39 (ddd, *J* = 14.4, 8.5, 5.9 Hz, 1H), 2.34 – 2.19 (m, 4H), 2.14 (tt, *J* = 8.3, 4.8 Hz, 3H), 1.73 – 1.56 (m, 4H), 1.55 – 1.45 (m, 2H), 1.45 – 1.30 (m, 9H) δ 0.94 – 0.89 (m, 3H).

^{13}C NMR (101 MHz, MeOD) δ 179.0, 136.5, 134.2, 130.7, 130.1, 77.9, 73.9, 72.2, 56.1, 50.9, 44.3, 38.4, 37.2, 33.0, 28.1, 27.1, 26.4, 26.2, 23.7, 14.4.

$[\alpha]^{25}_D$ = +23.3 (c=0.42, THF). Lit. [+23.5, c = 1.0, THF],[4] [+23.5 (natural material), c = 1.0, THF],[5] [24.9, 0.57, THF].[6]

All physical data is in accordance with literature.[4-6]

NMR Spectra:

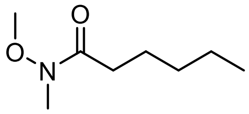

^{1}H NMR (400 MHz, $CDCl_3$) of **S1a**

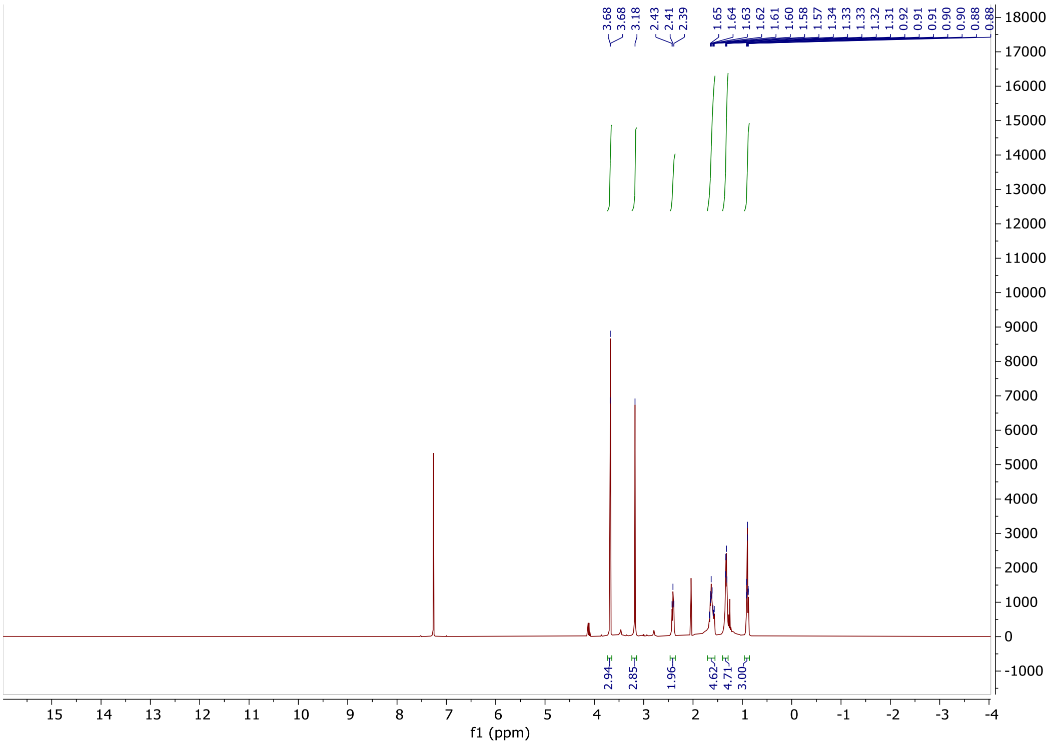

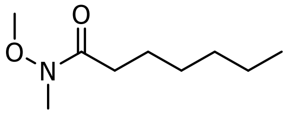

^{1}H NMR (400 MHz, $CDCl_3$) of **S1b**

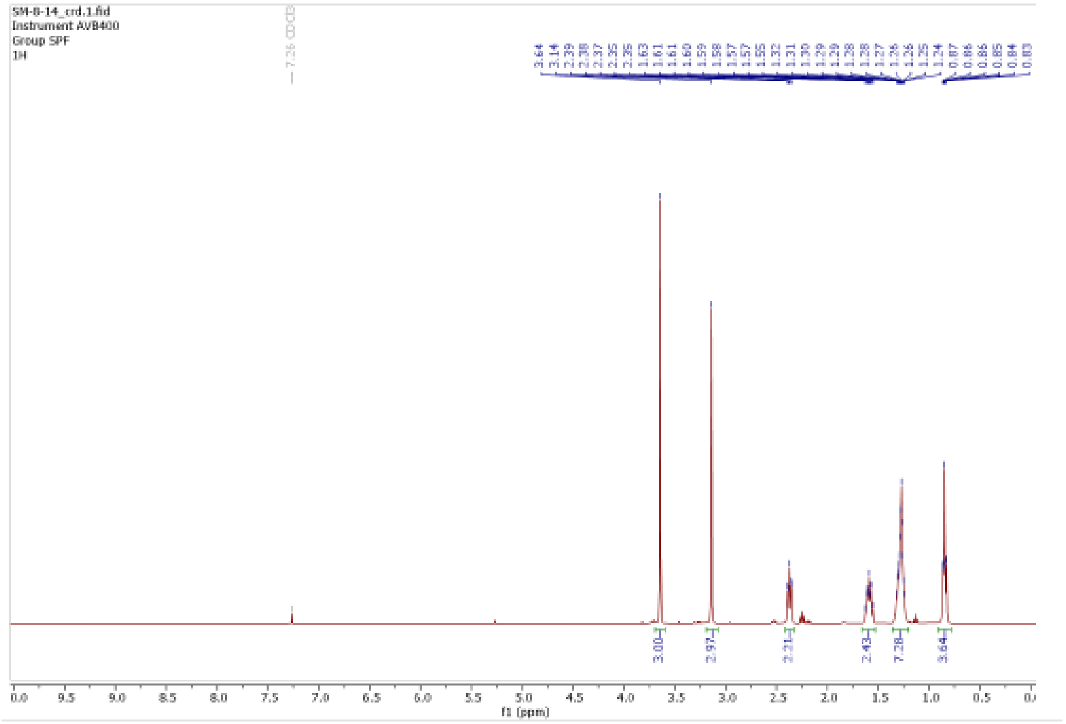

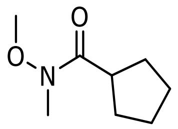

^{1}H NMR (400 MHz, $CDCl_3$) of **S1c**

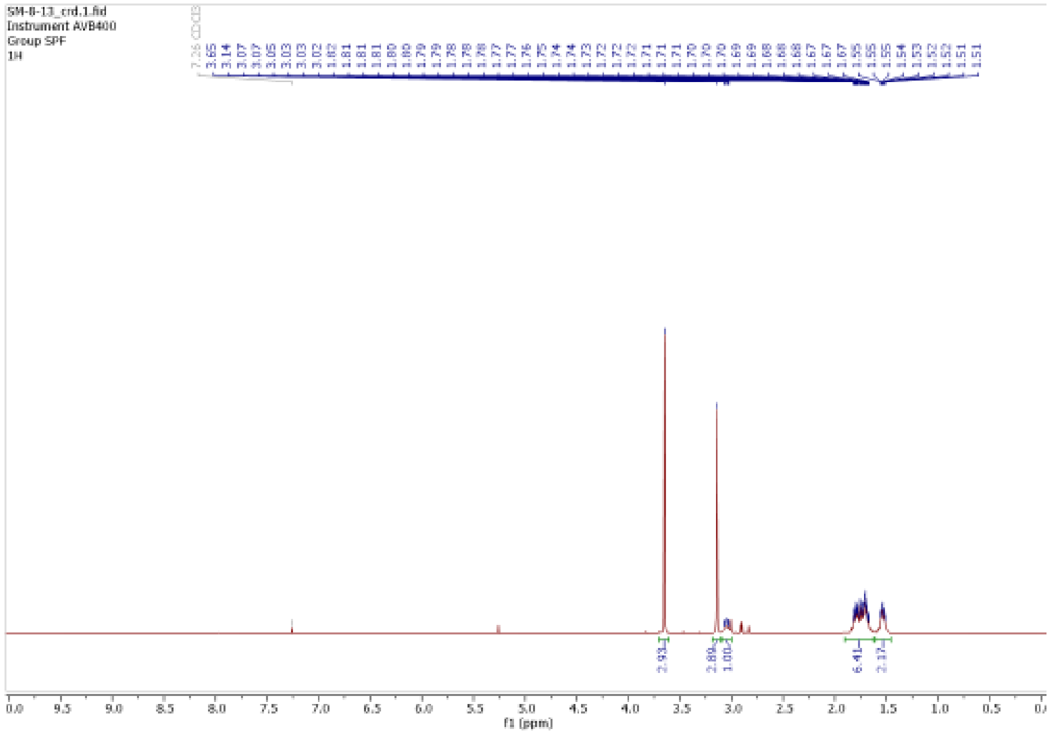

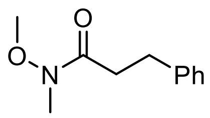

^{1}H NMR (400 MHz, $CDCl_3$) of **S1d**

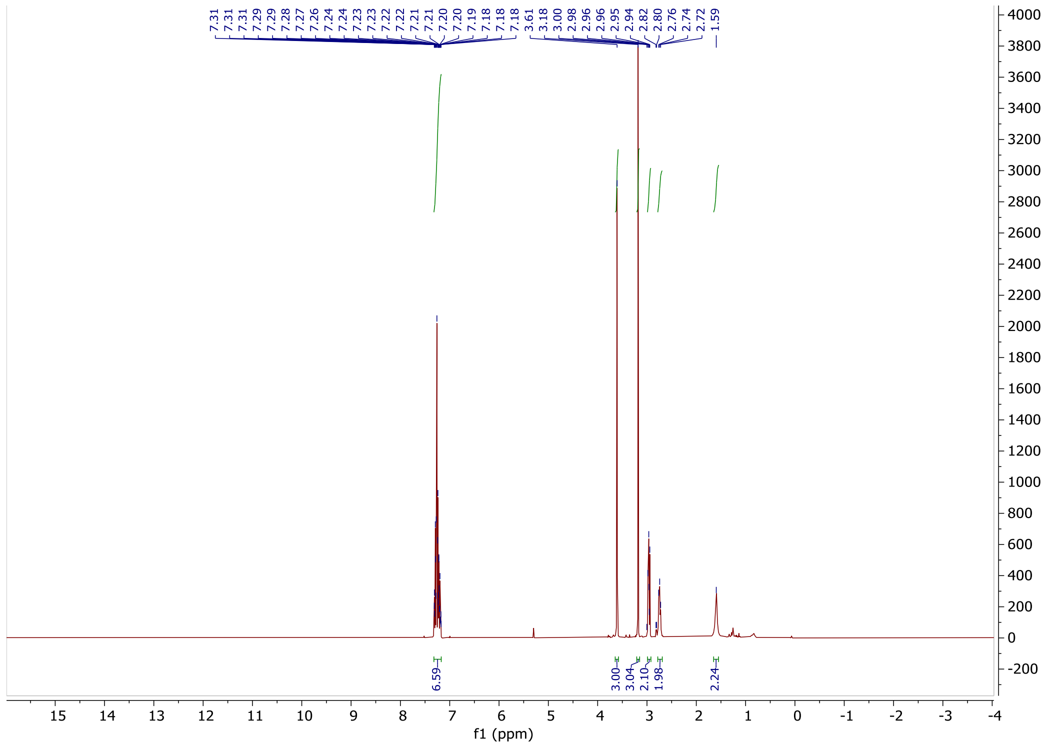

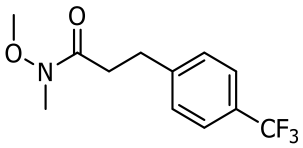

^1H NMR (400 MHz, $CDCl_3$) of **S1e**

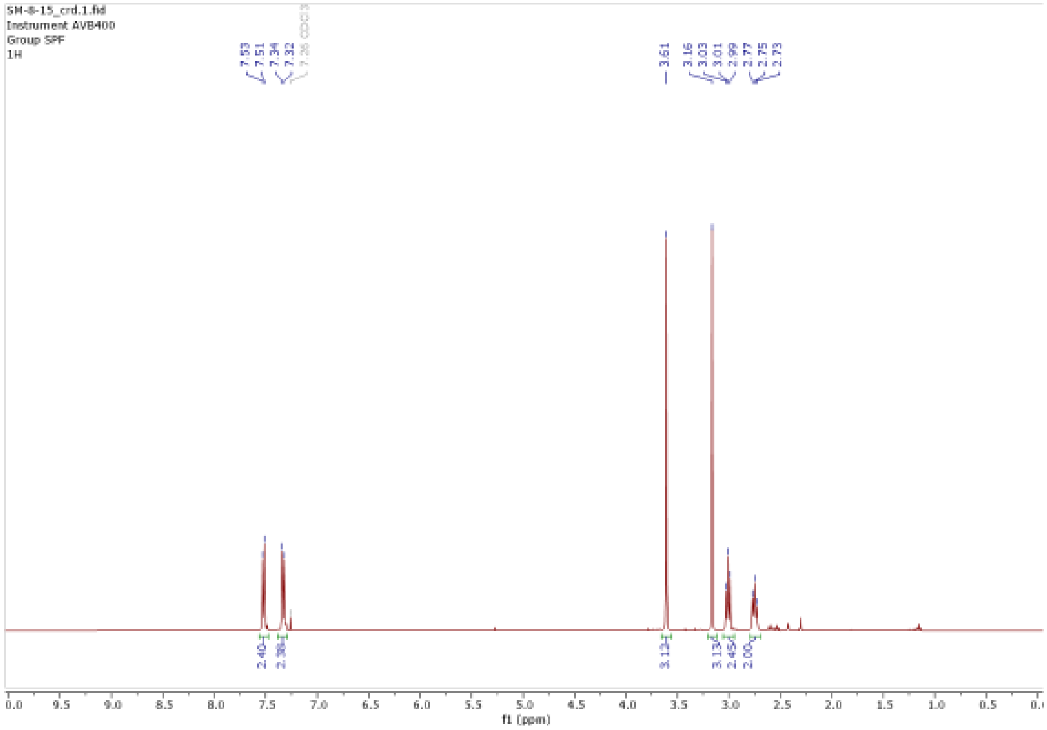

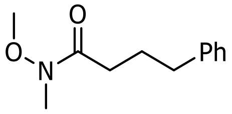

^1H NMR (400 MHz, $CDCl_3$) of **S1f**

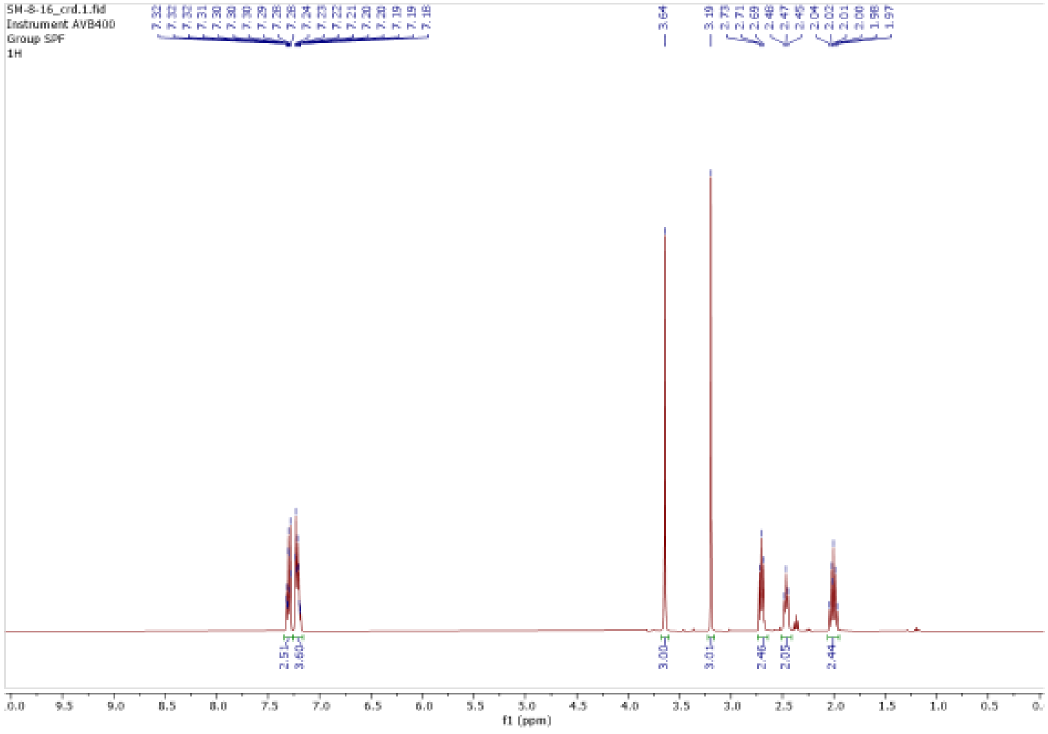

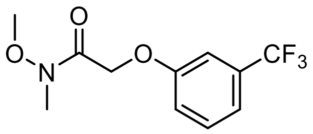

^1H NMR (400 MHz, $CDCl_3$) of **S1g**

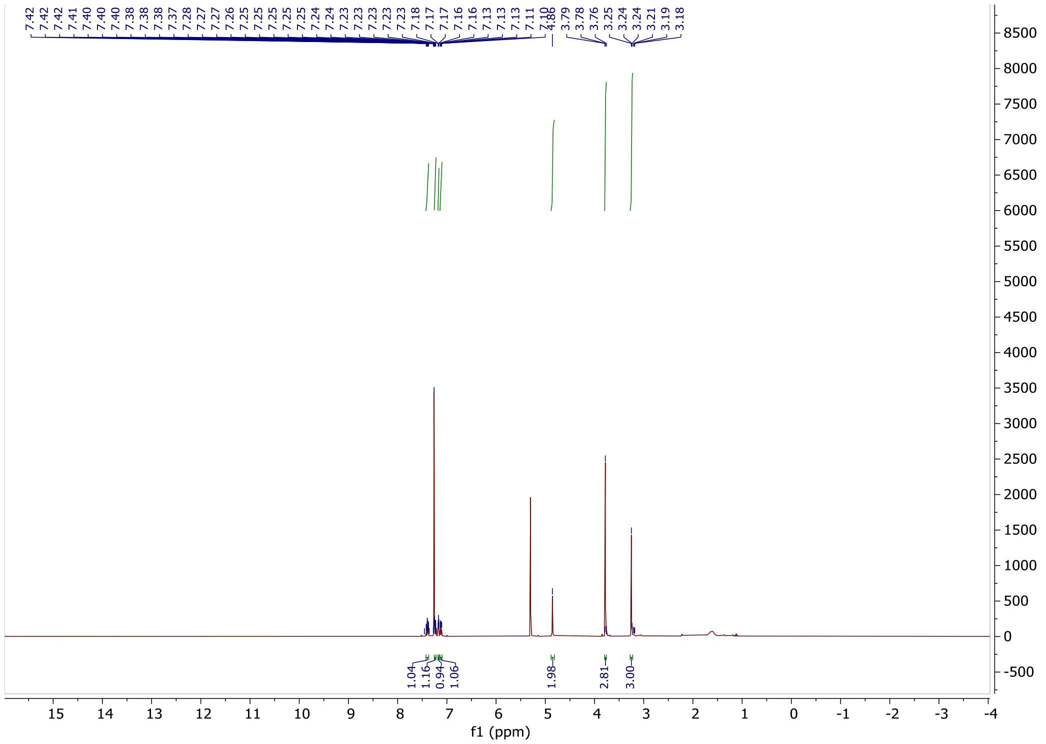

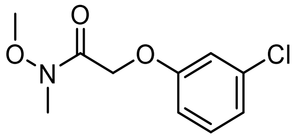

^1H NMR (400 MHz, $CDCl_3$) of **S1h**

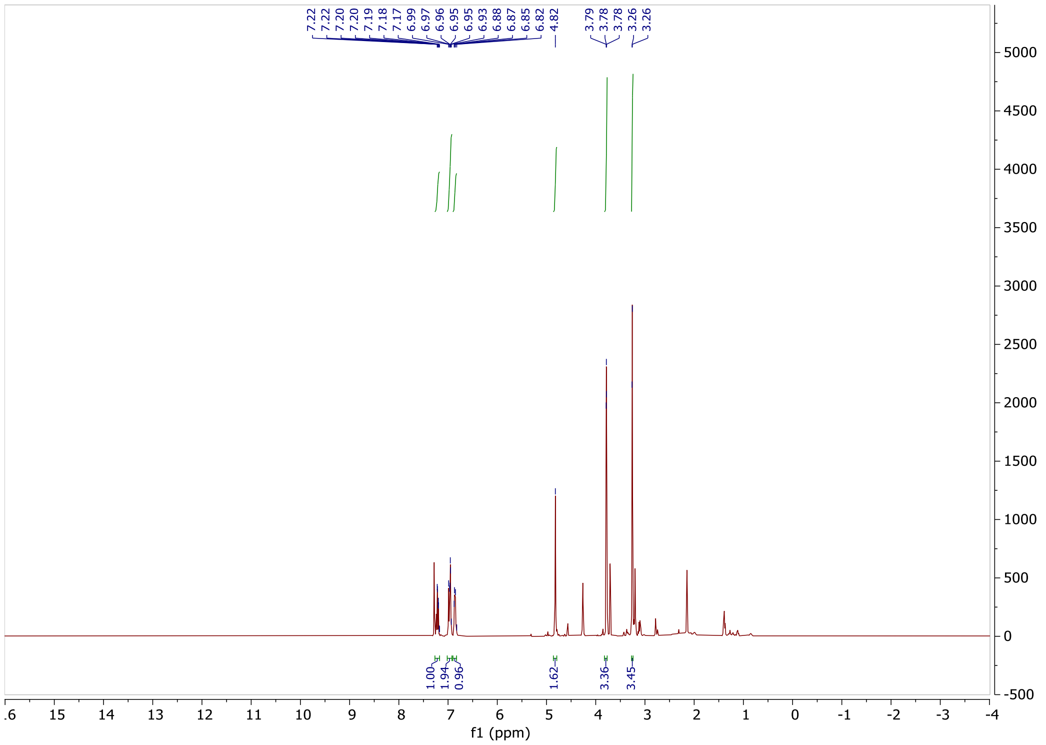

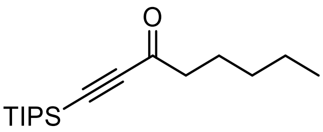

^{1}H NMR (400 MHz, $CDCl_3$) of **2a**

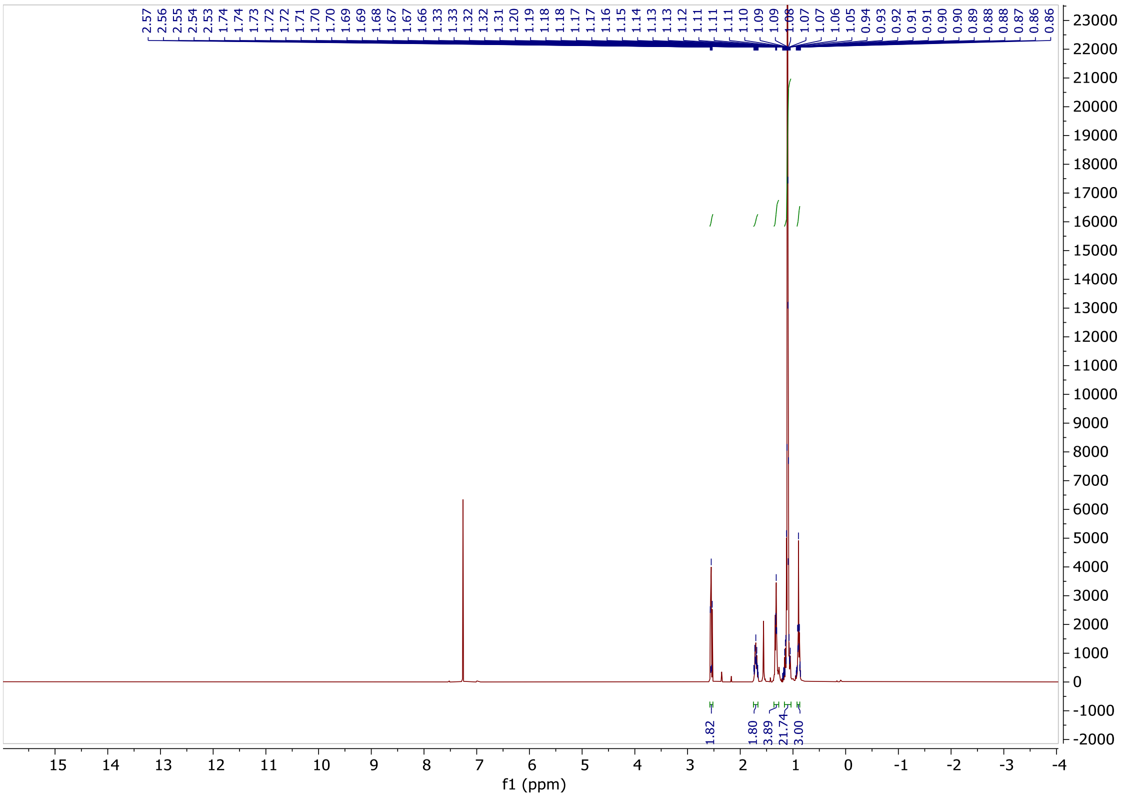

^{13}C NMR (101MHz, $CDCl_3$) of **2a**

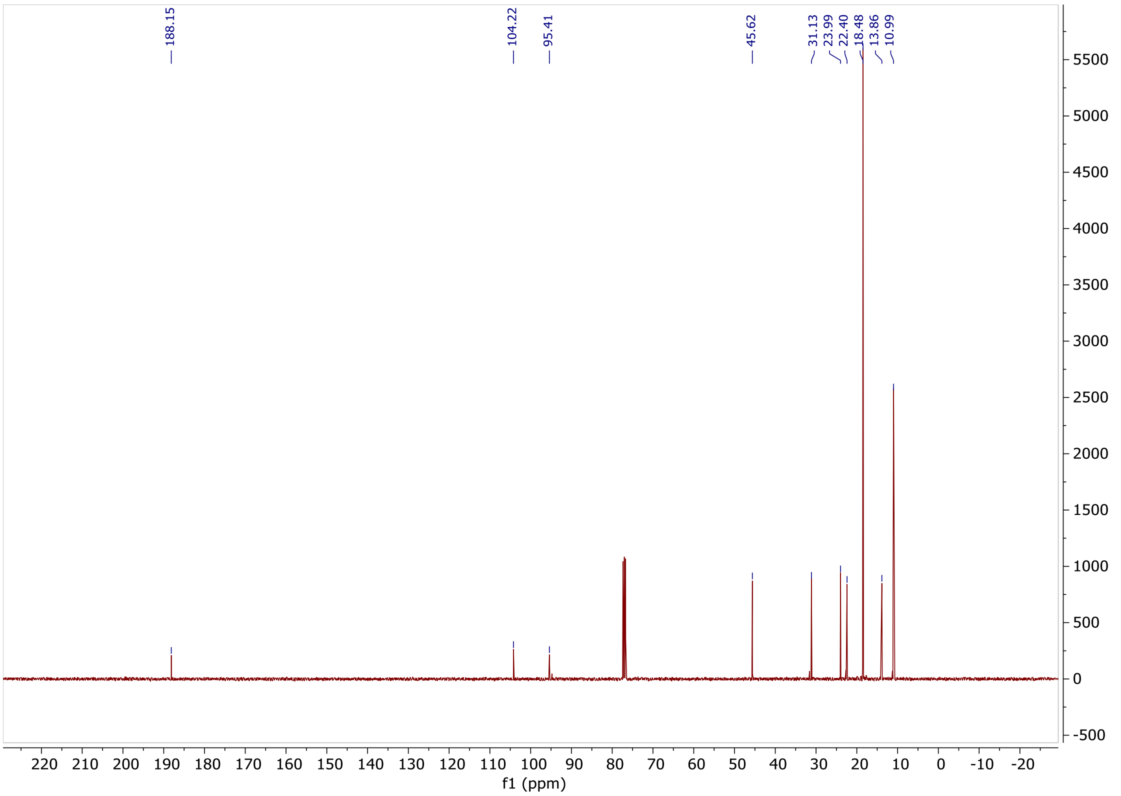

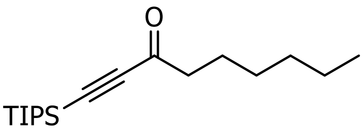

^{1}H NMR (400 MHz, $CDCl_3$) of **2b**

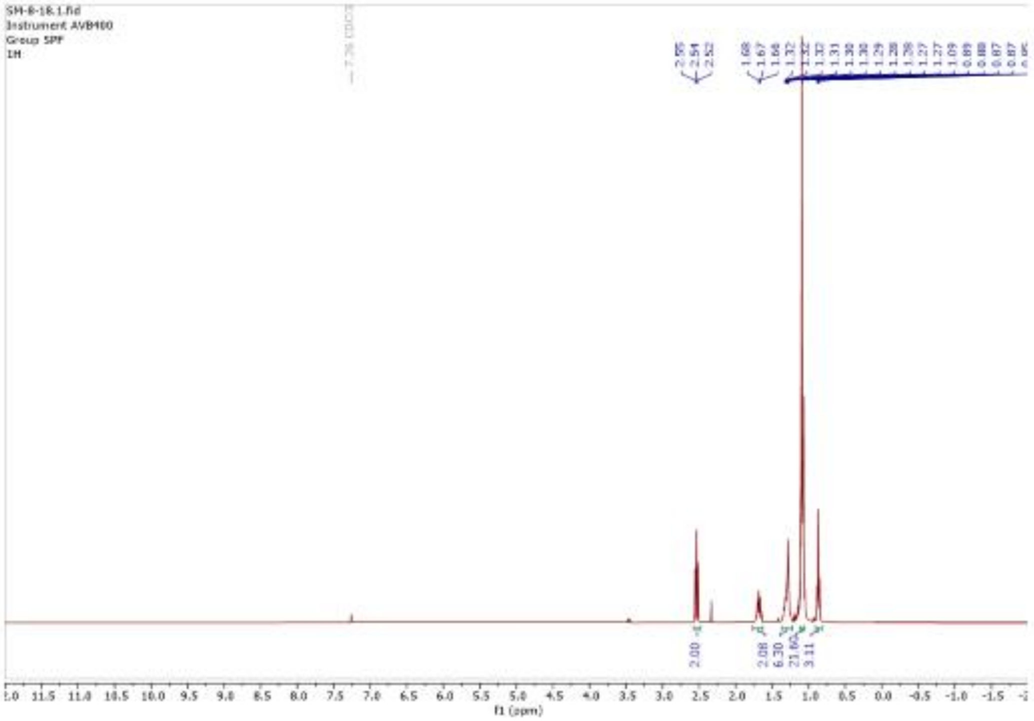

^{13}C NMR (101MHz, $CDCl_3$) of **2b**

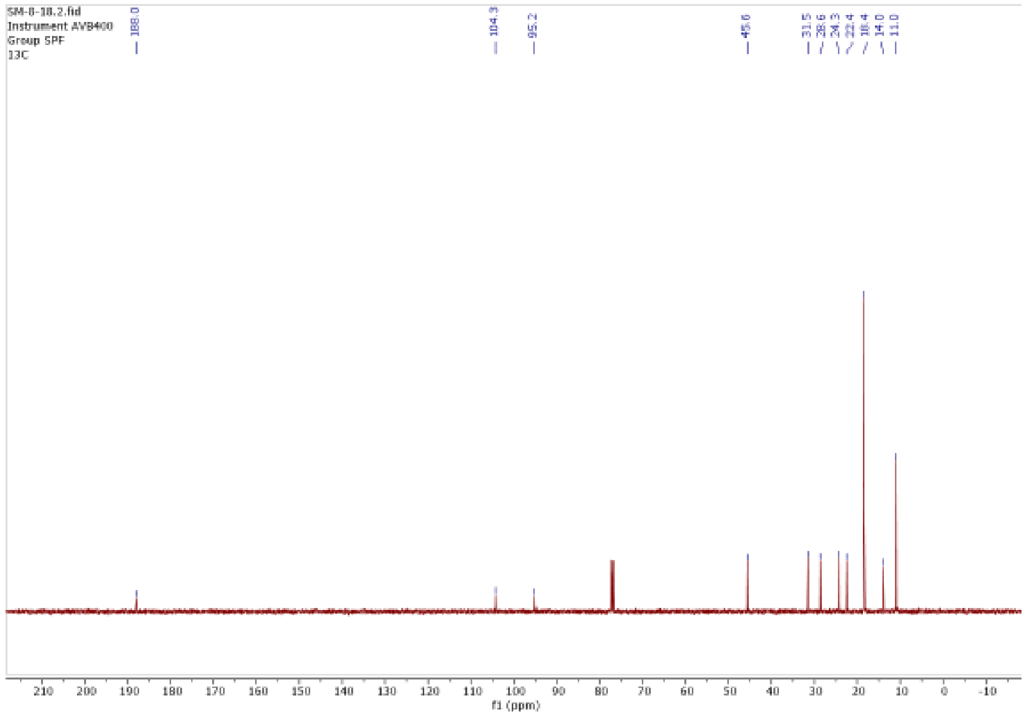

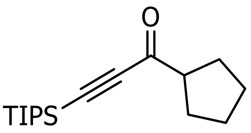

^{1}H NMR (400 MHz, $CDCl_3$) of **2c**

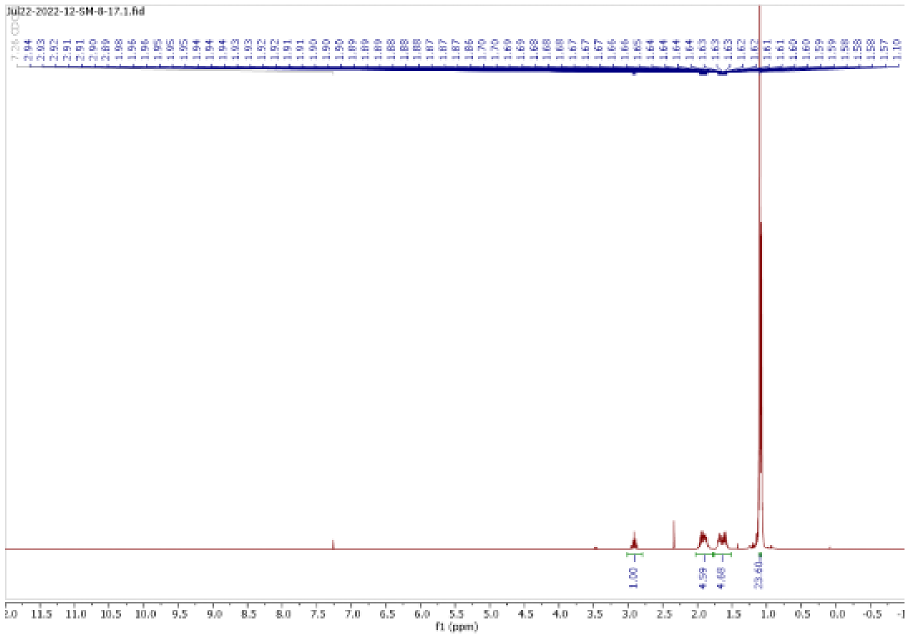

^{13}C NMR (101MHz, $CDCl_3$) of **2c**

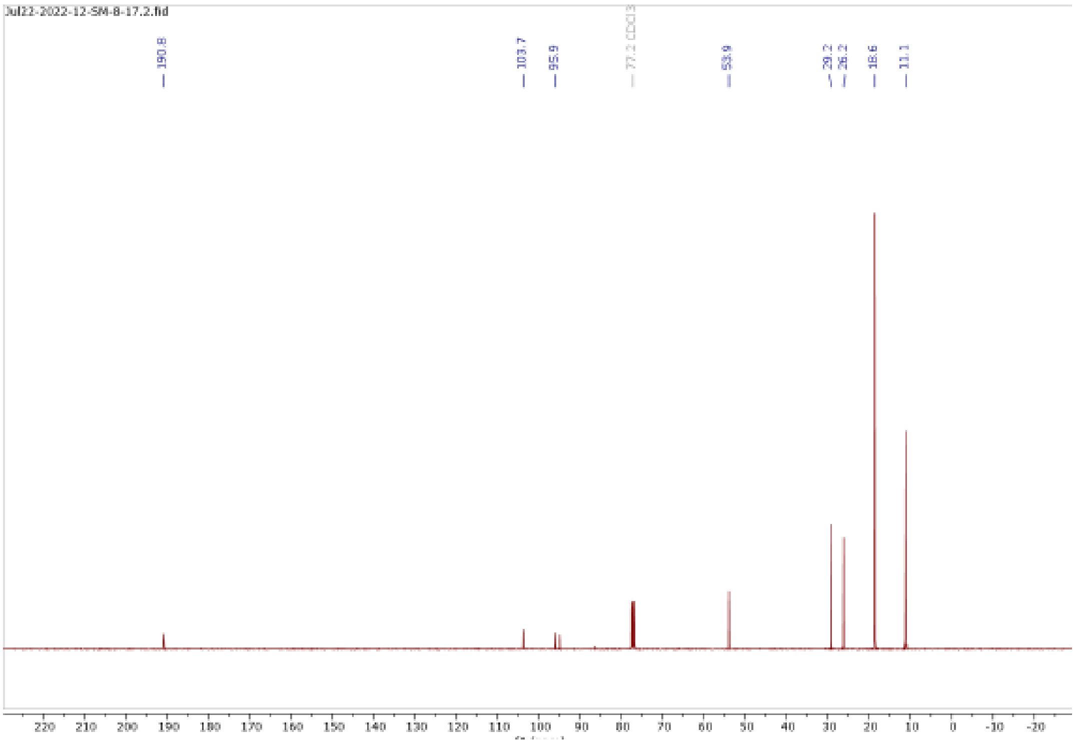

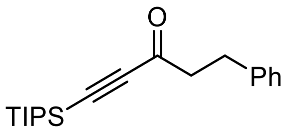

^{1}H NMR (400 MHz, $CDCl_3$) of **2d**

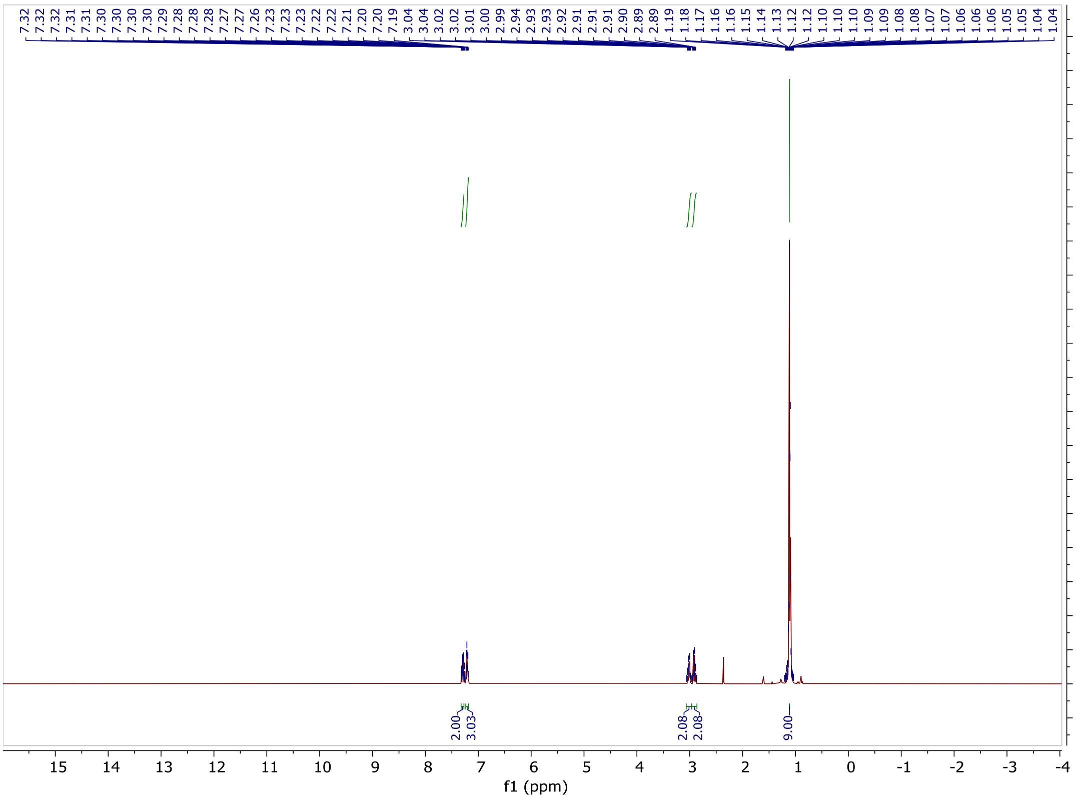

^{13}C NMR (101MHz, $CDCl_3$) of **2d**

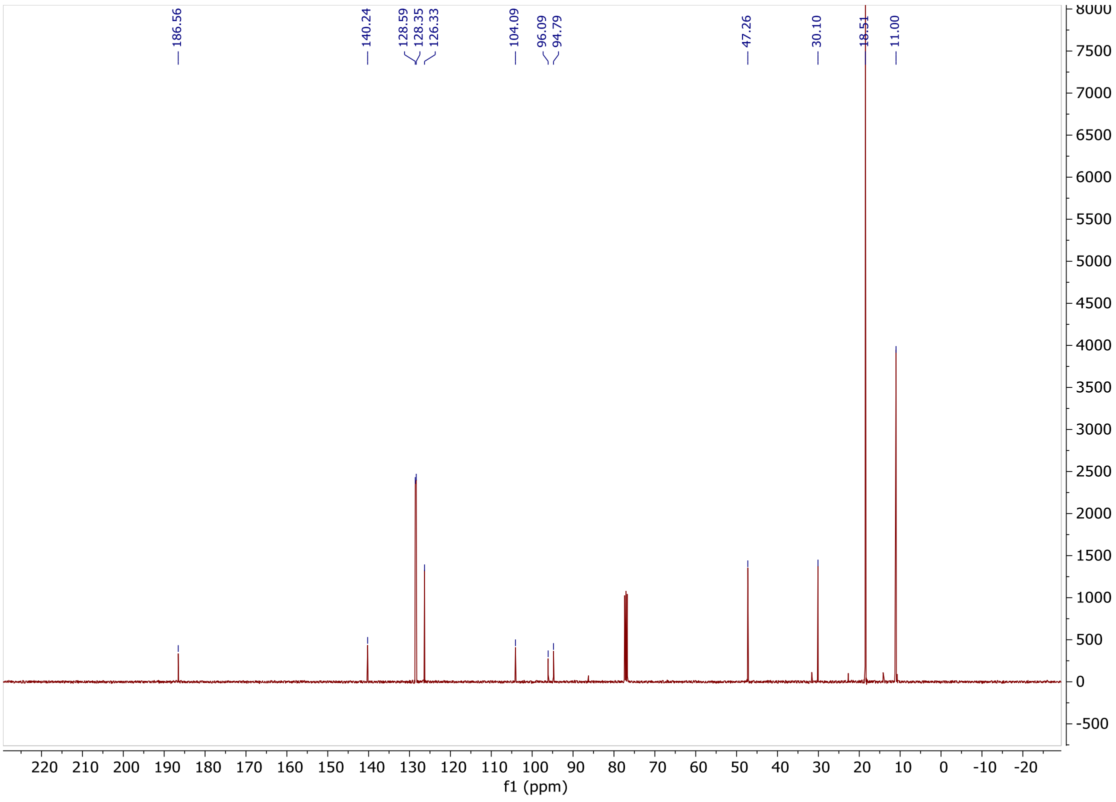

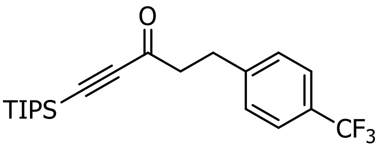

^{1}H NMR (400 MHz, $CDCl_3$) of **2e**

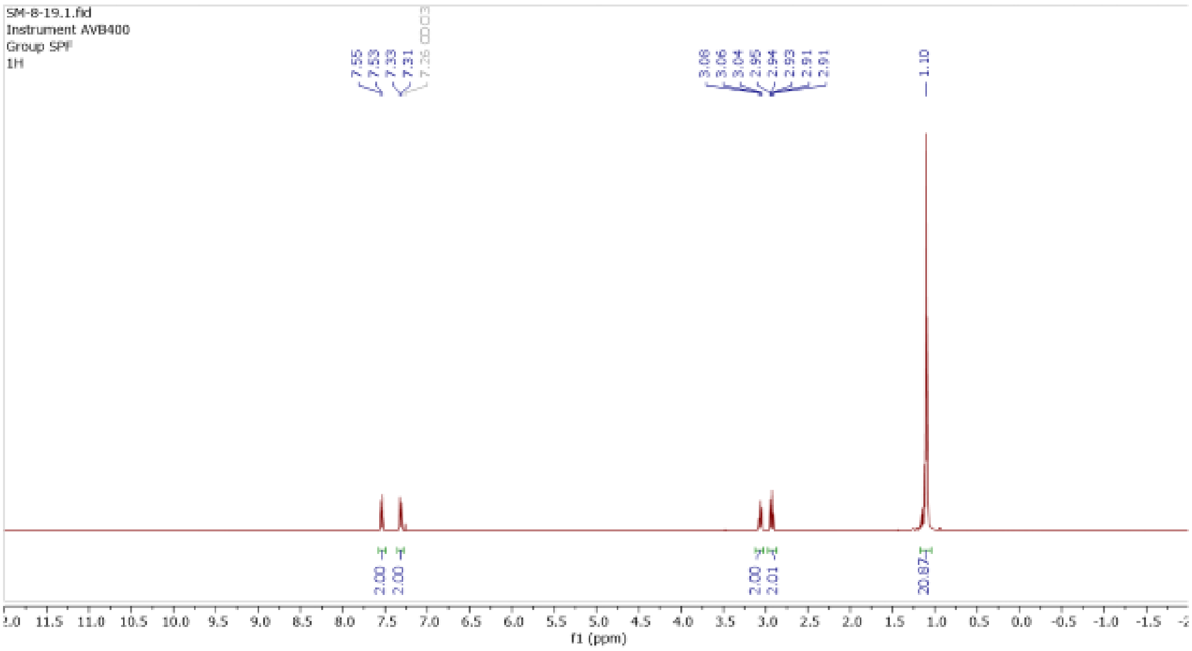

^{13}C NMR (101MHz, $CDCl_3$) of **2e**

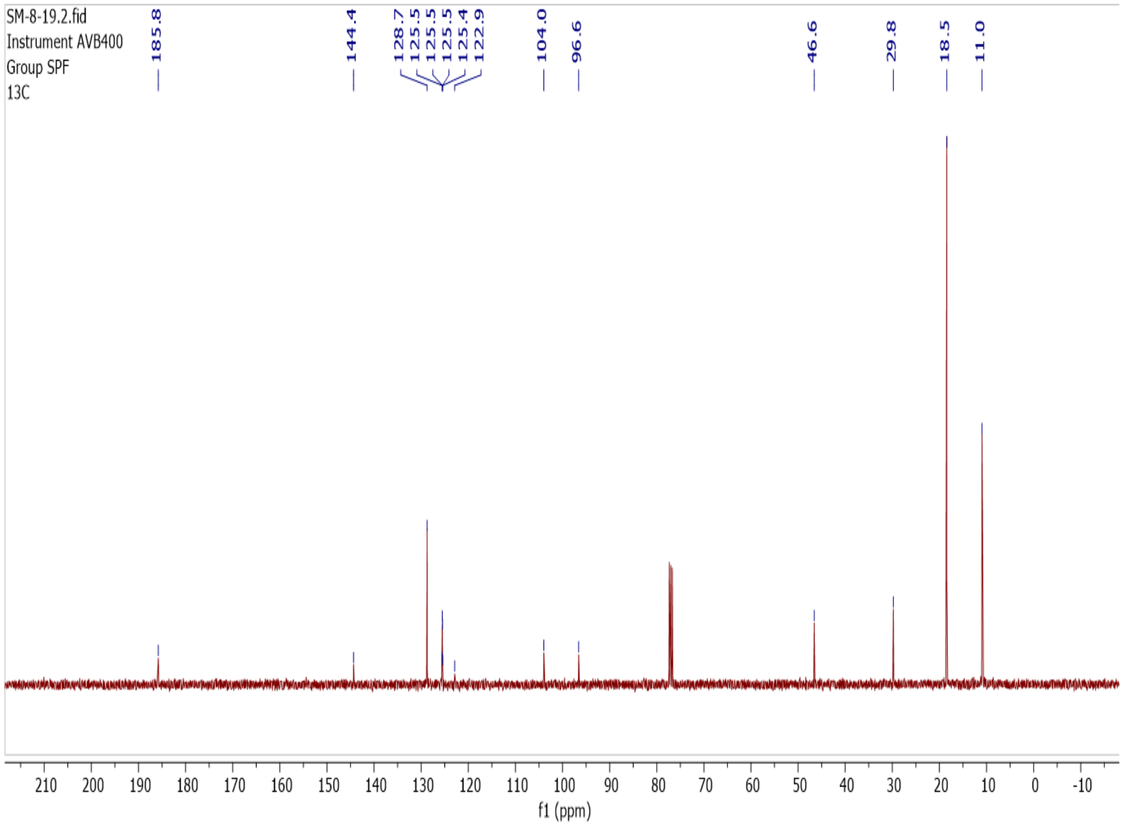

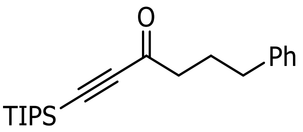

^{1}H NMR (400 MHz, $CDCl_3$) of **2f**

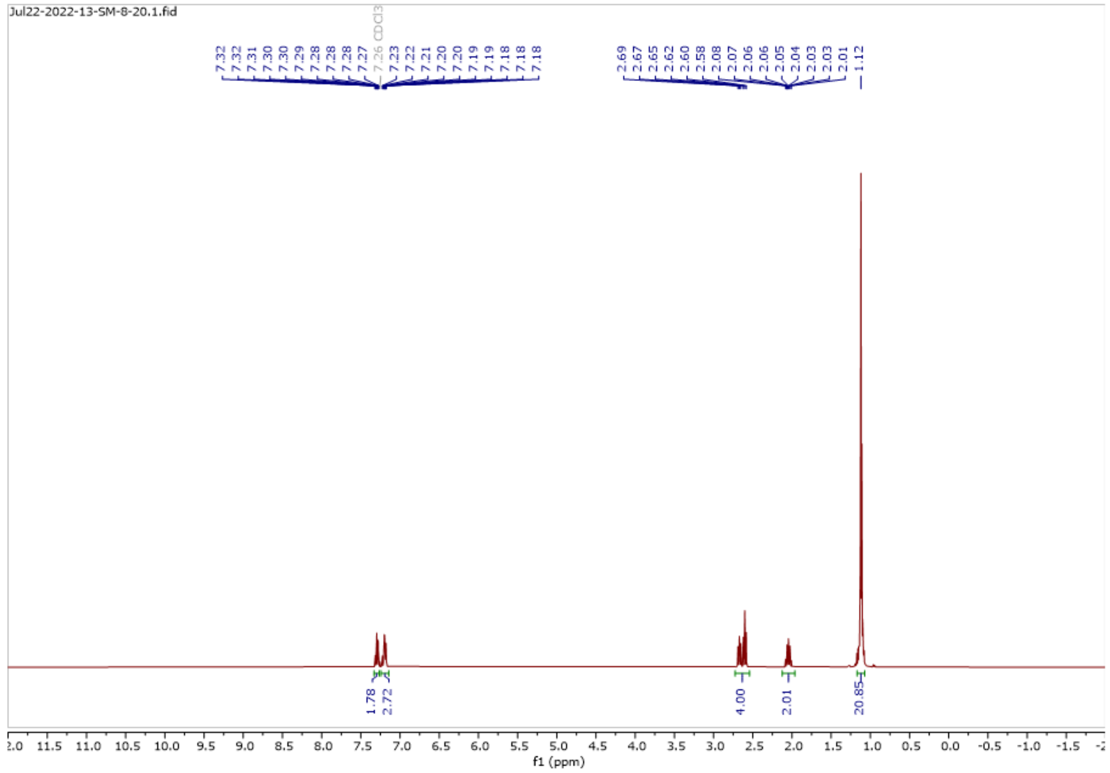

^{13}C NMR (101MHz, $CDCl_3$) of **2f**

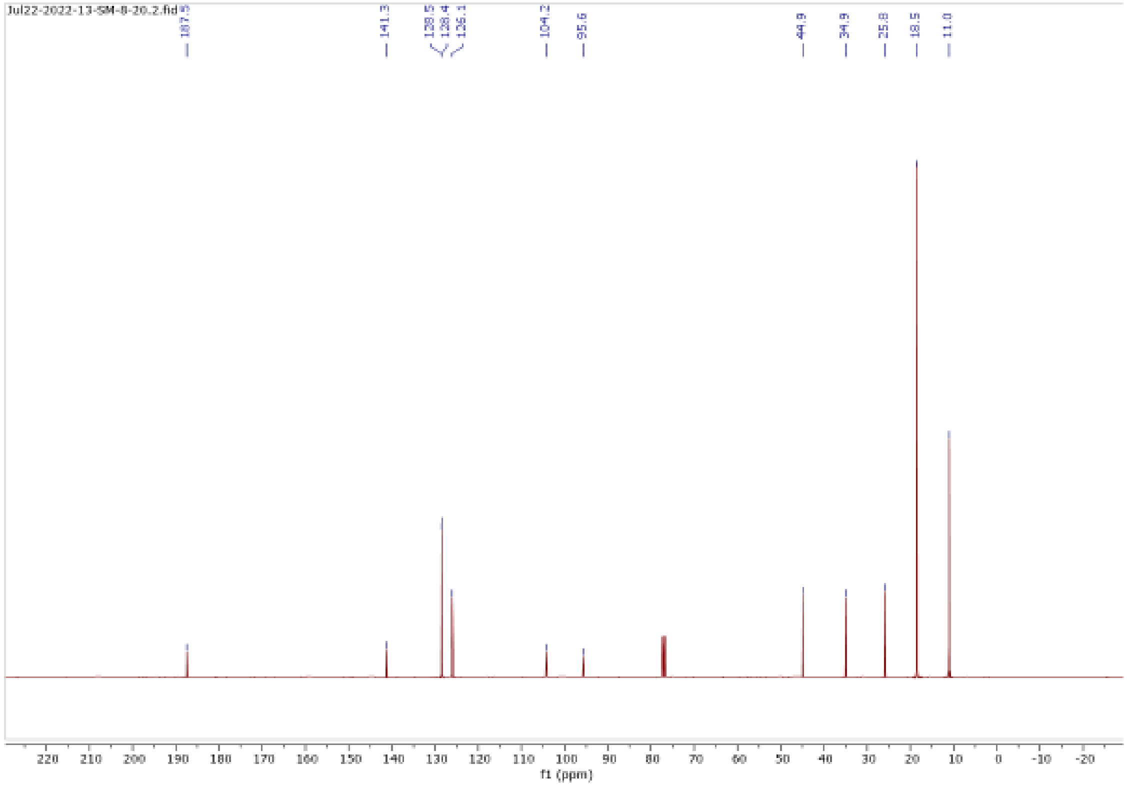

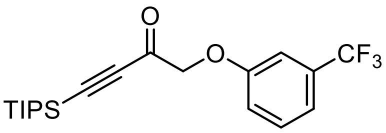

^{1}H NMR (400 MHz, $CDCl_3$) of **2g**

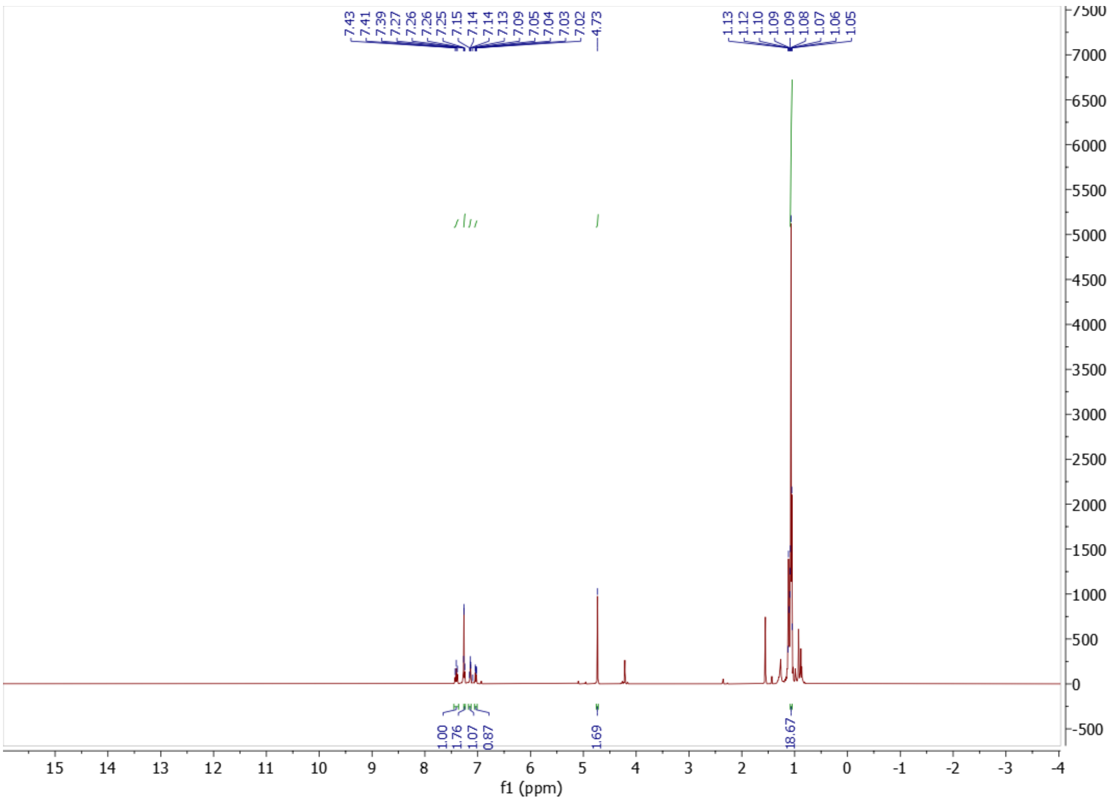

^{13}C NMR (101MHz, $CDCl_3$) of **2g**

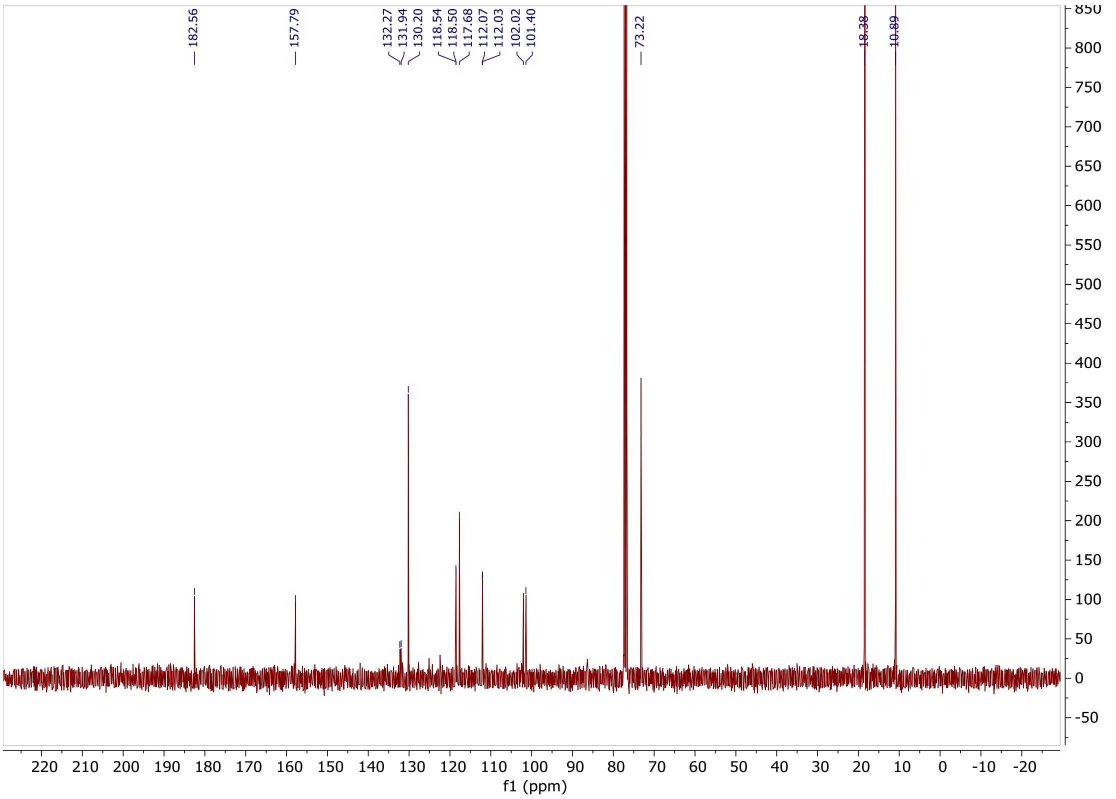

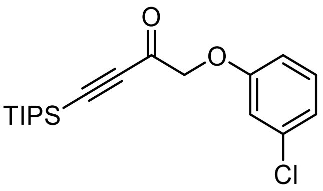

^{1}H NMR (400 MHz, $CDCl_3$) of **2h**

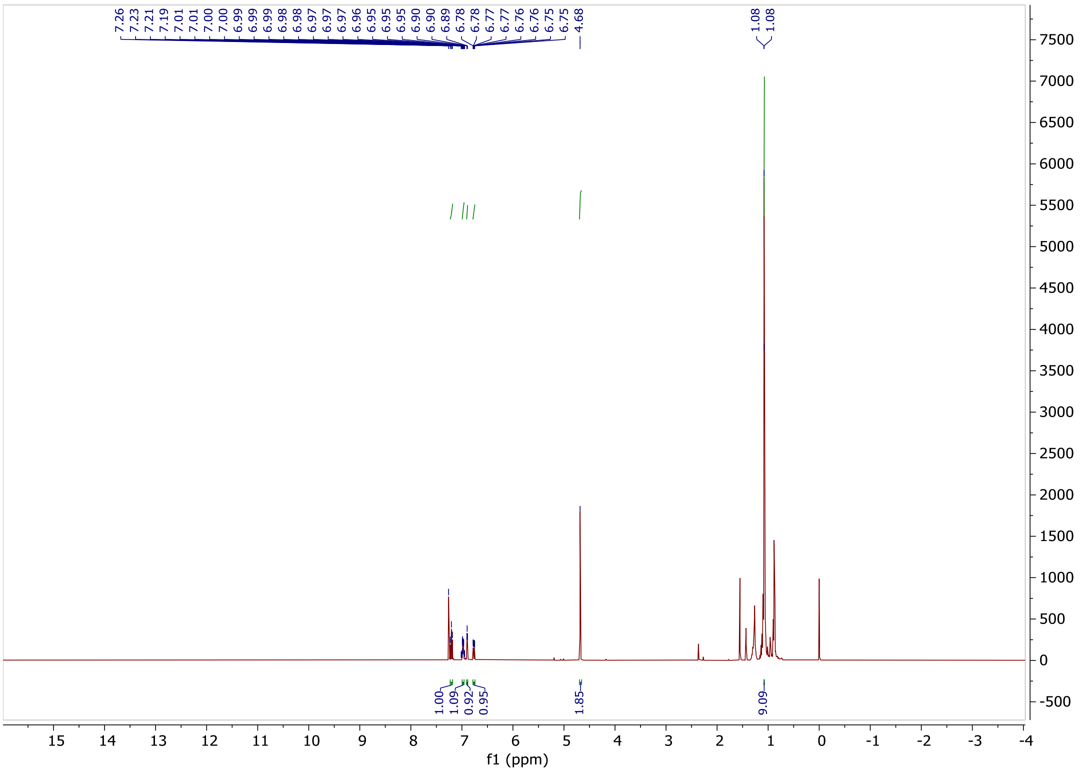

^{13}C NMR (101MHz, $CDCl_3$) of **2h**

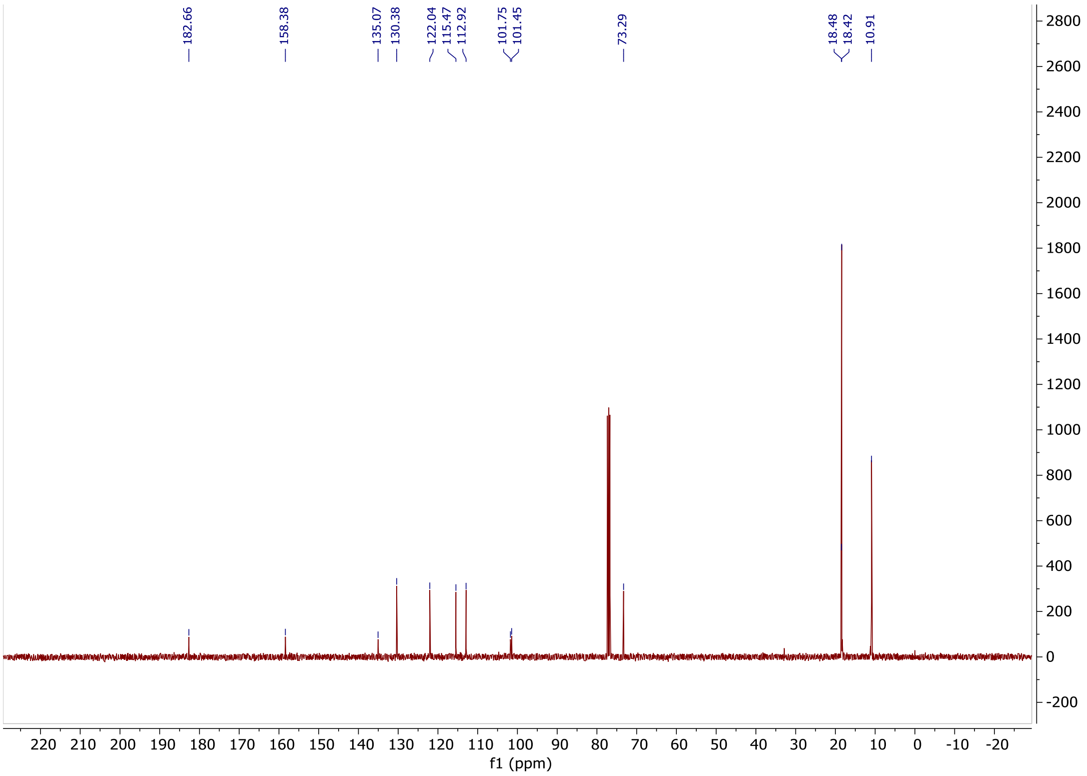

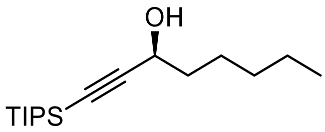

^{1}H NMR (400 MHz, $CDCl_3$) of **3a**

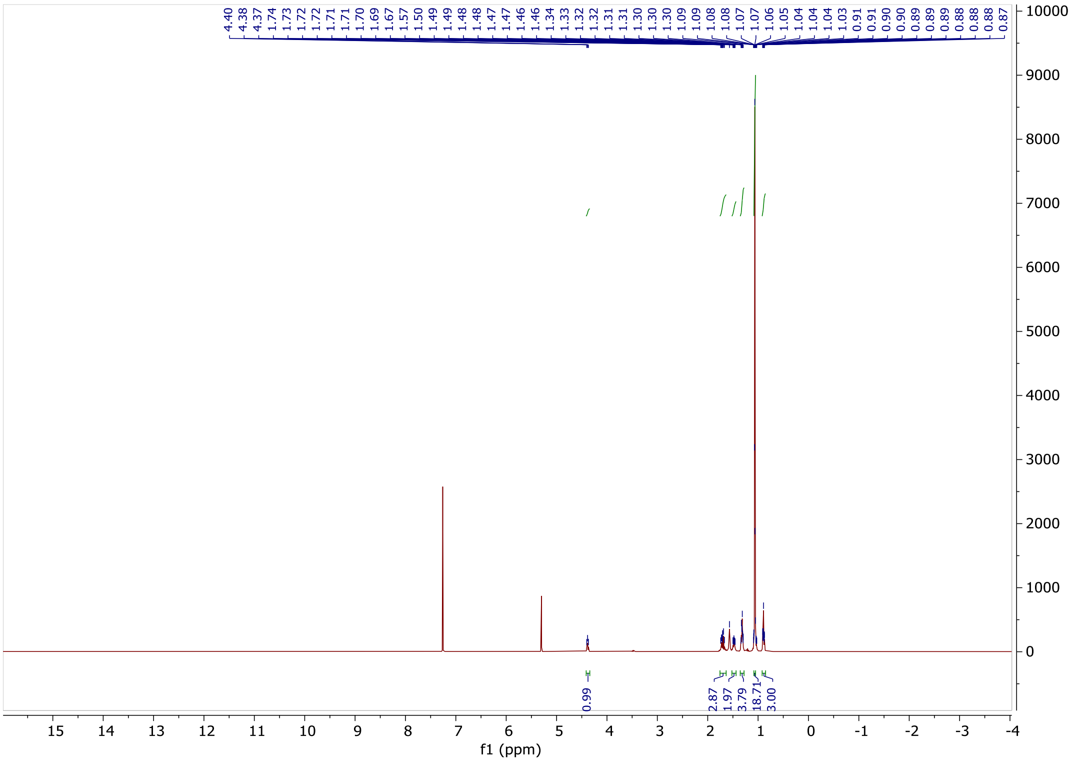

^{13}C NMR (101MHz, $CDCl_3$) of **3a**

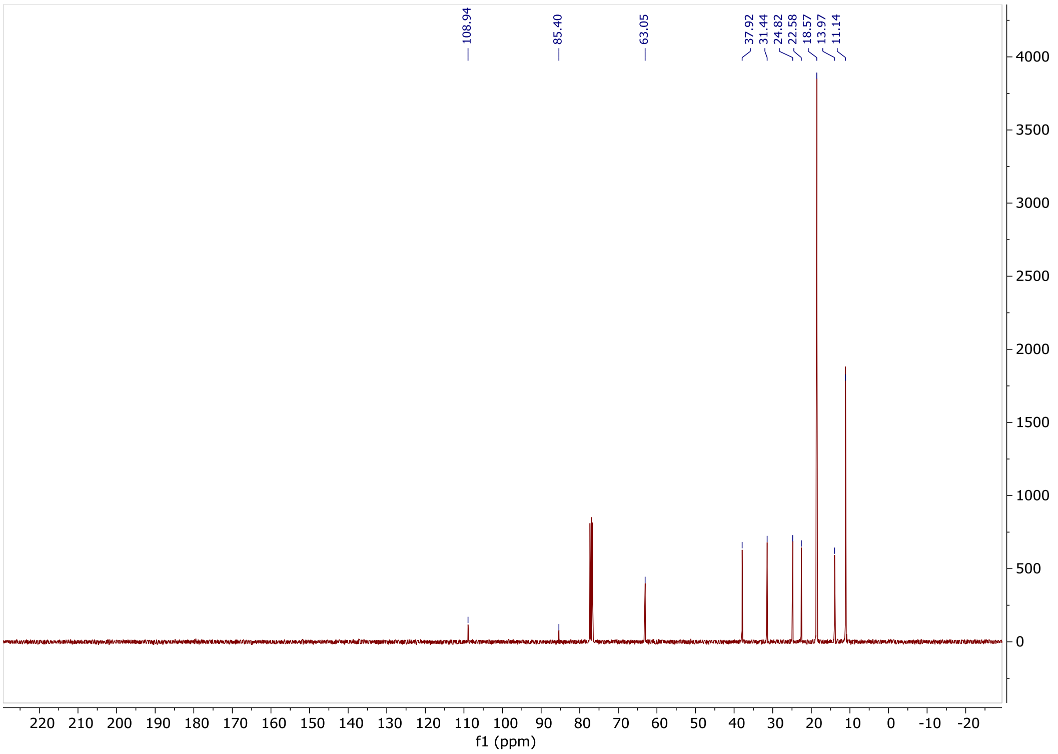

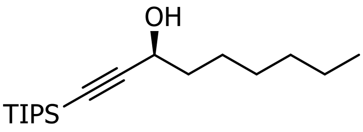

^{1}H NMR (400 MHz, $CDCl_3$) of **3b**

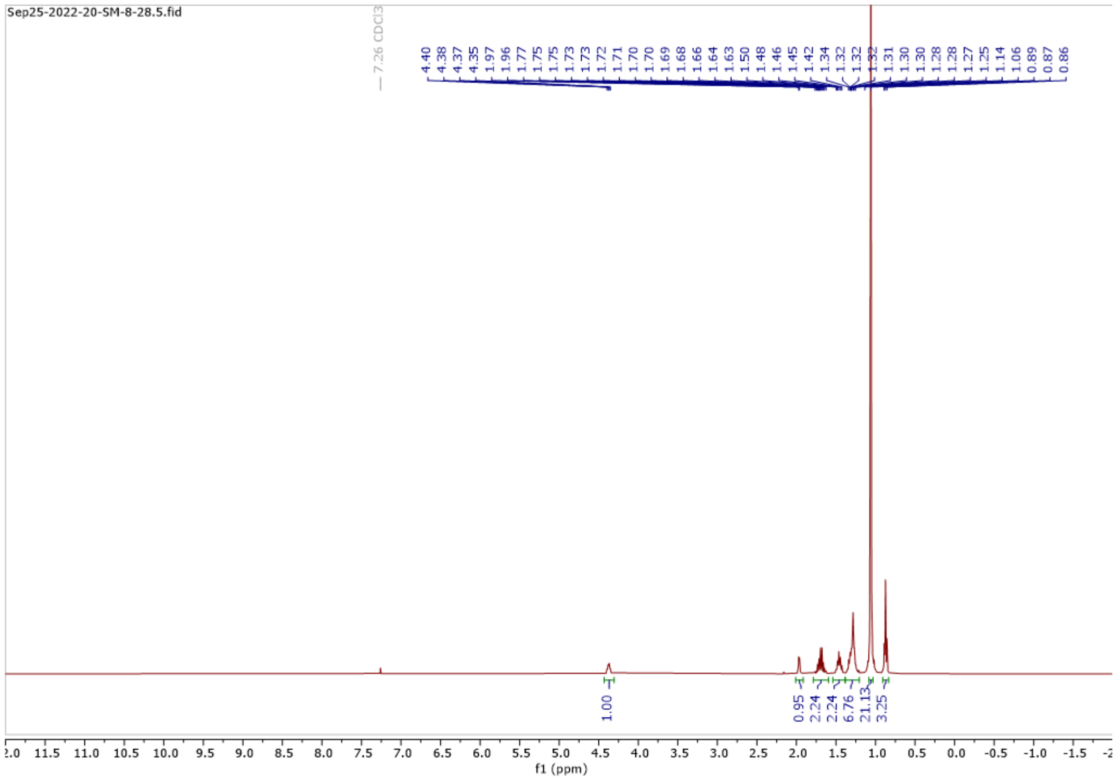

^{13}C NMR (101MHz, $CDCl_3$) of **3b**

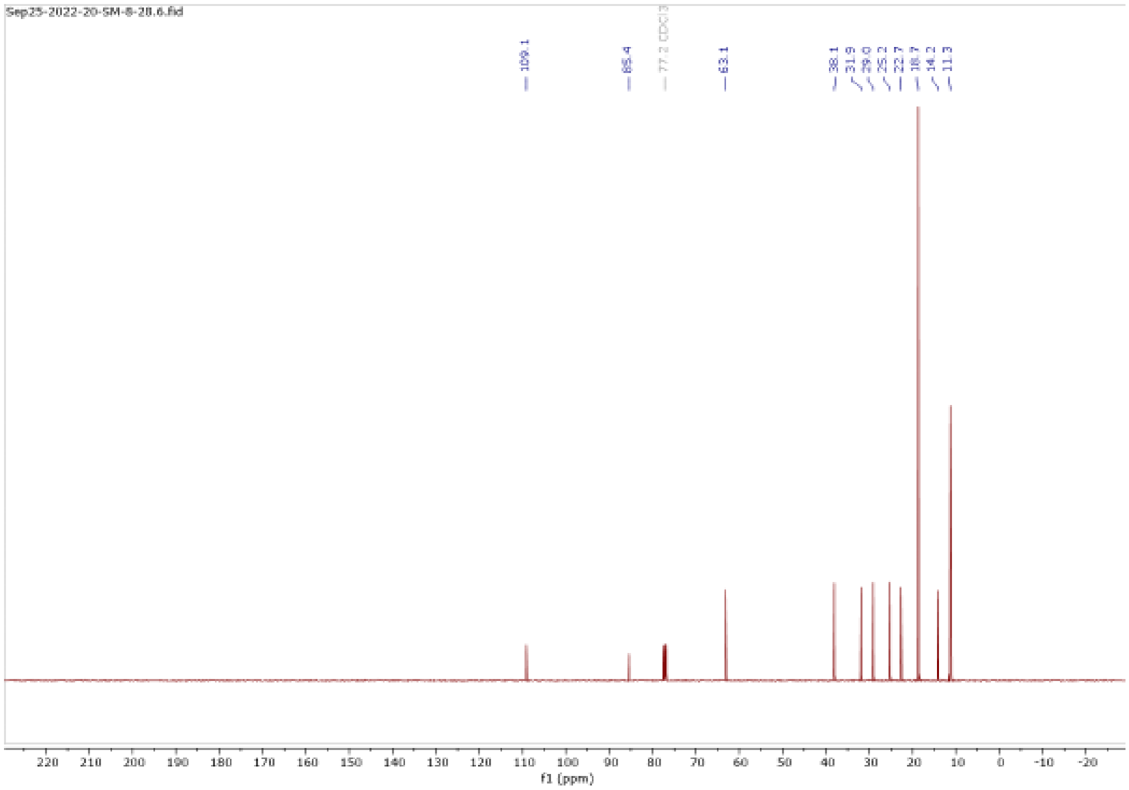

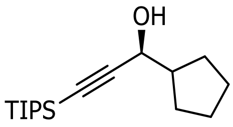

^{1}H NMR (500MHz, $CDCl_3$) of **3c**

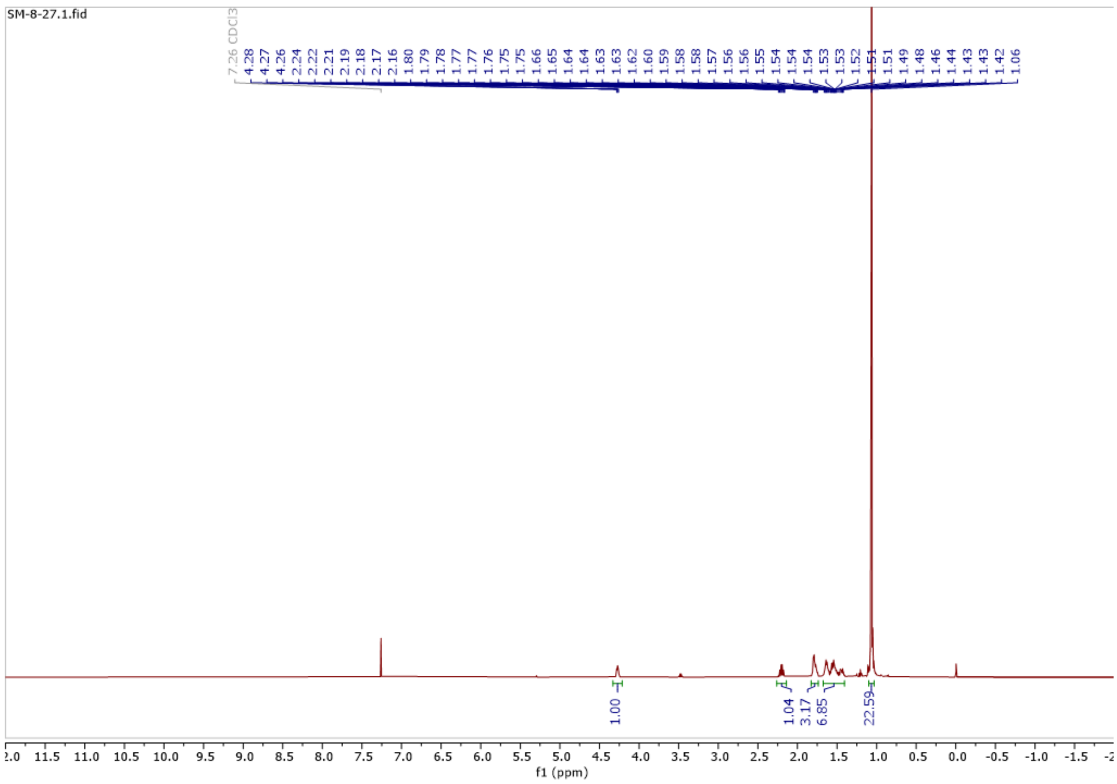

^{13}C NMR (126MHz, $CDCl_3$) of **3c**

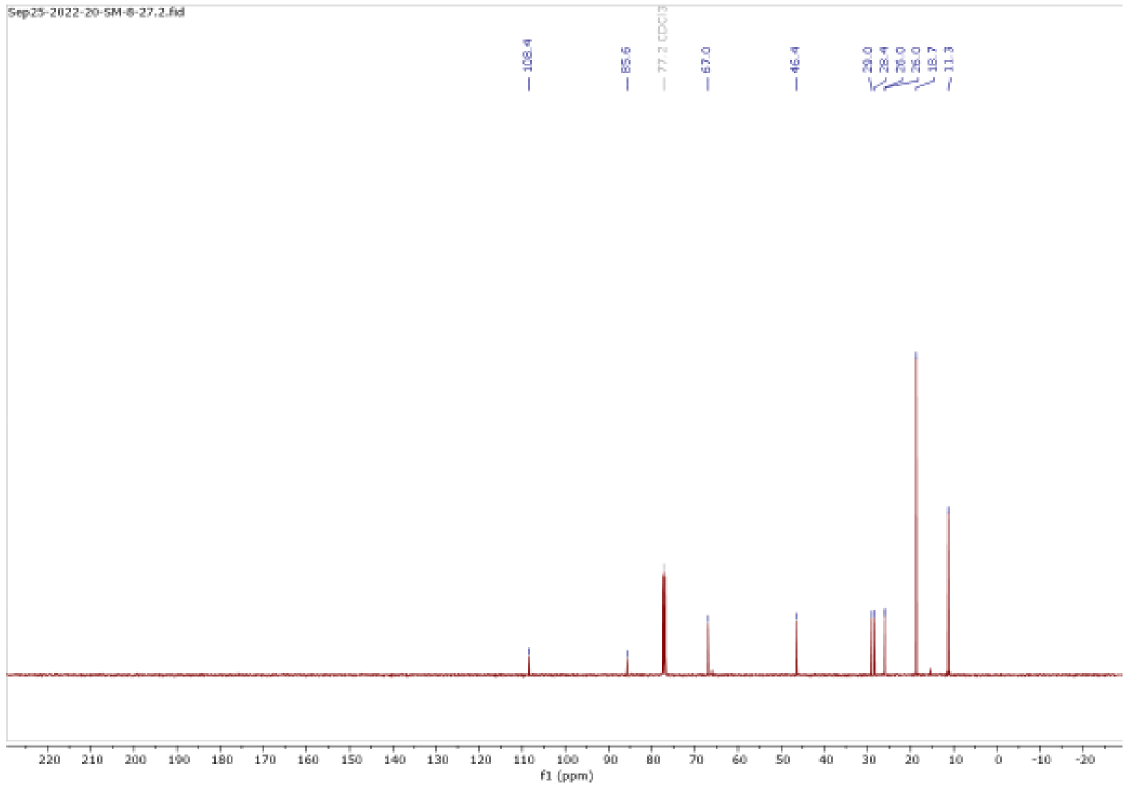

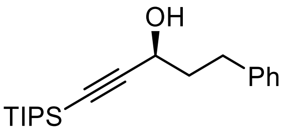

TIPS

^{1}H NMR (400 MHz, $CDCl_3$) of **3d**

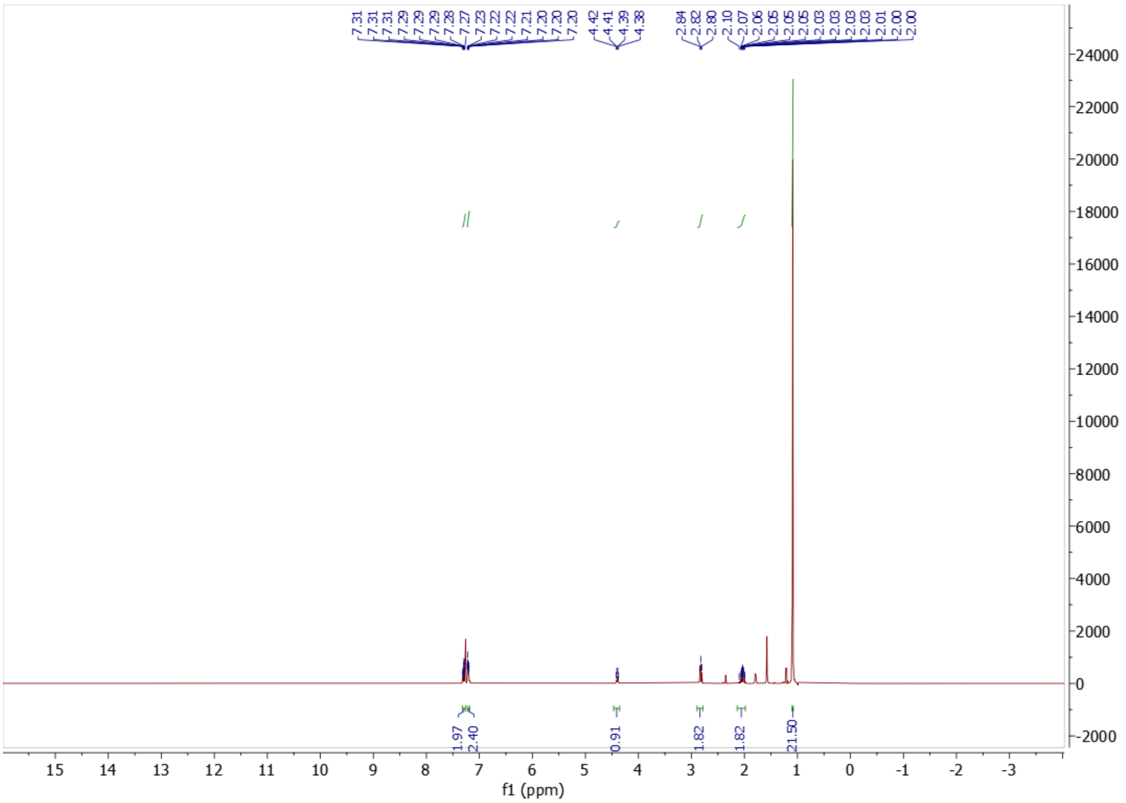

^{13}C NMR (101MHz, $CDCl_3$) of **3d**

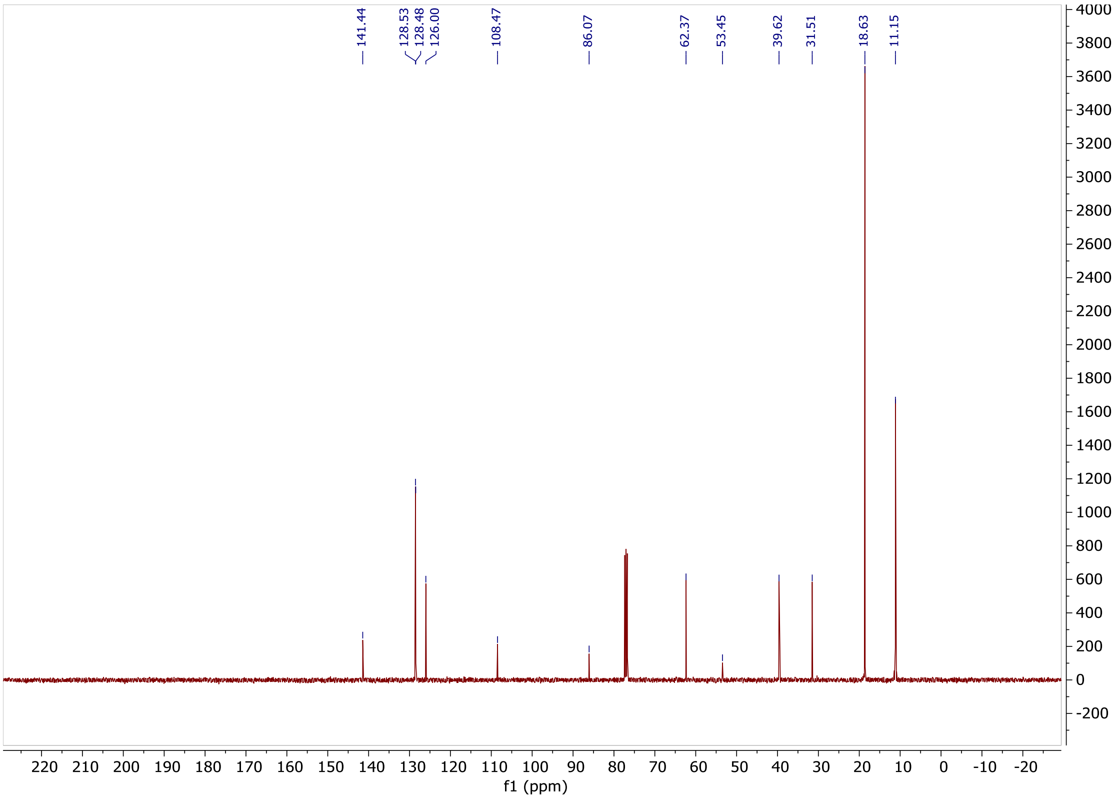

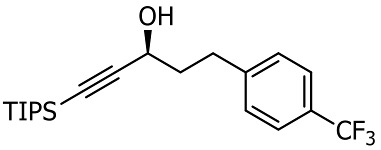

^{1}H NMR (400 MHz, $CDCl_3$) of **3e**

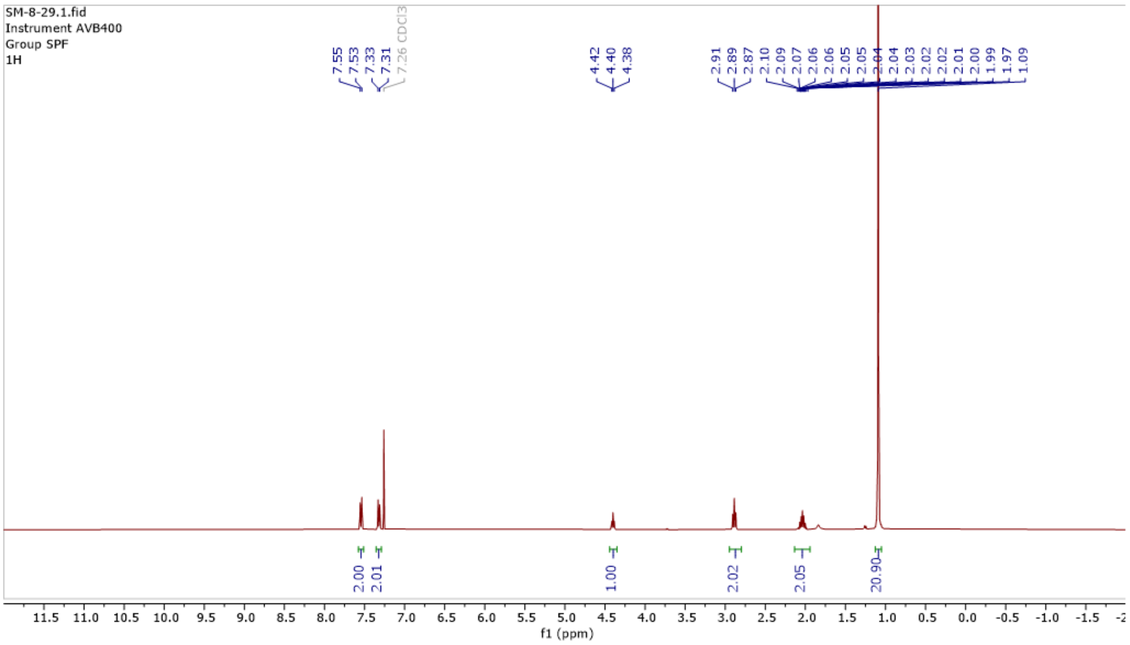

^{13}C NMR (101MHz, $CDCl_3$) of **3e**

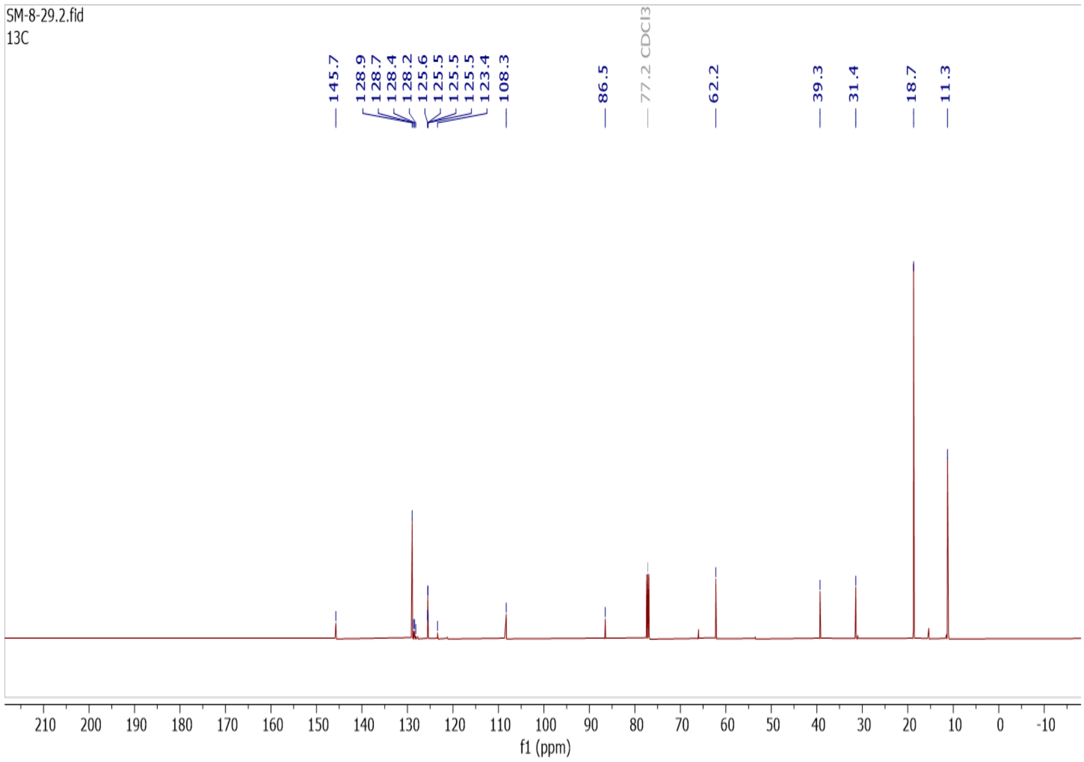

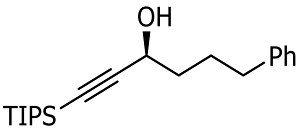

^{1}H NMR (400 MHz, $CDCl_3$) of **3f**

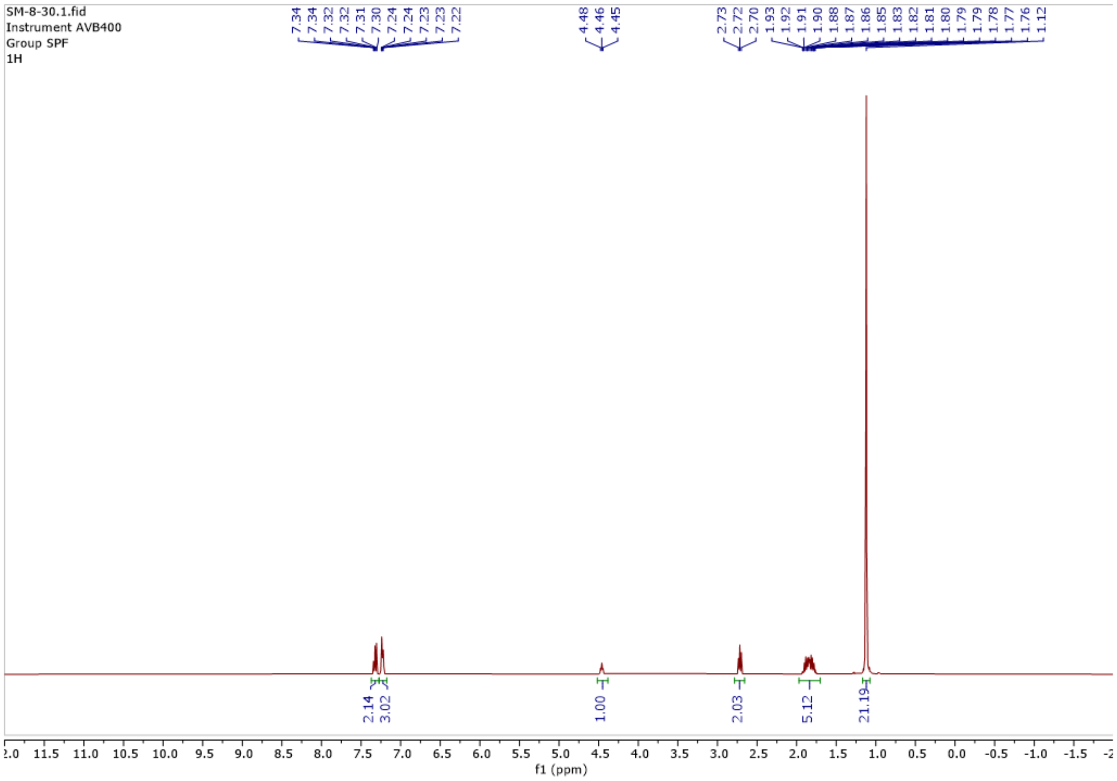

^{13}C NMR (101MHz, $CDCl_3$) of **3f**

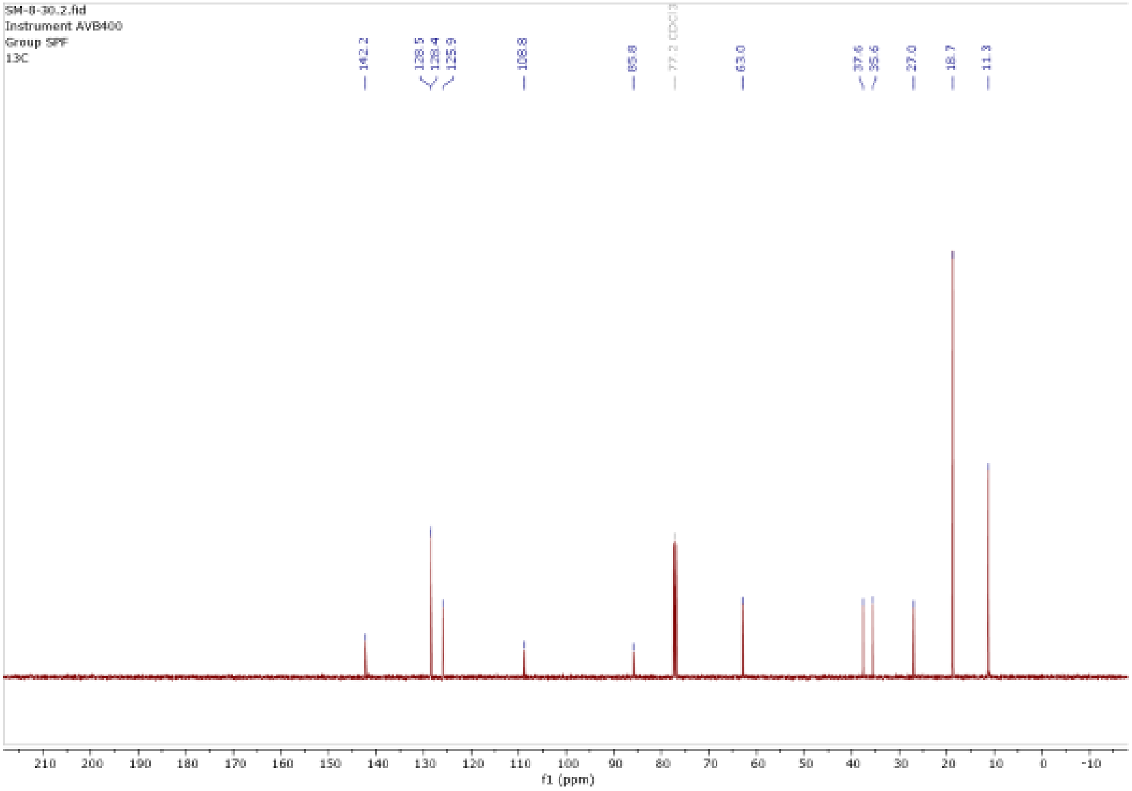

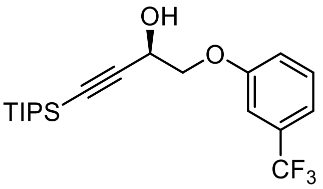

^{1}H NMR (400 MHz, $CDCl_3$) of **3g**

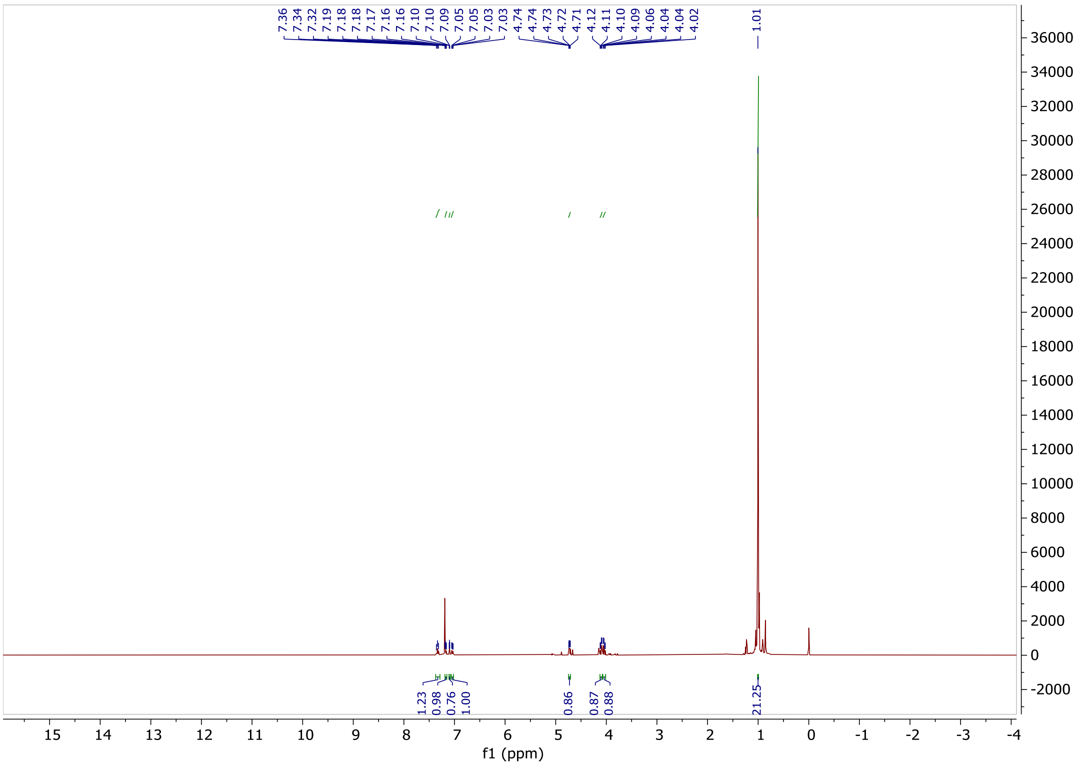

^{13}C NMR (101MHz, $CDCl_3$) of **3g**

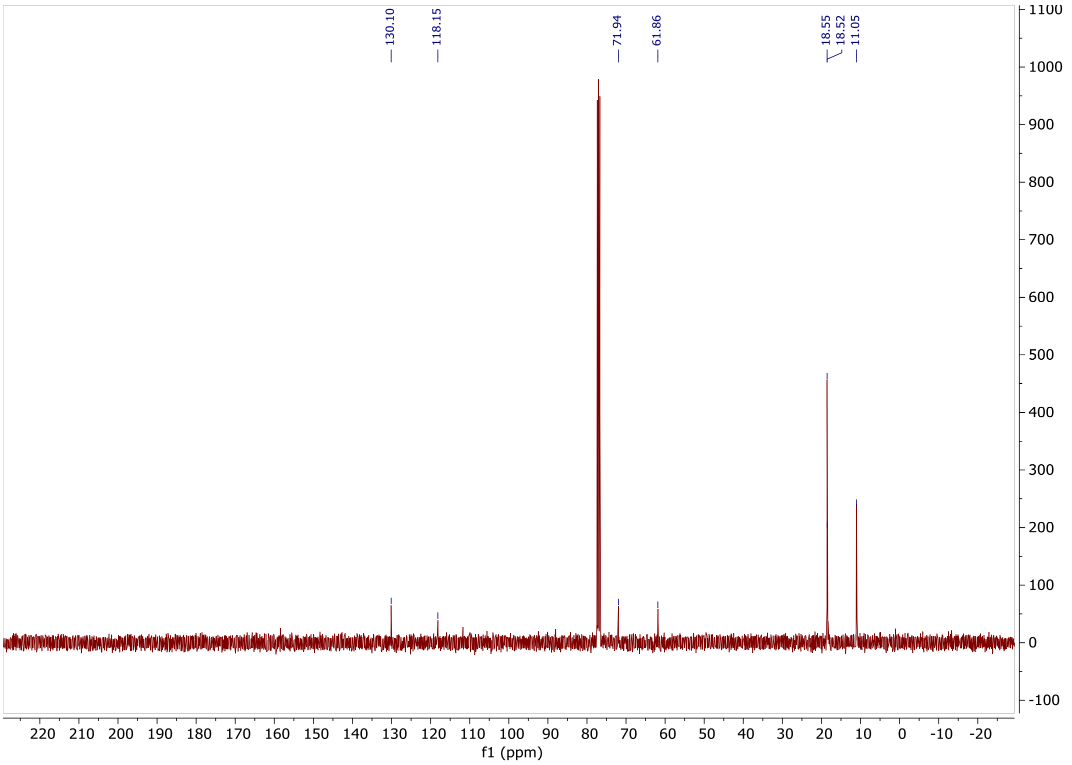

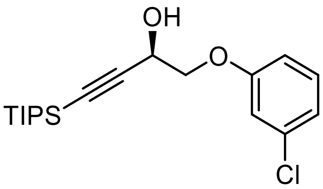

^{1}H NMR (400 MHz, $CDCl_3$) of **3h**

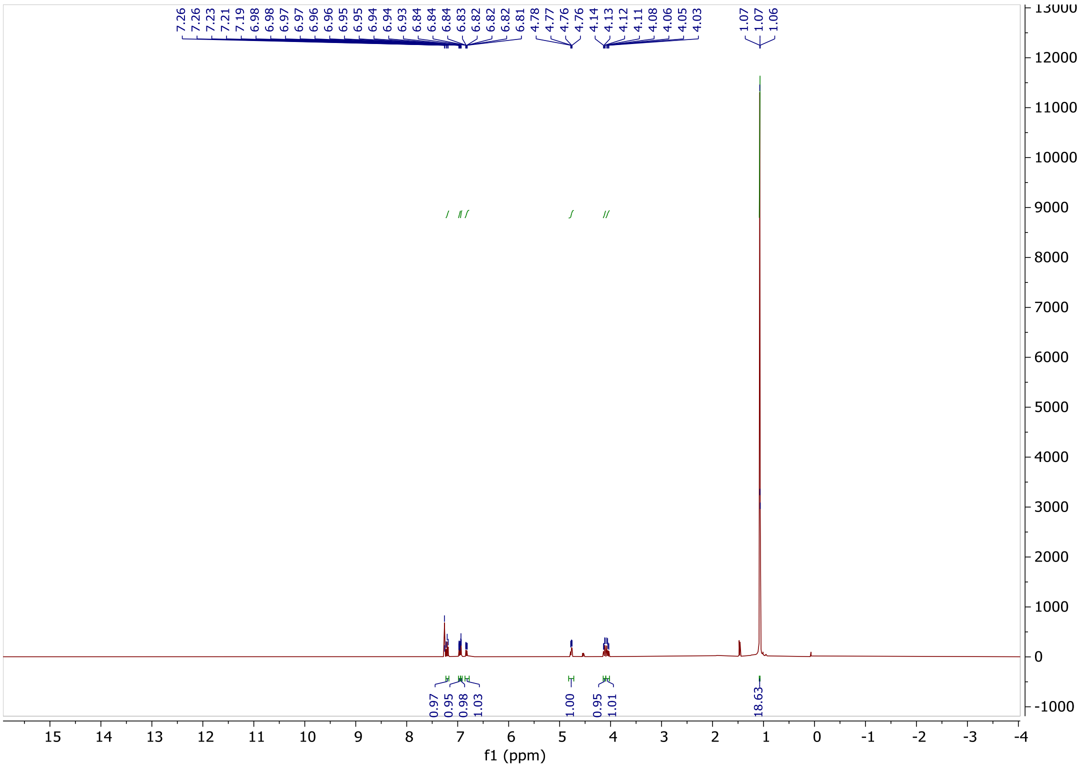

^{13}C NMR (101MHz, $CDCl_3$) of **3h**

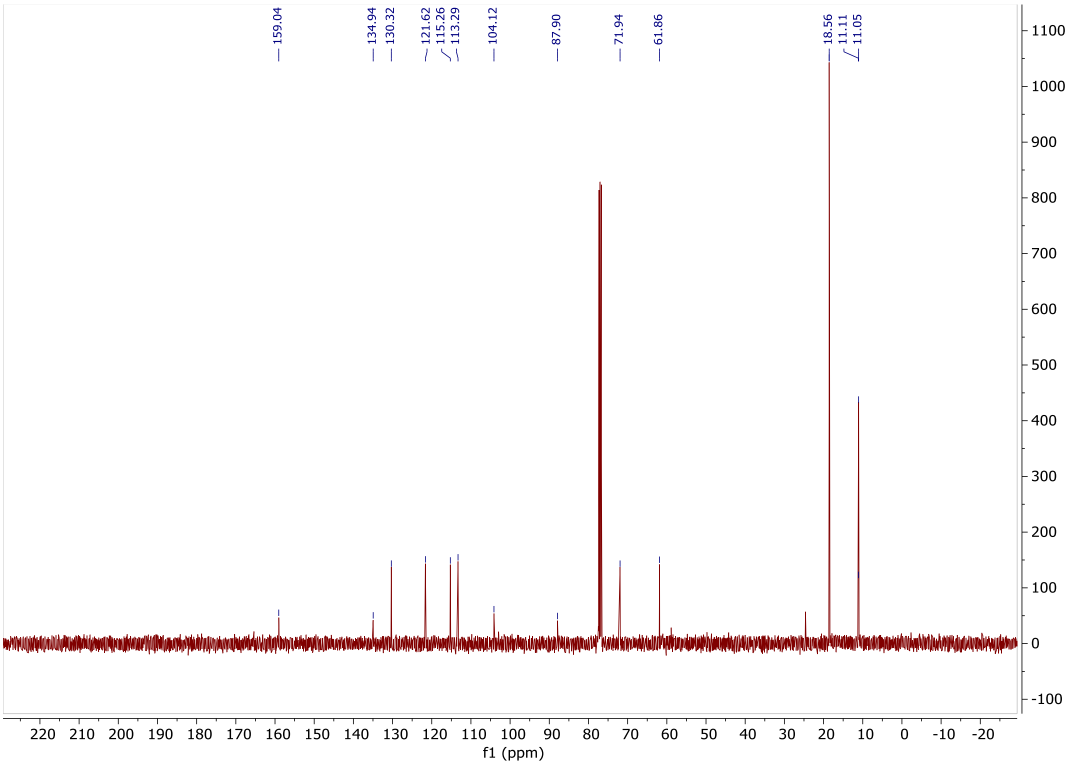

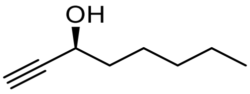

^{1}H NMR (400 MHz, $CDCl_3$) of **S2a**

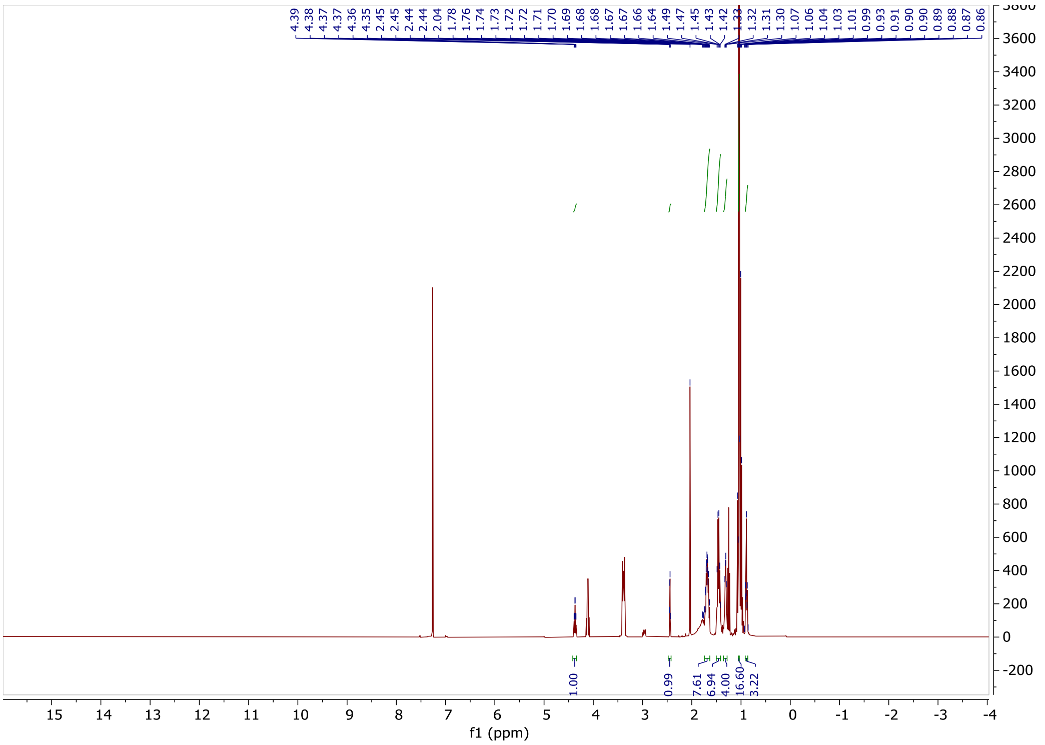

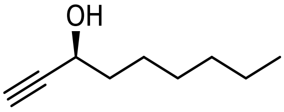

^{1}H NMR (400 MHz, $CDCl_3$) of **S2b**

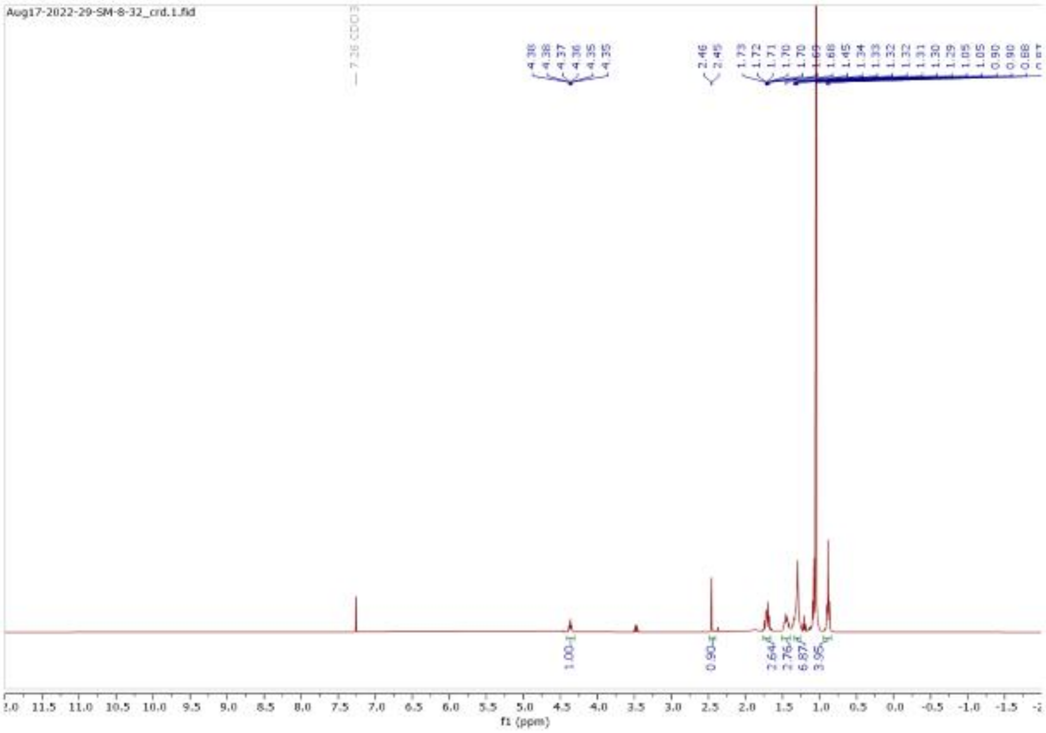

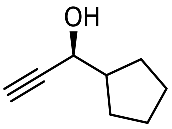

^{1}H NMR (400 MHz, $CDCl_3$) of **S2c**

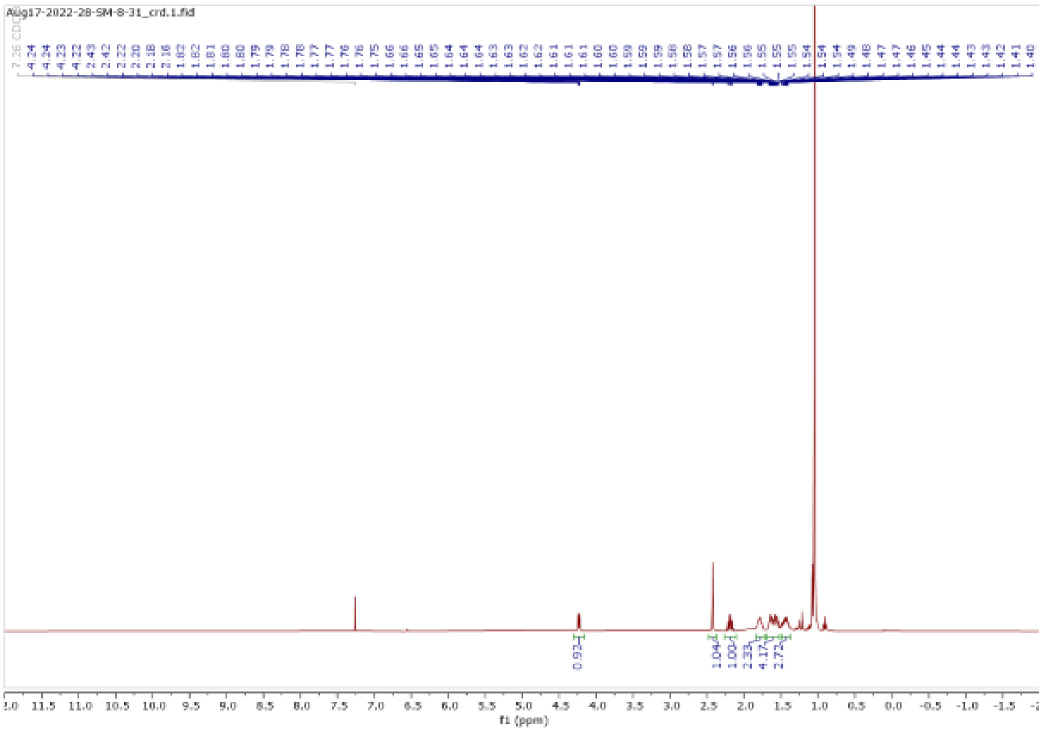

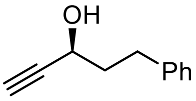

^{1}H NMR (400 MHz, $CDCl_3$) of **S2d**

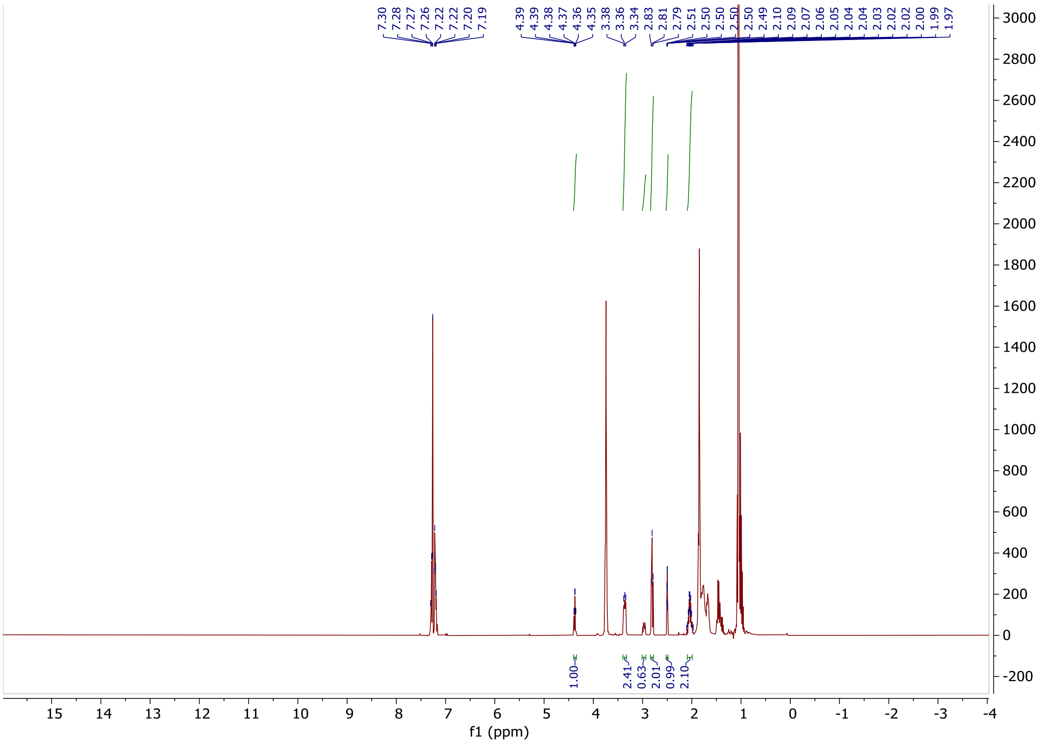

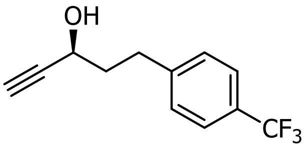

^{1}H NMR (400 MHz, $CDCl_3$) of **S2e**

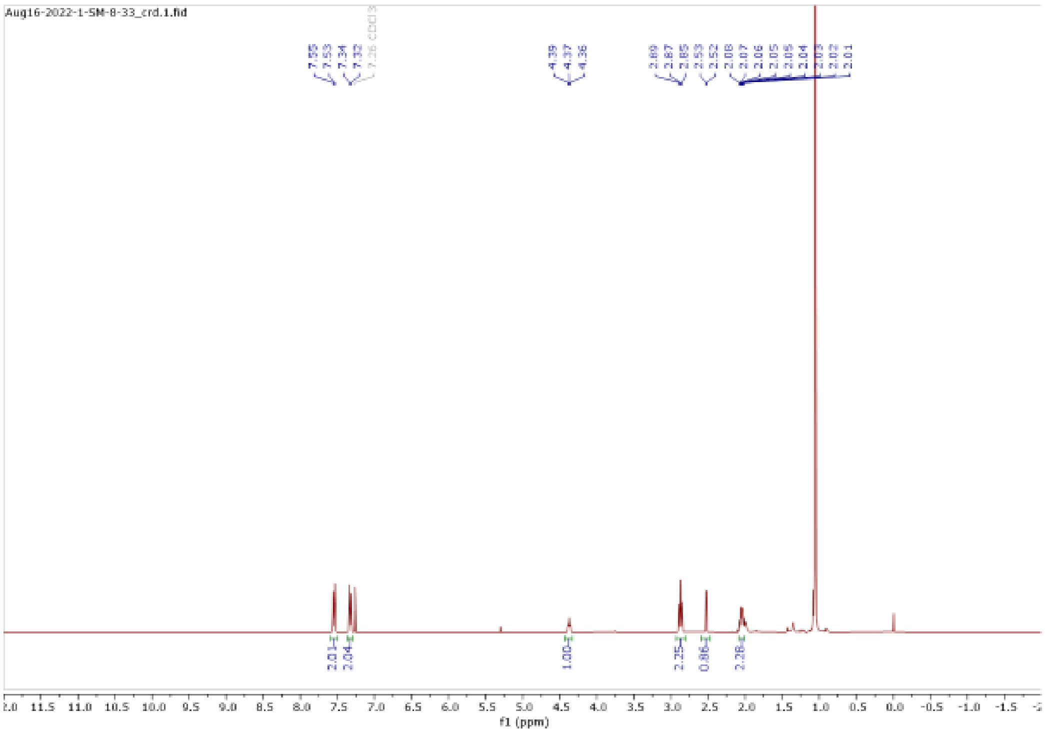

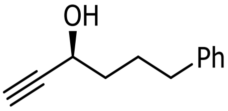

^{1}H NMR (400 MHz, $CDCl_3$) of **S2f**

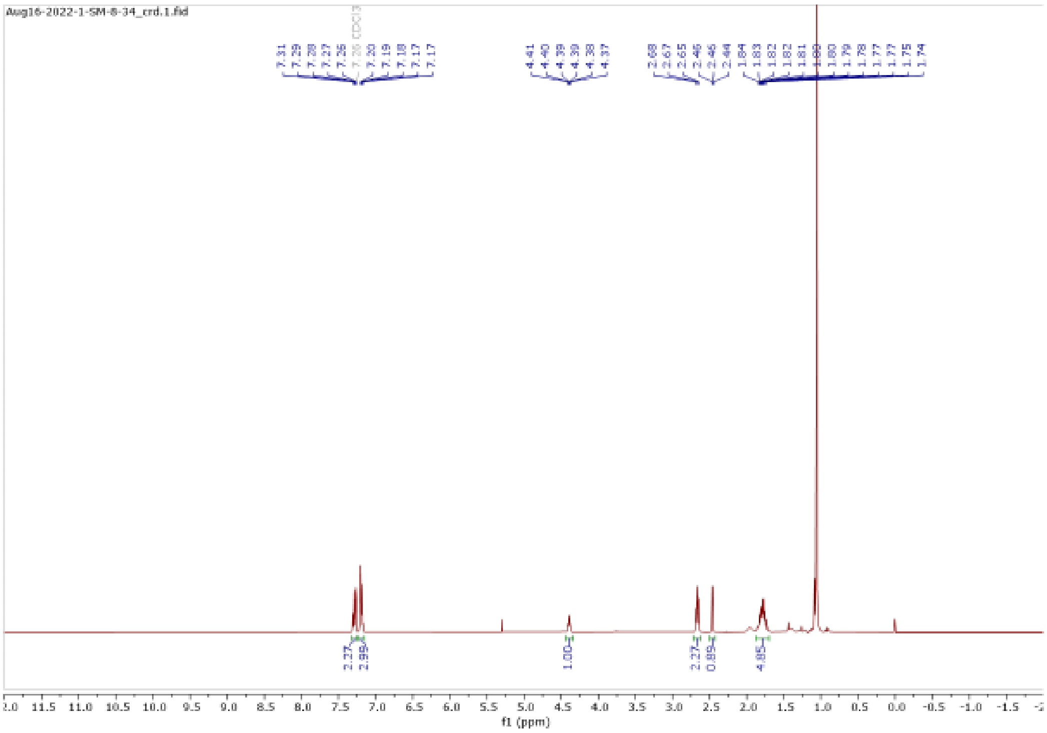

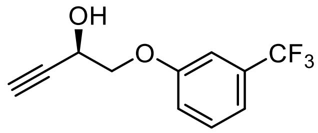

^{1}H NMR (400 MHz, $CDCl_3$) of **S2g**

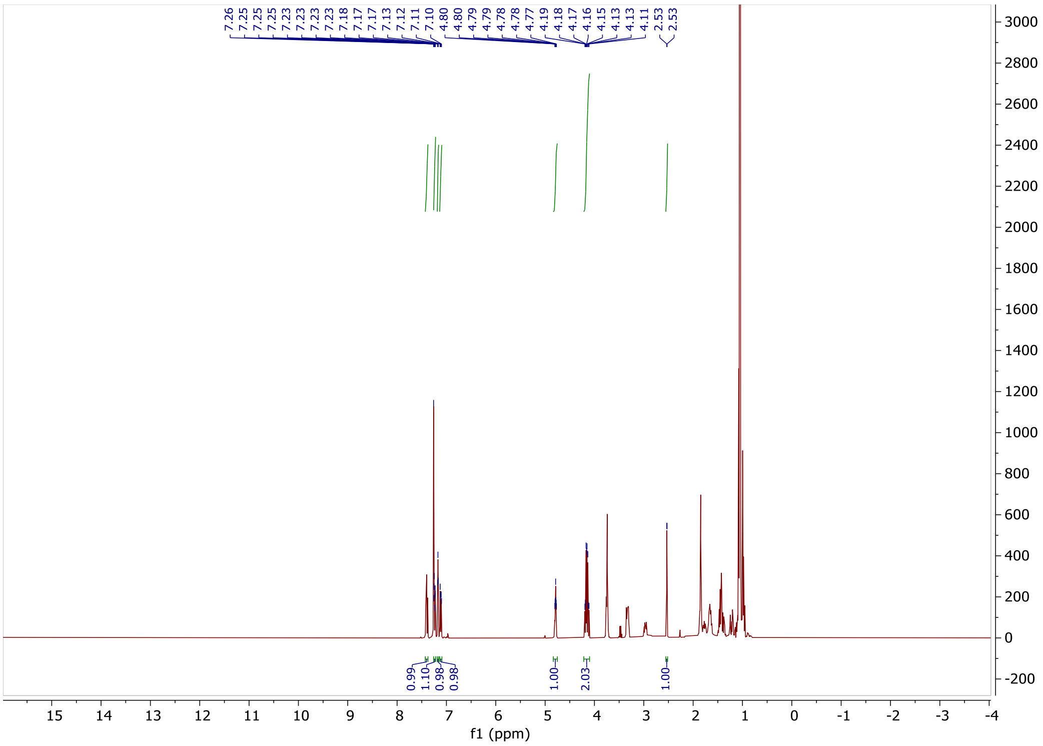

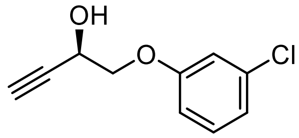

^{1}H NMR (400 MHz, $CDCl_3$) of **S2h**

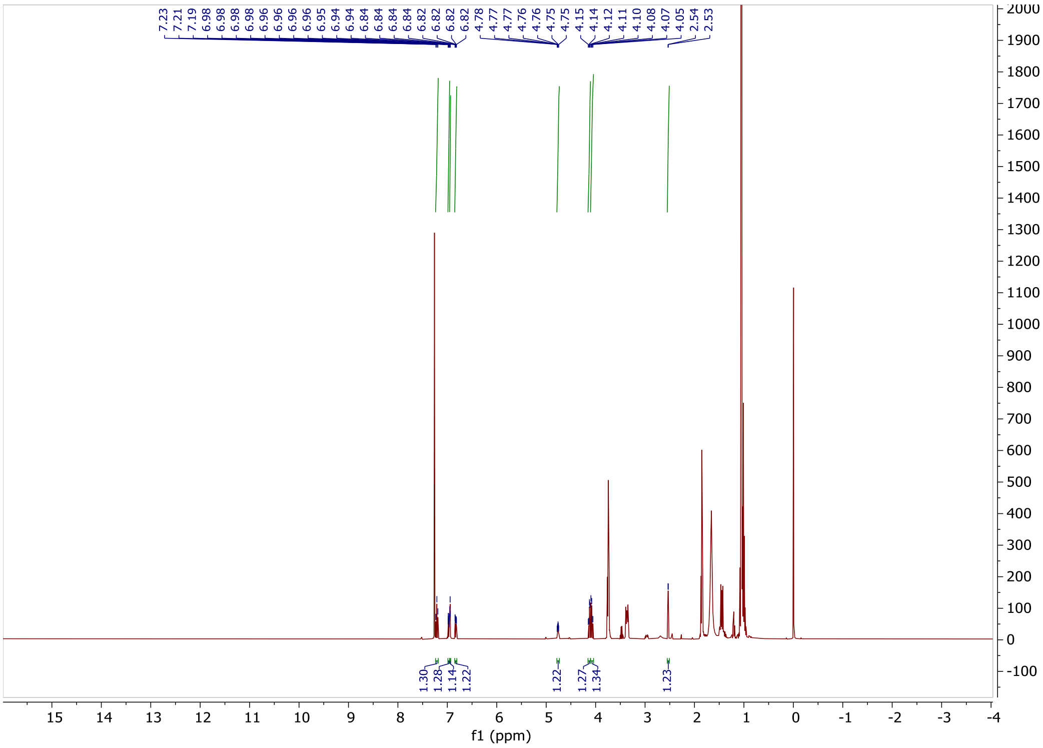

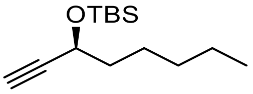

^{1}H NMR (400 MHz, $CDCl_3$) of **S3a**

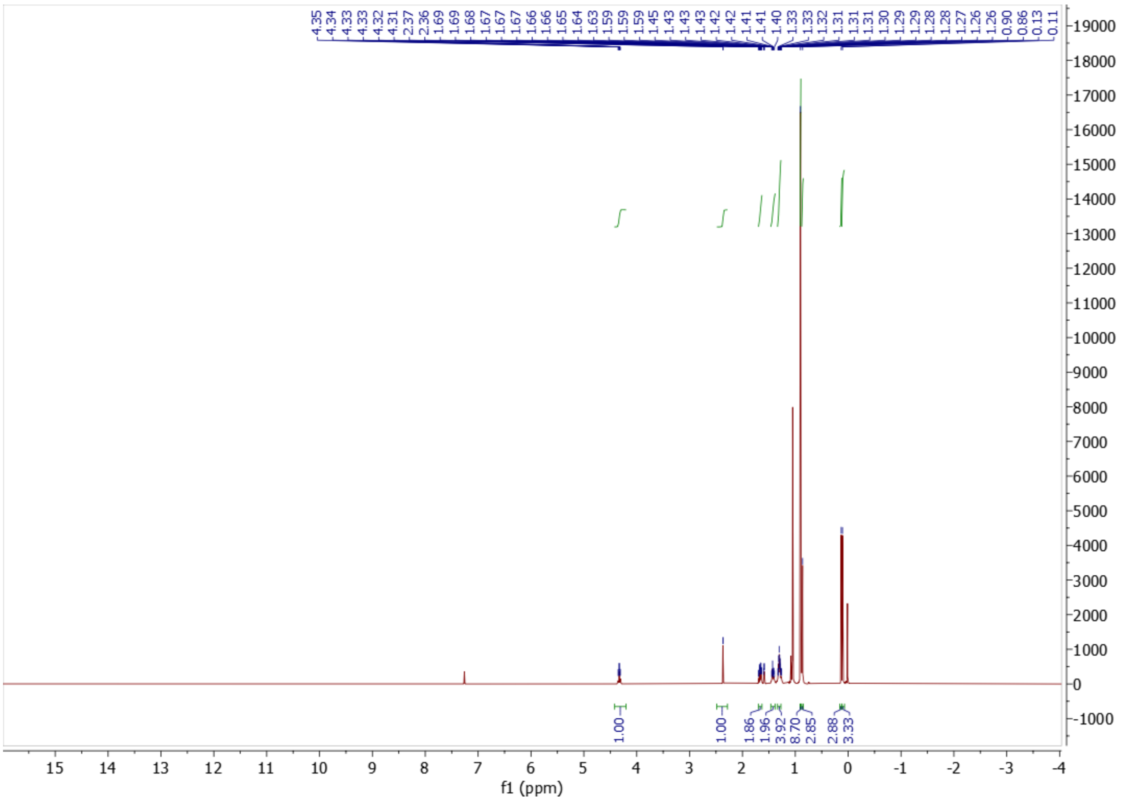

^{13}C NMR (101MHz, $CDCl_3$) of **S3a**

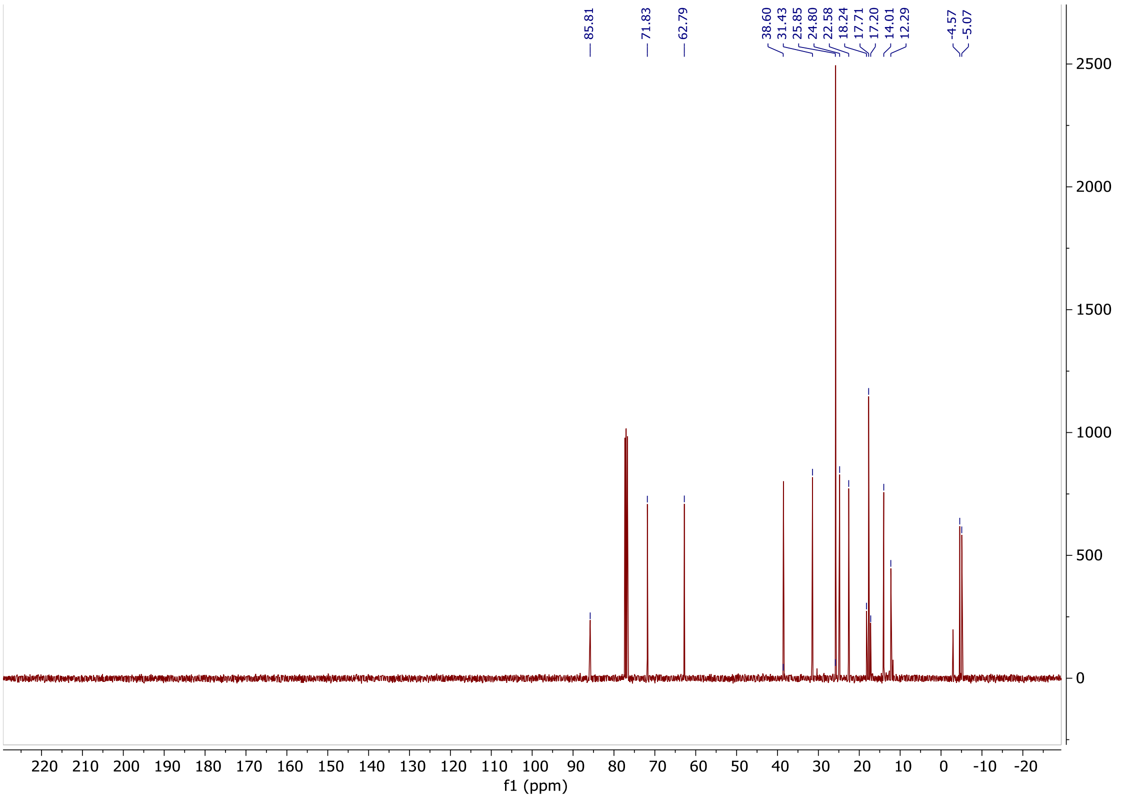

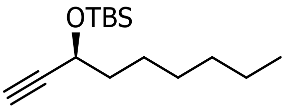

^{1}H NMR (500MHz, $CDCl_3$) of **S3b**

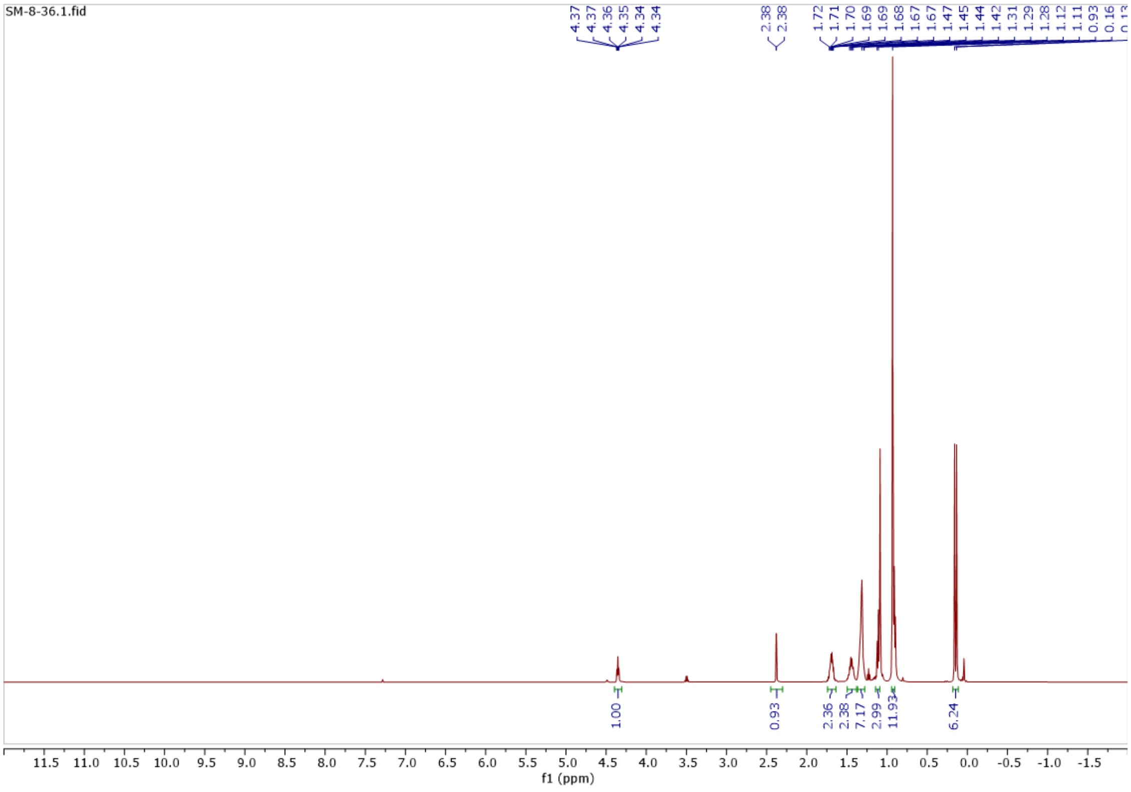

^{13}C NMR (126MHz, $CDCl_3$) of **S3b**

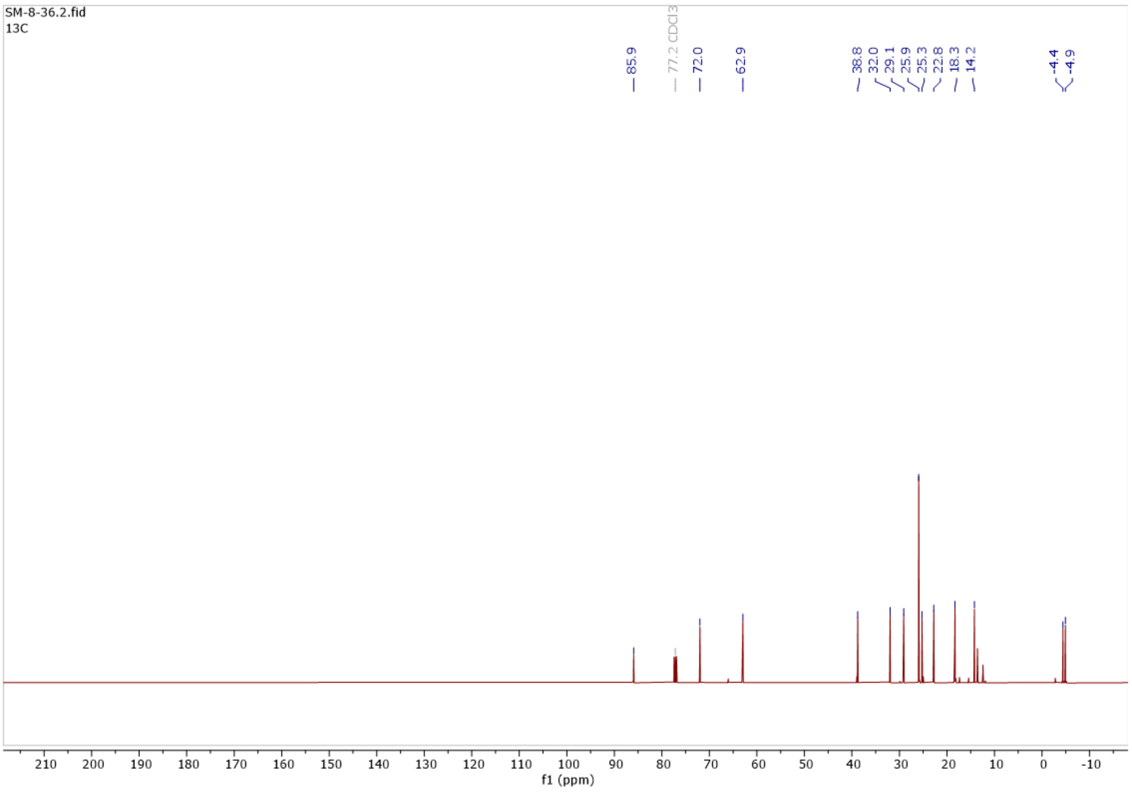

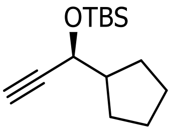

^{1}H NMR (500MHz, $CDCl_3$) of **S3c**

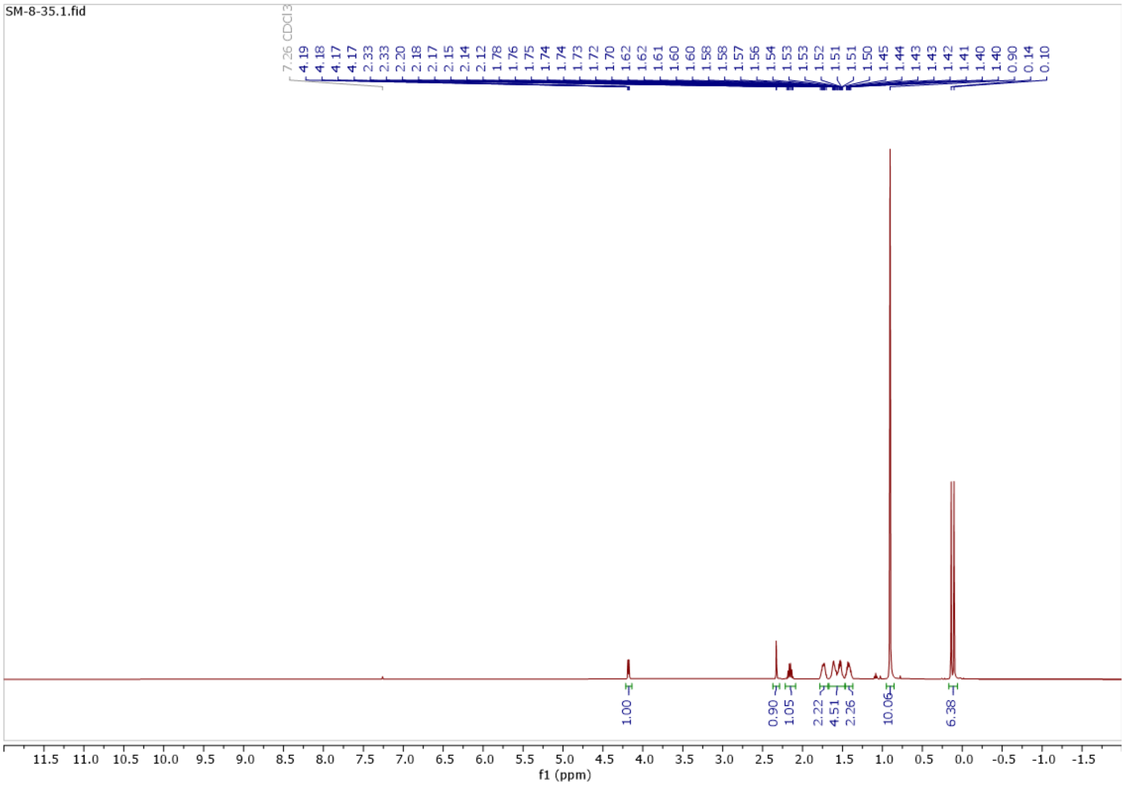

^{13}C NMR (126MHz, $CDCl_3$) of **S3c**

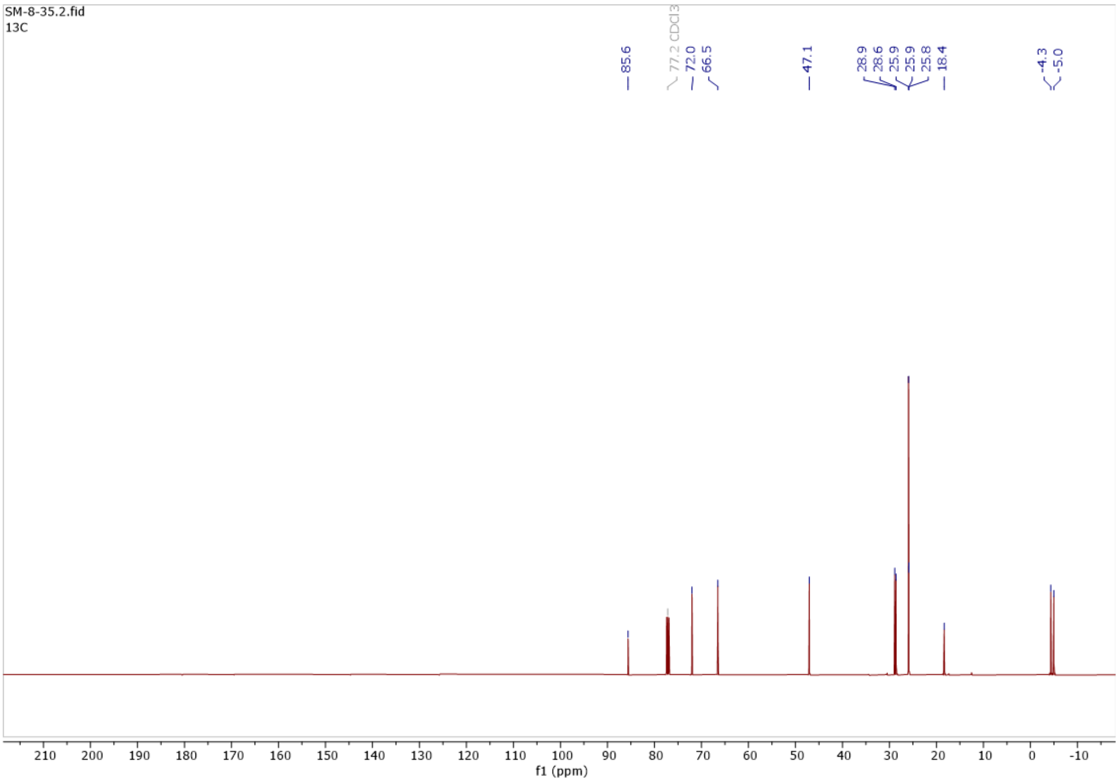

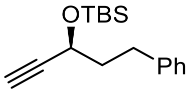

^{1}H NMR (400 MHz, $CDCl_3$) of **S3d**

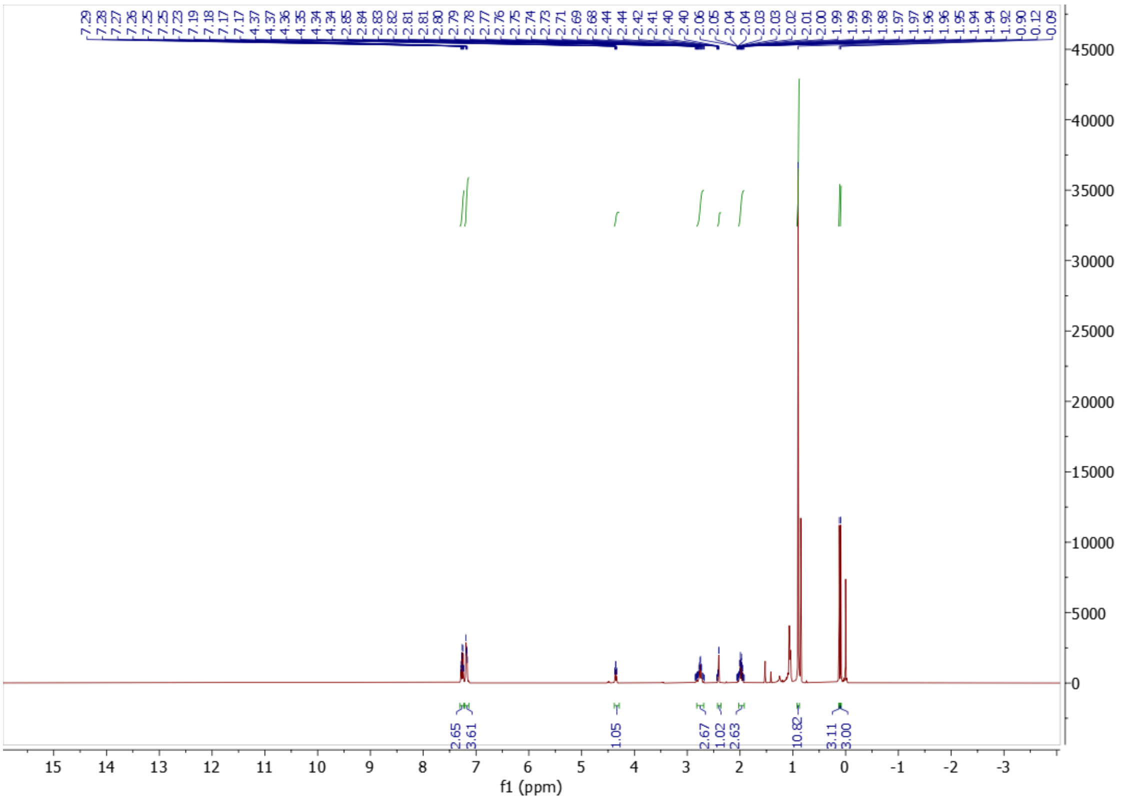

^{13}C NMR (101MHz, $CDCl_3$) of **S3d**

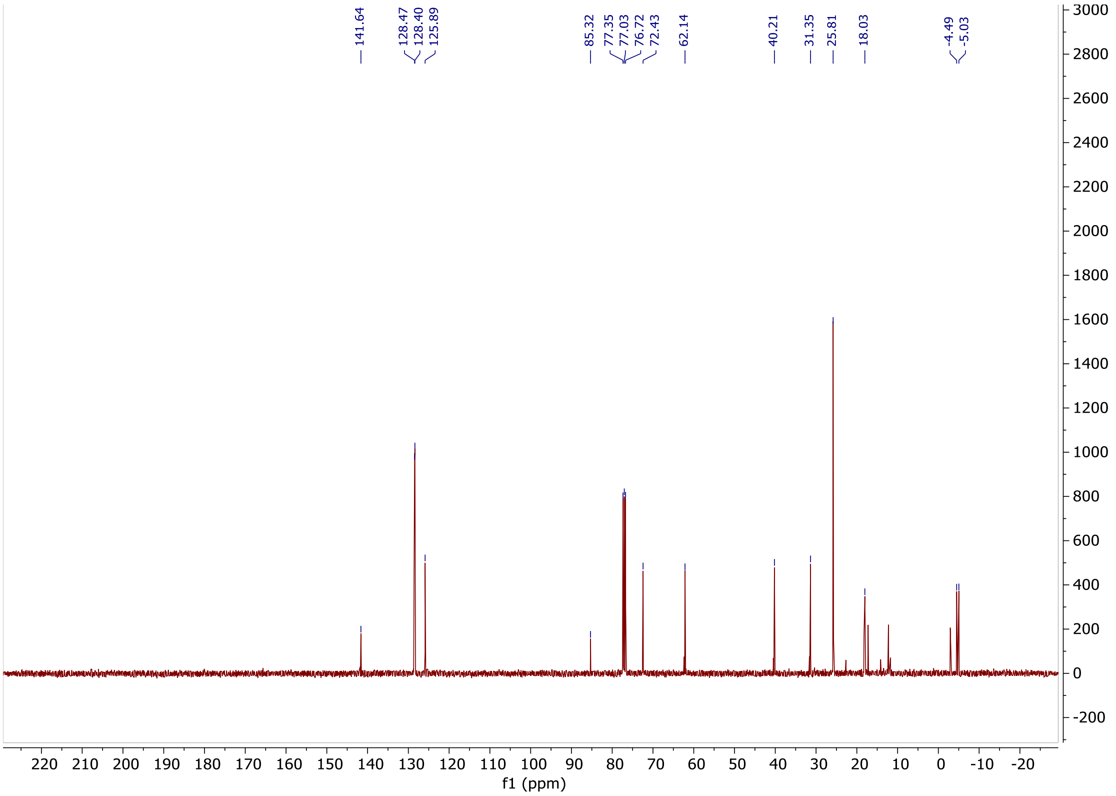

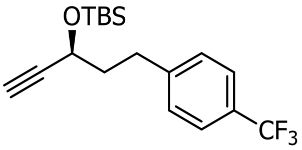

^{1}H NMR (500 MHz, $CDCl_3$) of **S3e**

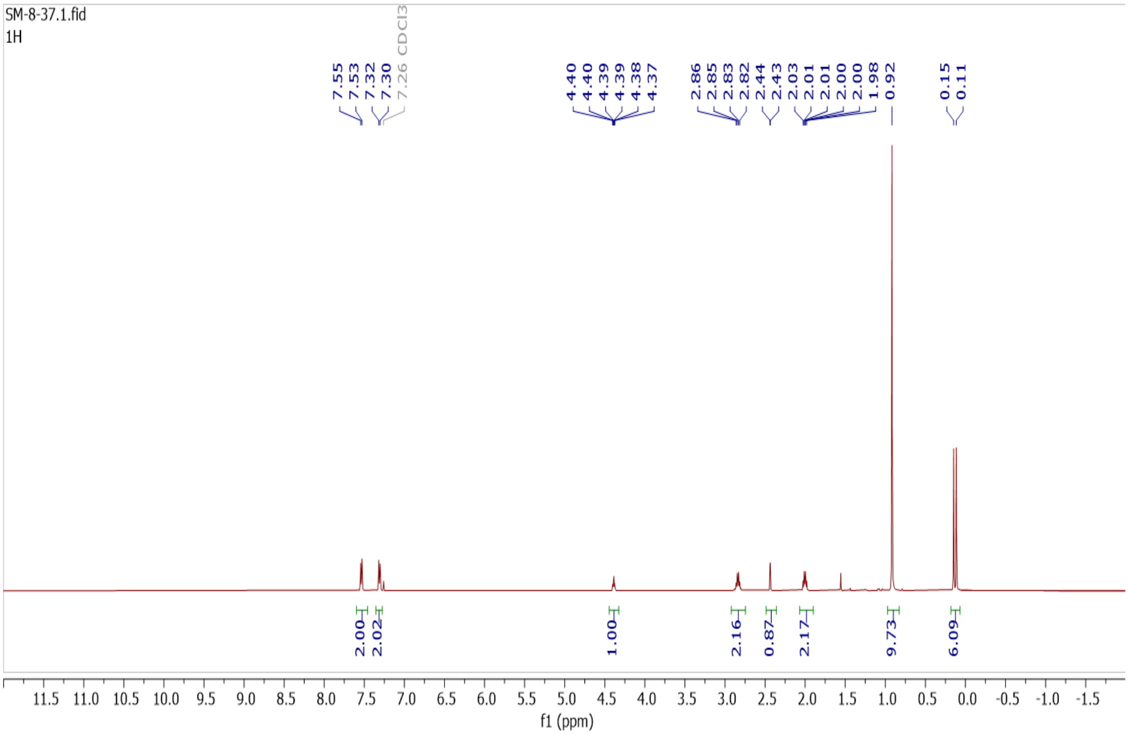

^{13}C NMR (126 MHz, $CDCl_3$) of **S3e**

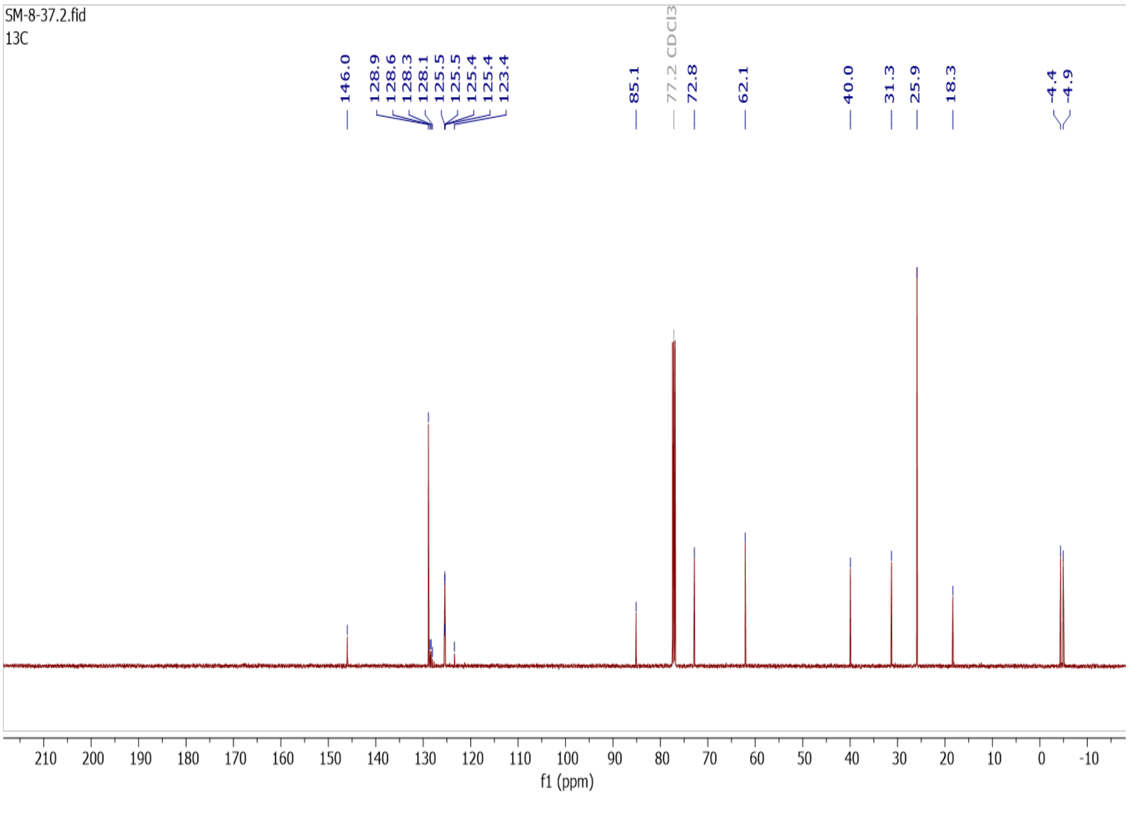

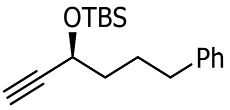

^{1}H NMR (500 MHz, $CDCl_3$) of **S3f**

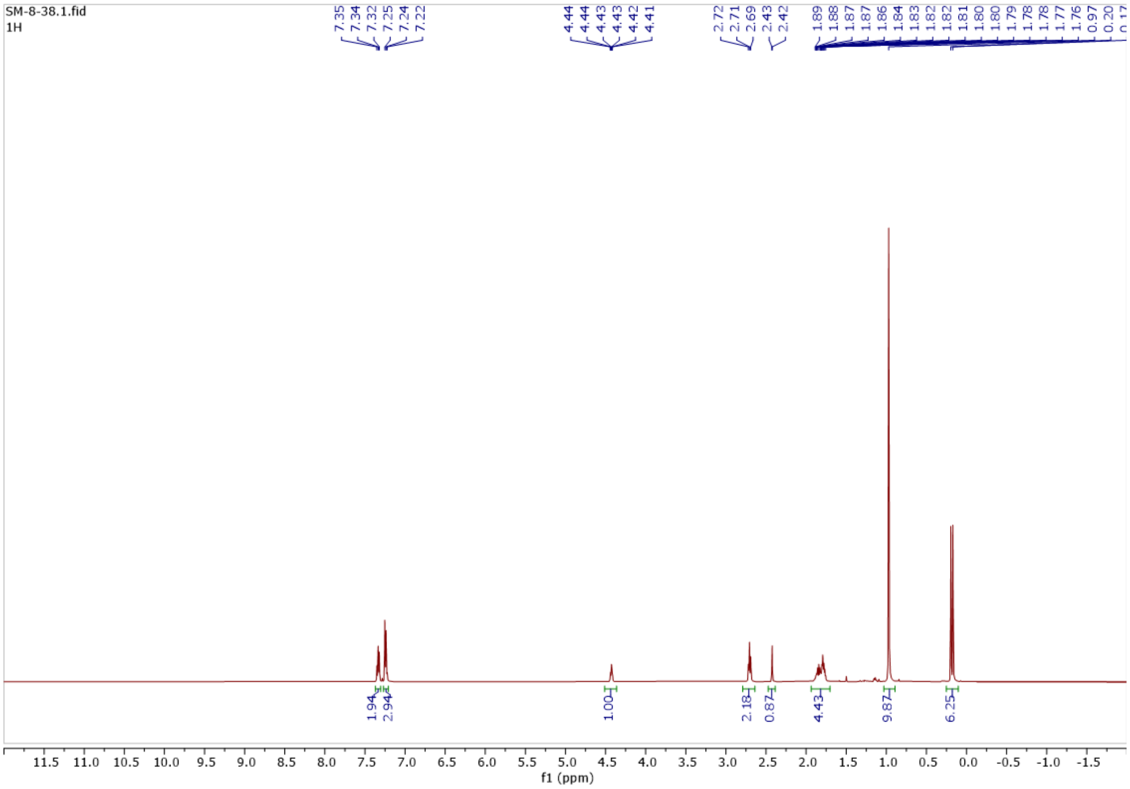

^{13}C NMR (126MHz, $CDCl_3$) of **S3f**

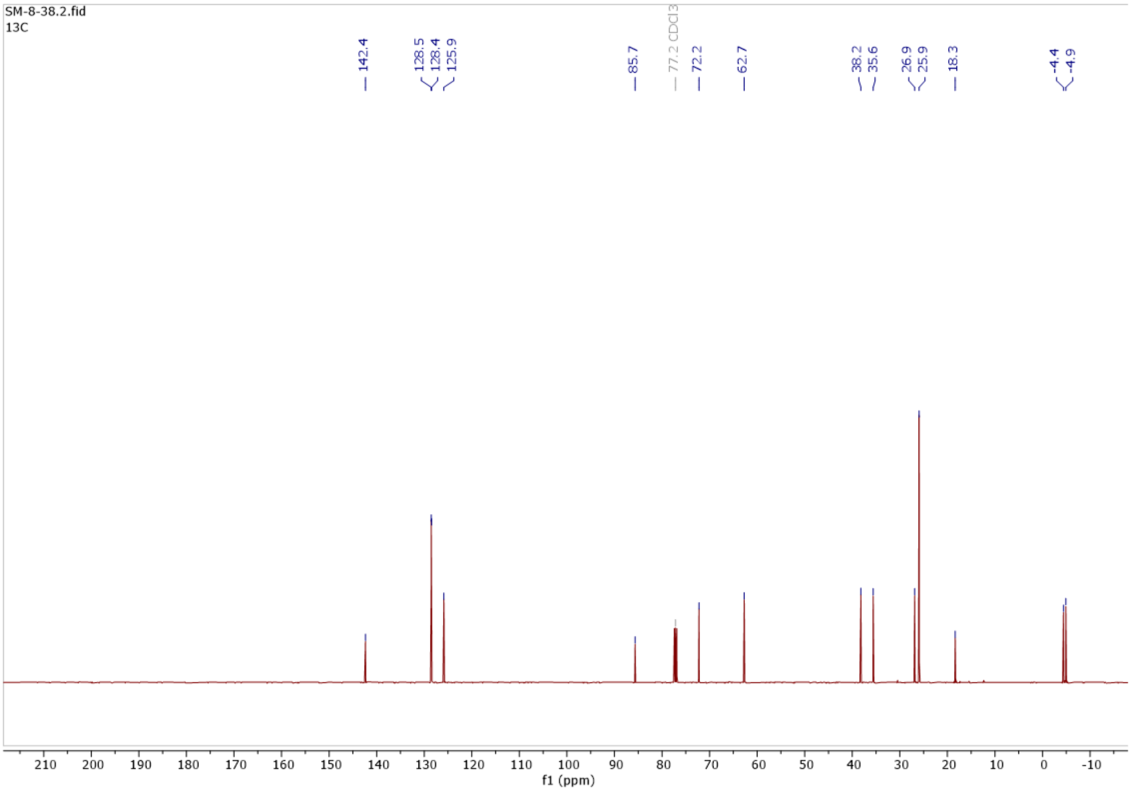

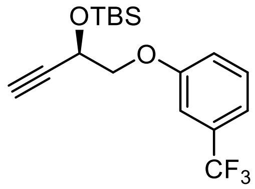

^{1}H NMR (400 MHz, $CDCl_3$) of **S3g**

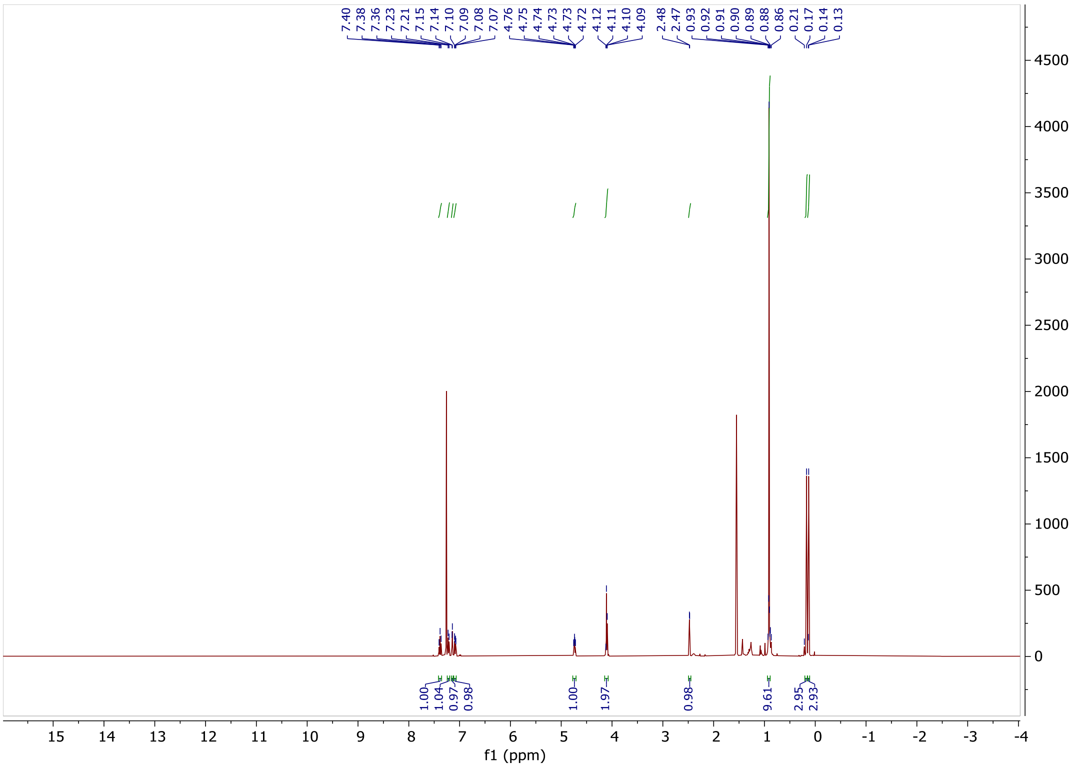

^{13}C NMR (101MHz, $CDCl_3$) of **S3g**

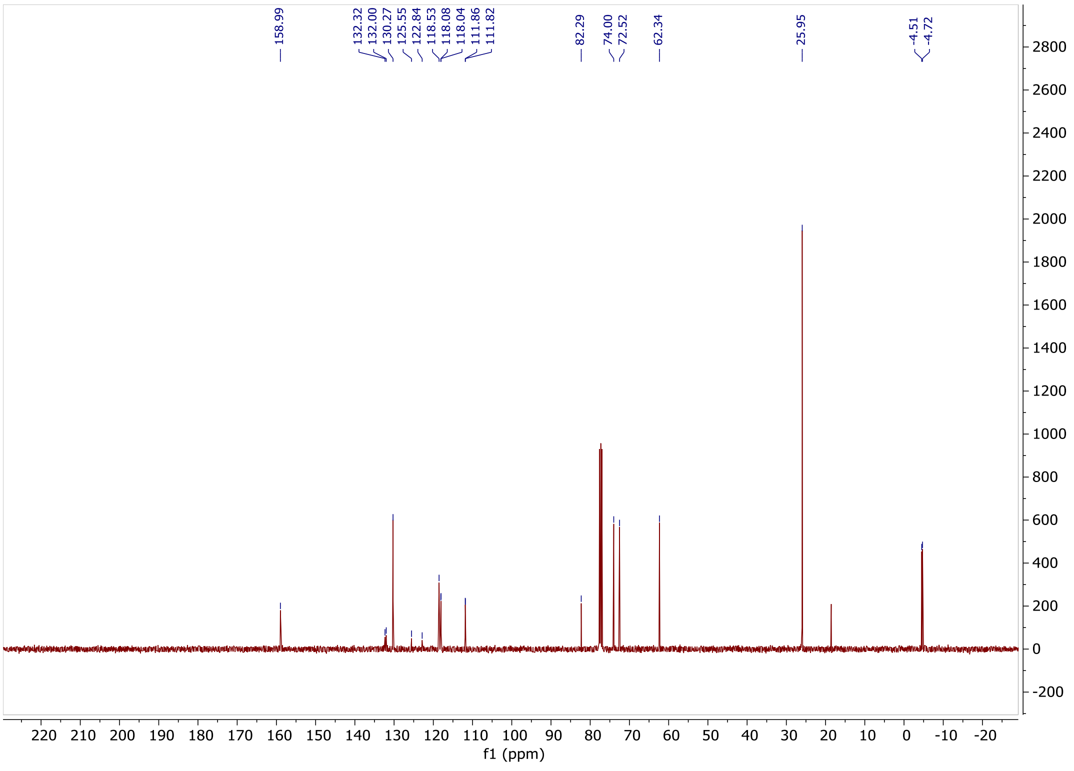

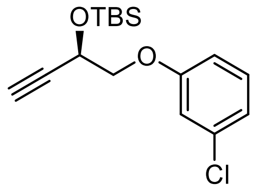

^{1}H NMR (400 MHz, $CDCl_3$) of **S3h**

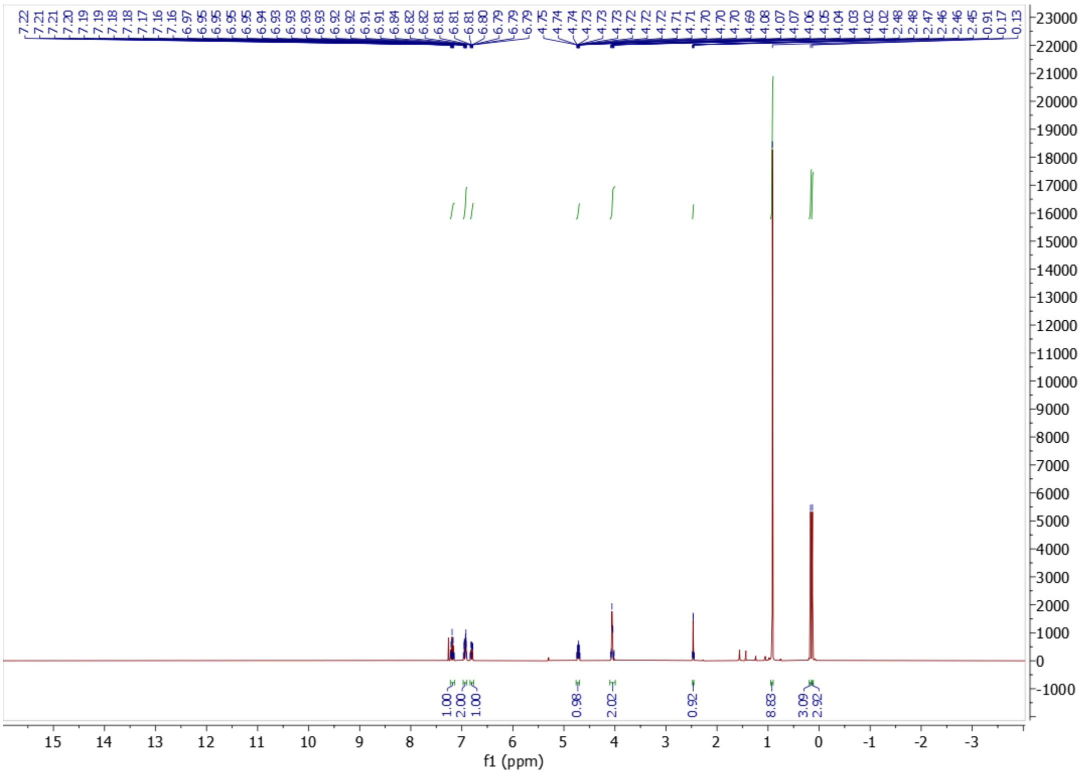

^{13}C NMR (101MHz, $CDCl_3$) of **S3h**

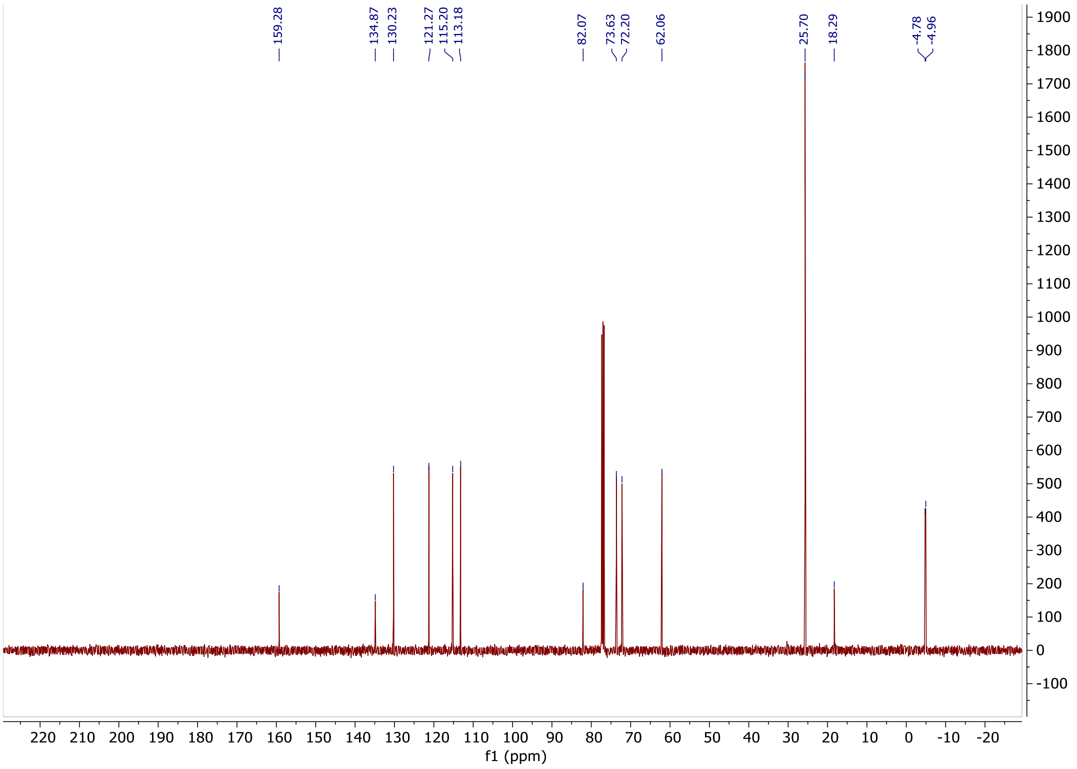

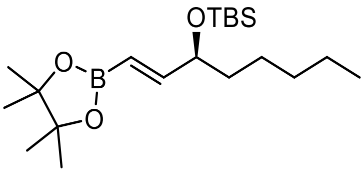

^{1}H NMR (400 MHz, $CDCl_3$) of **4a**

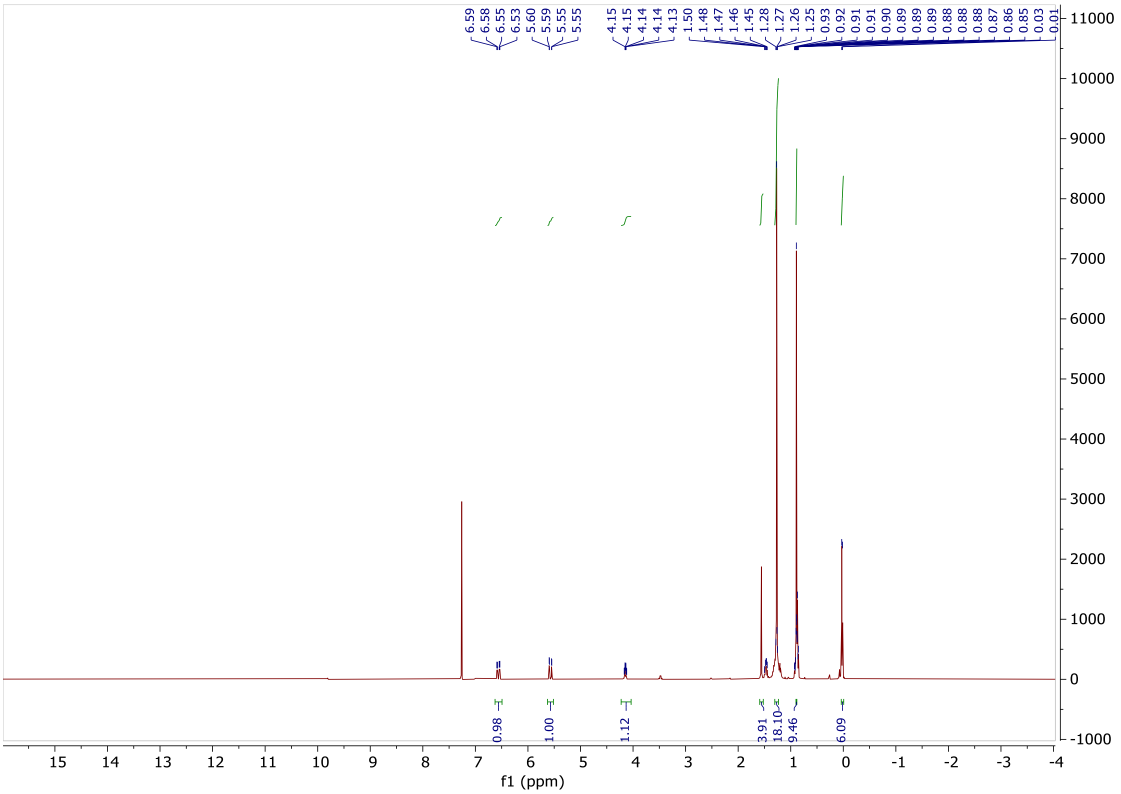

^{13}C NMR (101 MHz, $CDCl_3$) of **4a**

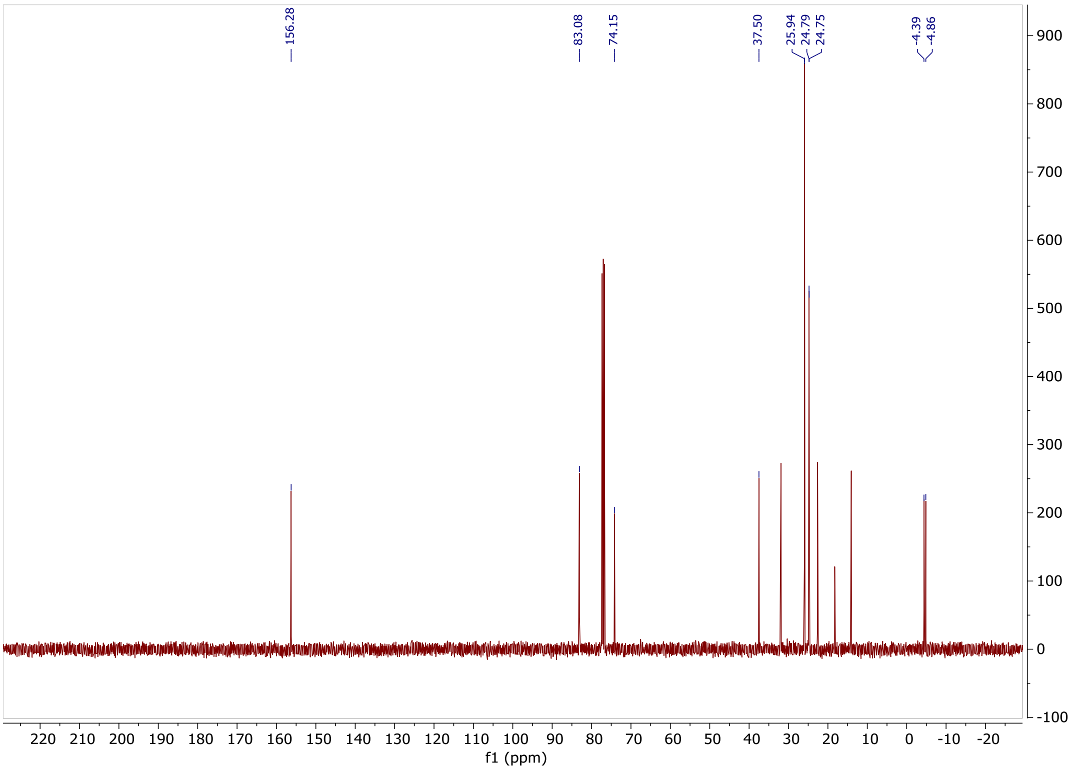

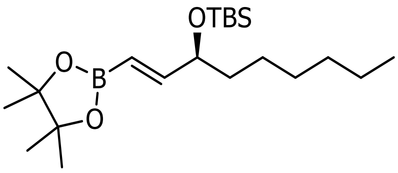

^{1}H NMR (400 MHz, $CDCl_3$) of **4b**

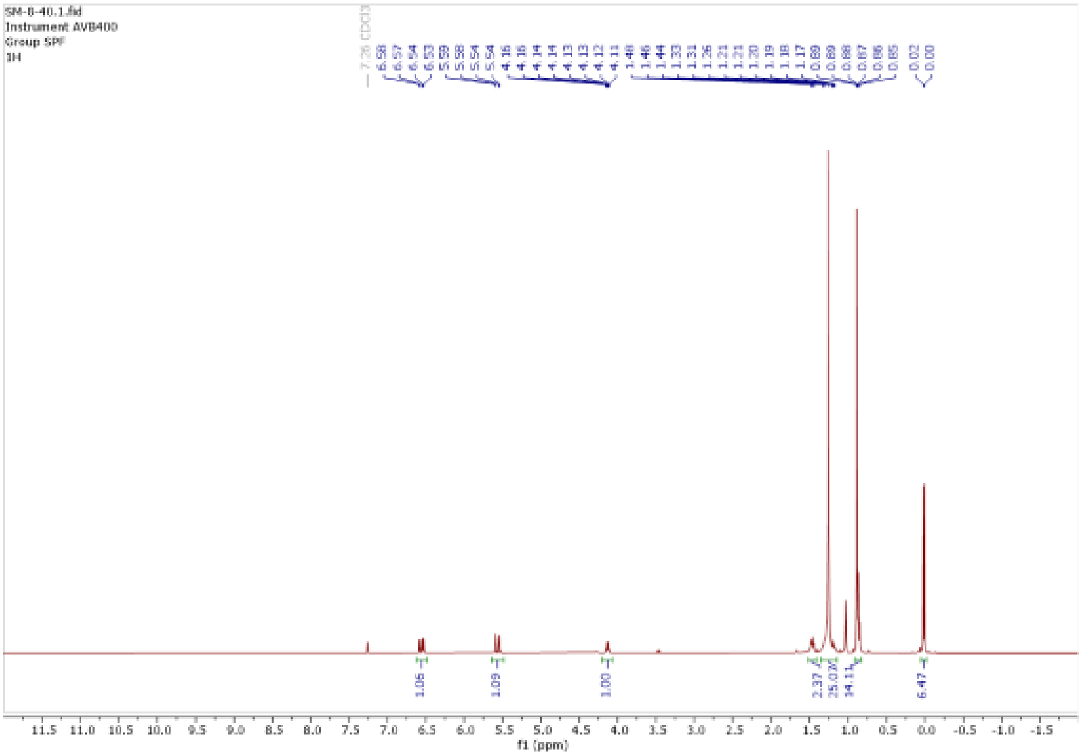

^{13}C NMR (101 MHz, $CDCl_3$) of **4b**

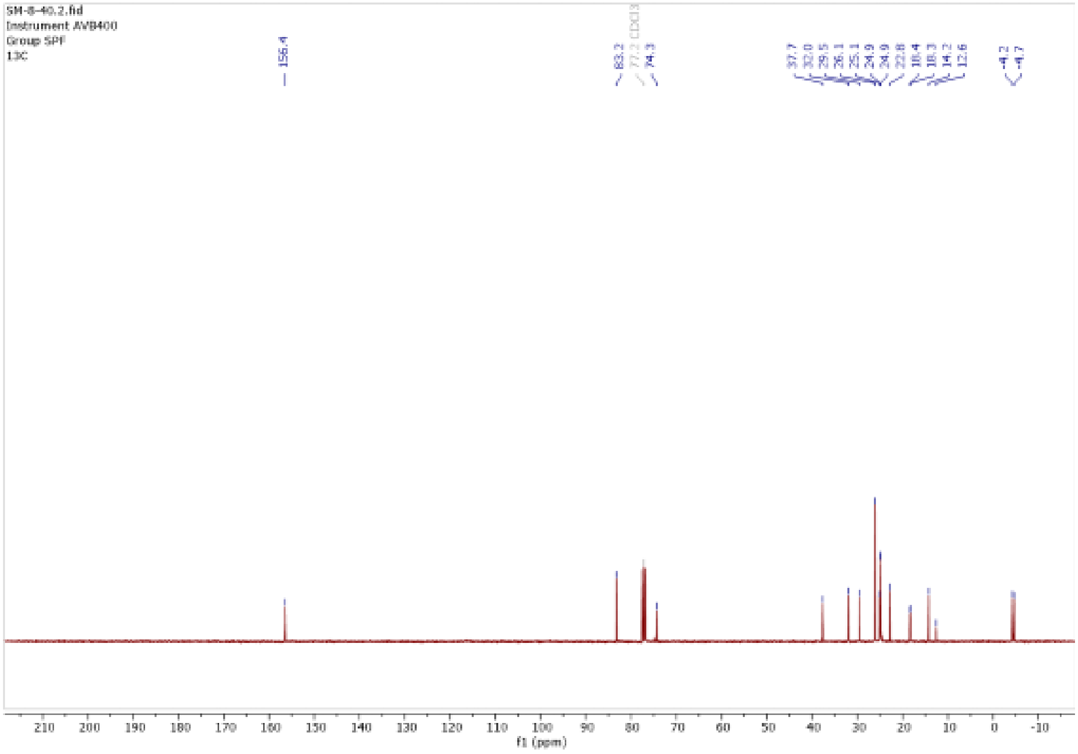

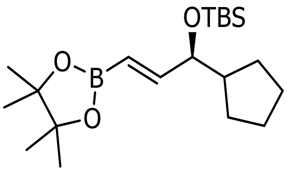

^{1}H NMR (400 MHz, $CDCl_3$) of **4c**

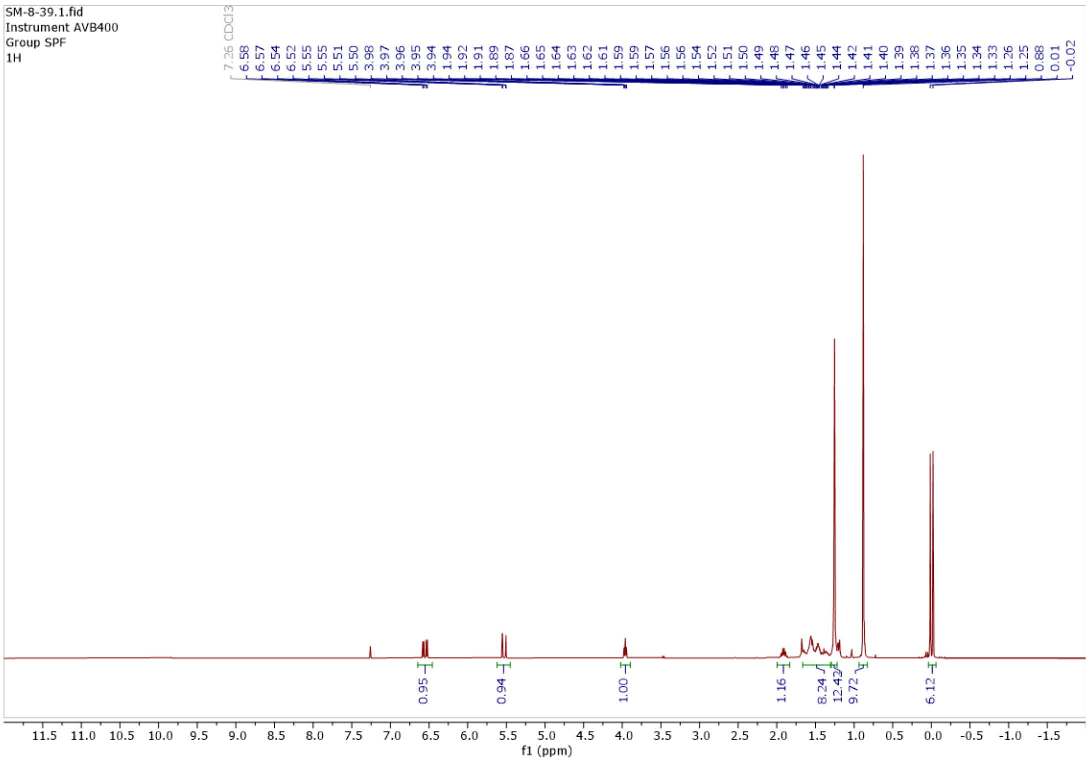

^{13}C NMR (101 MHz, $CDCl_3$) of **4c**

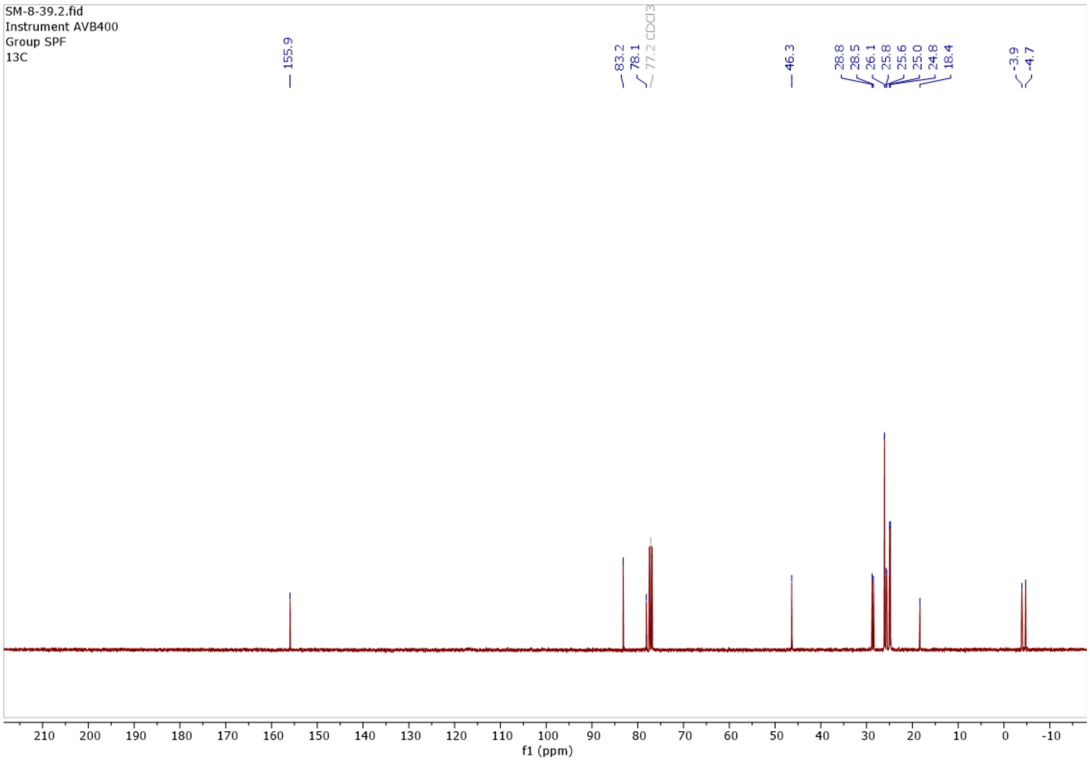

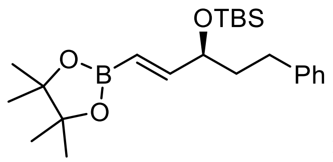

^{1}H NMR (400 MHz, $CDCl_3$) of **4d**

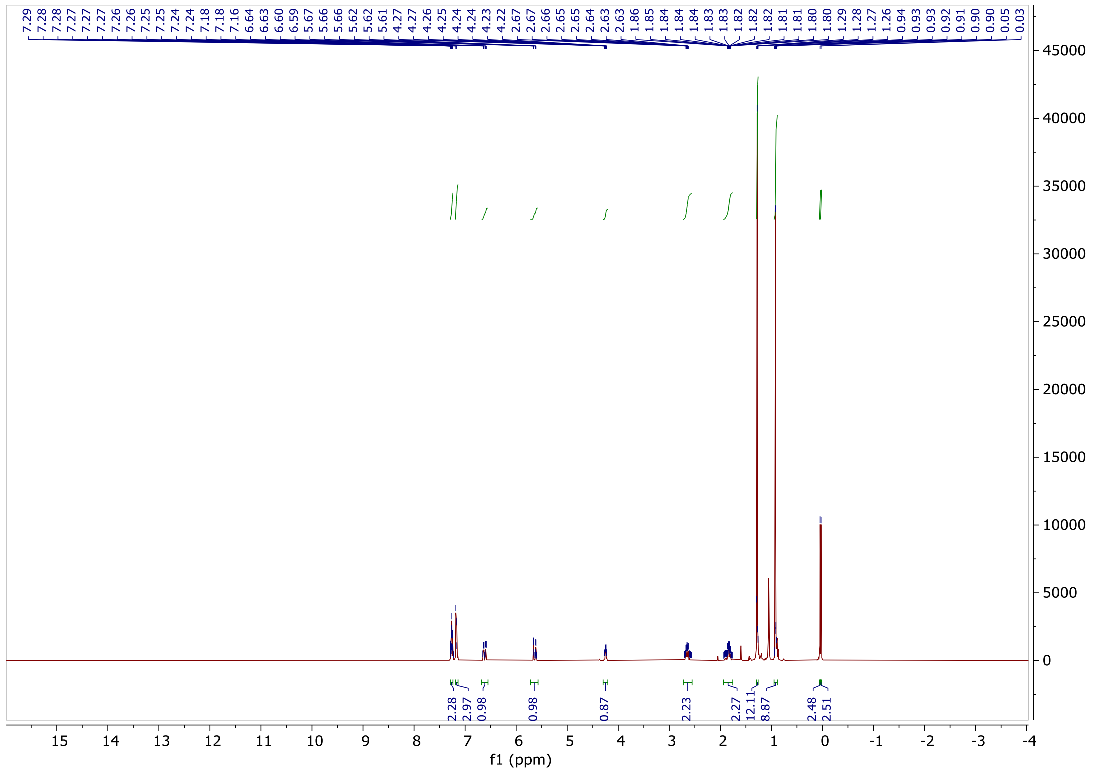

^{13}C NMR (101 MHz, $CDCl_3$) of **4d**

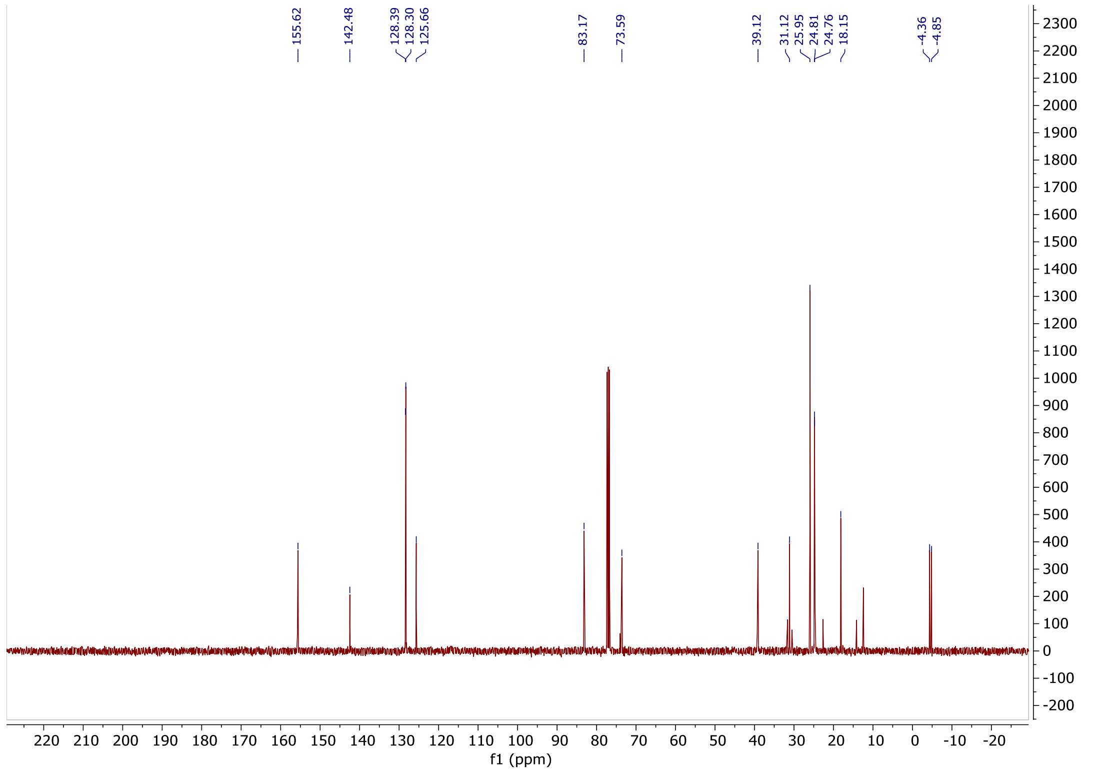

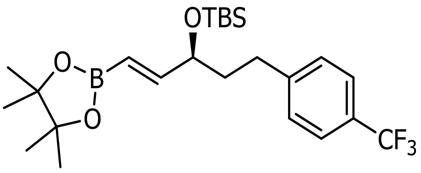

^{1}H NMR (400 MHz, $CDCl_3$) of **4e**

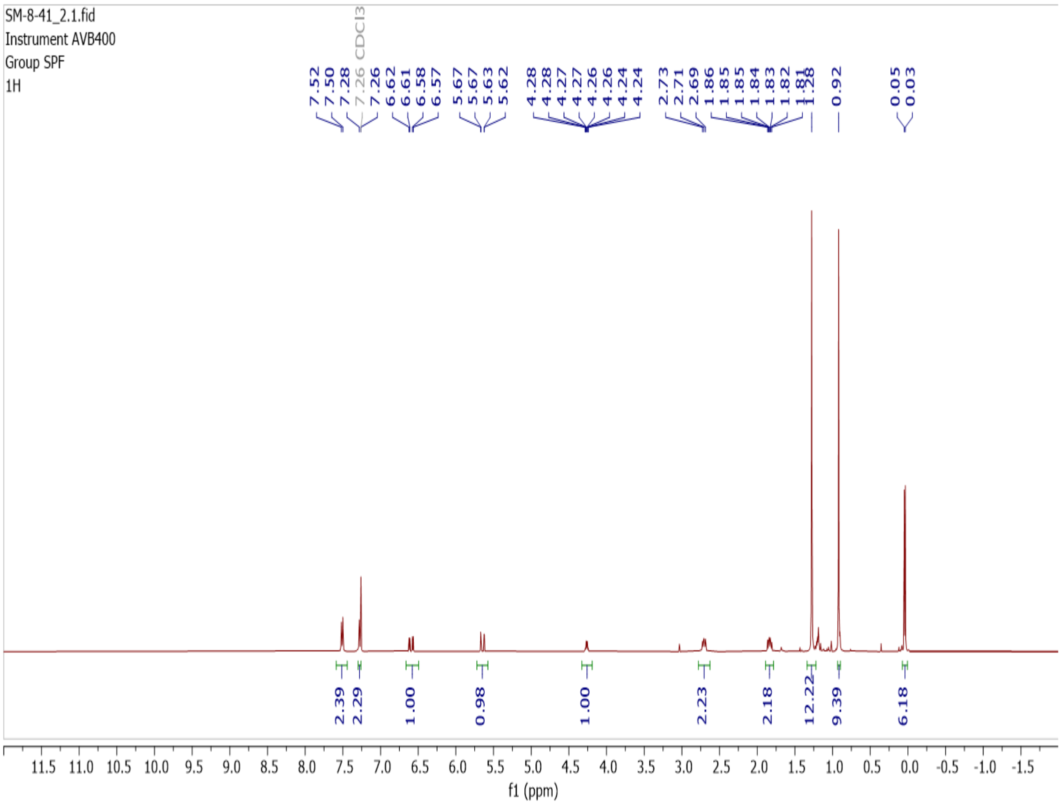

^{13}C NMR (101 MHz, $CDCl_3$) of **4e**

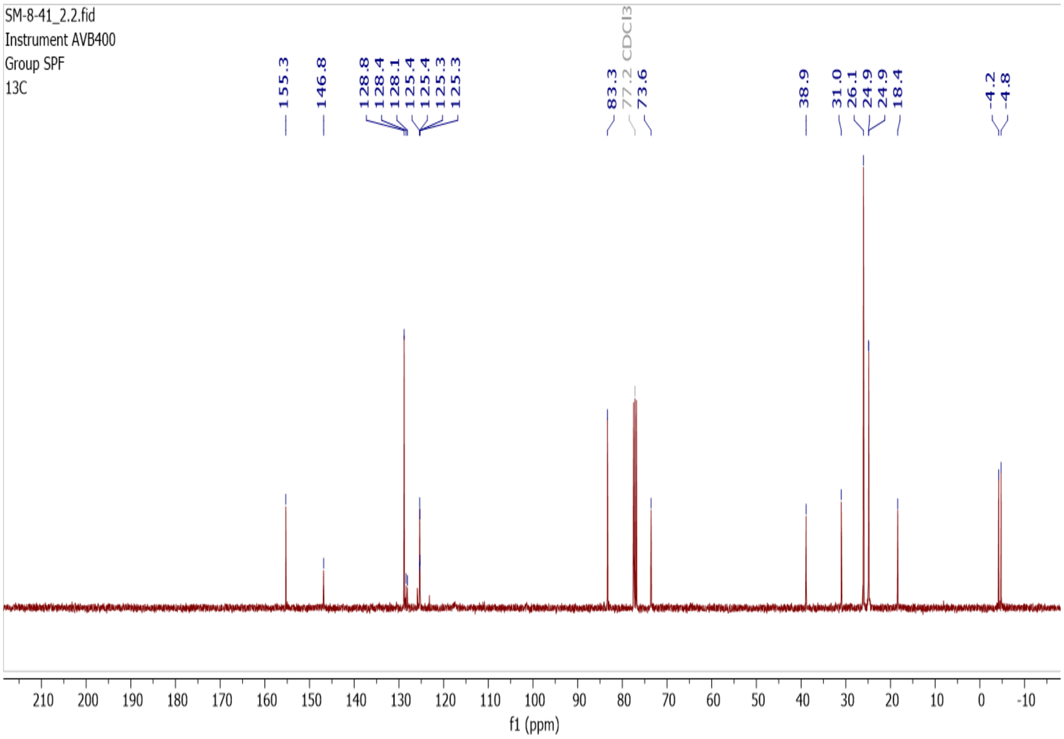

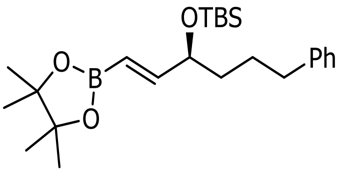

^{1}H NMR (400 MHz, $CDCl_3$) of **4f**

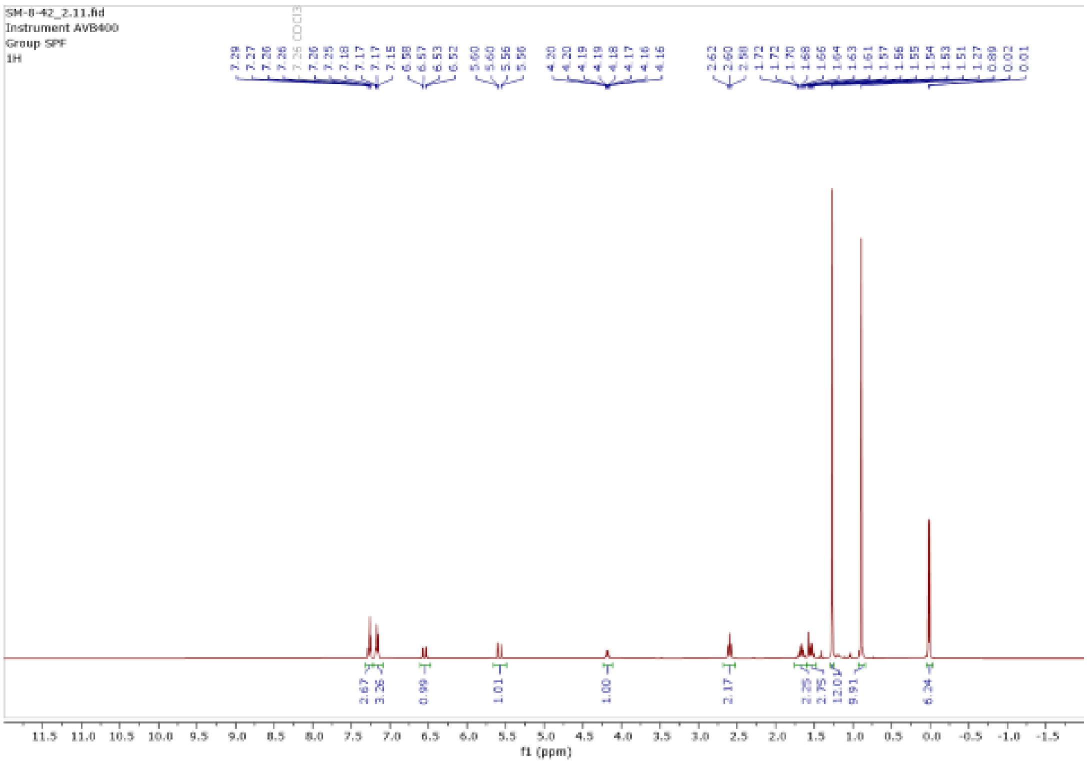

^{13}C NMR (101 MHz, $CDCl_3$) of **4f**

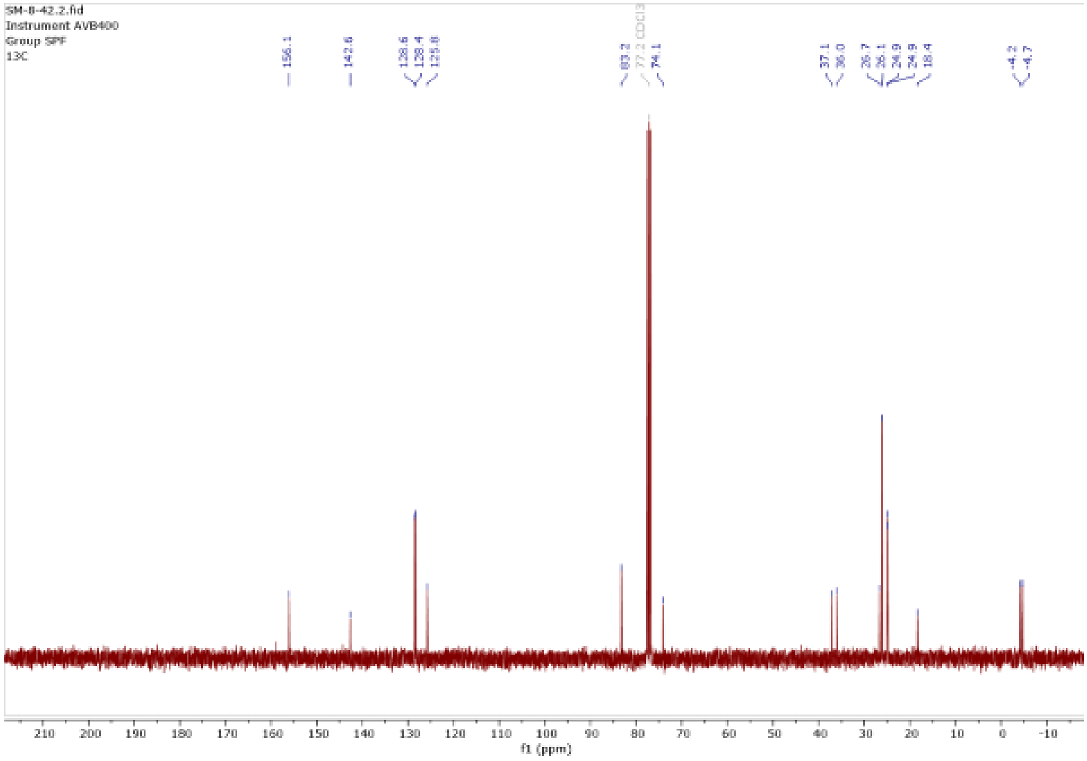

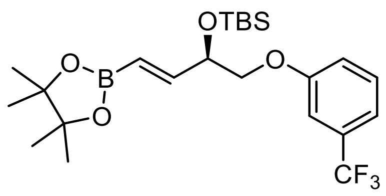

^{1}H NMR (400 MHz, $CDCl_3$) of **4g**

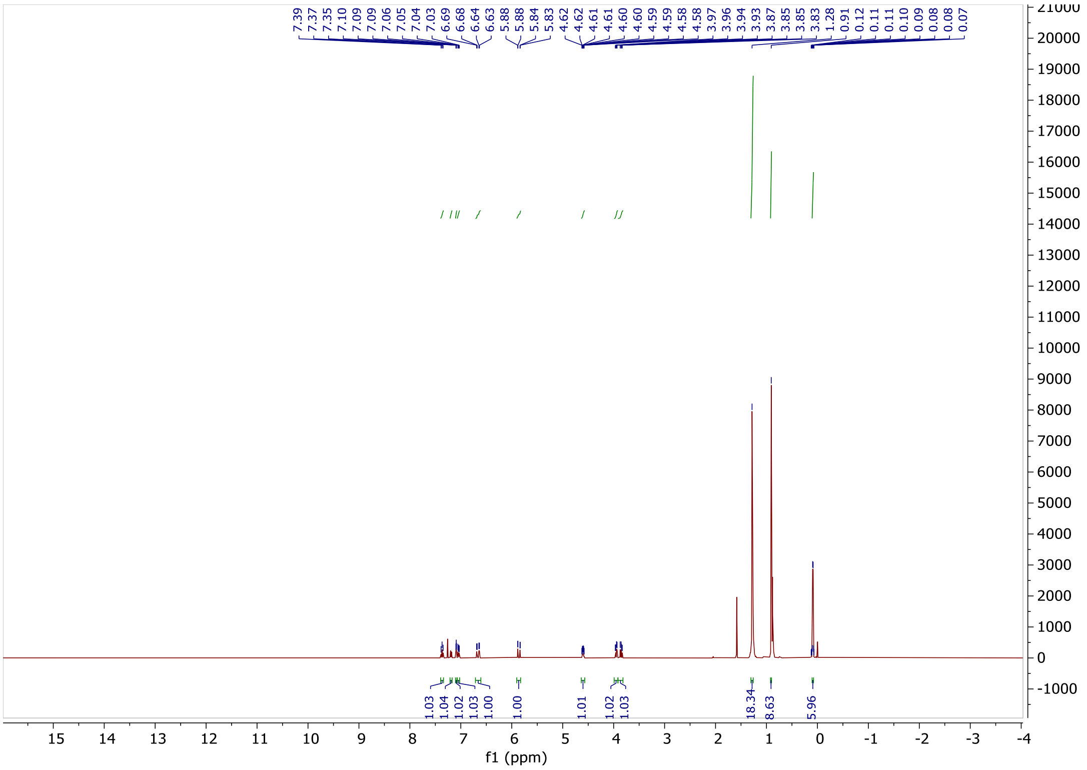

^{13}C NMR (101 MHz, $CDCl_3$) of **4g**

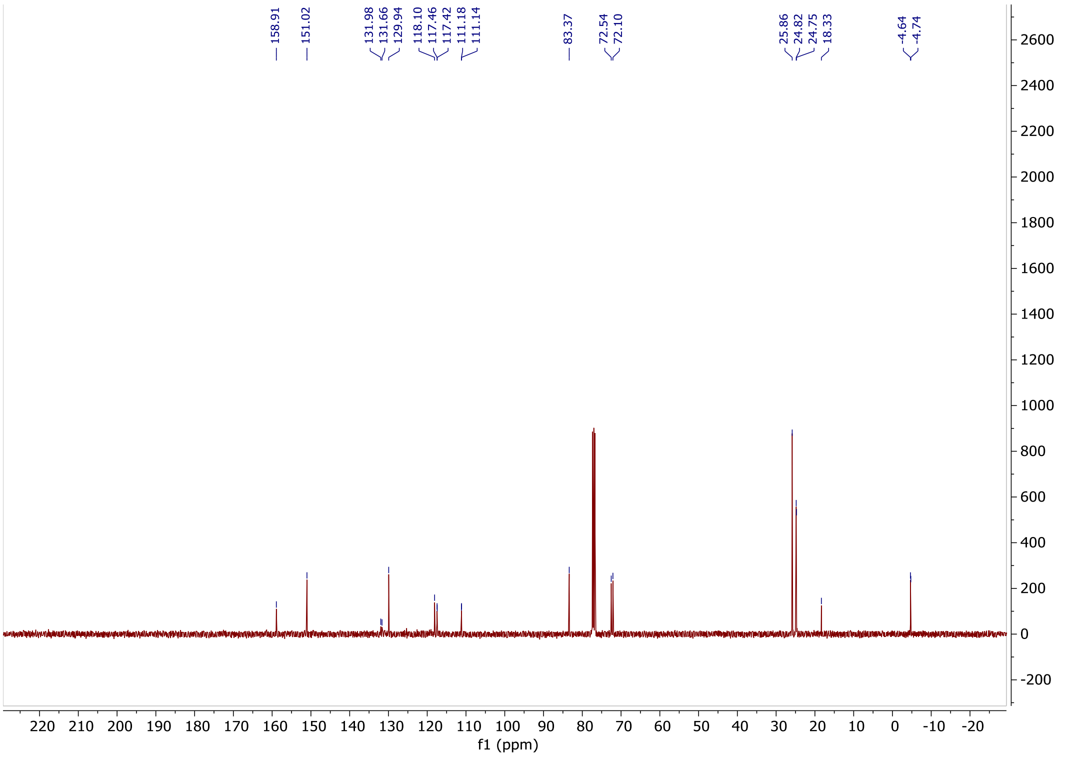

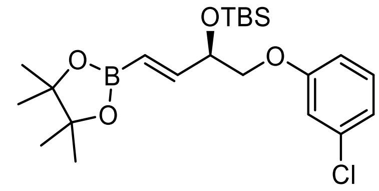

^{1}H NMR (400 MHz, CDCl$_3$) of **4h**

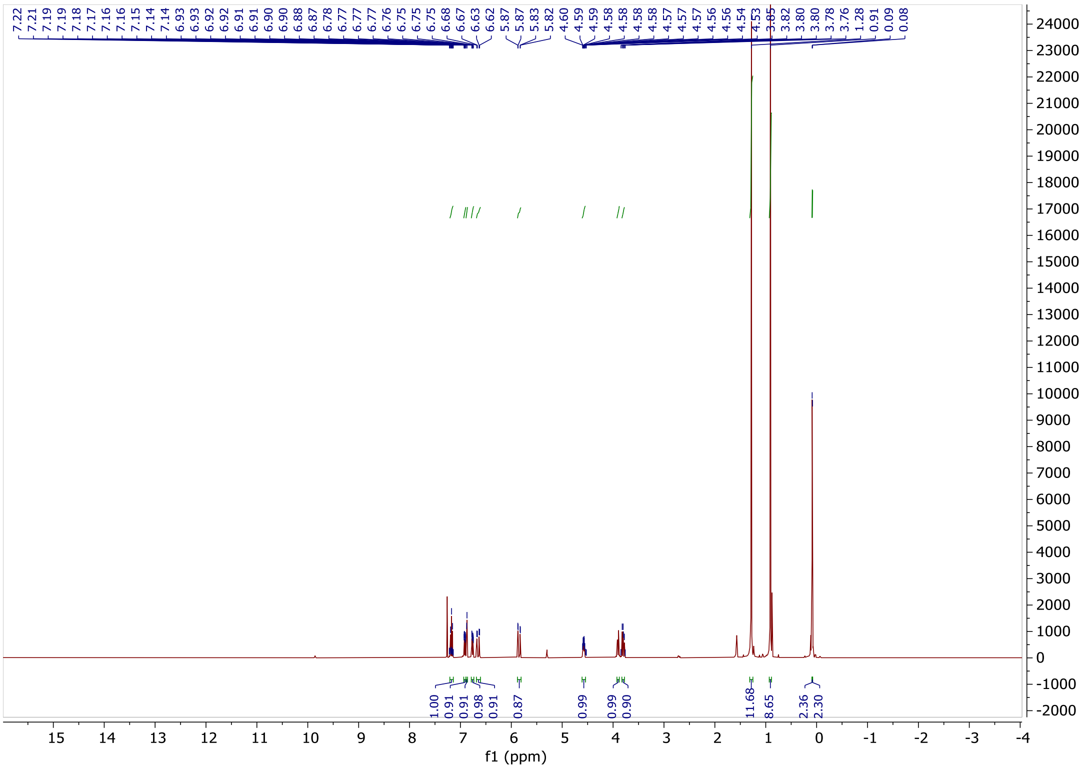

^{13}C NMR (101 MHz, CDCl$_3$) of **4h**

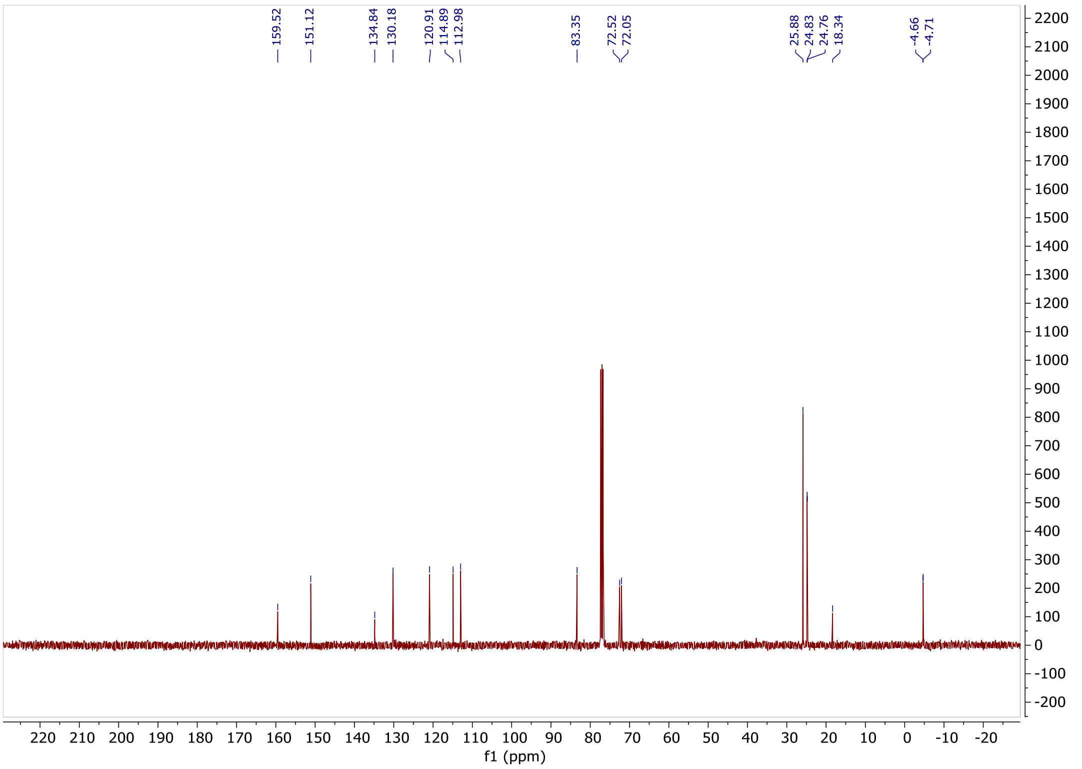

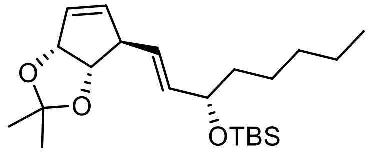

^{1}H NMR (400 MHz, $CDCl_3$) of **6**

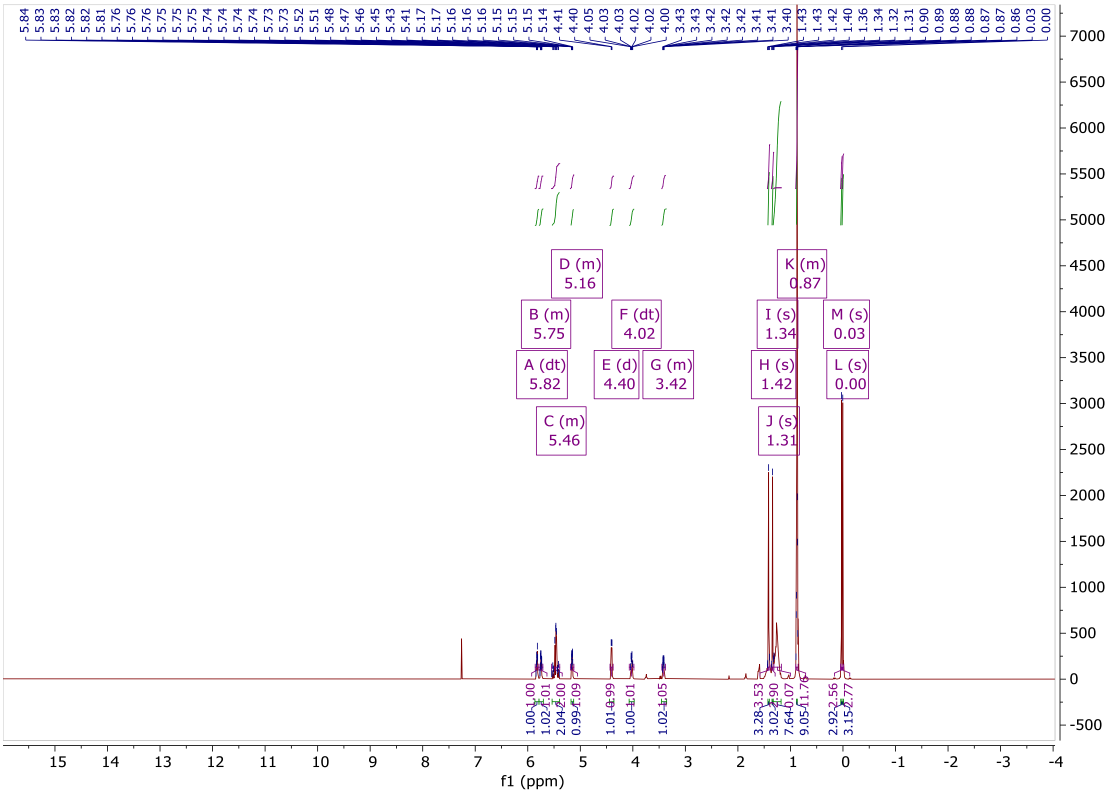

^{13}C NMR (101 MHz, $CDCl_3$) of **6**

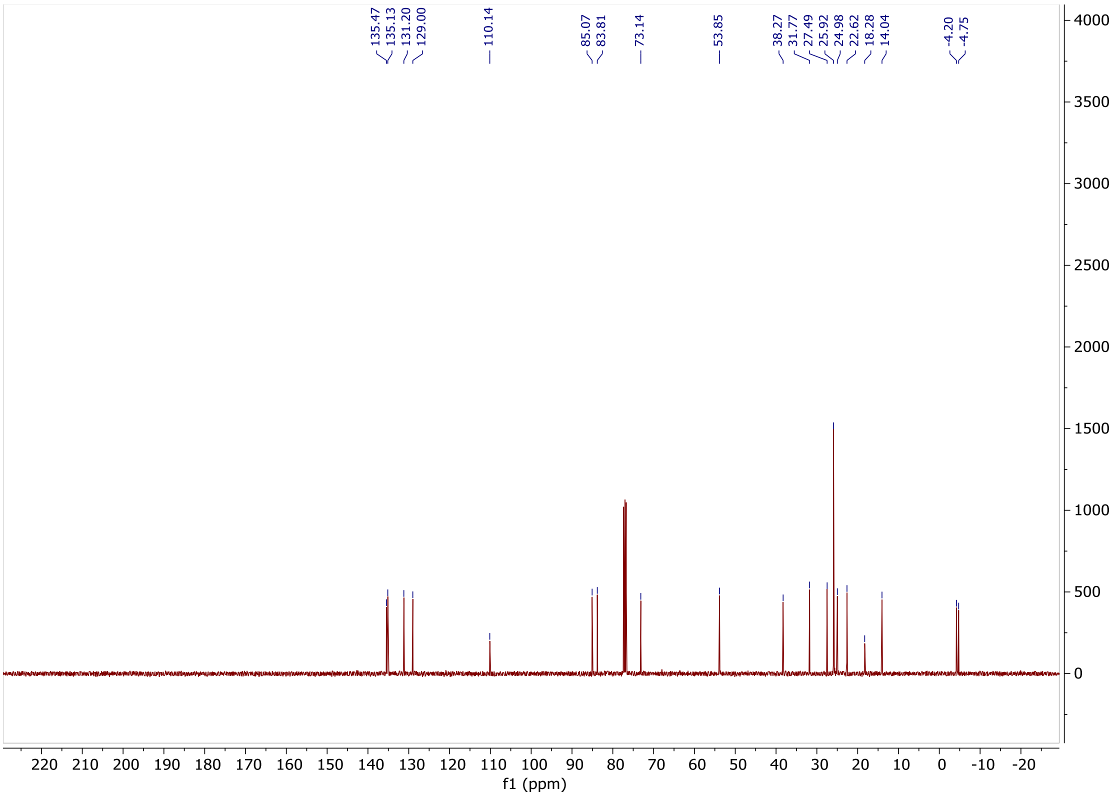

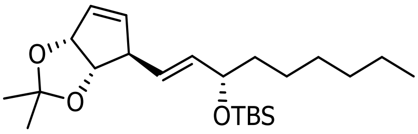

^{1}H NMR (500 MHz, $CDCl_3$) of **7**

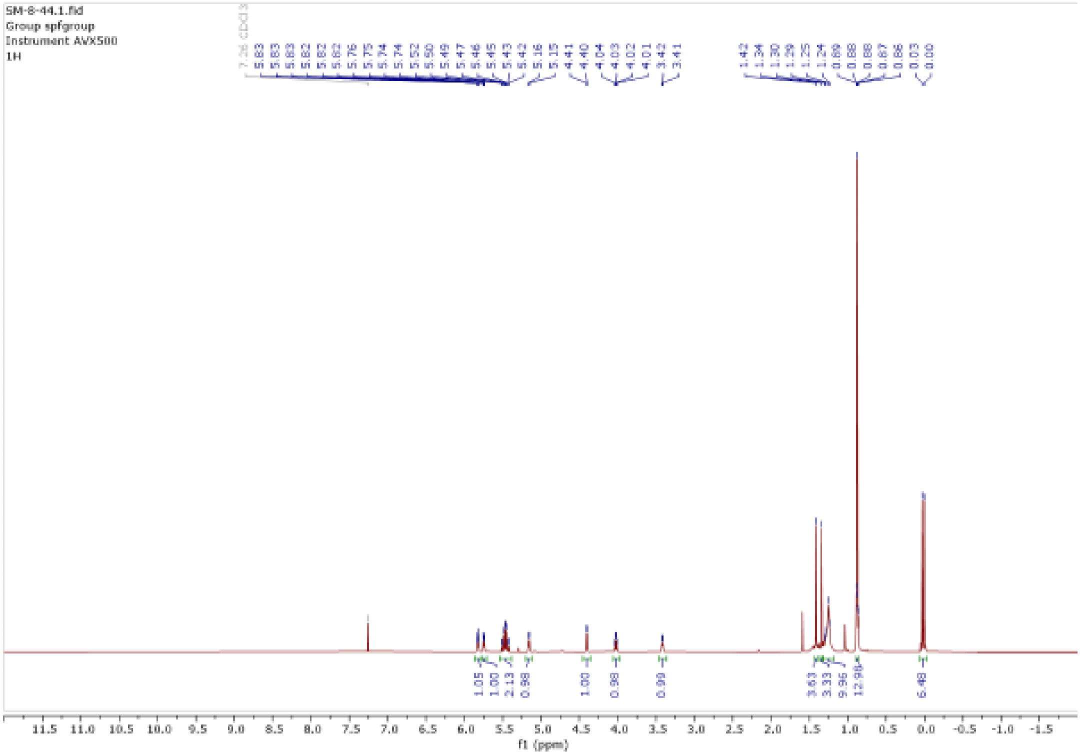

^{13}C NMR (126 MHz, $CDCl_3$) of **7**

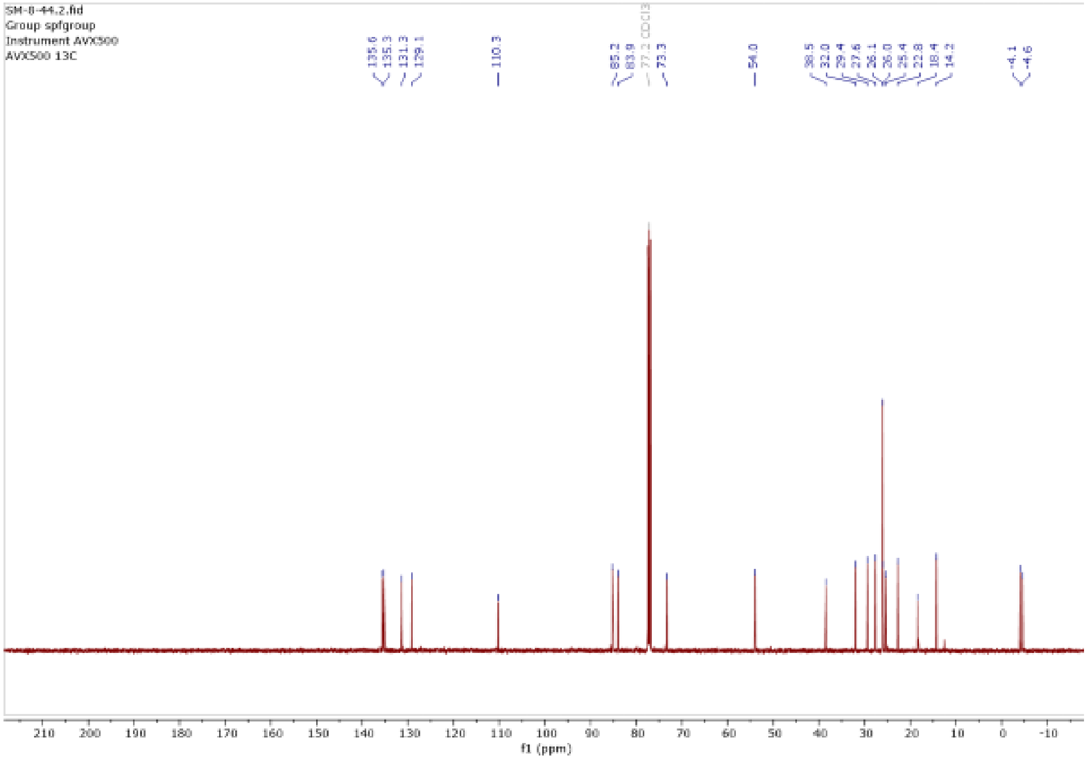

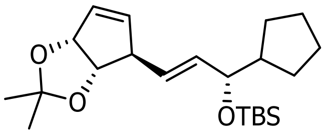

^{1}H NMR (500 MHz, $CDCl_3$) of **8**

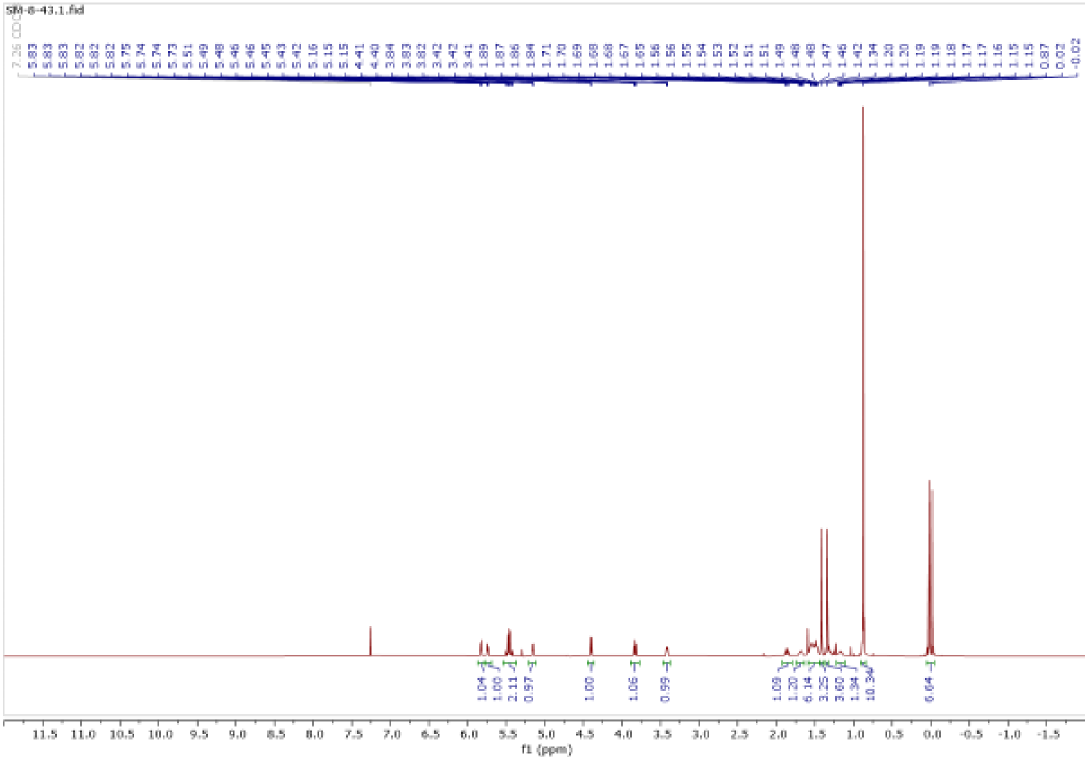

^{13}C NMR (126 MHz, $CDCl_3$) of **8**

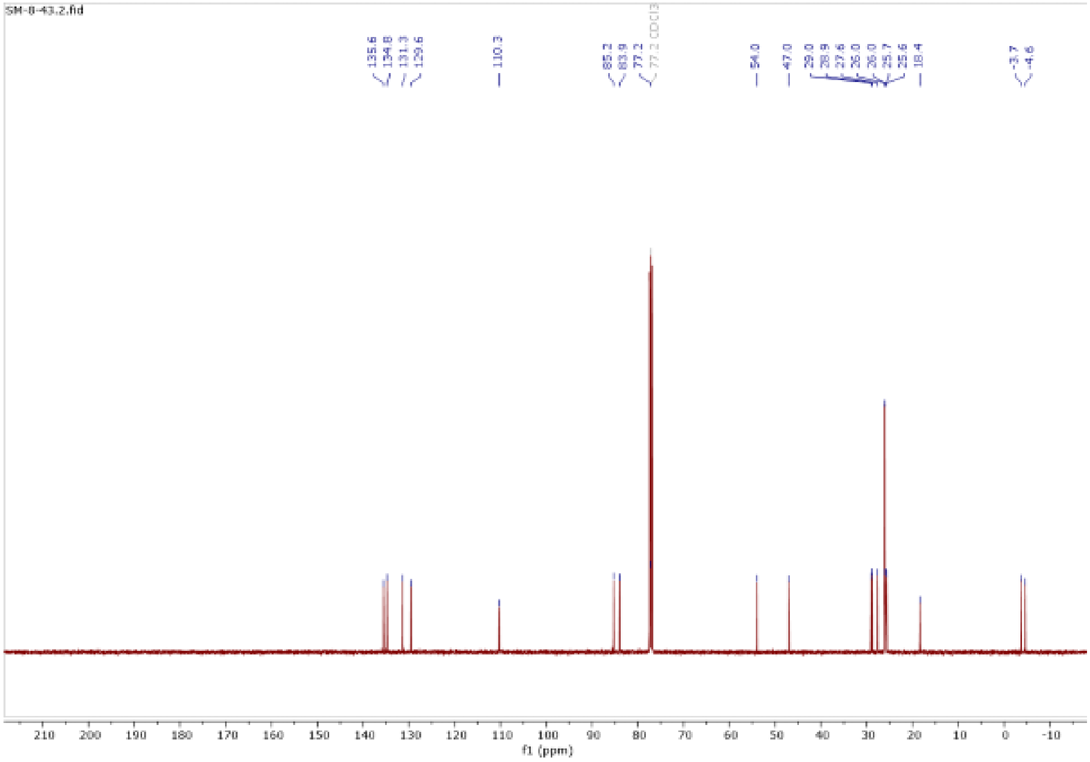

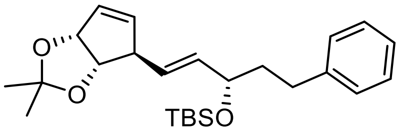

^{1}H NMR (400 MHz, $CDCl_3$) of **9**

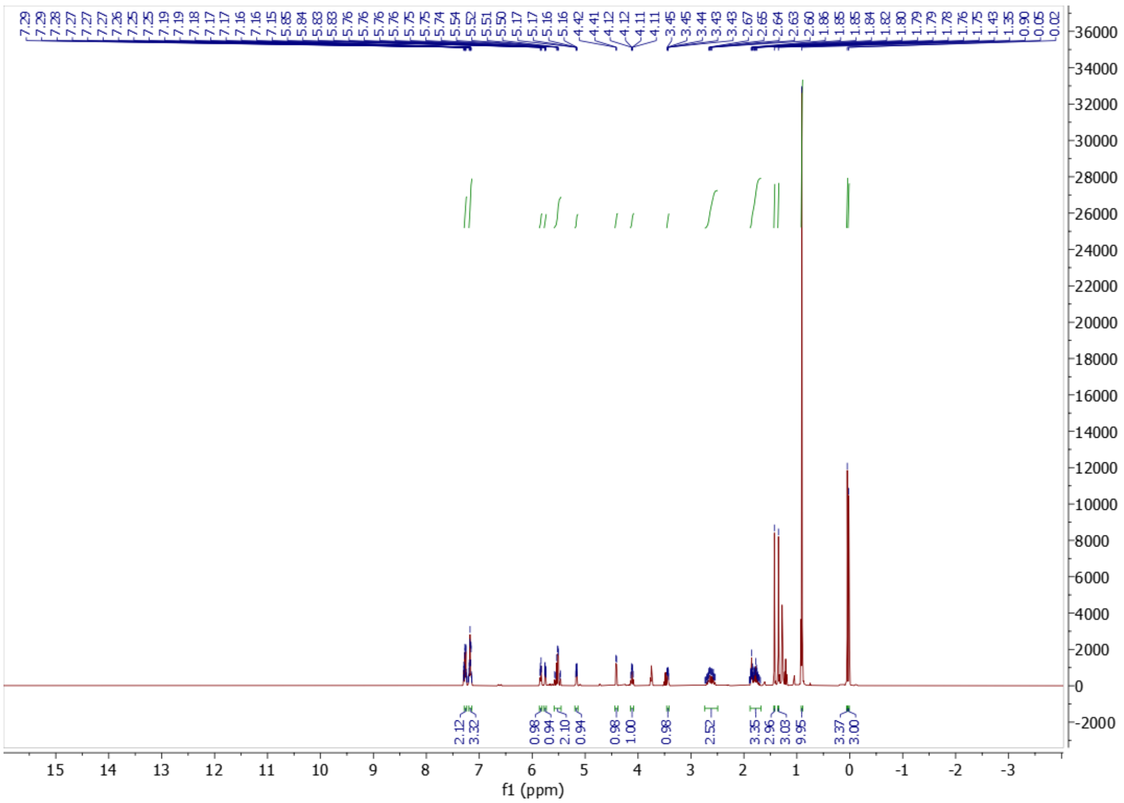

^{13}C NMR (101 MHz, $CDCl_3$) of **9**

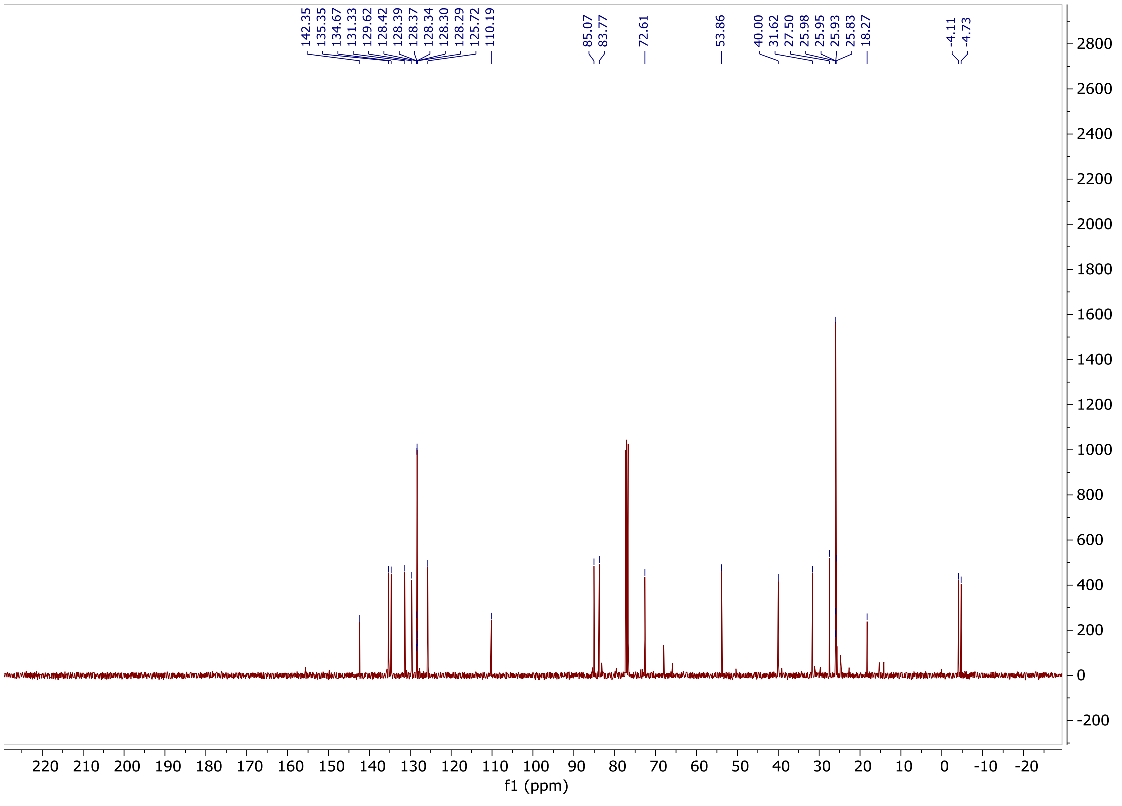

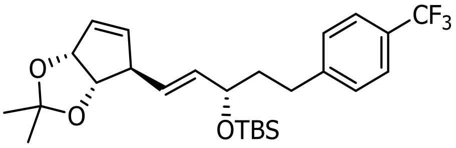

^{1}H NMR (400 MHz, $CDCl_3$) of **10**

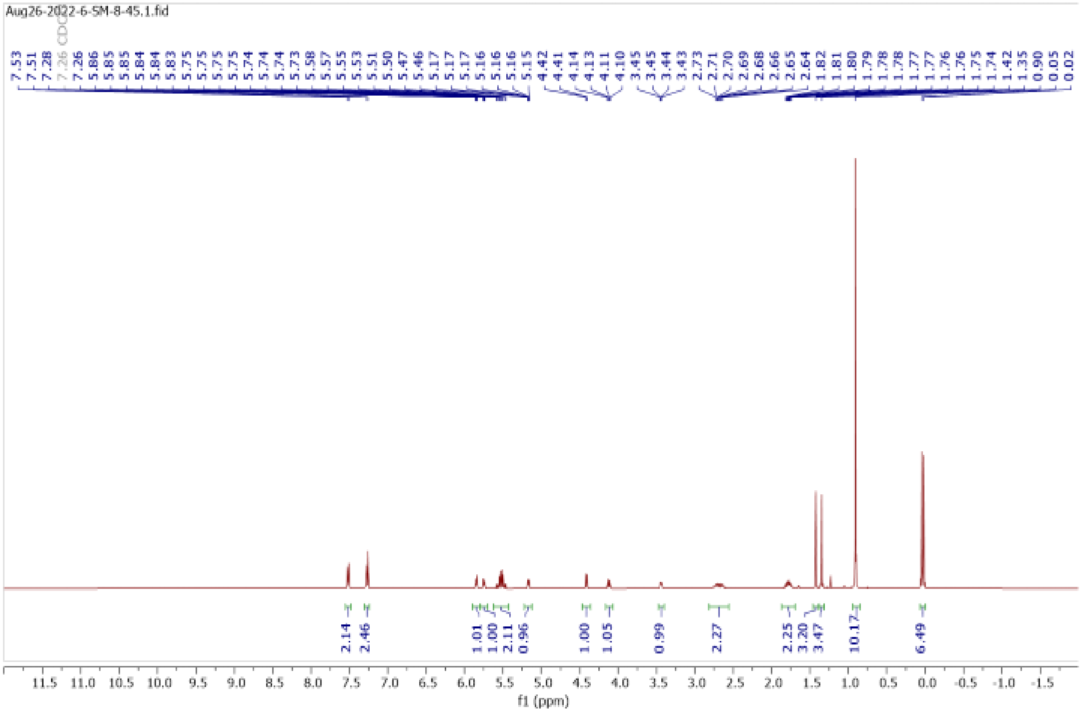

^{13}C NMR (101 MHz, $CDCl_3$) of **10**

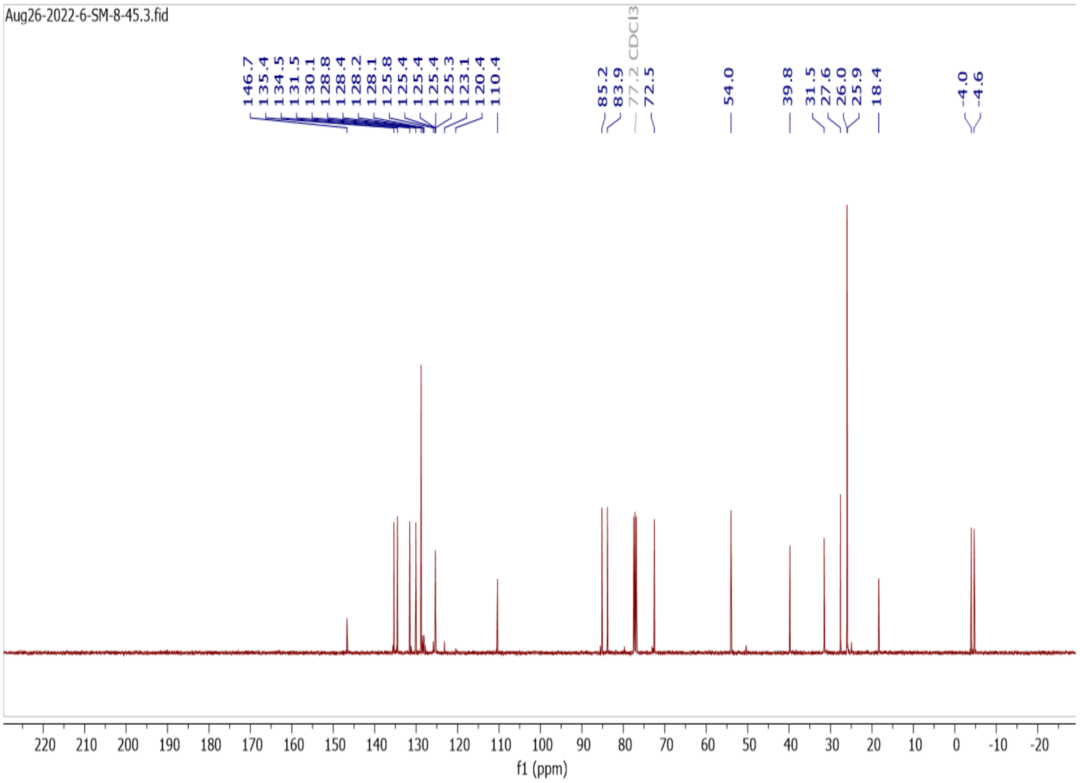

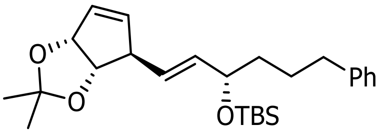

^{1}H NMR (400 MHz, $CDCl_3$) of **11**

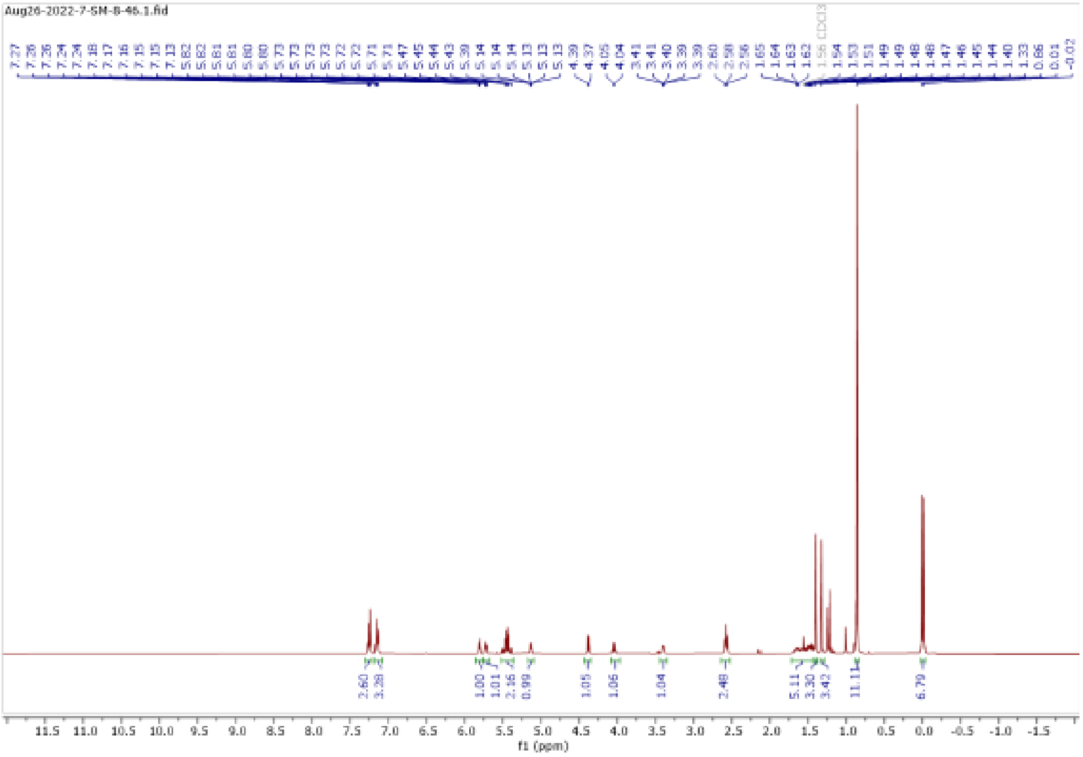

^{13}C NMR (101 MHz, $CDCl_3$) of **11**

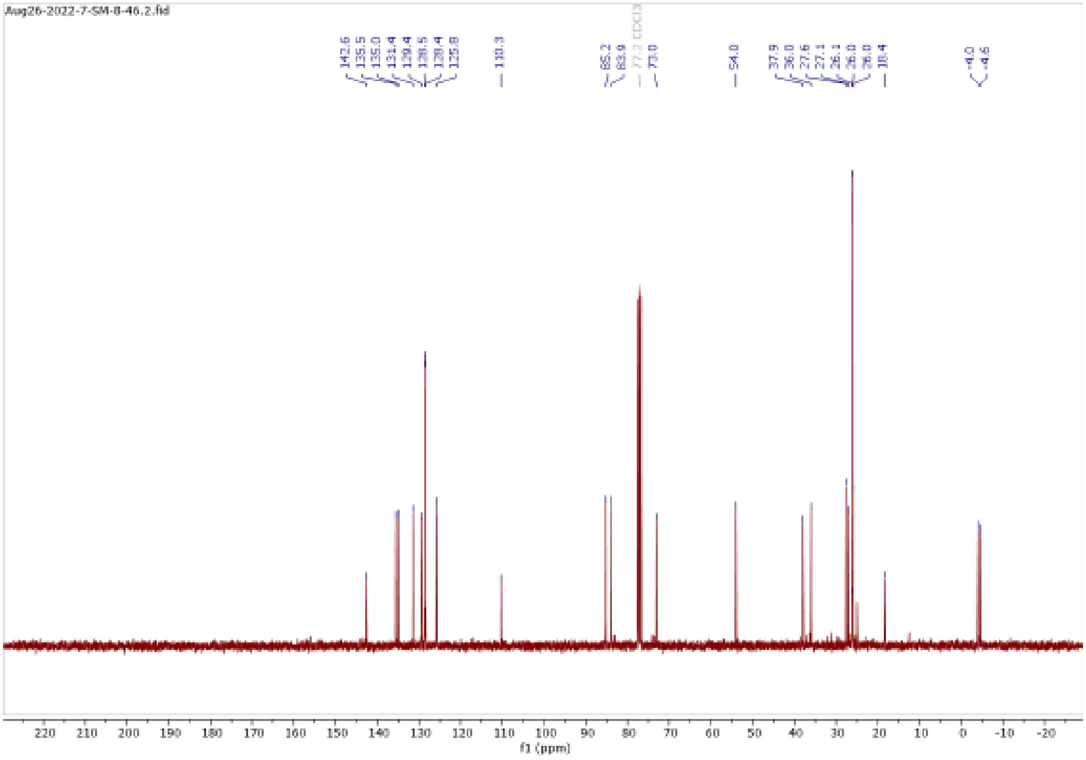

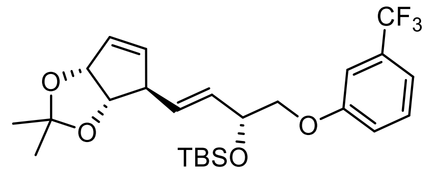

^{1}H NMR (400 MHz, $CDCl_3$) of **12**

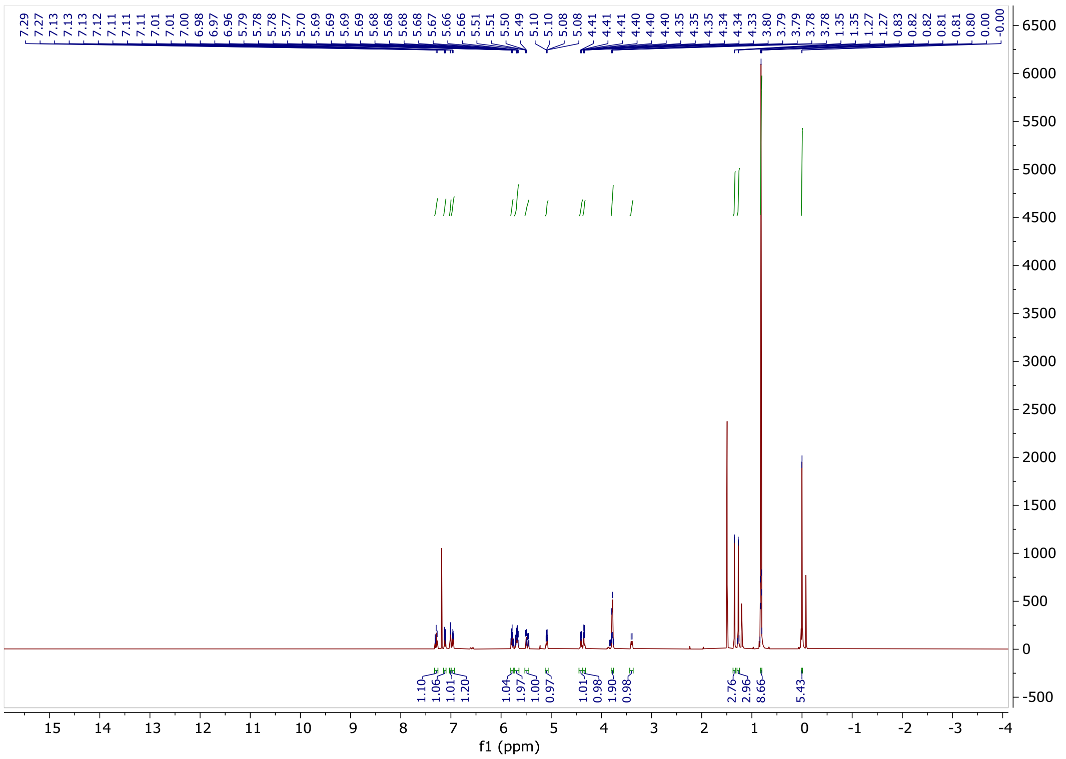

^{13}C NMR (101 MHz, $CDCl_3$) of **12**

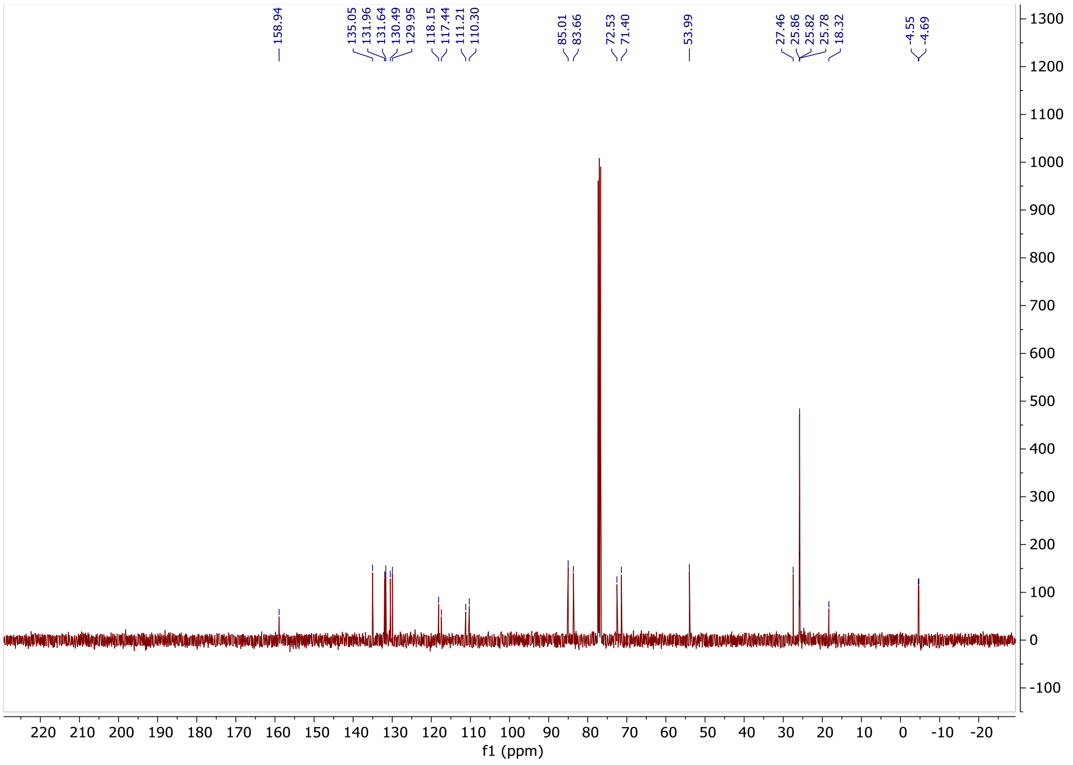

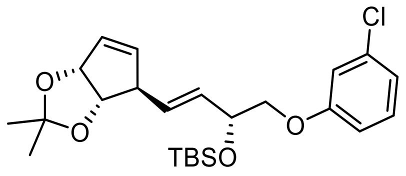

^{1}H NMR (400 MHz, $CDCl_3$) of **13**

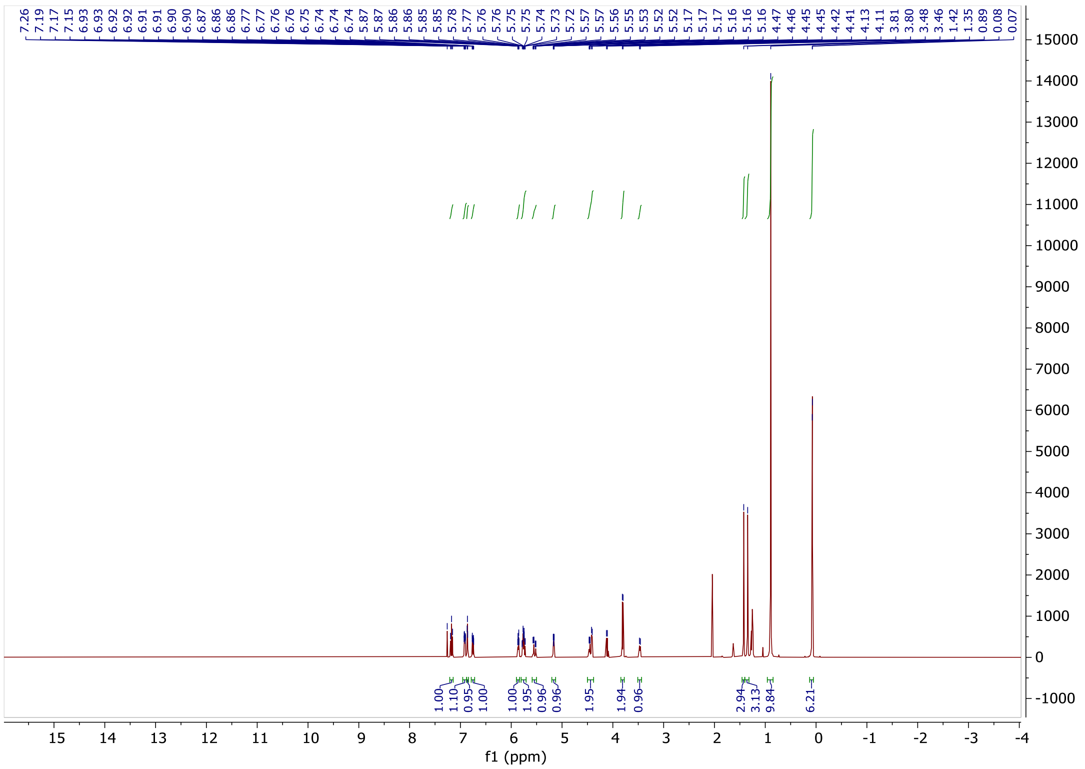

^{13}C NMR (101 MHz, $CDCl_3$) of **13**

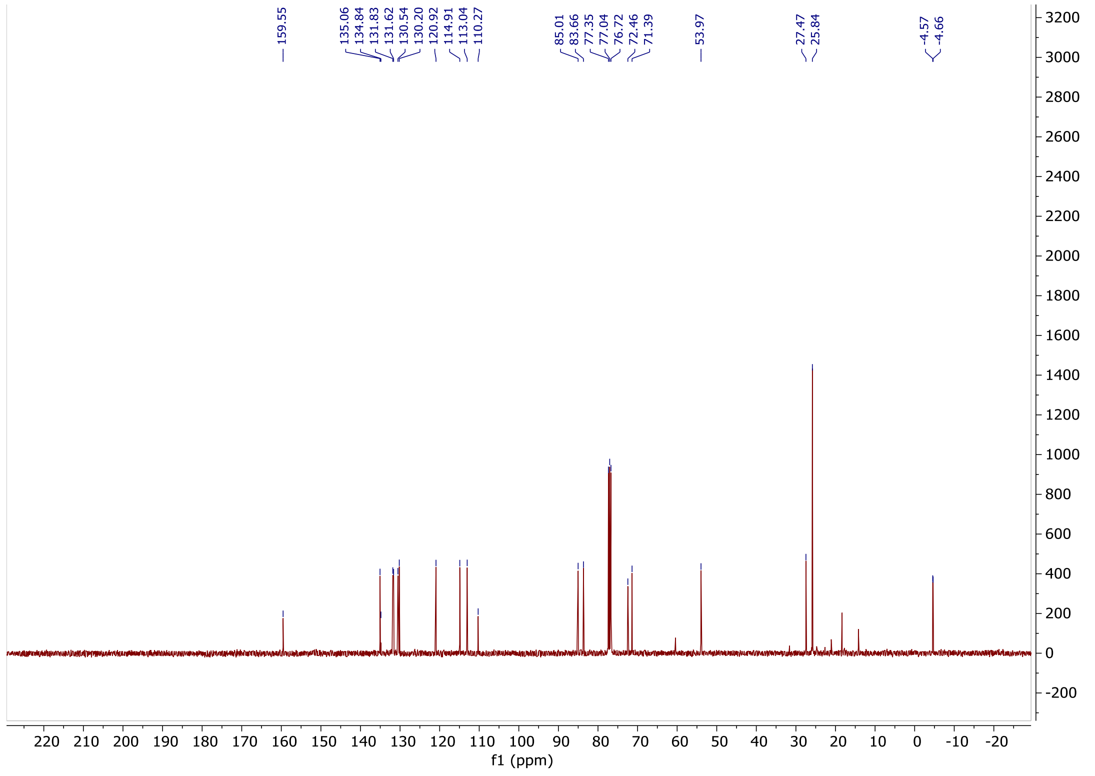

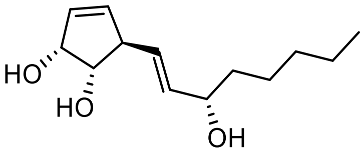

^{1}H NMR (400 MHz, $CDCl_3$) of **S4**

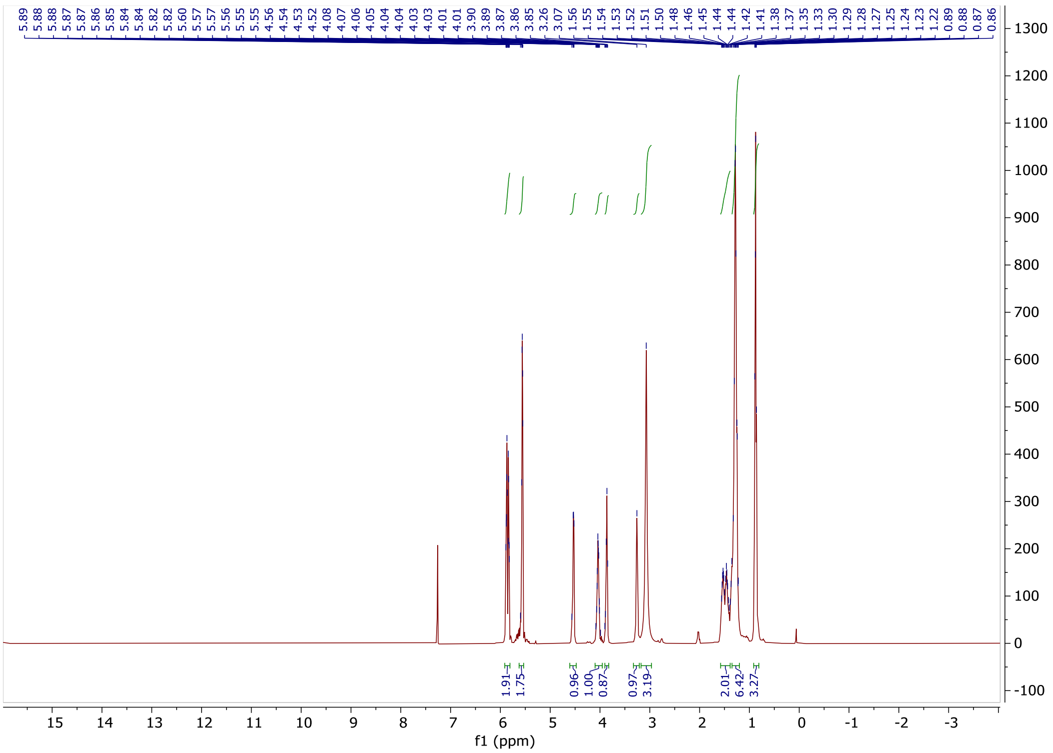

^{13}C NMR (101 MHz, $CDCl_3$) of **S4**

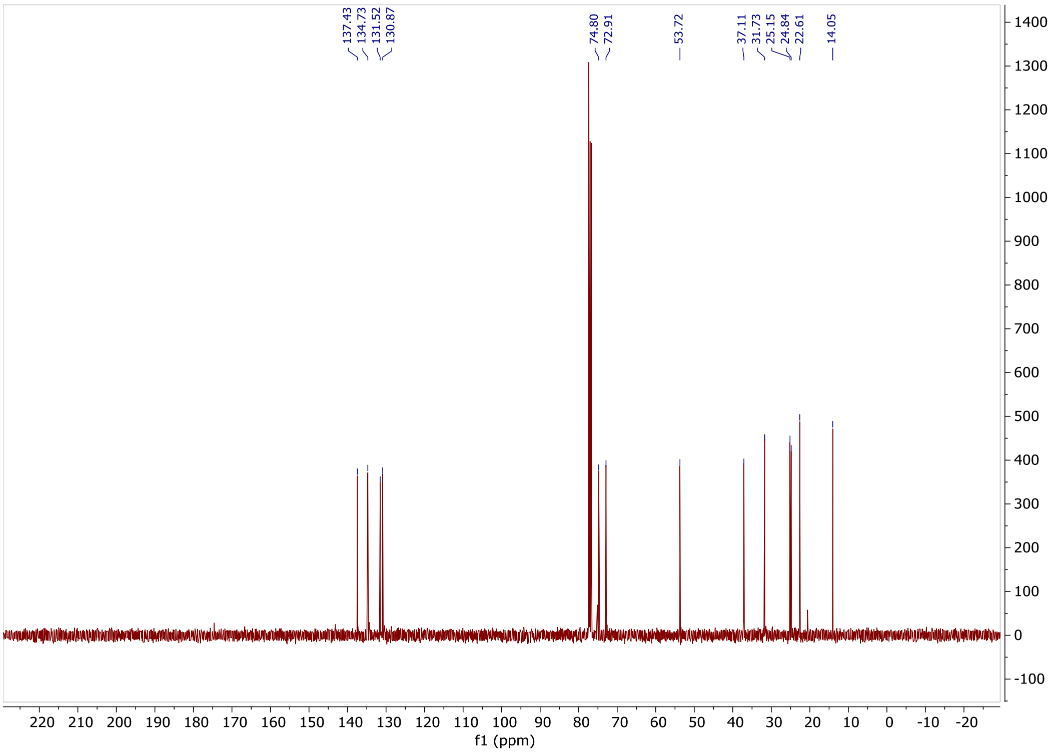

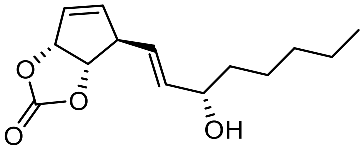

^{1}H NMR (400 MHz, $CDCl_3$) of **14**

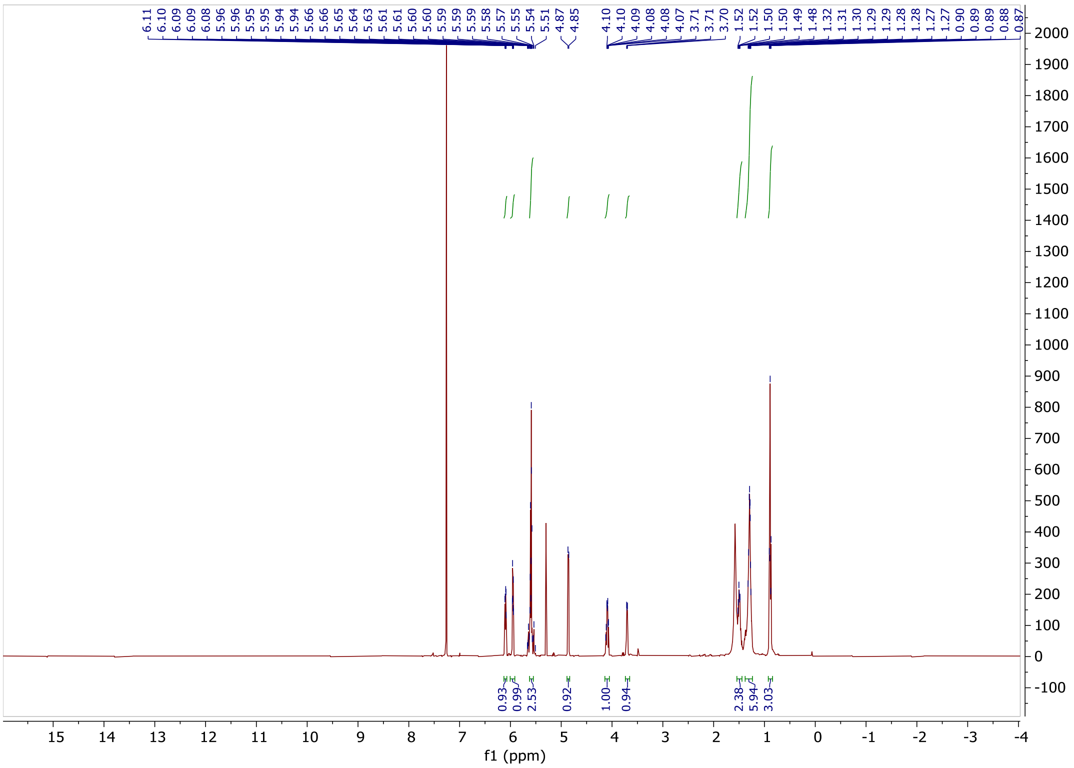

^{13}C NMR (101 MHz, $CDCl_3$) of **14**

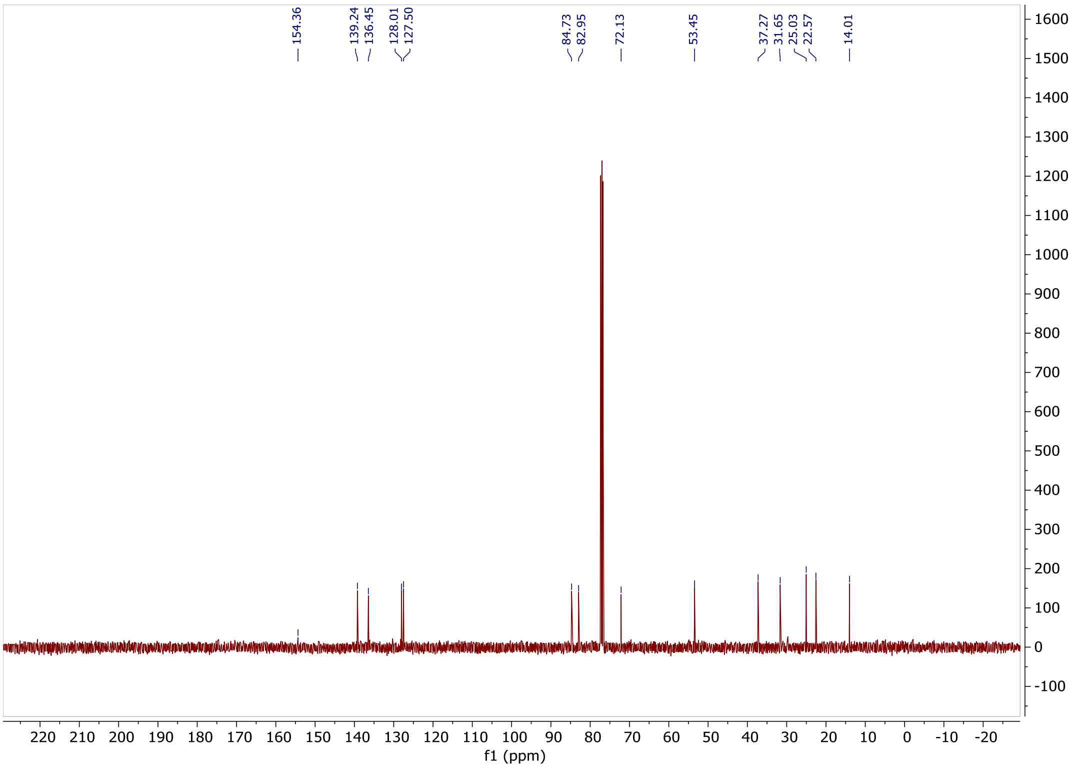

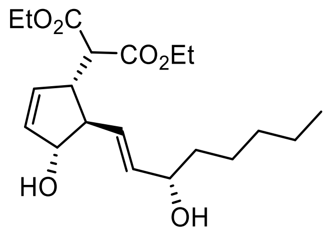

^{1}H NMR (400 MHz, $CDCl_3$) of **15**

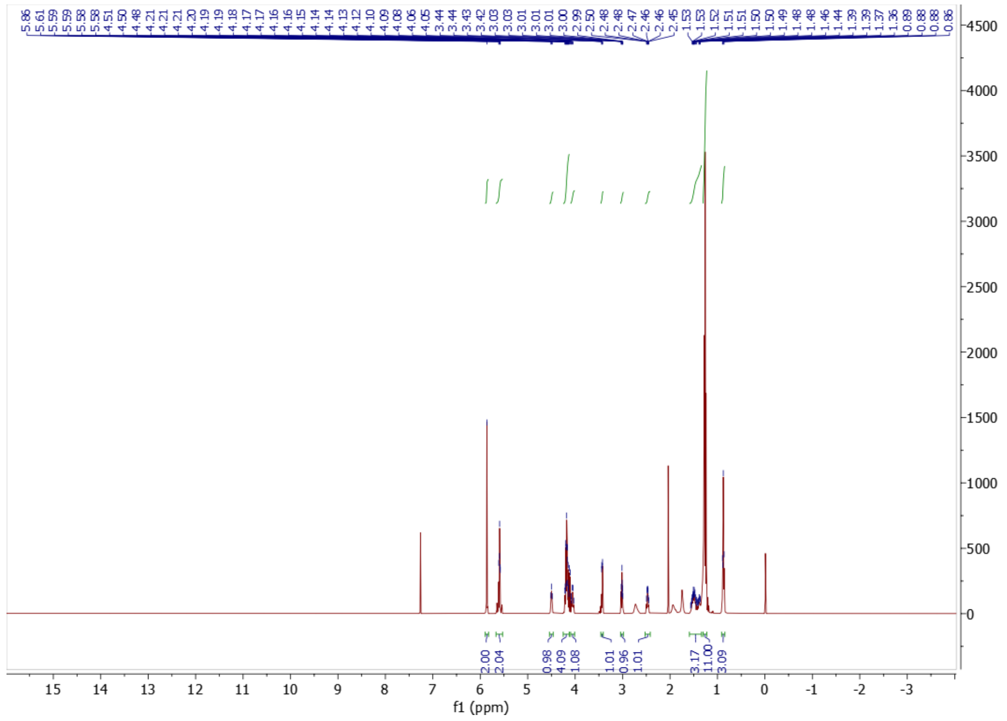

^{13}C NMR (101 MHz, $CDCl_3$) of **15**

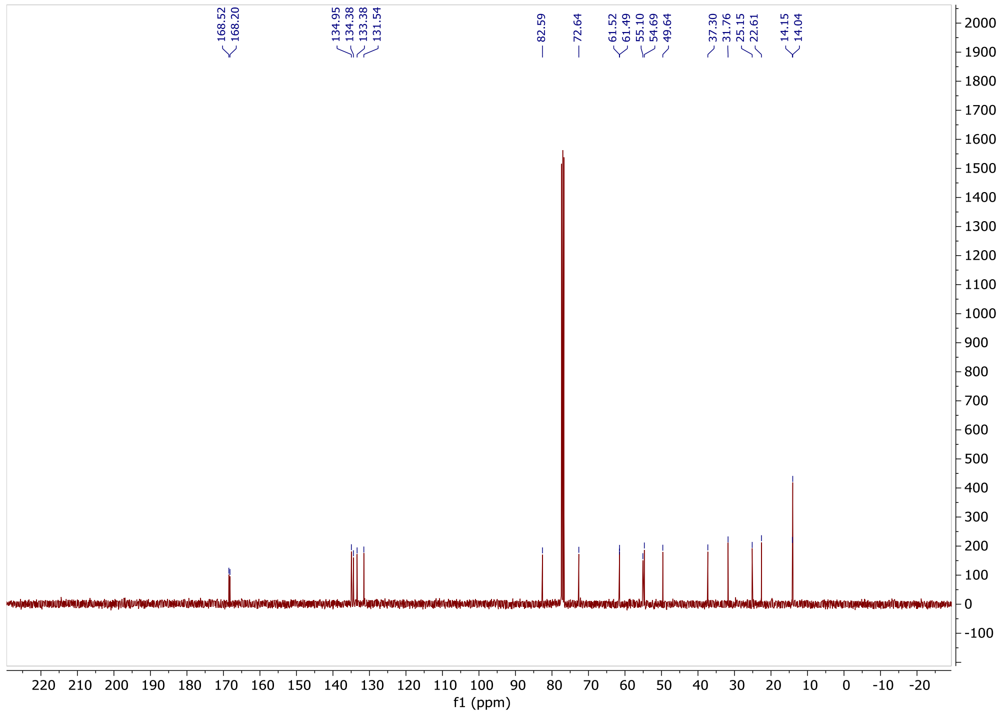

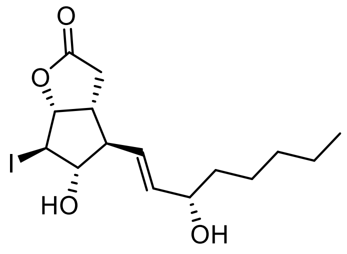

^{1}H NMR (400 MHz, C_2D_6O) of **S5**

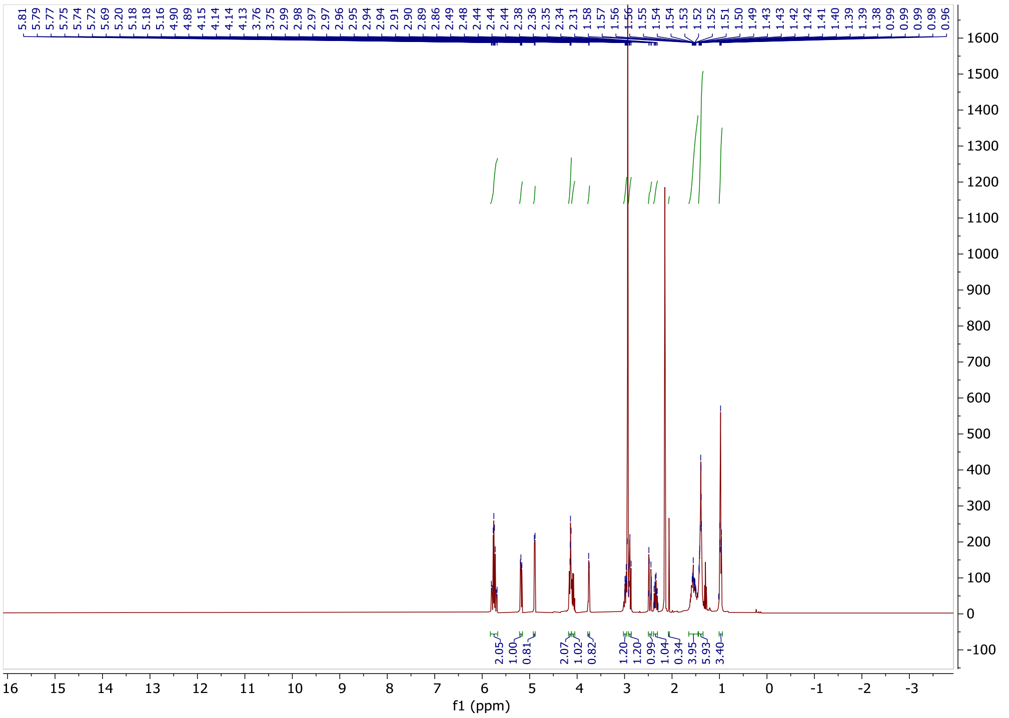

^{13}C NMR (101 MHz, C_2D_6O) of **S5**

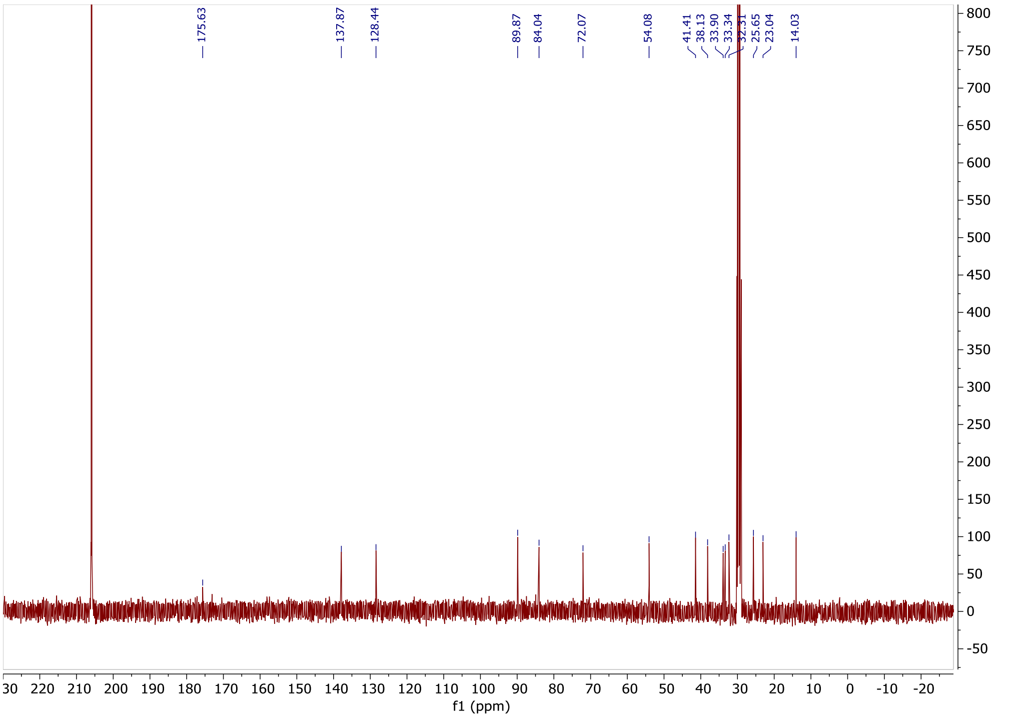

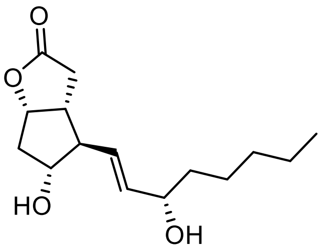

^{1}H NMR (400 MHz, $CDCl_3$) of **17**

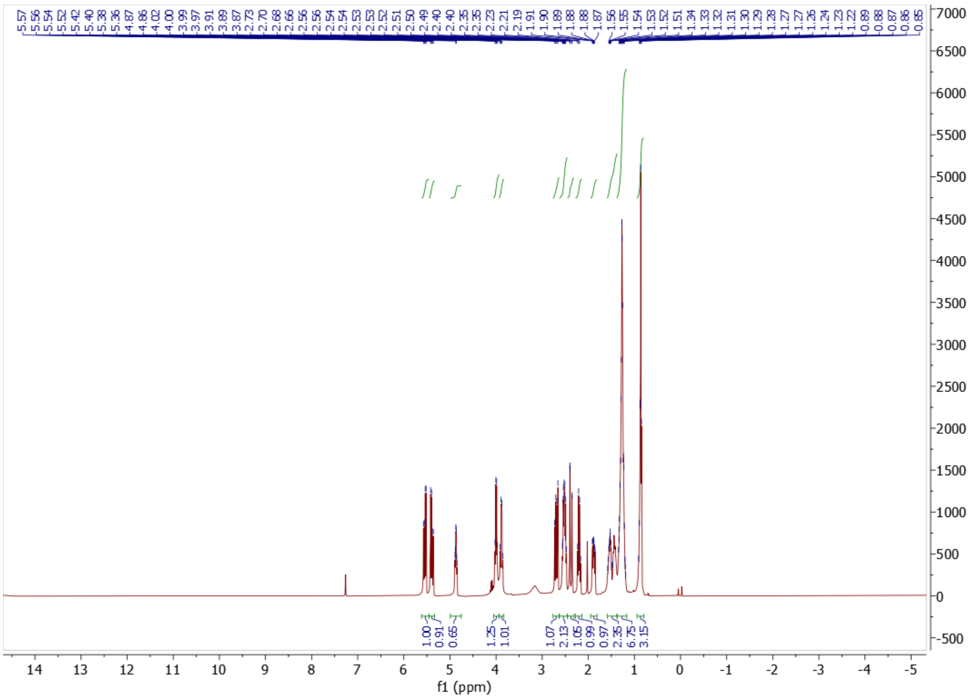

^{13}C NMR (101 MHz, $CDCl_3$) of **17**

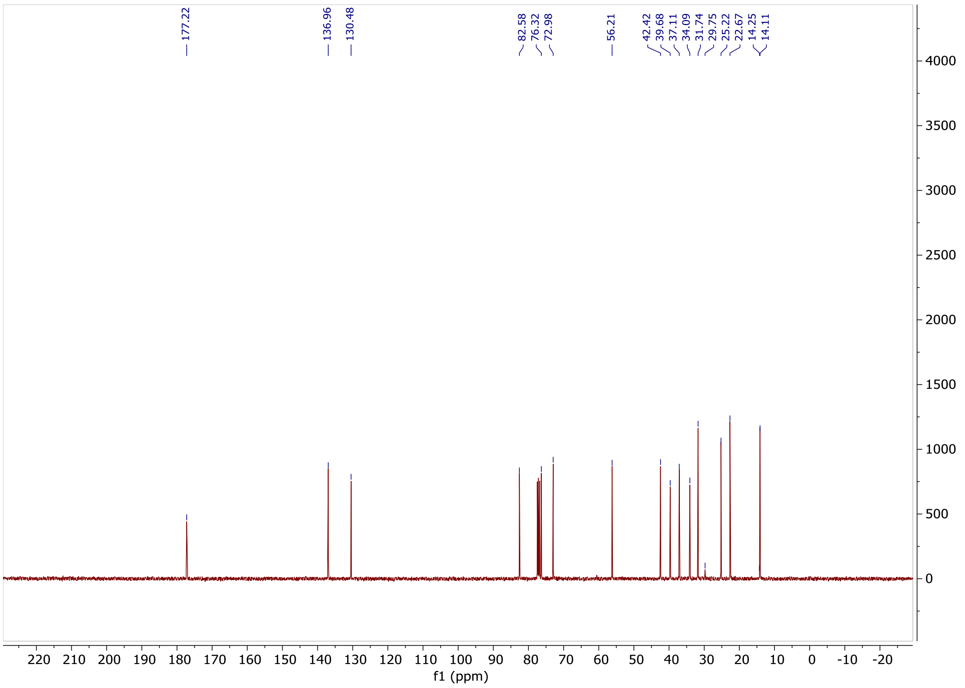

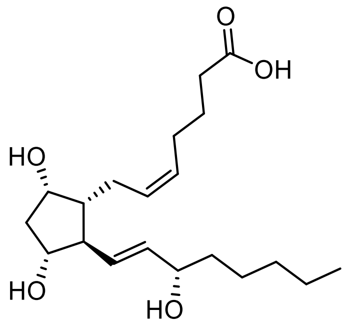

^{1}H NMR (400 MHz, CD_3OD) of PG F2α

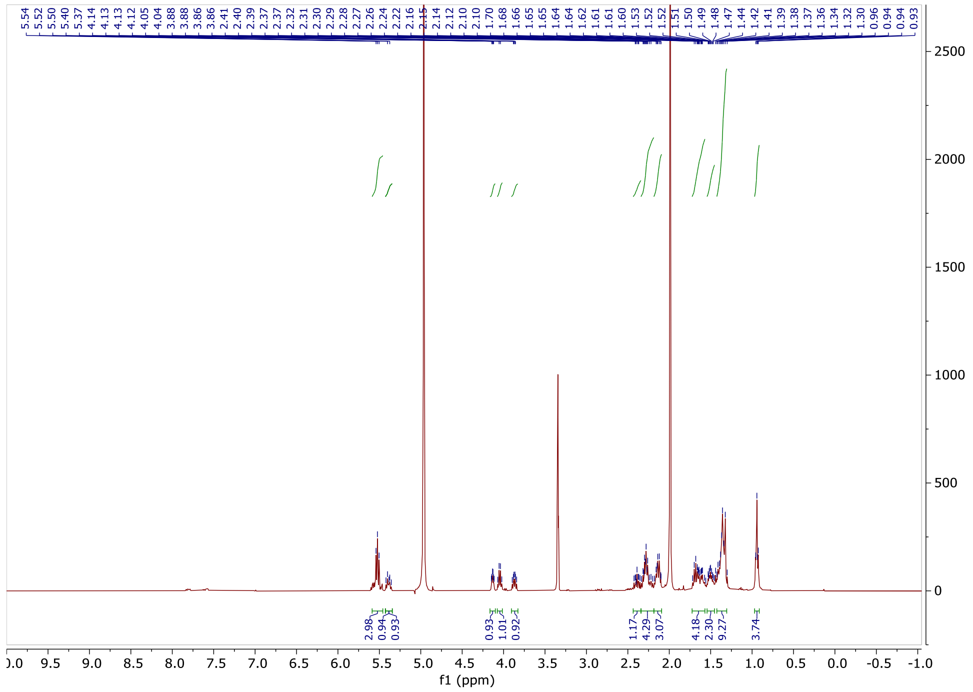

^{13}C NMR (101 MHz, CD_3OD) of PG F2α

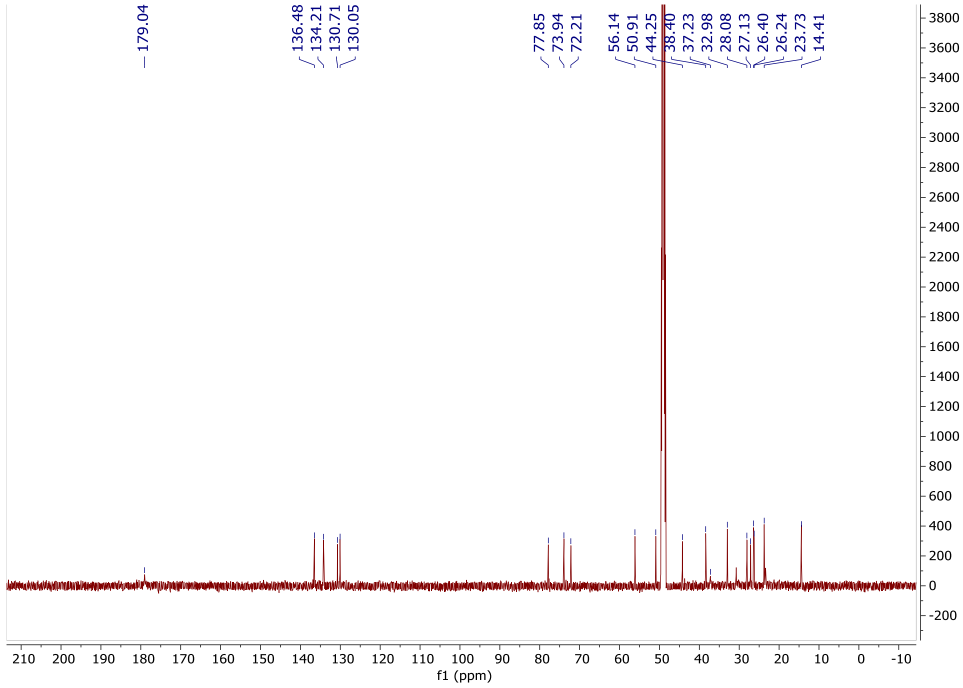

SFC Traces:

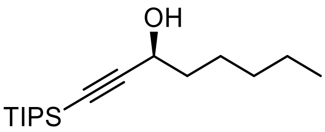

Rac **3a**

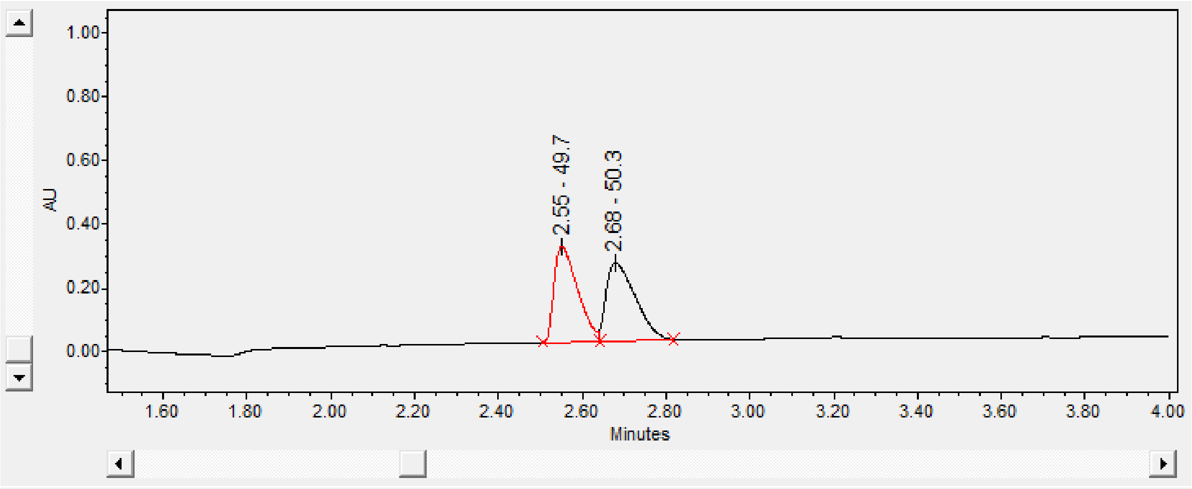

	Name	Retention Time (min)	Area (µV*sec)	% Area	Height (µV)	Int Type	Peak Type
1		2.551	1168443	49.68	300377	bv	Unknown
2		2.678	1183590	50.32	245653	vb	Unknown

Ent **3a**

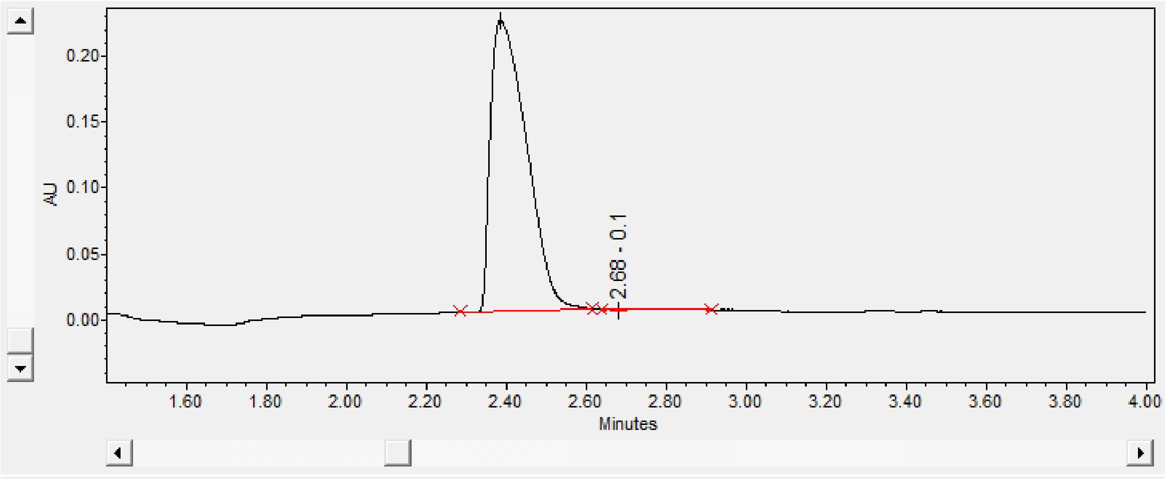

	Name	Retention Time (min)	Area (µV*sec)	% Area	Height (µV)	Int Type	Peak Type
1		2.385	1379605	99.87	220360	bb	Unknown
2		2.680	1796	0.13	-425	bb	Unknown

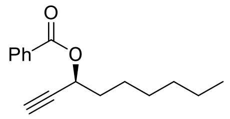

Rac **Bz-S3b**

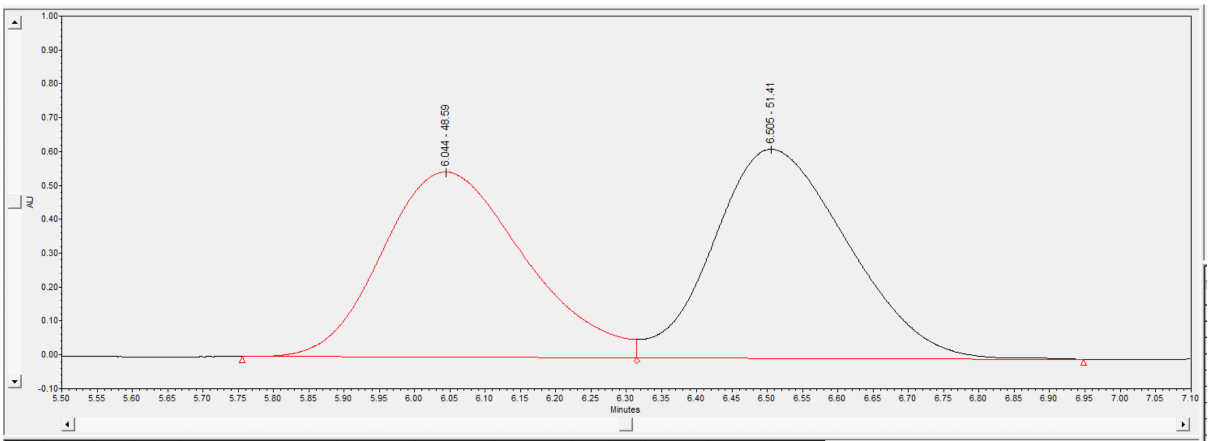

	Name	Retention Time (min)	Purity1 Angle	Purity1 Threshold	PDA/FLR Match1 Spect. Name	PDA/FLR Match1 Angle	PDA/FLR Match1 Threshold	PDA/FLR Match1 Lib. Name	Area (µV*sec)	% Area	Height (µV)	Int Type	Amount	Units	Peak Type	Peak Codes
1		6.044							7682203	48.59	546864	bv			Unknown	
2		6.505							8127617	51.41	617056	vb			Unknown	

Ent **Bz-S3b**

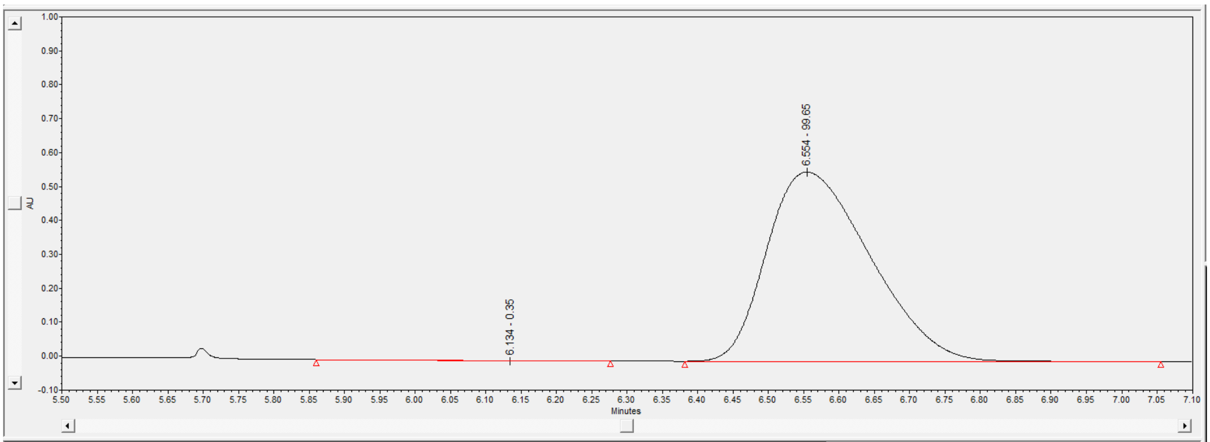

	Name	Retention Time (min)	Purity1 Angle	Purity1 Threshold	PDA/FLR Match1 Spect. Name	PDA/FLR Match1 Angle	PDA/FLR Match1 Threshold	PDA/FLR Match1 Lib. Name	Area (µV*sec)	% Area	Height (µV)	Int Type	Amount	Units	Peak Type	Peak Codes
1		6.134							19930	0.35	-1687	bb			Unknown	
2		6.554							5672318	99.65	558579	bb			Unknown	

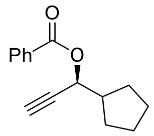

Rac **Bz-S3c**

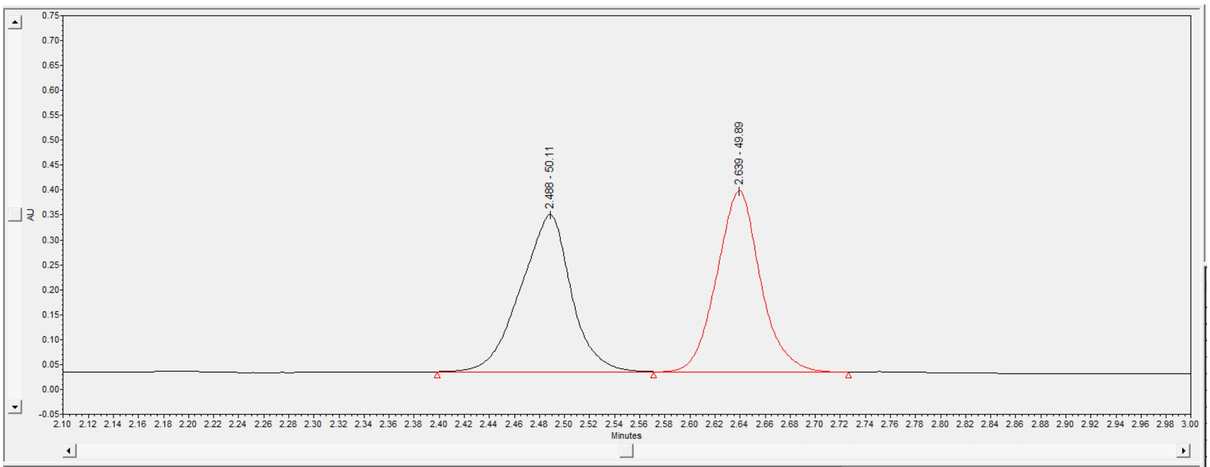

	Name	Retention Time (min)	Purity1 Angle	Purity1 Threshold	PDA/FLR Match1 Spect. Name	PDA/FLR Match1 Angle	PDA/FLR Match1 Threshold	PDA/FLR Match1 Lib. Name	Area (µV*sec)	% Area	Height (µV)	Int Type	Amount	Units	Peak Type	Peak Codes
1		2.488							882292	50.11	315397	bb			Unknown	
2		2.639							878414	49.89	363586	bb			Unknown	

Ent **Bz-S3c**

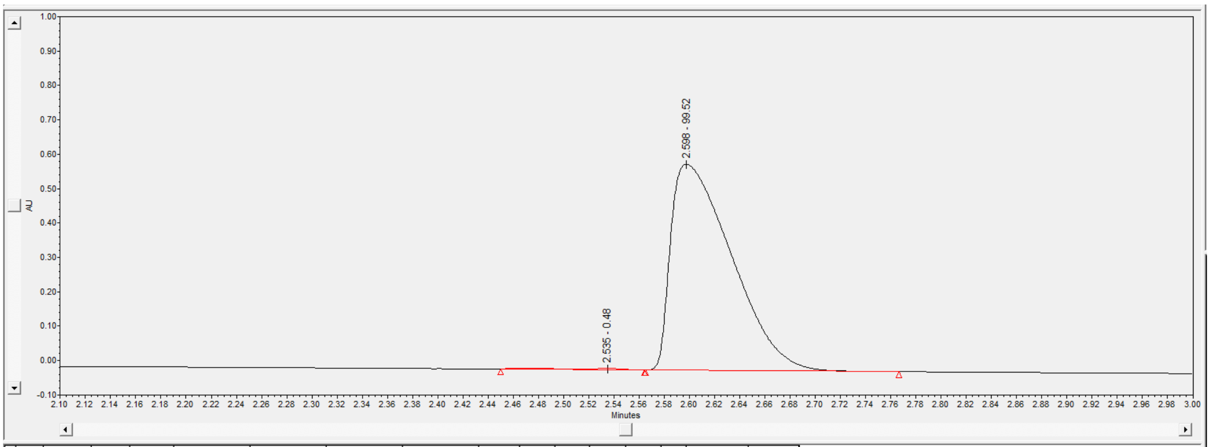

	Name	Retention Time (min)	Purity1 Angle	Purity1 Threshold	PDA/FLR Match1 Spect. Name	PDA/FLR Match1 Angle	PDA/FLR Match1 Threshold	PDA/FLR Match1 Lib. Name	Area (µV*sec)	% Area	Height (µV)	Int Type	Amount	Units	Peak Type	Peak Codes
1		2.535							9796	0.48	3120	bb			Unknown	
2		2.598							2014214	99.52	599515	bb			Unknown	

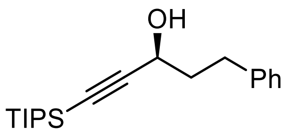

Rac **3d**

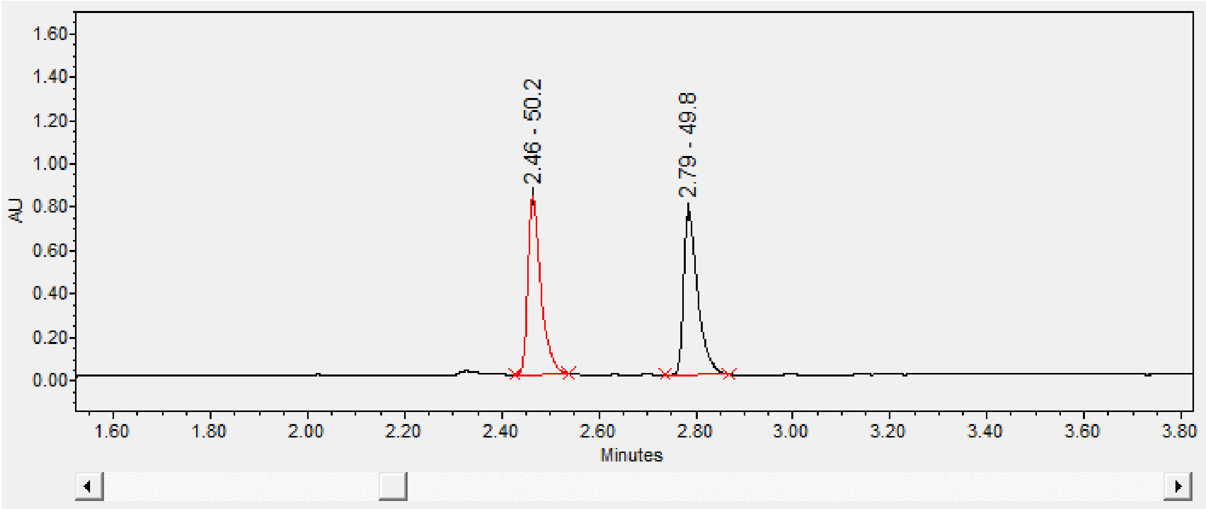

lame	Retention Time (min)	Area (μV*sec)	% Area	Height (μV)	Int Type	Peak Type
	2.464	1537076	50.19	822041	bb	Unknown
	2.785	1525341	49.81	756695	bb	Unknown

Ent **3d**

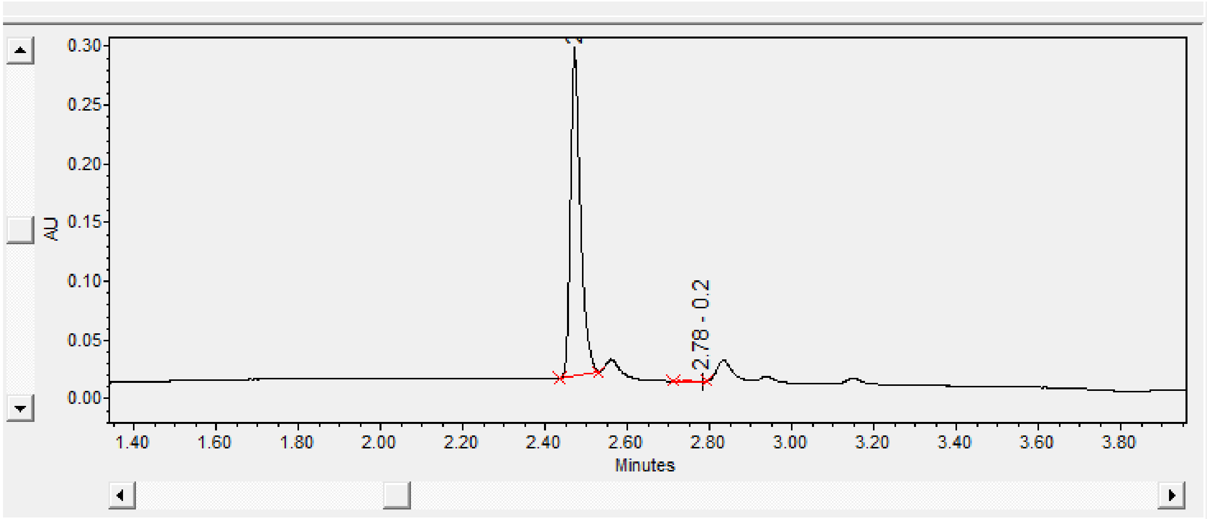

	Name	Retention Time (min)	Area (μV*sec)	% Area	Height (μV)	Int Type	Peak Type
1		2.472	467088	99.84	273270	bb	Unknown
2		2.782	736	0.16	-286	bb	Unknown

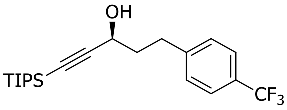

Rac **3e**

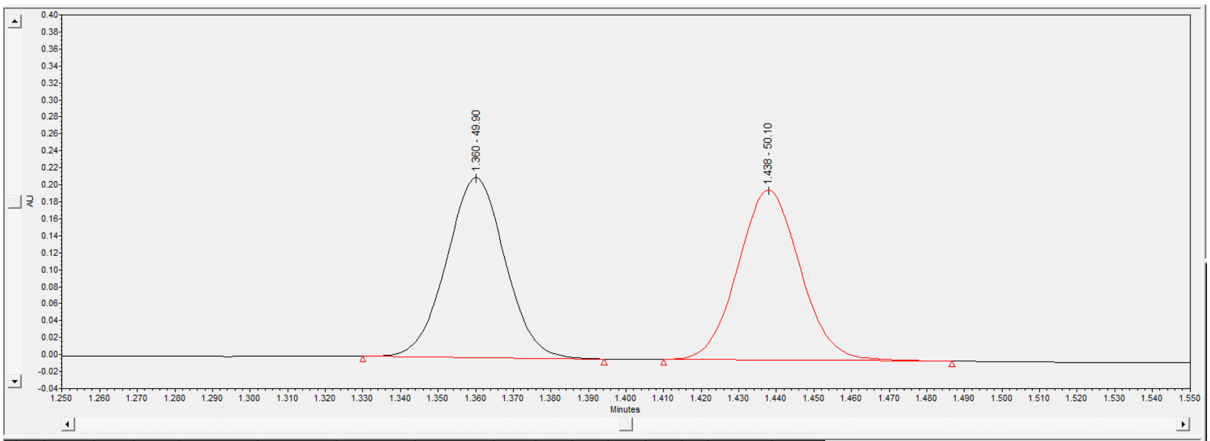

	Name	Retention Time (min)	Purity1 Angle	Purity1 Threshold	PDA/FLR Match1 Spect. Name	PDA/FLR Match1 Angle	PDA/FLR Match1 Threshold	PDA/FLR Match1 Lib. Name	Area (µV*sec)	% Area	Height (µV)	Int Type	Amount	Units	Peak Type	Peak Codes
1		1.360							228497	49.90	212024	bb			Unknown	
2		1.438							229393	50.10	199892	bb			Unknown	

Ent **3e**

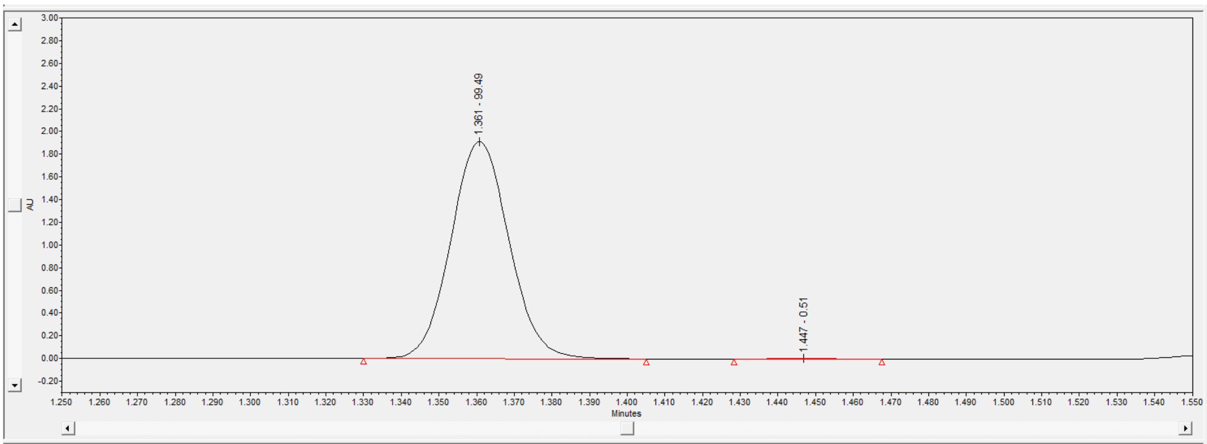

	Name	Retention Time (min)	Purity1 Angle	Purity1 Threshold	PDA/FLR Match1 Spect. Name	PDA/FLR Match1 Angle	PDA/FLR Match1 Threshold	PDA/FLR Match1 Lib. Name	Area (µV*sec)	% Area	Height (µV)	Int Type	Amount	Units	Peak Type	Peak Codes
1		1.361							2044734	99.49	1915342	bb			Unknown	
2		1.447							10558	0.51	9848	bb			Unknown	

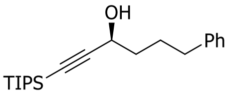

Rac **3f**

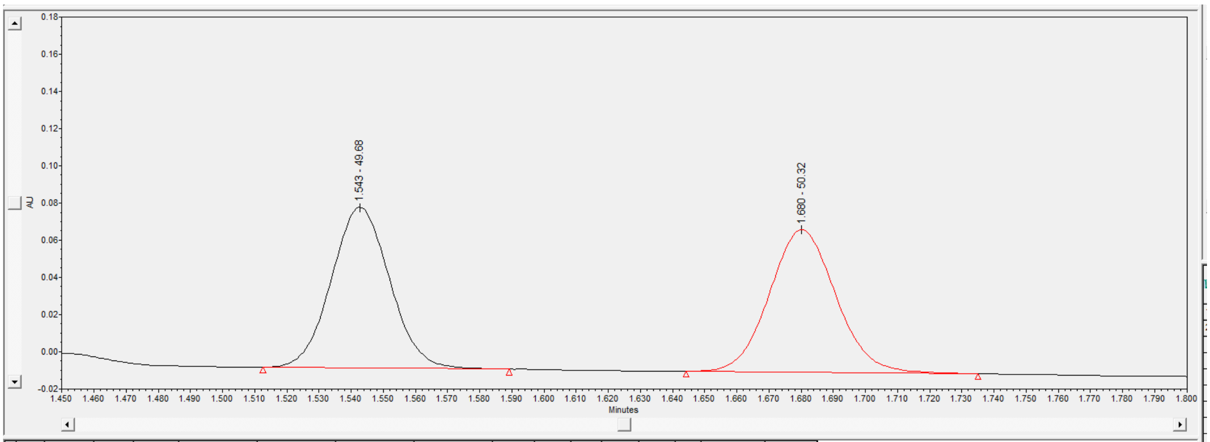

	Name	Retention Time (min)	Purity1 Angle	Purity1 Threshold	PDA/FLR Match1 Spect. Name	PDA/FLR Match1 Angle	PDA/FLR Match1 Threshold	PDA/FLR Match1 Lib. Name	Area (µV*sec)	% Area	Height (µV)	Int Type	Amount	Units	Peak Type	Peak Codes
1		1.543							109150	49.68	86513	bb			Unknown	
2		1.680							110561	50.32	76811	bb			Unknown	

Ent **3f**

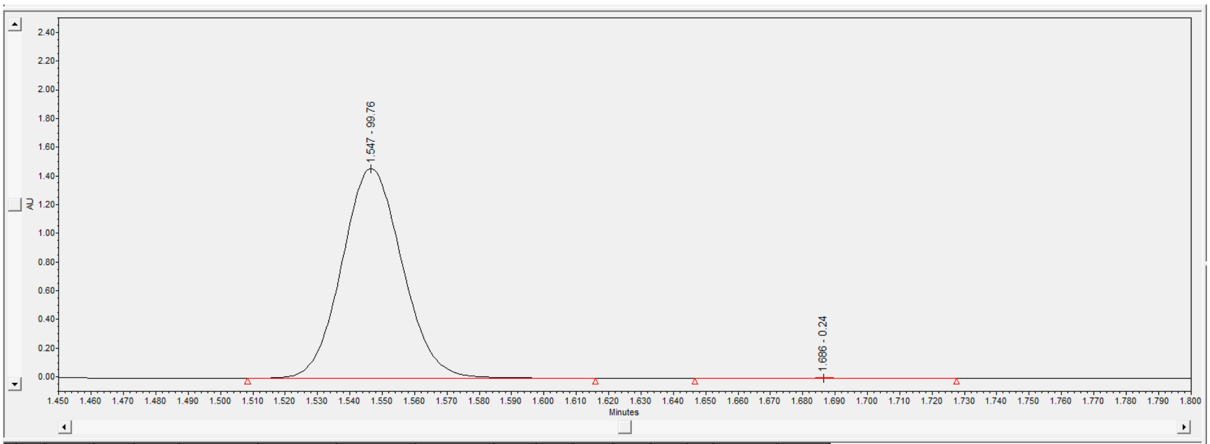

	Name	Retention Time (min)	Purity1 Angle	Purity1 Threshold	PDA/FLR Match1 Spect. Name	PDA/FLR Match1 Angle	PDA/FLR Match1 Threshold	PDA/FLR Match1 Lib. Name	Area (µV*sec)	% Area	Height (µV)	Int Type	Amount	Units	Peak Type	Peak Codes
1		1.547							1889187	99.76	1459363	bb			Unknown	
2		1.686							4536	0.24	3167	bb			Unknown	

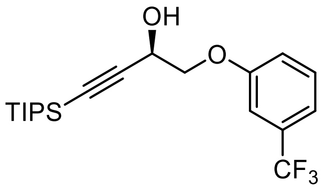

Rac **3g**

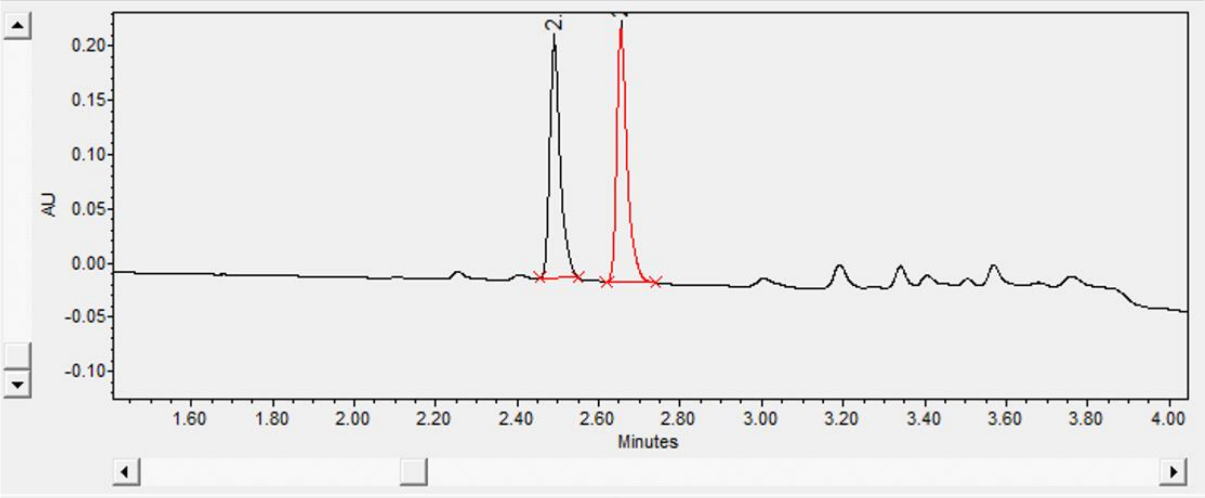

	Name	Retention Time (min)	Area (µV*sec)	% Area	Height (µV)	Int Type	Peak Type
1		2.491	391450	48.55	217827	bb	Unknown
2		2.655	414881	51.45	234289	bb	Unknown

Ent **3g**

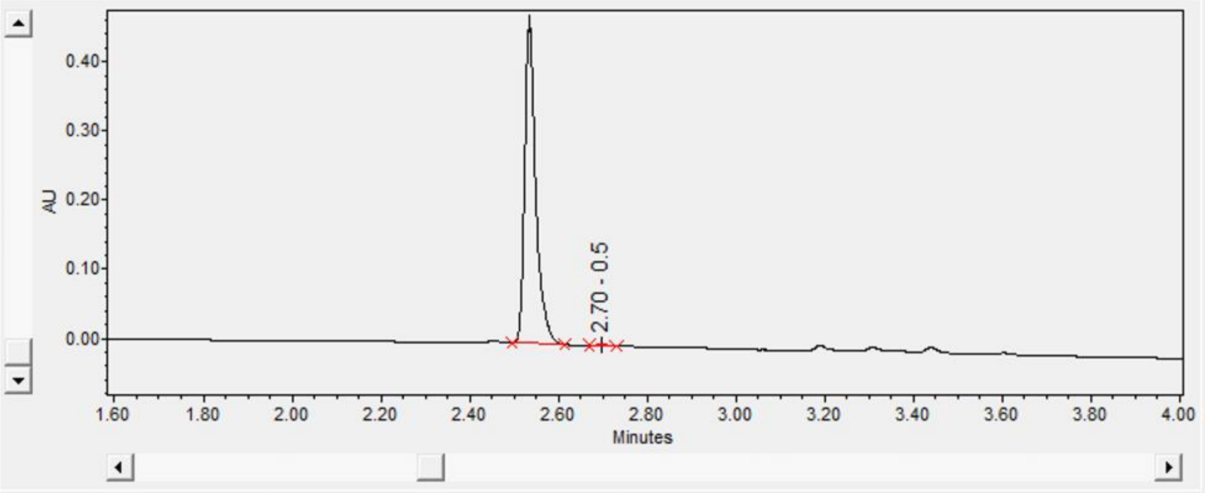

	Name	Retention Time (min)	Area (µV*sec)	% Area	Height (µV)	Int Type	Peak Type
1		2.535	805567	99.47	462780	bb	Unknown
2		2.697	4297	0.53	2765	bb	Unknown

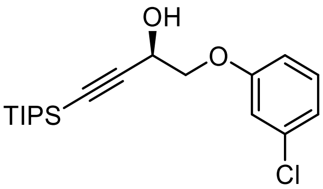

Rac **3h**

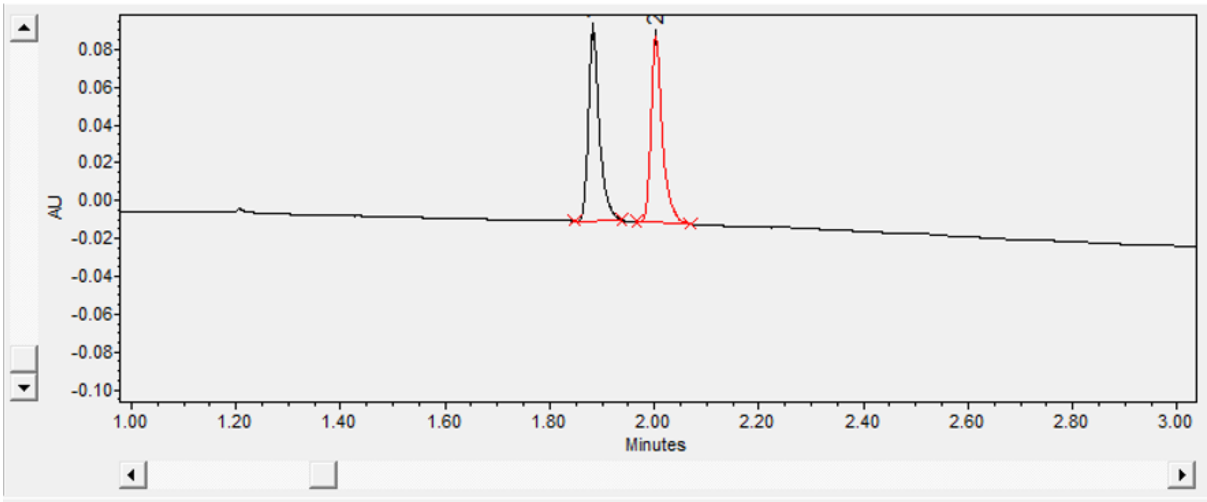

	Name	Retention Time (min)	Area (µV*sec)	% Area	Height (µV)	Int Type	Peak Type
1		1.883	146913	49.57	100337	bb	Unknown
2		2.003	149444	50.43	98593	bb	Unknown

Ent **3h**

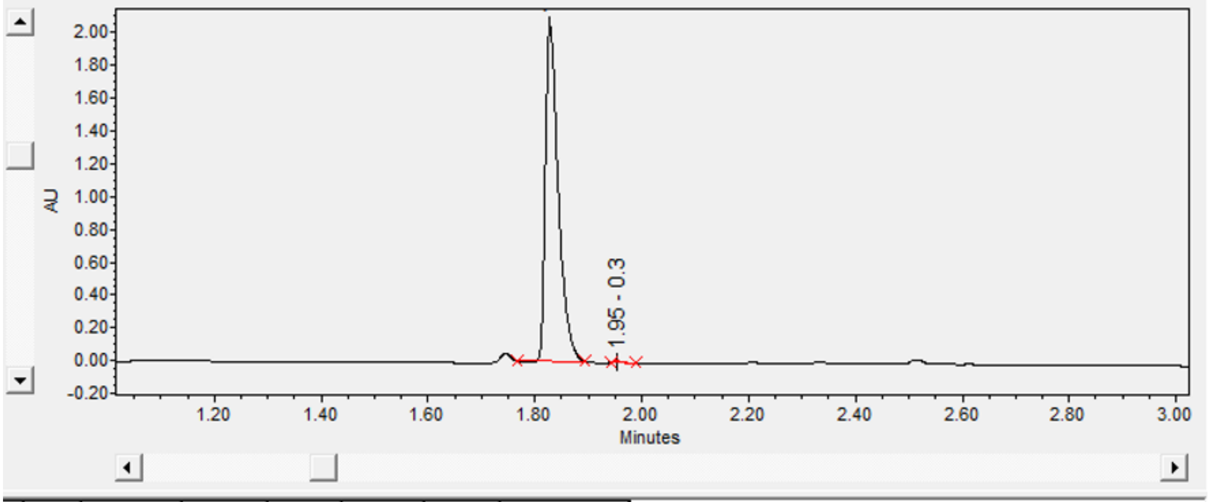

	Name	Retention Time (min)	Area (µV*sec)	% Area	Height (µV)	Int Type	Peak Type
1		1.828	3475642	99.71	2048335	bb	Unknown
2		1.954	9984	0.29	9038	bb	Unknown

References

(1) Zhu, K.; Hu, S.; Liu, M.; Peng, H.; Chen, F.-E. Access to a Key Building Block for the Prostaglandin Family via Stereocontrolled Organocatalytic Baeyer-Villiger Oxidation. *Angew. Chem., Int. Ed.* **2019**, *58*, 9923–9927.

(2) Fleming, I.; Winter, S. B. D. Stereocontrol in Organic Synthesis Using Silicon-Containing Compounds. A Formal Synthesis of Prostaglandins Controlling the Stereochemistry at C-15 Using a Silyl-to-Hydroxy Conversion Following a Stereochemically Convergent Synthesis of an Allylsilane. *J. Chem. Soc. Perkin Trans. 1* **1998**, *17*, 2687–2700.

(3) Davies, J.; Roberts, S. M.; Reynolds, D. P.; Newton, R. F. Enantio-Complementary Total Asymmetric Syntheses of Prostaglandin E2 and Prostaglandin F2α. *J. Chem. Soc. Perkin Trans. 1* **1981**, *2*, 1317–1320.

(4) Coulthard, G.; Erb, W.; Aggarwal, V. K. Stereocontrolled Organocatalytic Synthesis of Prostaglandin PGF2α in Seven Steps. *Nature* **2012**, *489*, 278–281.

(5) Corey, E. J.; Schaaf, T. K.; Huber, W.; Koelliker, U.; Weinshenker, N. M. Total Synthesis of Prostaglandins F2α and E2 as the Naturally Occurring Forms. *J. Am. Chem. Soc.* **1970**, *92*, 397–398.

(6) Sheddan, N. A.; Mulzer, J. Cross Metathesis as a General Strategy for the Synthesis of Prostacyclin and Prostaglandin Analogues. *Org. Lett.* **2006**, *8*, 3101–3104.

(7) Corey, E. J.; Imwinkelried, R.; Pikul, S.; Xiang, Y. Bin. Practical Enantioselective Diels-Alder and Aldol Reactions Using a New Chiral Controller System. *J. Am. Chem. Soc.* **1989**, *111*, 5493–5495.

(8) Corey, E. J.; Weinshenker, N. M.; Schaaf, T. K.; Huber, W. Stereo-Controlled Synthesis of Prostaglandins F2α and E2 (Dl). *J. Am. Chem. Soc.* **1969**, *91*, 5675–5677.
